# Do astigmatid teeth matter: a tribological review of cheliceral chelae in co-occuring mites from UK beehives

**DOI:** 10.1007/s10493-023-00876-2

**Published:** 2024-04-19

**Authors:** Clive E. Bowman

**Affiliations:** Mathematical Institute, University of Oxford, Oxford, OX2 6GG UK

**Keywords:** Biomechanics, Composite tools, Functional ecomorphology, Model system, Path analysis, Velocity ratio

## Abstract

The dentition of the chelal moveable digit in cohabiting astigmatids from UK beehives (i.e., *Carpoglyphus lactis* (Linnaeus), *Glycyphagus domesticus* (DeGeer), and *Tyrophagus putrescentiae* (Schrank)) is characterised for the first time using quantitative tribological measures within a 2D mechanical model. The trophic function of astigmatid chelae are reviewed in terms of macroscopic tools used by humans including hooking devices, pliers, shears, rasps and saws. Comparisons to oribatid claws and isopod dactyli are made. The overall pattern of the moveable digit form of *T. putrescentiae* is not just a uniformly shrunken/swollen version between the other two taxa at either the macro- or micro-scale. Mastication surface macro-roughness values are in the range of international Roughness Grade Numbers N5–N6. The moveable digit of *C. lactis* has low rugosity values compared to the glycyphagid and acarid (which are topographically more similar and match that roughness typical of some coral reef surfaces). *C. lactis* has the most plesiomorphic moveable digit form. The mastication surface of all three species as a chewing tool is distinctly ornamented despite the moveable digit of *C. lactis* looking like a bar-like beam. The latter has more opportunities to be a multifunctional tool behaviourally than the other two species. Little evidence of any differences in the ‘spikiness’ of any ‘toothiness’ is found. Some differences with laboratory cultured specimens are found in *C. lactis* and possibly *T. putrescentiae* suggesting where selection on the digit may be able to occur. The chelal surface of *T. putrescentiae* has been deformed morphologically during evolution the most, that of *C. lactis* the least. Repeated localised surface differentiation is a feature of the moveable digit in *G. domesticus* compared to the likely more concerted changes over certain nearby locations in *T. putrescentiae*. An impactful chelal teeth design is present in *G. domesticus* but this is more equivocal in *T. putrescentiae*. Pockets within the mastication surface of the glycyphagid (and to some extent for the acarid) may produce foodstuff crunch forces of the scale of the chelal tips of oribatids. The moveable digit dentition of *G. domesticus* is adapted to shred foodstuff (like a ripsaw) more than that of the grazing/shearing dentition of *T. putrescentiae*. The collecting ‘picker‘ design of *C. lactis* posterior teeth matches the size of *Bettsia alvei* hyphae which attacks hive-stored pollen. Detritus accumulated in chelal digit gullets through a sawing action matches the smallest observed ingested material. The dentition of *C. lactis* should produce less friction when moving through food material than *G. domesticus*. *C. lactis* is the most hypocarnivorous and may ‘skim’ through fluids when feeding. Astigmatid teeth do matter. The three commensal species can avoid direct competition. Future work is proposed in detail.

## Introduction

**Fig. 1:**
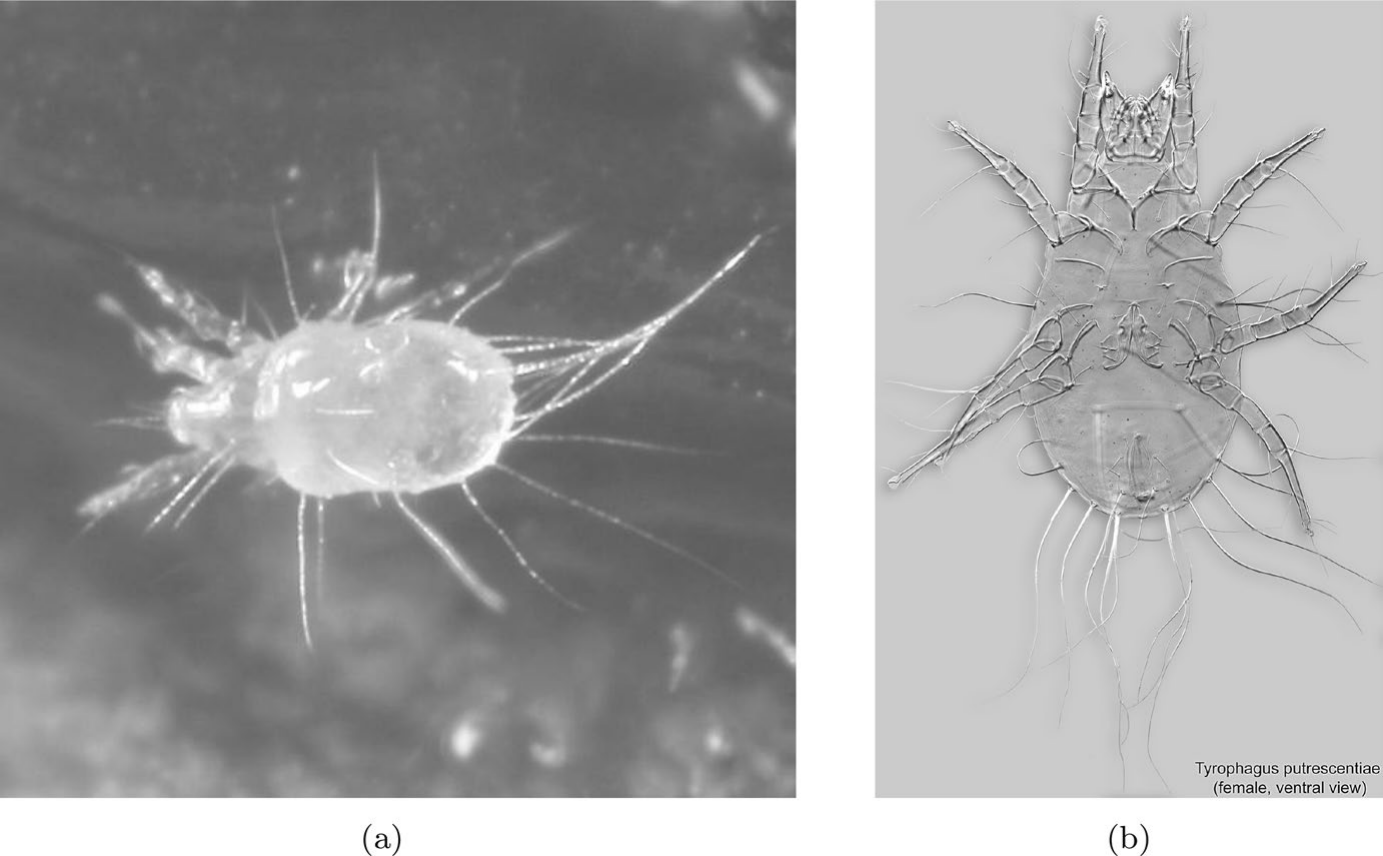
Example acarid astigmatids. **a** *Tyrophagus* sp. in a tortricid moth (probably *Episimus argutanus*) witch-hazel (*Hamamelis virginiana*) leaf-roll, Pelham, Hampshire County, Massachusetts, USA, August 3, 2013 © 2013 Charley Eiseman with permission. **b** *Tyrophagus putrescentiae* female. Note birefringent cheliceral chelae anteriorly. Photo by Pavel Klimov, Bee Mite ID (idtools.org/id/mites/beemites) with permission

**Fig. 2:**
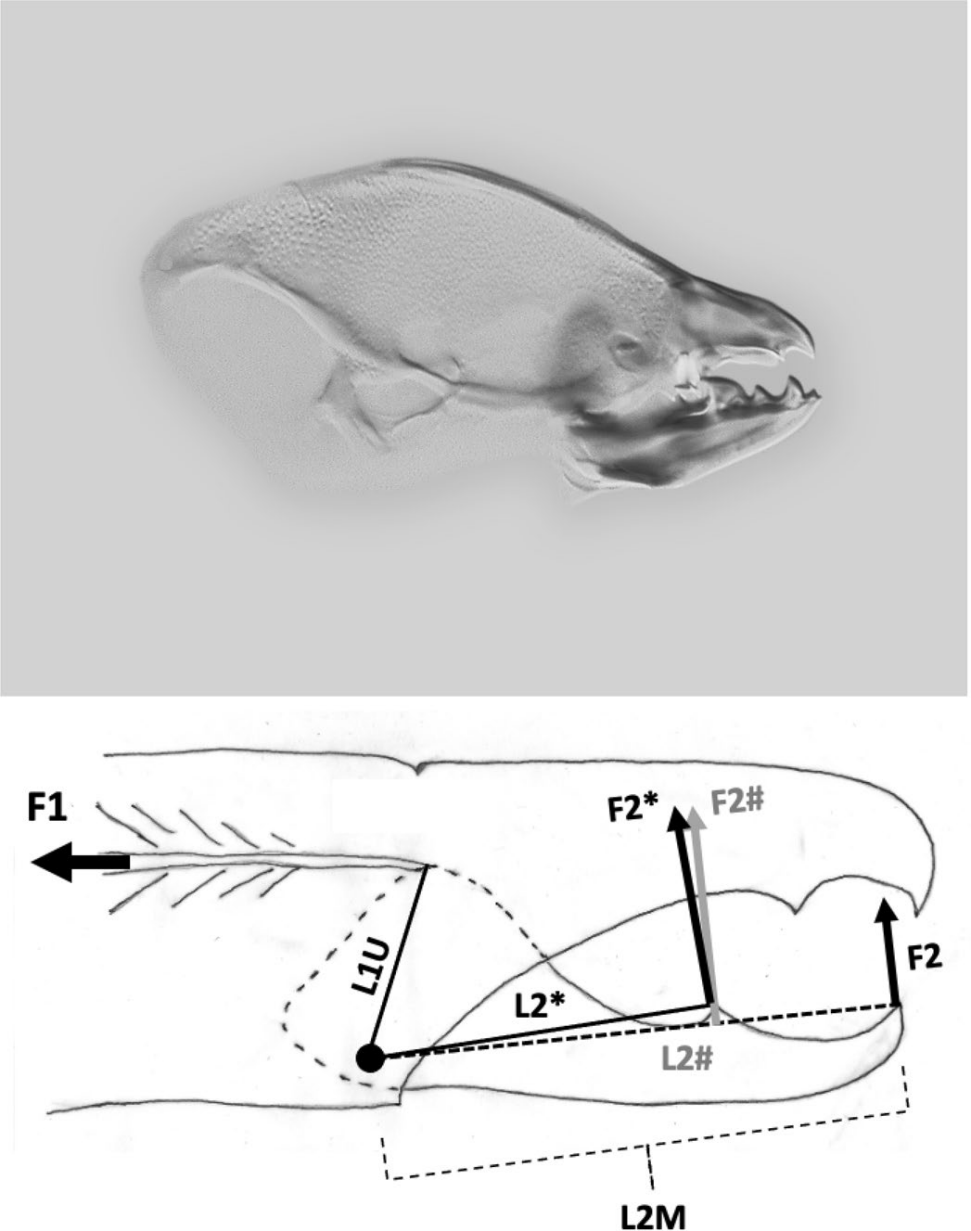
Upper: Enlarged lateral view of a chelicera of *Chaetodactylus krombeini*. Note dentate chela to right end of cheliceral shaft. Tendons and musculature inside the cheliceral base actuate the (lower) moveable digit against the (upper) fixed digit. The gleaming actinochitinous nature of the digits points to their evolutionary origin from setae/ambulacra (Grandjean 1947). From a colour photograph ex Pavel Klimov with permission. *Lower*: Stylised acarine cheliceral chelal statics (amended from a personal drawing by Don Johnston, with permission). The moveable digit tip has a velocity ratio $=\frac{L1U}{L2M}$ (around the condyle, shown as a black circle) and a closing force *F*2 for any applied adductive force *F*1 (appropriately resized by the subtended angle of the tendon). The moveable digit tooth (illustrated as an ‘ornamentation’ at [*x*, *y*] location with respect to the *L*2*M* axis) has a different output moment arm $L2^{\ast}$, a consequent different velocity ratio ($\frac{L1U}{L2^{\ast}}$) and a different crunch force $F2^{\ast}$ (that points slightly posteriorly compared to the direction of *F*2). The location of this asperity along the reference *L*2*M* axis is at $x=L2^{\#}$ which (if there was no height i.e., $y=0$) would induce a different crunch force $F2^{\#}$ (in grey) parallel to *F*2. Note that the upper surface of the moveable digit basally (i.e., proximally to the condyle) rises like the ‘coronoid’ process in vertebrate lower mandibles (Nayak et al. 2015; Kahlon and Agnihotrri 2020)

Extracting nutritive value from potential food-stuffs is a challenge for any animal. Whereas some free-living mites like (most) mesostigmatids only imbibe fluids and rely heavily upon extra-corporeal digestion, cryptostigmatid and astigmatid acarines generally consume solid material. The latter mycophagous (and part herbivorous) arthropods must thus use relatively more internal gut digestive processes after their food material is broken-up orally than fluid feeders. Free-living saprophagous astigmatids like *Tyrophagus* spp. (Fig. 1) masticate their food with chelate chelicerae (Fig. 2). These oral structures act like grasping ‘jaws‘ (Akimov 1985) that gnaw on (and grind Akimov and Oksentyuk 2018) foodstuffs just as vertebrate mandibles/maxillae do triturating the material using their teeth. Dentition, on the basic chelate-dentate cheliceral form, can be regarded as a type of surface ‘roughness’.

How things work matters. Studies of the jaws in other animals has shown that morphological and biomechanical mandibular disparity are decoupled (MacLaren et al. 2017). The mechanical design of chelicerae as a Class I lever producing a static pressure system of forces applied to food (when the chela is in an occlusal or near occlusal position) has recently been compared across a wide variety of laboratory-cultured free-living astigmatids (Bowman 2021c). That review showed that although overall most such astigmatids are designed as generally ‘swollen-sized’ variants of each other, there are macroscopically different oral designs for different lifestyles. For example, *Carpoglyphus lactis* (Linnaeus) was categorised as a fragmentary feeding specialist with chelae of low aspect ratio, weak dentition (Johnston 1965) and feeble crunch force. *Glycyphagus domesticus* (DeGeer) was classed as a typical glycyphagid omnivorous pan-saprophage consuming large, hard food morsels. *Tyrophagus putrescentiae* (Schrank) was seen to be an archetypal generalist microsaprophagous fragmentary feeding acarid. However, Bowman (2021c) in attempting to find biological correlates did not specifically examine any aspects of chelal dentition.

Would these differences be important in the wild? Head shapes can predict feeding habits in fish (e.g., Aguilar-Medrano et al. 2011). In crabs there is a trade off between strength and speed in the function of their chelae (Schenk and Wainwright 2011). Bowman (2023a) recounts how three saprophagous mite species co-occurring in UK beehives can avoid trophic competition with each other by virtue of the differences in the overall mechanical design of their cheliceral chela. This is a useful field-based ‘model system’ to test assertions out on. At least *T. putrescentiae* and *C. lactis* are assumed to be scavengers in bee hives (Okabe et al. 2000). All three species were estimated (by Bowman 2023a) to be able to burrow into a substrate at equivalent to their body size in as short a time as an hour. As effective burrowers, they would be expected to have a small anterior ‘head-like’ region (and thus probably a weak mouthpart bite force) like fossorial worm lizards in order to experience less resistance when digging (Baeckens et al. 2017). Indeed wild-collected *Carpoglyphus lactis* (Carpoglyphidae) did have elongate tweezer-like chelae suitable for processing soft food morsels. However, wild-collected *Glycyphagus domesticus* (Glycyphagidae) had robust chelicerae packing a strong crunch (much like the anomalous mollusc-feeding *Trogonophis wiegmanni* amongst the amphisbaenians). Wild-collected *Tyrophagus putrescentiae* (Acaridae) was somewhere in between in chelal form. Each mite species had teeth on their chelal digits, but again these were not investigated.

Studies in extinct vertebrate animals have found multiple morpho-functional solutions to herbivory in their jaws and teeth (‘regimes’ sensu Button and Zanno 2020). Across diverse lineages (Benson and Barrett 2020), ‘convergent regime 1’ was defined by elongate gracile crania together with reduced and simplified biting surfaces to the jaws and a low relative bite force. If ‘cranium’ was replaced with ‘gnathosoma’, could this what *C. lactis* typifies i.e., a design echoing herbivory in birds and other reptiles? ‘Convergent regime 2’ (also shown in multiple clades) was described by more powerful bite forces at the rear of the jaw, densely packed dentitions with more complex biting surfaces, and mandibles robust to bending and torsion. Could this be what *G. domesticus* typifies, i.e., a design resembling advanced mammalian herbivory? Is *T. putrescentiae* some general purpose intermediate? Since the three mites are of different magnitudes, chelal teeth as chitinous structures would be expected to change in size with any overall swelling/shrinkage of mite form. Departures from this isometry may thus be useful to explain preferred diets.

### A question arises. Do astigmatid teeth matter?

That is, is the overall difference in trophic design between the three UK beehive species confirmed by how their moveable digit teeth (i.e., their dentition) might function comparatively? Could differentiation in dentition (that is mastication surface roughness) be enough to allow competitive co-existence in the bee hive? Further, what macroscopic tool used by humans might the chelae approximate in function?

**Fig. 3:**
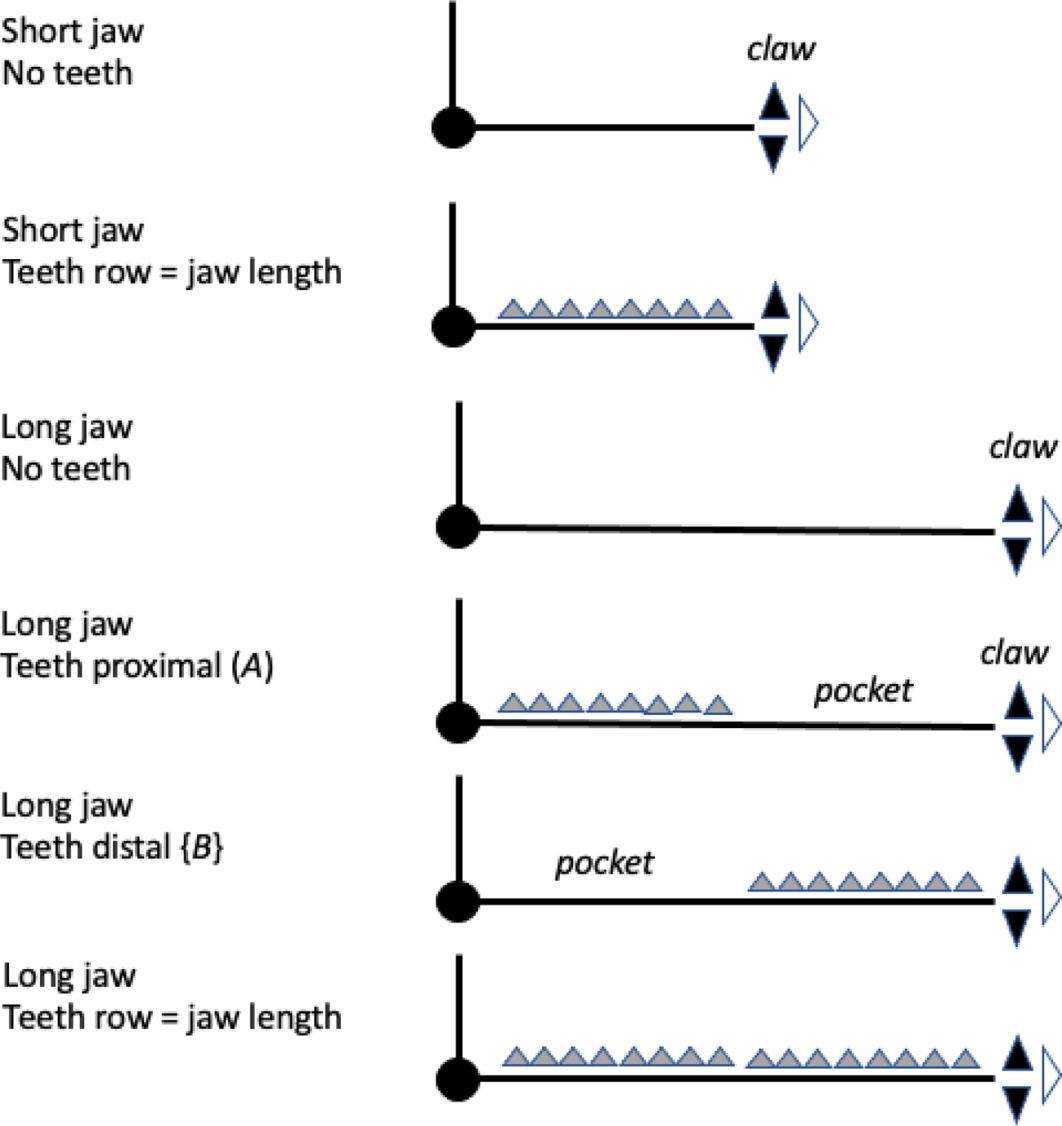
Astigmatid moveable digits stylised as ‘L’-shaped jaws with asperities (grey triangles) separated by tiny gullets (white gaps) all together forming rows of teeth (on a horizontal ramus). Black circle = articulating condyle. Moveable digits can be short or long in length, with or without saw-like teeth along their length and can have a distal stabbing tip (open triangle), a reflexed-up tip (black triangle pointing up) effectively making a ’claw’ behind it, or a reflexed-down tip (black triangle pointing down). For long jaws, teeth may be essentially proximal to the condyle (Type *A*), or distal to it (Type *B*). The lack of teeth ($\equiv$ contiguous gullets) can form a ‘pocket’ which would fill up with food material. Asperities can be merged to form cutting or slicing blades. Distal teeth and a reflexed up chisel-like moveable digit tip are adaptations to tear and bite off chunks for further processing later. Marine algal grazing Galapagos iguanas are examples of a short jaw with teeth row $\approx$ jaw length. Type A matches ‘convergent regime 1’ in non-avian dinosaurs, Type B matches ‘convergent regime 2’ (Benson and Barrett 2020)

In essence, the moveable digit of an astigmatid mite can be considered as a tiny tool comprised of a swinging bar-like beam fixed at one end, adorned with ornamentations (Fig. 3) occluding against a similar fixed digit with analogous opposing structures. Free-living astigmatids have oligodont and heterodont digits (Johnston 1965), which have been informally described and illustrated over many years by various acarologists (e.g., Akimov 1985). Invariably they have a recurved upwards moveable digit tips. The author knows of no species with downward facing ‘tusks’ like the jaw in extinct proboscid vertebrates suitable to strip vegetation or even for digging/rooting (Lucas and Morgan 2008; Lucas and Alvarado 2010). However, a quantitative synthesis as composite tools for different life habits has yet to be attempted for astigmatids.

As Schmähling et al. (2006) say: “In the development and production of industrial parts, both the macroscopic shape and the microstructure of the surface on a μm-scale strongly influence the parts’ properties.“ So the question above can be reframed as, does chelal microstructure (present in these three UK beehive astigmatids) give them any differential trophic gain? In particular as to how the whole might behave as a functional tool. A plesiomorphic assumption of an un-adorned jaw like a rod or flat beam is supported in other animals, for instance the mandible in early reptiles (Crompton and Parkyn 1963). So, the measurable advantage in their own right that teeth (‘asperities‘), gullets (also known when contiguous as ‘pockets‘ or hook-like ‘claws’ herein) and blades ($\equiv$ merged teeth) give to mastication should be by first comparing the actual moveable digit to the properties of a similar bar-like beam without such ornamentations. Only then can cross-species contrasts of dentition be confidently made and the potential non-competitive coexistence of different species in a single habitat safely explained.

Indeed, none of the particular digit elements (teeth, gullets, pockets and blades) should be just considered singly. They all relate to one another and should be taken as a whole to create the best geometry for the foodstuff trituration task at hand. This can be independent of evolutionary modifications at the level of the whole chelicera. That is, there may not only be a degree of independence between body design (*IL*), gnathosomal investment, cheliceral muscle power (*F*1), and chelal design (*VR*) as shown by Bowman (2021b, c, 2023a, b), but also a partial decoupling of digit ornamentation in astigmatid evolution towards different lifestyles. Mathematical arguments are very useful in allowing one to objectively compensate, augment, or offset the positive and negative effects of one element by tweaking another in such tools. These will be used herein to relate mite chelae to the design and operational properties of human-scale tools such as saws (Disston 1907). As Moulton et al. (2023) say in the mathematical modelling of pitcher plants: “..linking form and function enables us to test hypotheses related to the function of features such as shape and ornamentation...”.

**Fig. 4:**
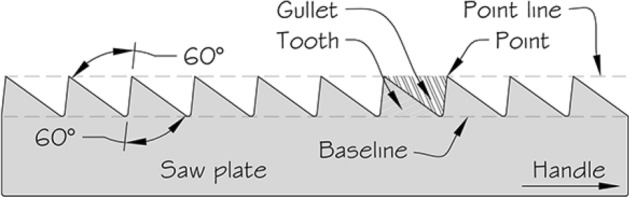
Basic saw tooth nomenclature (©Isaac Smith 2012 with permission)

Basic saw tooth nomenclature can be found in Fig. 4. Note that conventional Western-style saws are pushed to effect a cut whereas the Japanese-style of saw is pulled. Typically, if the teeth point away from the handle, a saw cuts on the ‘push’ stroke. Generally, push stroke saws are designed for cutting through tougher materials as it is easier to exert pressure on the saw when pushing it rather than pulling it. Typically, if the teeth point back towards the handle, the saw cuts on the ‘pull’ stroke. Generally, pull saws have thinner blades which are designed for making more delicate and precise cuts. As well as this, the motion of pulling the saw towards one rather than pushing it gives the human user more control over each stroke of the saw. This makes it easier to cut in a straight line and achieve a neat finish. The blade on a pull stroke saw is generally thinner and more delicate. As a result, greater care should be taken when using this type of saw to ensure one does not damage the blade. Saws that cut on both the push and pull stroke are designed for fast aggressive sawing, and will cut and remove more material with each stroke. As a result, they will shred material and be much less likely to produce a neat finish. On such saws, typically, the teeth are not angled backwards or forwards, and instead, point straight down. To what extent can these macro-scale observations be applied to any micro-scale trophic adaptations of the three astigmatid species to further explain their co-existence in UK beehives? In holding and cutting foodstuff do mite teeth also saw through it as the food is pulled to and fro by the gnathosoma?

## Aim

Using the comparison of field-collected specimens from the same origin of the three commonly found UK beehive astigmatid mites *Carpoglyphus lactis*, *Glycyphagus domesticus*, and *Tyrophagus putrescentiae*, this review seeks to show how the two dimensional profile of the tooth, gullet and blade-like form of the mastication surface on their moveable digits (i.e., their morphology) varies in a rational way. Further to test if this advantage as a tool is more than would be expected by chance across these three species. An attempt will also be made to assess the scale of developmental change needed to form the moveable digit dentition during evolution.

The first step in analysing the mechanics of a surface contacting another (like the moveable digit touching foodstuff) is their characterisation (Tavares 2015). Four concepts: Velocity ratio; Mastication surface; Average velocity ratio; and Variance of velocity ratio, will be key in determining the Expected results when mapping morphology to practical function. These are all elaborated in the Explanatory Appendix. The three concepts form the basis of the Hypothesis tests used. The main tests will be by *z*, *t*, $\chi ^2$ and *F*-tests of the actual observed versus expected moveable digit feature sizes, velocity ratios and their variances given that moveable digit tip velocity ratio. This conditional test needs to be in the context of what the arrangement along the moveable digit looks like quantitatively if there had been no evolutionary differentiation at all (i.e., where the moveable digit is assumed to be an un-ornamented bar-like beam of uniform density in form, itself subject to possibly different evolutionary pressures at the whole cheliceral scale). So, this main test will be supported by *z*, *t*, $\chi ^2$ and *F*-tests of the expected versus theoretically expected moveable digit feature sizes, velocity ratios and their variances given that moveable digit tip velocity ratio.

Although chelae may appear simple at first glance, closer inspection reveals multiple cutting edges in different planes on different features at different physical scales arranged in different ways (i.e., there are digit model types), so a variety of simple quantitative descriptors of digit surface waviness or macro-roughness using tribology (Bhushan 2000) for the first time will also be used. Tribology is a system science that deals with parts in relative motion, and related friction, adhesion, lubrication and wear phenomena (Gebeshuber and Gordon 2011). At the very similar length scales of the three beehive inhabitants, sophisticated fractal modelling (Zahouani et al. 1998) is left for future work.

## Materials and methods

**Fig. 5:**
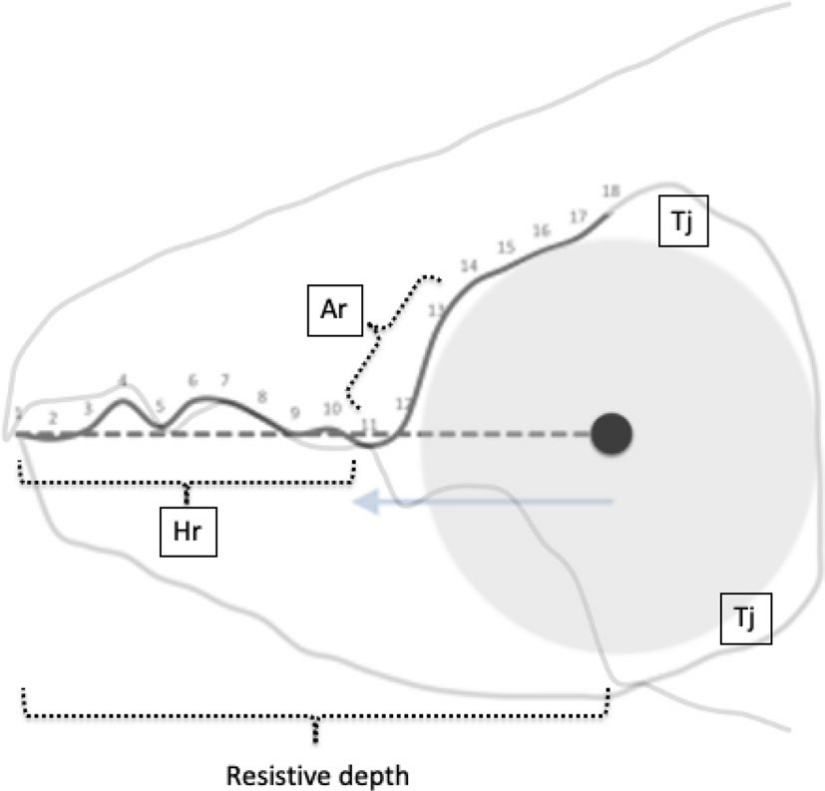
Nomenclature mapping for astigmatid moveable digit. Illustrated with larger female *Tyrolichus casei* (Oudemans) chelicera for clarity. Hr = horizontal ramus with teeth and gullets forming mastication surface (or ‘potential tooth row’ and ends at $x_{11}$). Ar = ascending ramus of coronoid process (hyperbolic-shaped, posterior of mastication surface, extends variably towards semi-landmark 18). Grey area = basal ramus body of coronoid process. Tj = tendon junctions (dorsal adductive, ventral abductive). Lower part of moveable digit deepened to afford resistance to reactive forces on chelal occlusion (Bowman 2023a). Black circle = condyle. Dashed line = output moment lever arm (*L*2*M*). Heavy black line = moveable digit profile of semi-landmarks (2–18). Large grey arrow = length of adductive lever moment arm *L*1*U* (see Bowman 2021c) forwards from condyle in *L*2*M* direction. Adductive tendon inserts on coronoid process just posterior of semi-landmark 18

The same 52 female specimens of *Carpoglyphus lactis*, *Glycyphagus domesticus* and *Tyrophagus putrescentiae* as investigated by Bowman (2023a) and the microscopical methods therein were used. Two landmarks (moveable digit tip, condyle) and 17 semi-landmarks (numbered as in Fig. 5) were assayed.

The nomenclature for astigmatid mouthparts is summarised in Johnston (1965). Fig. 5 maps the terminology used for animal jaws onto their use for an astigmatid chelicera.

Twenty specimen samples from laboratory cultures (Ca4, G5 and T13 listed in Bowman 2021c) were used for informal comparison where relevant. T13 belongs to Griffith’s breeding group ‘A’ for which an *ad hoc* small heterogeneous sample of seventeen female museum specimens was also available (Table 1).

**Table 1:** Details of *Tyrophagus putrescentiae*—museum specimens

Label	Where Found		Date	D1	D2	L2
ACY77/237 upper	Heathcote, NSW, Australia	Galls on flowers of *Acacia longifolia*	9 Nov 1976	34.0	92.2	35.6
ACY77/237 lower	Heathcote, NSW, Australia	Galls on flowers of *Acacia longifolia*	9 Nov 1976	30.3	101.4	48.1
820322-3C upper	Liverpool GB, (m.v. “Anita”, LIV45.82)	French rape seed ex Tonnay Chareste	16 Mar 1982	33.5	104.2	41.6
820322-3C lower	Liverpool GB, (m.v. “Anita”, LIV45.82)	French rape seed ex Tonnay Chareste	16 Mar 1982	34.0	108.0	41.3
820322-2(B) left	Liverpool GB, (Lennox Whschpool, LIV46.82B)	Mouldy damp Indian walnut kernels	5 Mar 1982	47.3	125.9	46.7
820322-2(B) right	Liverpool GB, (Lennox Whschpool, LIV46.82B)	Mouldy damp Indian walnut kernels	5 Mar 1982	47.7	120.8	50.5
820816-2B upper	Merrionnydd District Council GB, (LIV139.82B)	From house kitchen of converted bakery	9 Aug 1982	45.4	118.0	42.0
820816-2B lower	Merrionnydd District Council GB, (LIV139.82B)	From house kitchen of converted bakery	9 Aug 1982	36.3	117.2	45.1
820816-2G upper	Merrionnydd District Council GB, (LIV139.82G)	From house kitchen of converted bakery	9 Aug 1982	32.3	96.7	45.8
820816-2G lower	Merrionnydd District Council GB, (LIV139.82G)	From house kitchen of converted bakery	9 Aug 1982	35.1	98.1	49.6
820816-2H left	Merrionnydd District Council GB, (LIV139.82H)	From house kitchen of converted bakery	9 Aug 1982	36.3	127.9	46.3
820816-2H right	Merrionnydd District Council GB, (LIV139.82H)	From house kitchen of converted bakery	9 Aug 1982	39.4	119.8	41.0
820406-4-M6842	Liverpool Docks, GB	<not recorded>	1982	37.6	107.2	44.0
820406-4-M6844	Liverpool Docks, GB	<not recorded>	1982	36.4	122.0	44.7
820406-4-M5855	Liverpool Docks, GB	<not recorded>	1982	34.3	107.0	39.4
820406-4-M6313-ST24a	Liverpool Docks, GB	<not recorded>	1982	33.9	110.3	41.4
820406-4-M6313-ST24b	Liverpool Docks, GB	<not recorded>	1982	29.5	115.1	45.2
Summary				37.1 (5.29)	112.7 (10.54)	43.3 (4.68)

All were classified as representing isolated breeding group ‘A’ of Griffiths (1979). Dorsal setal lengths in μm (as averages of left and right sidea). Summary is mean with (SD) underneath it

**Fig. 6:**
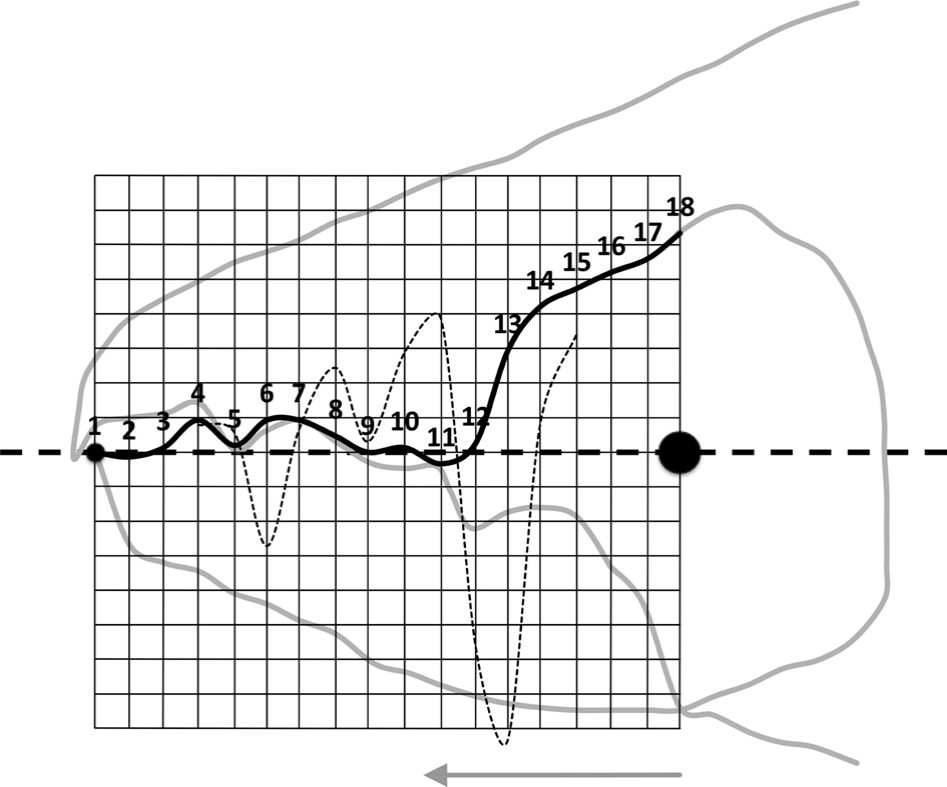
Measurement scheme illustrated with larger female *Tyrolichus casei* (Oudemans) chelicera for clarity, scaled to match standard grid overlay. Small black dot = moveable digit tip (landmark 1). Large black dot = condyle. Dashed line orientation to adductive moment lever arm (*L*2*M*). Heavy black line = moveable digit profile of semi-landmarks (2–18). Dotted line = profile jerk. Large grey arrow = length of adductive lever moment arm *L*1*U* (see Bowman 2021c) forwards from condyle in *L*2*M* direction. Note tooth around semi-landmark 4, gullet around semi-landmark 5 and blade (semi-landmarks 6 onwards) finishing approximately at an equivalent distance to *L*1*U* from the condyle (i.e., after semi-landmark 11) where moveable digit profile jerk is at a maximum

The cheliceral chelal moveable digits were characterised on a standard grid (Fig. 6).

Analyses were done in Excel2011 and R version 3.4.4 (2018-03-15) using untransformed data. Heat-maps and 3D plots used Graphis 2.7.3. For an item ‘A’ or ‘ABC’, ${\hat{A}}$ or ${\widehat{ABC}}$ indicates estimates of A or ABC respectively derived from the observed data.

### Quantitative analysis

**Fig. 7:**
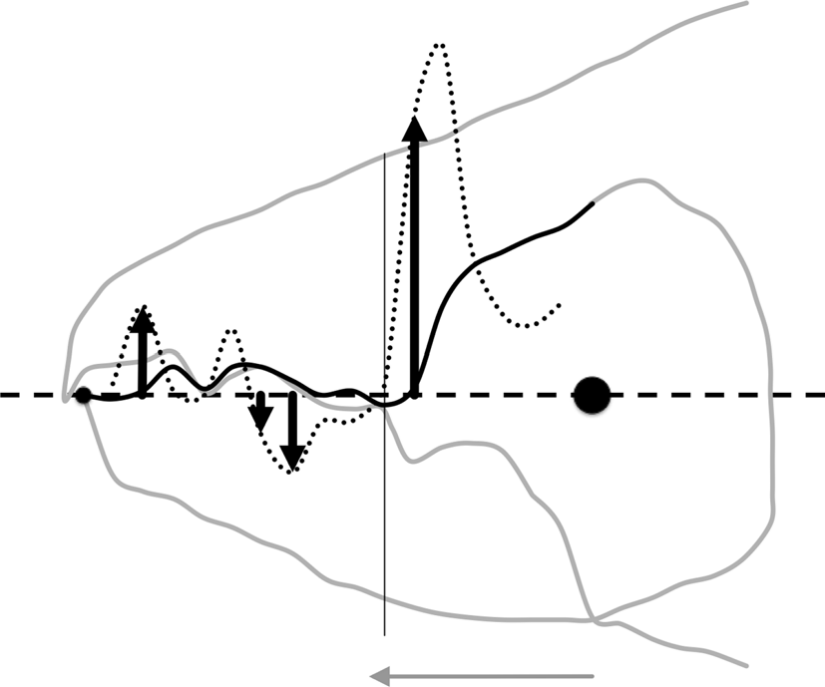
Deflection of *L*2*M* basis producing elongate mastication surface (larger female *Tyrolichus casei* (Oudemans) chelicera for clarity). Dotted line is gradient or slope (deflection) of the moveable digit profile at that point. Bold black arrows show notable locations and magnitude of flexing of moveable digit surface ($f_i$ at $x_{i}\quad i=3,7,8,12$) compared to a beam-like basis. Such bending induces a local slope change from the horizontal. Note that the end of the mastication surface (at $x_{11}$) is around the place where only *L*1*U* worth of digit length remains back to the condyle and this is just ahead of the area of maximum deflection. Adductive tendon inserts on coronoid process just posterior of semi-landmark 18

**Fig. 8:**
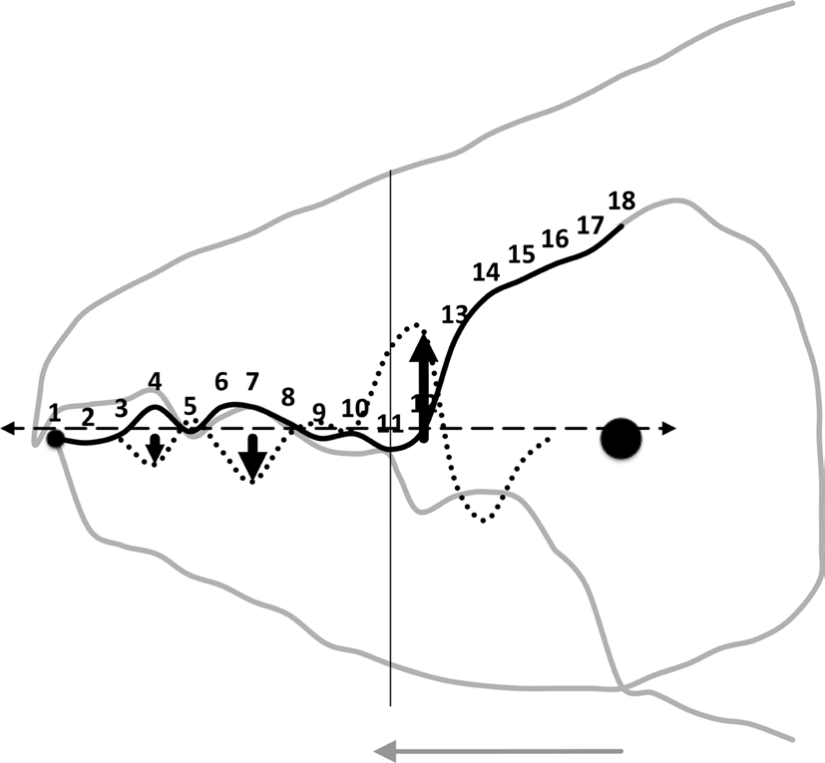
Example of smoothness ($var(curvature) = 0.1428$) of moveable digit dentition. Curvature of moveable digit profile plotted as dotted line (larger female *Tyrolichus casei* (Oudemans) chelicera for clarity). Note end of mastication surface (at $x_{11}$) is around place where only *L*1*U* worth of digit length remaining to condyle. Locations $x_{i}\; i=4, 7, 12$ show largest magnitude of shear or creep of surface ($h_{i}$). Distal chelal tooth at $i=4$ and medial broad tooth ($i=7$) clear from pattern of curvature. Moveable digit’s coronoid-like swelling (see Bowman 2021b) clear for $i=13{-}18$. Horizontal double arrow headed dashed line indicates *avg*(*y*) value $= 0.54$ for the mastication surface. Adductive tendon inserts on coronoid process just posterior of semi-landmark 18

As the first and second derivatives play a fundamental role in the study of the behaviour of a curve near a point (Schot 1978a), using each profile set of moveable digit measurements ($[x_{i}, y_{i}], \quad i=1 \ldots 18$) from Bowman (2023a), a series of sample derived estimates were calculated:

- velocity at $x_{i}$, or gradient $g_{i}=\frac{dy_{i}}{dx_{i}}=\frac{y_{i+1}-y_{i-1}}{x_{i+1}-x_{i-1}},\quad i=2\ldots17$ (Fig. 7)
- acceleration at $x_{i}$, or curvature $c_{i}=\frac{d^{2}y_{i}}{dx_{i}^{2}}=\frac{g_{i+1}-g_{i-1}}{x_{i+1}-x_{i-1}},\quad i=3\ldots16$ (Fig. 8)
- start of the chelal mastication surface, taken to be the tip of the moveable digit, $x_{1}$ (Fig. 6)
- end of the mastication surface $e=$ that $x_{i}$ where $c_{i}$ was at a maximum, given $y_{i}$ thereafter is monotonically increasing (Fig. 8). This marks the index $i_{e}$ at the rise of the coronoid-like process (i.e., the ‘ascending ramus‘) of the moveable digit (Bowman 2021b) and was invariably at that point along the *L*2*M* beam-like axis corresponding to a distance of *L*1*U* units from the condyle. This was in the same area as the maximum estimated ‘jerk’ (Sandin 1990) $\frac{d^{3}y_{i}}{dx_{i}^{3}}=\frac{c_{i+1}-c_{i-1}}{x_{i+1}-x_{i-1}},\quad i=4\ldots15$ — Fig. 6
- length of actual mastication surface $m=\sum _{i=2}^{i_{e}}\sqrt{(x_{i}-x_{i-1})^{2}+(y_{i}-y_{i-1})^{2}}$
- smoothness *s* = variance of curvature $c_{i}$ over the mastication surface (Fig. 8)

The mastication surface is described further in the Explanatory Appendix. Note that the actual length of the mastication surface subject to friction on any foodstuff is always going to be greater than the projection of the profile (shown in black in Fig. 1) onto the *L*2*M* beam-like axis (i.e., the dashed line between the two black circles in Fig. 1) that it covers (think of it as draping a thin piece of string over the peaks and troughs of the profile through to $x_{i_{e}}$). This elongation is a consequence of the deflection (as measured by $|y_{i}|$) of the profile from its *L*2*M* basis of $y=0$. This total ‘drape distance‘ (called ‘chain distance’ when used similarly by oceanographers) in Fig. 2 of Bowman (2023a) is $m= 20.3$ μm for example. Determining the length of an irregular arc segment by approximating the arc segment as connected (straight) line chordal segments is also called curve rectification.

Variance of curvatures is a standard measured criterion for surface smoothness. The smoothness of the gradient profile ($g_{i},\quad i=2\ldots17$) is the variance of its own curvature i.e., the variance of the ‘jerk’ on the original profile measures. As jerk (also known as jolt, surge or lurch) is a predictor of large accelerations of short scope (Schot 1978b) it is consilient that this is where the mastication surface abruptly ends and the profile of the ‘ascending ramus‘ of the coronoid process suddenly begins. The area of maximum moveable digit profile curvature invariably followed that of the location of maximum jerk. Jerk is taken to indicate the location of a major morphological change in the moveable digit profile form.

Each rise and fall of the upper moveable digit surface profile has its own velocity ratio value ($VR_{i}$, leading to a different *F*2, illustrated as Fig. 2 in Bowman 2023a). The hypotenuse between the $[x_{i},y_{i}]$ location and the condyle is given by $m_{i}=\sqrt{(x_{i})^{2}+(y_{i})^{2}}$ meaning that the velocity ratio at each location is $VR_{i}=\frac{L1U}{m_{i}}$. Averaging these over the mastication surface gives the observed average velocity ratio $O[VR]=\frac{1}{e}\cdot \sum _{i=1}^{i_{e}}VR_{i}$—see Explanatory Appendix.

### Tribological analysis

Eighteen (including the condyle) measurement positions suggest a maximum of six discrete features (i.e., teeth or gullets) could be detected before the anticipated rise of the coronoid process of the moveable digit i.e., a profile of [0, down, up, down, up, down, up, down, up, down, up, down, up, up,up,up,up,up] or, [0, up, down, up, down, up, down, up, down, up, down, up, up, up,up,up,up,up] with respect to the moveable digit tip—see Fig. 6. It is acknowledged that this grid spacing is a limit upon the fine detail of the profile that can be captured (since a feature must cover 3 increments) and that one might be unlucky to miss a particular surface plication at this granularity, but it was a practical compromise with respect to time taken for data capture. The roughness wavelength (aka ‘cutoff‘ $\lambda$c) is thus 3 increments. Herein, this tribological evaluation length ($\lambda$c) has been effectively standardised by the sampling length *L*2*M*. It is thus understood that a grid space of 1 indicates a slightly different actual spacing in μm for different specimens, but this is not about defining universal landmarks rather it is to deploy digit length adjusted semi-landmarks comparable across chelal designs.

Using the notation of Bhushan (2000), standard industrial tribological measures (taking *L*2*M* as the tribological reference line) were estimated as follows *over just the mastication surface*:

- $$R_{a}=CLA=AA=\tfrac{1}{i_{e}}\cdot \sum _{i=1}^{i_{e}}|y_{i}-avg(y)|$$ (where $$avg(y)=\tfrac{1}{i_{e}}\cdot \sum _{i=1}^{i_{e}}y_{i})$$
- $$R_{q}= \text {Root\; Mean\; Square}=\sqrt{\tfrac{i}{i_{e}}\cdot \sum _{i=1}^{i_{e}}(y_{i})^2}$$
- $$\sigma ^{2}=\tfrac{1}{i_{e}}\cdot \sum _{i=1}^{i_{e}}(y_{i}-avg(y))^{2}=R_{q}^{2}-(avg(y))^{2}$$ (where $$avg(y)=\tfrac{1}{i_{e}}\cdot \sum _{i=1}^{i_{e}}y_{i})$$
- $R_{p}$ is defined as the distance between the highest asperity (i.e., peak or summit) and the mean line ($avg(y)=\tfrac{1}{i_{e}}\cdot \sum _{i=1}^{i_{e}}y_{i}))$;
- $R_{v}$ is defined as the distance between the mean line ($avg(y)=\tfrac{1}{i_{e}}\cdot \sum _{i=1}^{i_{e}}y_{i})$) and the lowest valley;
- $R_{t}=R_{p}+R_{v}$ = distance from the highest asperity (i.e., peak or summit) to the lowest valley;

For Gaussian surfaces $\sigma \approx \sqrt{\tfrac{\pi }{2}}\cdot R_{a}$ (Seewig 2013). The $avg(y)>0$ for the mastication surface is indicated in Fig. 8. More detail and other descriptors can be found in Bhushan (2000). For mites, features are taken to be driven deterministically (although an empirical test of Gaussian isotropy will be made). Stochasticity is thus between sampled individuals. More details can be found in ISO 25178 https://en.wikipedia.org/wiki/ISO_25178.

Thinking of the moveable digit mastication surface [*x*, *y*] profile as a landscape where the *L*2*M* bar-like basis axis of $y_i=avg(y)$ is ‘sea-level’, means that hills and ‘flood-able’ valleys can be defined (i.e., those $x_i$ where $y_{i}> avg(y)$ and $y_{i}\le avg(y)$ respectively). Taking this simple idea of ‘sea-level asperities‘ forward, areas of ‘orogenesis’ (mountain-building upwards on the moveable digit mastication surface) or ‘erosion’ (i.e., similarly wider depressions into it downwards) can be highlighted (i.e., those contiguous values of $x_i$ where $g_{i}>avg(y)$ or $g_{i}<avg(y)$ respectively). This defines locally continuous regions of moveable digit mastication surface teeth, gullets and blades. A region of continuous peaks or ‘hills‘ (i.e., teeth) is denoted a ‘blade‘. A region of continuous contiguous valleys is denoted a ‘gullet‘. This is contingent upon the moveable digit mastication surface profile being either: down or level then up then down; or, level or up then down then up; or, down or level then up remaining up then down (or, conversely up or level then down remaining down then up) i.e., peaks become valleys by crossing zero downwards and valleys become peaks by crossing zero upwards. The number of peak regions and the number of valley regions (counted as $\eta _{p}$ and $\eta _{v}$ respectively ignoring the moveable digit tip and the end of the mastication surface) can be calculated.

The number of ‘zero crossings‘ ($N_{0}$) density Bhushan 2000 defined as the number of times the profile crosses the *avg*(*y*) mean ‘sea-level‘ line per unit length of mastication surface (i.e., number of crossings from mountainous regions to depression ‘gullet‘ regions and vice versa divided by $x_{i_{e}}$) is also calculated. The number of times a signal crosses can be used as a measure of boundary roughness (Kilday et al. 1993). In moving from the individual asperities to regions of them, a suitable summary [*x*, *y*] location could then be chosen of such potentially homologous teeth, gullets or blade features. These summary locations together with those of the moveable digit tip and the condyle (making sure that they all rescale back to real mite values) might then be used as formal landmarks for geometric morphometrics (Bookstein 2018), if and only if there are commonalities of region pattern ($\eta _{p}$, $\eta _{v}$ and $N_{0}$) across species (otherwise these ‘meta-features‘ too would be semi-landmarks).

### Statistical analysis

Any Welch‘s *t*-test used *t.test* in R. Even if the moveable digit was effectively un-ornamented in practice i.e., approximating a bar-like beam in two dimensional shape, it will have characteristics dependent upon the tip velocity ratio. The Technical Appendix gives derivations of the expected values and variances under the null of no ornamentation for a variety of measures about the mastication surface. For instance, mastication surface theoretical smoothness under the null of no ornamentation can be calculated by feeding values into

$$\begin{aligned} \left[ \frac{a^{2}}{3.(U-L)}\cdot \frac{U^{3}-L^{3}}{U^{3}.L^{3}} \right] - \left[ \frac{2a}{U-L}\cdot \frac{U^{2}-L^{2}}{U^{2}.L^{2}}\right] ^2 \end{aligned}$$

where $a=L1U$, $U=L2M$ and $L=L1U$. Note that this is using the values for that mite not using the average over individual mites. The differences between *O*[*VR*] and its estimated null value ${\hat{\varTheta }}$ were calculated for each specimen as $O[VR]-\widehat{E[VR]}$ and then the average and SE of the mean over appropriate sets of specimens were used in a two-sided *z*-test. The differences between the null value $\varTheta$ and its sample estimate $\widehat{E[VR]}$ were calculated for each specimen and then the average and SE of the mean over appropriate sets of specimens were used in a two-sided *z*-test. In theory, as the $variance(mean)=\tfrac{\sigma ^2}{n}$ and the summary mean estimates (*O*[*VR*] and $\widehat{E[VR]}$) depend upon the number of incremental locations digitised for a mastication surface (i.e., $1\ldots i_{e}$), weights of $\frac{1}{i_{e}}$ (normalised to sum to 1) could be used. Welch‘s weighted *t*-test then uses $precision=i_{e}$ in wtd.t.test from the package ‘weights‘ in R. However, in practice this was not found to make any difference to the conclusions.

Relative elongation (*rel*) is defined by the ‘drape length‘ of the mastication surface (*m*) divided by that distance along the bar-like *L*2*M* basis it covers when projected vertically (i.e., $rel=\frac{\text {length\;of\; mastication\;surface}}{x_{i_{e}}}=\frac{m}{e}$). This extension (indicating ‘toothiness‘) could be considered as a consequence of: either a developmental stretching up or down of $y_{i}$ at each $x_{i}$ (Fig. 6); or a general flexing of the beam-like *L*2*M* axis as it is bent up or down in the area around $x_{i}$ (Fig. 7); or, even a tearing shear or creep at that point (for example this shear would be large at $i=4,11$ in Fig. 8) during evolution. Taking these three options (see Explanatory Appendix):-

- stretch, as a point process, is estimated by $tel=\sum ^{i_{e}}_{i=1}|y_{i}|$ (Fig. 6)
- flex, as a local deforming process, is estimated by $f=\sum ^{i_{e}}_{i=1}|g_{i}|$ (Fig. 7)
- creep or shear, as a regional process, is estimated by $h=\sum ^{i_{e}}_{i=1}|c_{i}|$ (Fig. 8)

**Fig. 9:**
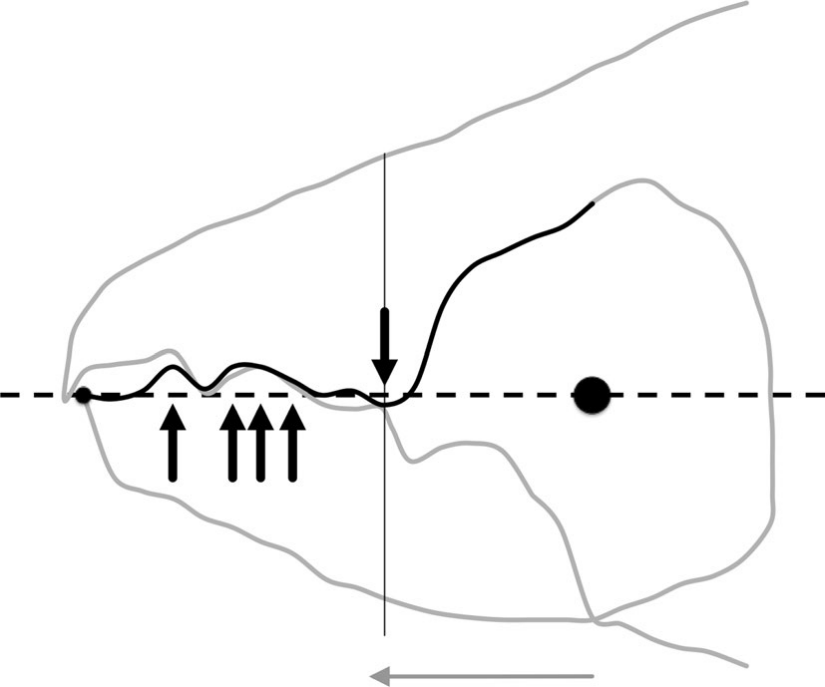
Deflection of *L*2*M* basis producing elongate mastication surface (larger female *Tyrolichus casei* (Oudemans) chelicera for clarity). Bold black arrows show notable locations of stretching or shrinkage of moveable digit ($t_i$ at $x_{i}\; i=4,6,7,11$) compared to a beam-like basis. Vertical line at $i=11$ signifies the end of mastication surface at essentially $x=L2M{-}L1U$. This scale of deviation from the *L*2*M* axis yields, for the mastication surface, a tribological $R_{a}$ value of 0.69 μm (matching an international Roughness Grade Number between *N*5 and *N*6); a tribological $\text {Root\;Mean\; Square}$ ($R_{q}$) value of 0.96 μm$^2$; a tribological $\sigma ^2$ value of 0.62; and, a tribological $R_{t}$ (= distance from the highest asperity, i.e., peak or summit to the lowest valley) value of 2.30 μm. Adductive tendon inserts on coronoid process just posterior of semi-landmark 18

*Per force* one would expect these to be such that $tel \ge f \ge h$ (i.e., repeated derivatives of a smooth continuous continuously differentiable function should usually get smaller in magnitude). Figure 9 illustrates matters for the larger *Tyrolichus casei*. A measure of the least bending energy necessary to bend a (mastication surface) rod to the desired shape can be calculated as $BE=\frac{1}{x_{i_{e}}}.\sum _{i=1}^{i_{e}}(\frac{c_{i}^{2}}{\sqrt{((1+g_{i}^{2})^{5})}})$ (Korn 1983). This averaging can be restricted over different regions of the digit (e.g., horizontal ramus versus ascending ramus with a common ‘knot’ at the end of the mastication surface) as required.

Given $x_{i_{e}}$, the consequence of stretching elongation is *m*. Stretching is the sum of effectively independent point changes separately along the moveable digit i.e., it is most likely to reflect statistically *Normal* morphological processes. So the above check for Gaussian surfaces that $\sigma \approx \sqrt{\tfrac{\pi }{2}}\cdot R_{a}$ is useful. The consequence of bending flexure can be estimated by $m_{f}=\sum _{i=3}^{e}\sqrt{(x_{i}-x_{i-1})^{2}+(g_{i}-g_{i-1})^{2}}$ (think of it as draping a thin piece of string now over the peaks and troughs of the profile of velocities $g_{i}$). This now effectively depends upon coincident changes at adjacent points either side of each location along the moveable digit (i.e., any morphological processes are spread out more and interact with each other over nearby locations). The consequence of creeping shear can be estimated by $m_{c}=\sum _{i=4}^{e}\sqrt{(x_{i}-x_{i-1})^{2}+(c_{i}-c_{i-1})^{2}}$ (think of it as draping a thin piece of string now over the peaks and troughs of the profile of accelerations $c_{i}$). This now effectively depends upon concerted changes at many points along the moveable digit (i.e., morphological processes are spread out even more and interact with each other over a significant proportion of the moveable digit). This measure is unlikely to reflect statistically *Normal* processes.

Dividing these last two measures by $x_{i_{e}}$ gives a relative flexure value ($r_{f}$) and relative creep value ($r_{c}$) respectively with which to interpret better the relative elongation of the surface *r* (‘toothiness‘) between species. The relative creep value is also known as the ‘total absolute curvature’. Fitting a Bezier function (like a cubic spline) requires four data points, so with relative elongation allowed for, considering relative flexure under-smooths the profile in comparison (as it is $\equiv$ to segments of three data points). However, then in turn also considering relative creep or shear would effectively over-smooth the profile in comparison (as it is $\equiv$ to segments of five data points). Given an 18 point profile, using relative ‘snap‘, ‘crackle‘ and ‘pop‘ (https://en.wikipedia.org/wiki/Fourth,_fifth,_and_sixth_derivatives_of_position) loses too much granularity in the digit ornamentation features, to be used.

Gape, reach and *F*1*AV* adductive force on the chelal closing tendon follows Bowman (2021b). Therefore the expected crunch force $F2AV=E[F2]$ theoretically over the likely mastication surface is $F1AV \cdot \varTheta$. These four parameters together along with smoothness and the length of mastication surface are taken to be design measures that could be related to trophic habits. Stretching, flexing and creeping are measures of the possible mechanism to achieve these morphologically. Together with the profile $y_{i}$ co-ordinates, all of the summaries above may be used in ordinations, multiple regressions, MANOVA etc., as needed.

## Results

Data for the characterisation of the moveable digit can be found in: Table 1 of Bowman (2023a) and herein as Tables 2, 3, 4 and 5 for *Carpoglyphus lactis*; Tables 6, 7, 8 and 9 for *Glycyphagus domesticus*; and, Tables 10, 11, 12 and 13 for *Tyrophagus putrescentiae* together with their matching mean values for the laboratory cultures of *C. lactis* (Ca4), *G. domesticus* (G5) and *T. putrescentiae* (T13) used by Bowman (2021c).

**Table 2:** *Carpoglyphus lactis* derived measures from original data in μm

Specimen	*O*[*VR*]	$\varTheta$	$\varPhi ^{2}$	Smoothness (*s*)	Stretch (*tel*)	Flex (*f*)	Creep (*h*)	Relative elongation (*r*)	$m_{f}$	$m_{c}$	$r_{f}$	$r_{c}$	*Potential* ‘Tooth row’ $(L2M{-}L1U)$
224(1)-1	0.662	0.589	0.0286	0.013	4.8	1.7	0.7	1.09	19.8	17.8	1.11	1.11	17.1
224(1)-5	0.541	0.525	0.0332	0.007	5.1	1.5	0.5	1.10	22.2	20.1	1.13	1.12	21.3
224(1)-6	0.512	0.531	0.0328	0.023	8.9	1.6	0.8	1.09	20.3	18.0	1.12	1.11	21.2
224(1)-6a	0.435	0.480	0.0357	0.006	6.1	1.0	0.5	1.07	20.3	18.2	1.12	1.12	22.5
224(1)-6b	0.505	0.500	0.0347	0.011	2.6	1.2	0.5	1.02	21.6	19.5	1.09	1.08	21.8
224(1)-6c	0.544	0.552	0.0314	0.012	5.4	1.3	0.6	1.08	19.8	17.8	1.14	1.14	19.9
224(1)-10	0.661	0.586	0.0288	0.010	7.5	1.3	0.7	1.10	20.8	18.6	1.12	1.10	17.9
224(1)-11	0.439	0.484	0.0355	0.017	6.4	1.3	0.6	1.04	23.6	21.3	1.11	1.11	26.4
224(1)-11a	0.646	0.581	0.0293	0.017	4.4	1.3	0.6	1.06	20.0	18.0	1.11	1.11	17.7
224(1)-11b	0.559	0.530	0.0329	0.041	5.3	1.4	0.6	1.08	21.4	18.6	1.15	1.10	19.7
224(1)-11c	0.557	0.557	0.0310	0.010	8.3	1.2	0.7	1.08	20.7	18.3	1.13	1.12	20.5
224(1)-12	0.510	0.529	0.0329	0.012	11.1	1.7	0.7	1.12	20.3	18.0	1.14	1.12	21.0
224(1)-12a	0.531	0.541	0.0322	0.010	4.4	1.2	0.5	1.02	22.3	20.3	1.09	1.09	23.5
224(1)-13	0.421	0.472	0.0361	0.021	9.7	1.8	0.7	1.05	19.5	17.6	1.14	1.14	22.1
224(1)-13a	0.378	0.459	0.0366	0.007	6.4	1.2	0.7	1.03	17.9	16.1	1.11	1.12	22.7
224(1)-15	0.471	0.508	0.0342	0.011	4.7	0.6	0.3	1.03	20.4	18.3	1.14	1.13	21.9
224(1)-15a	0.408	0.461	0.0365	0.011	3.8	1.2	0.6	1.05	19.9	17.8	1.10	1.11	23.0
224(1)-15b	0.440	0.505	0.0344	0.007	5.6	1.5	0.6	1.05	19.7	17.6	1.13	1.13	23.6
224(1)-16	0.538	0.548	0.0317	0.004	10.0	1.0	0.2	1.03	20.3	18.4	1.12	1.13	20.9
224(1)-18	0.663	0.589	0.0286	0.022	4.7	1.2	0.4	1.05	22.4	20.0	1.13	1.11	19.2
224(1)-19	0.459	0.495	0.0349	0.022	8.4	0.9	0.3	1.07	17.9	15.9	1.14	1.12	19.1
Summary	0.518	0.525	0.0329	0.012	6.4	1.3	0.6	1.06	20.5	18.4	1.12	1.12	21.2
	(0.0866)	(0.0417)	(0.00262)	(0.0084)	(2.27)	(0.29)	(0.16)	(0.029)	(1.39)	(1.29)	(0.017)	(0.015)	(2.23)
Ca4 ($n=20$)	0.604	0.571	0.0295	0.016	6.5	1.4	0.7	1.05	18.0	16.2	1.12	1.12	11.8

Summary is mean, median or geometric mean as appropriate. SD in (....)

**Table 3:** *Carpoglyphus lactis*. Tribological parameters of mastication surface

Specimen	$R_{a}$ (μm)	$R_{q}$ (μm)	$\sigma ^{2}$	$\frac{\sigma }{R_{a}}$	$R_{p}$ (μm)	$R_{v}$ (μm)	$R_{t}$ (μm)	No. of peaks ($\eta _{p}$)	No. of valleys ($\eta _{v}$)	No. of crossings ($N_{0}$)	$\frac{N_{0}}{x_{i_{e}}}$
224(1)-1	0.43	0.54	0.250	1.17	0.95	− 0.77	1.60	2	3	4	0.21
224(1)-5	0.41	0.56	0.306	1.34	1.43	− 0.64	2.07	3	2	4	0.19
224(1)-6	0.37	0.84	0.289	1.47	1.50	− 0.55	2.05	2	2	2	0.10
224(1)-6a	0.33	0.62	0.175	1.27	0.82	− 0.50	1.32	2	2	3	0.15
224(1)-6b	0.22	0.29	0.063	1.16	0.38	− 0.45	0.83	3	3	5	0.23
224(1)-6c	0.40	0.57	0.235	1.21	1.13	− 0.52	1.65	2	2	3	0.16
224(1)-10	0.45	0.71	0.319	1.24	1.10	− 0.59	1.70	2	2	3	0.15
224(1)-11	0.52	0.67	0.344	1.12	0.96	− 1.03	1.98	1	2	2	0.09
224(1)-11a	0.33	0.49	0.161	1.21	0.93	− 0.60	1.53	1	2	2	0.10
224(1)-11b	0.37	0.57	0.219	1.27	1.12	− 0.58	1.59	2	2	3	0.15
224(1)-11c	0.44	0.96	0.357	1.37	1.20	− 0.76	2.33	2	2	3	0.15
224(1)-12	0.59	1.13	0.541	1.24	1.45	− 0.93	2.38	2	2	3	0.15
224(1)-12a	0.26	0.46	0.101	1.23	0.60	− 0.48	1.08	2	3	4	0.18
224(1)-13	0.54	1.00	0.369	1.12	0.83	− 1.02	1.85	1	2	2	0.11
224(1)-13a	0.29	0.67	0.111	1.14	0.58	− 0.37	0.83	2	2	3	0.17
224(1)-15	0.21	0.54	0.074	1.27	0.47	− 0.49	0.96	3	3	5	0.25
224(1)-15a	0.37	0.46	0.184	1.17	0.56	− 0.64	1.20	3	3	5	0.25
224(1)-15b	0.43	0.59	0.256	1.19	0.61	− 1.07	1.68	1	2	2	0.10
224(1)-16	0.47	1.01	0.366	1.30	0.54	− 1.55	1.72	1	1	1	0.05
224(1)-18	0.44	0.53	0.275	1.20	1.03	− 0.66	1.68	1	1	1	0.05
224(1)-19	0.37	1.04	0.294	1.46	0.88	− 1.30	1.45	3	2	4	0.23
Summary	0.39	0.68	0.222	1.25	0.91	− 0.74	1.59	2	2	3	0.15
	(0.099)	(0.229)	(0.1164)	(0.099)	(0.331)	(0.305)	(0.444)	(0.74)	(0.57)	(1.20)	(0.060)
Ca4 ($n=20$)	0.39	0.68	0.202	1.22	0.86	− 0.69	1.41	1	2	2	0.15

For Gaussian surfaces $\frac{\sigma }{R_{a}}\approx \sqrt{\tfrac{\pi }{2}}=1.25$ (Bhushan 2000). Number of peaks and number of valley does not include moveable digit tip. Number of crossings does not include shift from $L2M=0$ axis at moveable digit tip, nor any shift after $x_{i{e}}$. Summary is mean, median or geometric mean as appropriate. SD in (....)

**Table 4:** *Carpoglyphus lactis*. Stochastic characterisation

Specimen	$\widehat{E[VR]}={\hat{\varTheta }}$	Ov[VR]	$\widehat{Var[VR]}=\widehat{\varPhi ^{2}}$	$\frac{1000\cdot R_{t}}{IL}$
224(1)-1	0.662	0.0747	0.0748	7.02
224(1)-5	0.541	0.0470	0.0471	7.83
224(1)-6	0.513	0.0325	0.0328	8.51
224(1)-6a	0.435	0.0238	0.0239	5.94
224(1)-6b	0.505	0.0456	0.0457	3.56
224(1)-6c	0.544	0.0352	0.0353	6.81
224(1)-10	0.662	0.0788	0.0790	7.42
224(1)-11	0.439	0.0237	0.0238	8.01
224(1)-11a	0.646	0.0697	0.0700	6.61
224(1)-11b	0.560	0.0546	0.0551	6.46
224(1)-11c	0.559	0.0383	0.0390	9.60
224(1)-12	0.511	0.0318	0.0320	10.01
224(1)-12a	0.531	0.0354	0.0354	4.24
224(1)-13	0.422	0.0211	0.0211	8.24
224(1)-13a	0.379	0.0135	0.0135	3.62
224(1)-15	0.472	0.0260	0.0262	4.01
224(1)-15a	0.408	0.0207	0.0207	5.04
224(1)-15b	0.440	0.0173	0.0174	7.15
224(1)-16	0.540	0.0345	0.0351	7.04
224(1)-18	0.663	0.0748	0.0751	7.15
224(1)-19	0.462	0.0265	0.0281	6.66
Summary	0.519	0.0348	0.0351	6.71
	(0.0865)	(0.02018)	(0.02019)	(1.806)
Ca4 (n = 20)	0.605	0.0489	0.0494	7.10

Summary is mean, median or geometric mean as appropriate. SD in (....)

**Table 5:** *Carpoglyphus lactis* sorted by type

Specimen	Type	CLE	*γ* (°)	Pocket *EVR*	Pocket vol.	F1	Pocket *E*[*F*2]	Slicing blade Retr.	Cutting edge Retr.	Slicing blade Protr.	Cutting edge Protr.
224(1)-1	A	13.0	139	0.509	103.5	992.8	505.3	6.4	–	–	1.3
224(1)-6b	A	10.6	165	0.350	39.4	1071.6	375.1	11.0	–	–	0.5
224(1)-11b	A	15.4	145	0.450	130.4	1096.5	493.4	5.0	–	–	1.6
224(1)-12	A	14.3	141	0.423	155.4	934.3	395.2	5.6	–	–	2.0
224(1)-16	A	14.7	149	0.447	118.8	902.3	403.3	1.9	–	–	0.8
224(1)-18	A	14.2	163	0.503	86.5	1240.8	624.1	2.3	–	–	1.1
224(1)-11	B	06.2	96	0.619	69.1	888.2	549.8	–	1.4	17.1	–
224(1)-12a	B	16.3	85	0.586	272.0	1198.0	702.0	–	0.8	6.2	–
224(1)-13	B	05.4	81	0.585	66.6	962.4	563.0	–	1.2	13.9	–
224(1)-19	B	14.1	104	0.400	87.5	854.7	341.9	–	1.2	0.9	–
224(1)-5	Indet.										
224(1)-6	Indet.										
224(1)-6a	Indet.										
224(1)-6c	Indet.										
224(1)-10	Indet.										
224(1)-11a	Indet.										
224(1)-11c	Indet.										
224(1)-13a	Indet.										
224(1)-15	Indet.										
224(1)-15a	Indet.										
224(1)-15b	Indet.										
	A	13.7	150	0.447	105.7	1039.7	466.1	5.4	–	–	1.2
	B	10.5	92	0.548	123.8	975.8	539.2	–	1.2	9.5	–
	Overall					1014.2 (132.74)					

Specimen	Shank of hook Retr.	Total Retr.	Tear prop. Retr.	Shank of hook Protr.	Total Protr.	Tear prop. Protr.	$\tfrac{CLE}{CLI}$	$R_p[VR]$	$R_p[F2]$	$x_{R_p}$	No. teeth^a^	Pitch^a^
224(1)-1	14.4	27.4	0.47	–	–	–	0.14	0.713	707.5	12.9	4	0.21
224(1)-6b	19.8	30.4	0.35	–	–	–	0.11	0.436	467.3	10.6	3	0.14
224(1)-11b	13.3	28.7	0.54	–	–	–	0.16	0.678	743.4	15.4	3	0.15
224(1)-12	16.2	30.5	0.47	–	–	–	0.15	0.589	550.7	14.3	3	0.15
224(1)-16	16.7	31.3	0.47	–	–	–	0.15	0.622	561.0	14.6	4	0.20
224(1)-18	16.6	30.8	0.46	–	–	–	0.14	0.696	864.2	14.2	3	0.14
224(1)-11	12.9	19.0	0.32	17.1	23.2	0.26	0.06	0.513	455.7	17.1	4	0.17
224(1)-12a	12.3	28.6	0.57	6.2	22.5	0.72	0.16	0.395	473.5	6.2	4	0.18
224(1)-13	10.8	15.9	0.32	13.9	19.0	0.27	0.06	0.485	466.6	13.9	4	0.21
224(1)-19	12.4	26.5	0.53	0.0	14.1	1.00	0.16	0.280	239.2	0.0	3	0.17
224(1)-5										19.9	3	0.14
224(1)-6										14.4	3	0.15
224(1)-6a										14.2	3	0.15
224(1)-6c										14.0	3	0.16
224(1)-10										Indet.	4	0.20
224(1)-11a										16.4	4	0.20
224(1)-11c										Indet.	4	0.20
224(1)-13a										14.3	3	0.17
224(1)-15										Indet.	3	0.15
224(1)-15a										Indet.	3	0.15
224(1)-15b										Indet.	4	0.21
	16.2	29.8	0.46	–	–	–	0.14	0.622	649.0	13.7		
	12.1	22.5	0.44	9.3	19.7	0.56	0.11	0.418	408.7	9.3		
								0.541 (0.1436)	552.9 (178.30)	13.3 (5.15)	3.4 (0.53)	0.17 (1.791)

^a^Subjective measures. Indet. = indeterminate. – = data impossible. Summary is mean, median or geometric mean as appropriate. SD in (....)

The measures: idiosomal index *IL*, reach (*CLI*), gape (*L*2*M*), $x_{i_{e}}$, *m*, distance from the condyle (in *L*1*U* units), $\delta ^{\ast}$, G, $VR_{tip}$, bite/grab max volume $M_{vG}$, thickness at condyle, *L*1*U*, food fragment truncated volume $TM_{vG}$, estimated idiosomal volume, no. of bite/grab equivalents, excavation time equivalents, $i_{e}$, and *CHI* are discussed in Bowman (2023a) (and not herein). Note Bowman (2023a) labels the herein defined $\delta ^{\ast}$ as $\delta$ therein.

Mean values for the laboratory cultures for the measures: *IL*, reach (*CLI*), gape (*L*2*M*), $x_{i_{e}}$, *m*, distance from the condyle (in *L*1*U* units), $\delta ^{\ast}$, G, $VR_{tip}$, bite/grab max volume $M_{vG}$, thickness at condyle, *L*1*U*, food fragment truncated volume $TM_{vG}$, estimated idiosomal volume, no. of bite/grab equivalents, excavation time equivalents, $i_{e}$, and *CHI* are shown in Table 14 for comparison as necessary with Bowman (2023a).

The set of museum specimens of *T. putrescentiae* was markedly different throughout, suggesting the possibility of local differentiation in this cosmopolitan pest species to different locales and habitats. For sure *T. putrescentiae* can be found in other sorts of beehives such as Africanized honey bees in Brazil (Teixeira et al. 2014). There, mites were found on the larvae, pupae, bee bread (fermented pollen mixture stored in the honeybee combs) and in the empty cells. Besides the mites, hyphae of an unidentified fungus were also observed abundantly on the combs, and along with the young larvae and pupae of *Apis mellifera*. For more information on the trophic characteristics of *T. putrescentiae* see Erban et al. (2015).

Despite flattening in the slide preparations, cheliceral digits may not have been in an exactly lateral view when measured. This may have introduced some extra variation in the results, especially those determining very small features and therefore this may impact some micro-roughness measure estimates. Whenever possible summary measures over specimens for a sample are used to make conclusions between species.

No clear evidence of a ‘gabelzhan‘ (i.e., an off-set tooth at the cheliceral fixed digit hooked tip as in some mesostigmatids) was seen. No adaptations to the cheliceral chelae like those found on the anterior mandible in the vertebrate hippopotamus, wild boars, *Babirusa* or Chinese water deer were observed. No evidence of any ‘snap-jaw‘ mechanism like in ant mandibles (Larabee et al. 2018) by which analogously the moveable digit and fixed digit tips would first grasp material, occlude (storing crushing energy) and then on increasing adductive force rapidly slide past each other abruptly reducing any space further between chelal ornamentations was seen. No ‘Rollplatte‘ as in uropodoids (Bowman 2021b) which may act as such a click mechanism or may indicate whole chelal head flexure (to tear up grabbed material like the end section of an elephants trunk) was observed.

Like Akimov and Gaichenko (1976) illustrate for *Acarus siro*, *Carpoglyphus lactis* and *Kuzinia laevis*, the angle at which the input moment lever arm length (*L*1*U*) subtends to the output moment lever are length (*L*2*M*) moveable digit axis was visually invariably around 90$^{\circ }$ for *C. lactis* and *Tyrophagus putrescentiae* herein but was markedly less than a right angle in *Glycyphagus domesticus* specimens. This contrasts with the opposite deviation illustrated for *Chortoglyphus arcuatus* by Akimov and Gaichenko (1976). These deviations do not change the leverage principles used in this study, but due to the effective change in the angle of the adductive tendon, do reduce the effective occlusive input force (*F*1) and thus the true crunch force (*F*2) against food in such species.

Comparison of the body size, reach and gape values in Tables 2, 6 and 10 with those from laboratory cultures kept at 20$^{\circ }$ C found in Bowman (2021c) shows that the experimental samples are larger in all aspects on average for all three species, suggesting either cooler environmental conditions (e.g., Bergmann‘s rule) during their growth or a more appropriate long-term diet than in the laboratory. There was a cold wintry start to April 1983 with much of the UK under a North Easterly wind with showers of snow and hail in places. The average daily temperature in Bristol was 8.0$^{\circ }$ C for the month when the bee-hive sample was taken. Although temperatures in the central cluster of honeybees is kept at 33–36 °C for their survival, comparison of the idiosomal index values for the experimental sample of *T. putrescentiae* to either laboratory-held T13 or T58 kept at various temperatures (Bowman 2021a) suggests that field conditions experienced for mite development in Redland on average was around 10 °C. Clearly these mites were inhabiting the peripheral regions of the hive. Note that the museum acarid specimens were particularly small indicating either poor diet or high temperature conditions during their development.

**Table 6:** *Glycyphagus domesticus* derived measures from original data in μm

Specimen	*O*[*VR*]	$\varTheta$	$\varPhi ^{2}$	Smoothness (*s*)	Stretch (*tel*)	Flex (*f*)	Creep (*h*)	Relative elongation (*rel*)	$m_{f}$	$m_{c}$	$r_{f}$	$r_{c}$	*Potential* ‘Tooth row’ $(L2M{-}L1U)$
224(2)-1	0.581	0.634	0.0249	0.037	8.4	2.6	1.2	1.32	16.8	13.9	1.19	1.15	19.2
224(2)-2	0.667	0.667	0.0219	0.056	8.8	3.1	1.6	1.33	16.2	14.0	1.22	1.17	15.3
224(2)-3	0.620	0.658	0.0227	0.030	5.7	2.3	1.1	1.17	15.8	13.2	1.21	1.18	17.4
224(2)-4	0.587	0.620	0.0261	0.048	10.4	3.3	1.5	1.27	17.2	14.5	1.22	1.17	17.9
224(2)-5	0.513	0.575	0.0297	0.095	13.1	3.9	2.0	1.36	18.4	15.0	1.26	1.18	20.1
224(2)-6	0.793	0.733	0.0159	0.041	6.4	3.1	1.4	1.21	14.7	12.8	1.17	1.15	12.0
224(2)-7	0.733	0.703	0.0187	0.100	8.1	2.1	1.4	1.32	15.8	13.1	1.26	1.18	13.0
224(2)-8	0.705	0.688	0.0200	0.038	5.3	2.1	1.2	1.23	15.7	13.2	1.23	1.19	14.0
224(2)-9	0.578	0.649	0.0235	0.021	6.1	1.9	0.7	1.21	14.4	12.0	1.20	1.20	18.6
224(2)-9a	0.706	0.707	0.0183	0.017	6.1	1.3	0.6	1.15	15.6	13.1	1.20	1.19	15.3
224(1)-10	0.570	0.607	0.0272	0.089	9.3	3.2	1.7	1.30	17.6	14.0	1.26	1.16	17.5
224(1)-20	0.631	0.666	0.0220	0.072	7.2	2.7	1.5	1.26	14.8	12.3	1.25	1.19	15.6
Summary	0.640	0.659	0.0222	0.046	7.9	2.6	1.3	1.26	16.1	13.4	1.22	1.18	16.3
	(0.0815)	(0.0454)	(0.00400)	(0.0288)	(2.28)	(0.73)	(0.40)	(0.067)	(1.22)	(0.88)	(0.030)	(0.016)	(2.53)
G5 ($n=20$)	0.623	0.657	0.0226	0.048	7.6	2.4	1.3	1.24	15.2	12.6	1.24	1.19	15.5

Summary is mean, median or geometric mean as appropriate. SD in (....)

Comparing across species, as expected *C. lactis* at 0.129 has a wider gape for its body size ($\frac{Gape}{IL}$) on average than the comparable body size acarid (where this ratio is 0.114)). *G. domesticus* is confirmed as a large reach, large gape taxon for its body size (average $\frac{Gape}{IL}=0.143)$. A good summary of *C. lactis* biology can be found by searching the term ‘Carpoglyphus’ at https://idtools.org/bee_mite/. Similarly, a good summary of *G. domesticus* biology with respect to bees can be found by searching the term ‘Glycyphagus’ at https://idtools.org/bee_mite/.

Using the definition of ‘gnathosomatisation’ in Bowman (2021b): the wild-collected *C. lactis*, culture Ca4, and the wild-collected *T. putrescentiae* would all be classed as micro-cephalic; culture T13 would be classed as meso-cephalic; and, both G5 and *G. domesticus* would be classed as mega-cephalic.

As expected the theoretical average velocity ratio values ($\varTheta$) are larger than the moveable digit $VR_{tip}$ values. *G. domesticus* and *T. putrescentiae* have very similar $\varTheta$ values on average (spanning 0.64 to 0.67), suggesting a similar bandwidth in masticatory flexibility. The beehive sample of *C. lactis* shows the lowest on average $\varTheta$, observed *O*[*VR*] and $VR_{tip}$ values. However, even that species could in theory produce a major multiplier of adductive muscle force on foodstuff held at the posterior parts of its mastication surface (where three small teeth are found).

### Geometric morphometric considerations

**Fig. 10:**
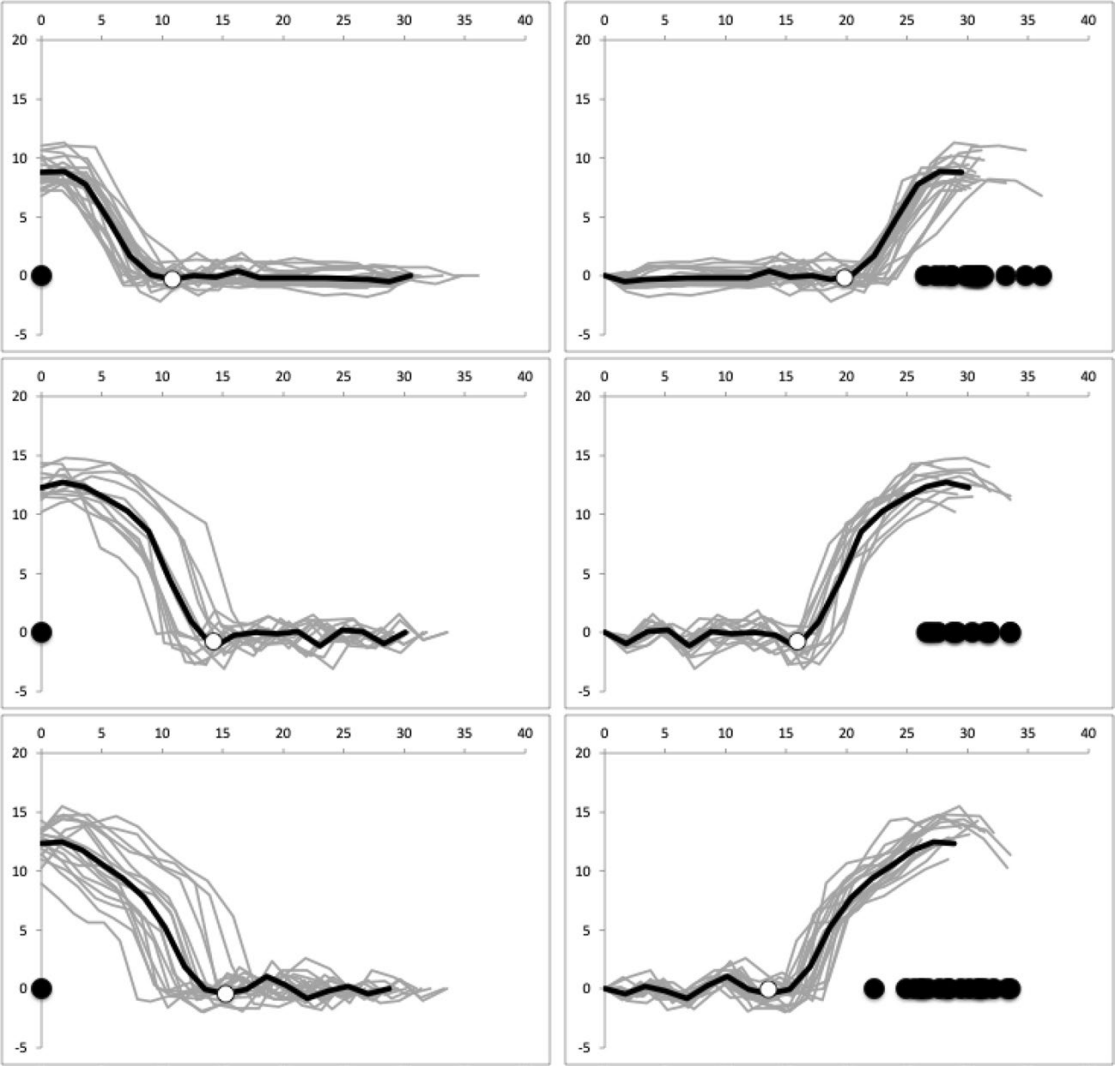
Individual (plus overall mean) raw profiles of chelal moveable digits (in μm) aligned in two different ways. $y=0$ is the *L*2*M* axis. Black circle = condyle. Grey lines each wild-collected specimen. Black line mean values including open circle at the end of the mastication surface. Top row *Carpoglyphus lactis*. Middle row *Glycyphagus domesticus*. Bottom row *Tyrophagus putrescentiae*. Left hand column is with the condyle as the reference origin. Right hand column with the moveable digit tip as the reference origin

Individual moveable digit profiles are displayed (together with the mean profile for each species) in Fig. 10. Of note is the much larger posterior (proximal) parts of the moveable digit in the glycyphagid and the greater inter-individual variation shown by *T. putrescentiae*. A central ‘tooth’ in *T. putrescentiae*, suitable for scraping or cracking food material is clear irrespective of the reference chosen. Such can be found in other arthropods e.g., the decapod genus *Cherax* (Lukhaup and Eprilurahman 2022).

There are many ways to empirically investigate size and shape of structures including measuring: orientation (angle), area, perimeter, directions like major axis and minor axis, major axis angle (versus a reference direction), compactness, elongation, eccentricity, circularity (roundness), convexity and convex perimeter or area, height to width aspect ratios, feret diameters, curl, fibre width, solidity, rectangularity and bounding box measures, topological measures like Euler number and chain codes, Richardson and fractal dimension, various variances against reference shapes and spatial moments (like centroids etc.), radial distances, entropies, etc., etc. Some have been used in mesostigmatid studies (Adar et al. 2012; Liu et al. 2017). Each is informative in answering a different question. Fourier (Staib and Duncan 1992), harmonic, elliptical and spectral decompositions can be deployed. If particular objects of defined shape are to be detected, the Hough transform can be used.

An increasingly popular way of visualising *morphological* change is to use geometric morphometrics (Bookstein 2018). By nature of the physics, the raw profiles in this review are partly pre-aligned. A Procrustean operation would simply budge individual profiles left-right and shrink and swell them to minimise overall variation (with respect to the overall Procrustean mean). The latter means that the comparative physical scale is partly lost.

Moreover, these raw profiles (in Fig. 10) show that depending upon whether the condyle is taken as the reference origin (on the left), or the moveable digit tip (as the reference origin on the right), profoundly changes one’s interpretation of where evolution may have driven character variation. In one case (see the left hand column) digit tip elongation is the main modality. In the other (see the right hand column) a shape change in the ascending ramus/coronoid process is the modality. Geometric morphometrics would blindly try to play off these around an overall Procrustean mean despite their different (physics-based) drivers (see Results and Discussion below). One would also need to rationally decide whether (and how) covariances should be standardised.

Further, little would be gained at this density of semi-landmarks (compared to the real dentition shapes and dentition roughness) if such were included into a geometric morphometric analysis even if the semi-landmarks were allowed to slide along a tangent manifold (Gunz and Mitteroecker 2013). To investigate this properly in follow-up work very large numbers of semi-landmarks across the folded surface of the moveable digit mastication surface would be needed. Even that would still require a claim of homology of digit curves over the three species, evidence for which is not clear.

Geometric landmarks, which form the basis for all morphometric measurements and latent shape variables, have no necessary correspondence to biological homology (MacLeod 2011). The mastication surface itself should be a homology across the mites but discontinuities (‘crinkles’) along it must be a feature of real life e.g., whole teeth missing, no gullets on a slicing blade etc., in any highly disparate structures (Bardua et al. 2019). Although Tables 5, 9 and 13 do indicate that on average the three species appear to have the same number of teeth (i.e. $\approx$ 3) on their moveable digits (and so these might be considered as ‘pukka’ landmarks), how to place a semi-landmark for the fourth asperity that sometimes is subjectively scored is debatable. Should it be proximal or distal of the three teeth, or between asperity 1 and 3, or between asperity 2 and 3, etc.,? Novel features are not straightforward to deal with on jaws (Gómez-Robles et al. 2011). A degree of *post hoc* trial and error and choice by the analyst including semi-landmark density would be needed to reconstruct imaginary profiles parsimoniously (see Shui et al. 2023).

The aim of this study was to elucidate the functional *physics-based* consequences of structures (themselves driven by morphological changes) at their actual scale. It was not necessarily to visualise that intrinsic morphological variation in its own right. So a geometric morphometric analysis with all its assumptions around normalisation, the linearisation of the Riemannian space of tensors (Dryden et al. 2009), applicability of rigid surface thin plate splines to developmental processes in vivo etc.) is eschewed herein as the first place to start digit investigations. In mites, *actual* size matters; Bowman (2021b), Seeman and Nahrung (2018) and Sidorchuk (2018).

### End of moveable digit mastication surface and the ‘drape’ or ‘chain’ distance

The observed location of the maximum jerk in the moveable digit surface ($x_{i_{e}}$) is shown in Tables 2, 6, 10 for the different specimens, and is discussed in Bowman (2023a). This, as a distance from the condyle in *L*1*U* units, is shown in Tables 3, 7, and 11. As invariably the next semi-landmark posteriorly from $x_{i_{e}}$ was that one showing maximum moveable digit profile curvature, it suggests that the moveable digit is definitely not working as a mastication surface by a distance of $\tfrac{19}{20}$ths of the distance from the condyle. That is, 1.07 *L*1*U* units for the experimental sample of *C. lactis*, 1.06 *L*1*U* units for the experimental sample of *G. domesticus*, and 1.02 *L*1*U* units for the experimental sample of *T. putrescentiae*. The moveable digit surface thus rises forming the ‘ascending ramus‘ on average at this point, just before the theoretical cut-off point for a functioning grasping or chewing ‘machine‘ (at 1.0, see Smith 1978).

**Table 7:** *Glycyphagus domesticus* tribological parameters of mastication surface

Specimen	$R_{a}$ (μm)	$R_{q}$ (μm)	$\sigma ^{2}$	$\frac{\sigma }{R_{a}}$	$R_{p}$ (μm)	$R_{v}$ (μm)	$R_{t}$ (μm)	No. of peaks ($\eta _{p}$)	No. of valleys ($\eta _{v}$)	No. of crossings ($N_{0}$)	$\frac{N_{0}}{x_{i_{e}}}$
224(2)-1	0.90	1.07	1.068	1.15	1.44	− 1.70	3.14	3	3	5	0.31
224(2)-2	0.81	1.24	0.959	1.21	1.33	− 2.02	3.34	3	3	5	0.33
224(2)-3	0.64	0.77	0.592	1.21	1.31	− 1.08	2.14	2	2	3	0.20
224(2)-4	0.80	1.27	0.793	1.11	1.61	− 1.23	2.84	3	3	5	0.31
224(2)-5	0.96	1.68	1.311	1.19	1.61	− 1.85	3.46	2	2	3	0.18
224(2)-6	0.62	0.81	0.591	1.23	1.64	− 1.11	2.53	2	2	3	0.21
224(2)-7	0.71	0.96	0.656	1.14	1.25	− 1.36	2.61	3	4	6	0.43
224(2)-8	0.51	0.62	0.383	1.21	1.04	− 0.89	1.94	3	3	5	0.35
224(2)-9	0.76	0.93	0.645	1.06	1.12	− 1.12	2.11	2	2	3	0.21
224(2)-9a	0.60	0.84	0.544	1.22	1.09	− 1.39	1.74	2	2	3	0.20
224(1)-10	0.75	1.27	0.821	1.21	1.11	− 1.84	2.95	3	3	5	0.32
224(1)-20	0.78	0.93	0.812	1.16	1.36	− 1.50	2.86	2	3	4	0.29
Summary	0.74	1.03	0.727	1.18	1.33	− 1.42	2.64	3*	3	5**	0.28
	(0.129)	(0.291)	(0.2549)	(0.052)	(0.215)	(0.360)	(0.559)	(0.52)	(0.65)	(1.11)	(0.077)
G5 ($n=20$)	0.69	1.02	0.656	1.19	1.30	− 1.31	2.56	2	2	3	0.26

For Gaussian surfaces $\frac{\sigma }{R_{a}}\approx \sqrt{\tfrac{\pi }{2}}=1.25$ (Bhushan 2000). Number of peaks and number of valley does not include moveable digit tip. Number of crossings does not include shift from $L2M=0$ axis at moveable digit tip, nor any shift after $x_{i{e}}$. Summary is mean, median or geometric mean as appropriate. *Mode (median is 2.5). **Mode (median is 4.5). SD in (....)

$x_{i_{e}}$ could be considered as the length of the *potential* ‘tooth row’ i.e., the region where teeth, pockets, blades etc., could be formed from fundamental asperities and gullets during evolution. For fixed *L*2*M* (i.e., a certain approximate body size, as lengths are generally reasonably correlated in free-living acarines Bowman 2023b), increasing the input moment arm *L*1*U* so that the digit tip velocity ratio (*VR*) increases means that there is less space for *potential* teeth since $x_{i_{e}} \approx (L2M{-}L1U)$. So ‘gnathosomatisation’ (sensu Bowman 2021b) by increasing gnathosomal width (and therefore increasing the input adductive force *F*1 by virtue of the sub-cylindrical nature of cheliceral segments) would infer an increased *L*1*U* and therefore for that mite size (i.e., that *L*2*M*) result in fewer teeth. This would apply to other animals, so many toothed mammalian insectivores (like shrews) should have elongate low velocity ratio jaws on a narrow head, and oribatids with high velocity ratios to crunch intractable food which tend to have a highly sclerotised moveable digit may have just one or two specialised teeth. A follow-up study of oribatids could confirm this.

Turning to the ‘drape distance‘ (*m*), the actual length of the chelal food chewing surface. Note that the moveable digit ‘drape distance‘ *m* will be greater than the average mastication surface length along the *L*2*M* axis ($x_{i_{e}}$ illustrated in Bowman (2023a) as the former allows for surface asperities and gullets. However, the length of the mastication surface for the experimental samples from the wild, even though they rank as expected between species, neither closely agree with *L*2*M* gape measures, nor are consilient with the distance of the end of the mastication surface from the condyle in *L*1*U* units (Tables 3, 7 and 11).

**Fig. 11:**
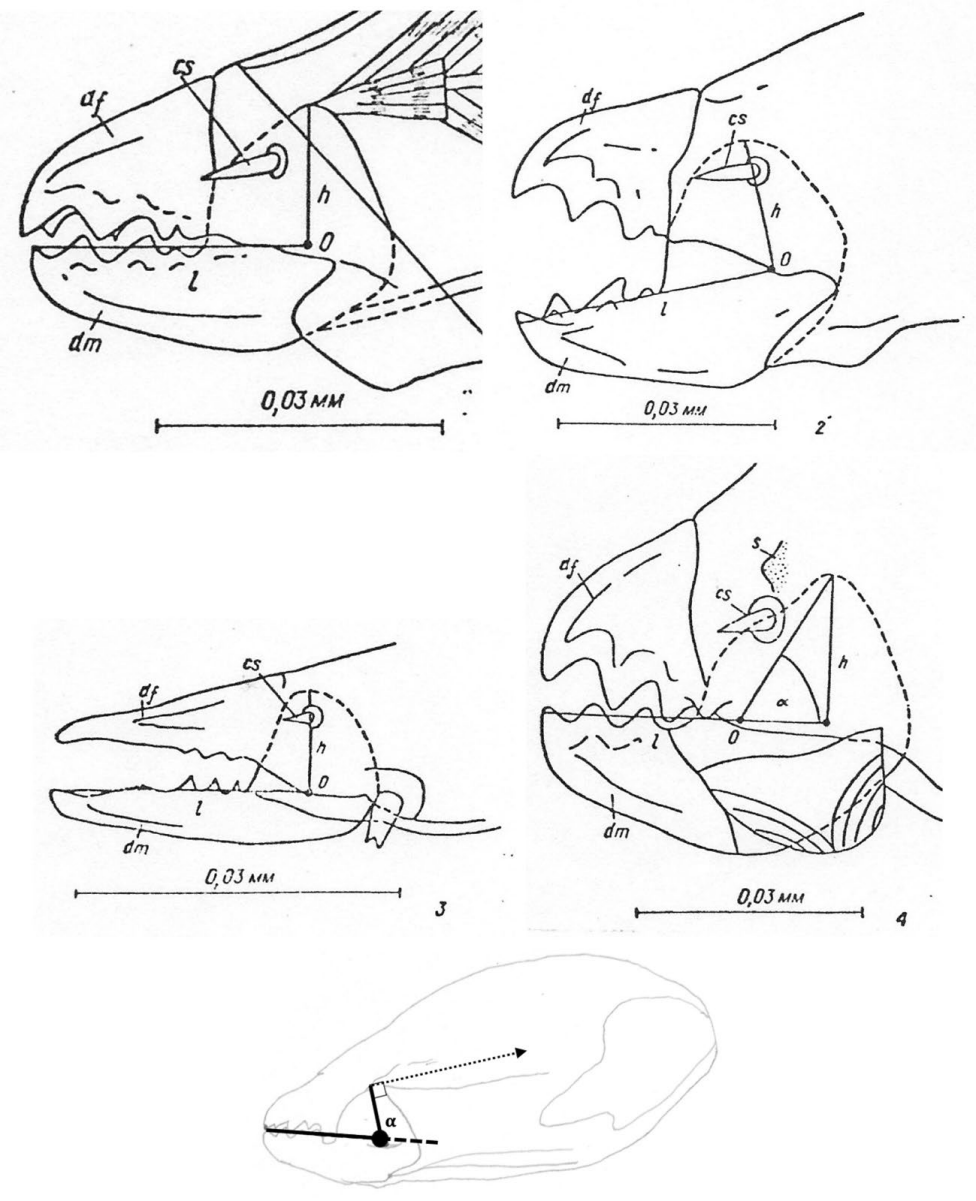
Typical astigmatid chelae showing two to three moveable digit teeth above the *L*2*M* axis (marked by solid line from O for condyle to moveable digit tip) or gullets below it. Top: *Acarus siro* on left, *Kuzinia laevis* on right. Middle: *Carpoglyphus lactis* on left, *Chortoglyphus arcuatus* on right. ©Akimov and Gaichenko (1976) reproduced with permission. df = fixed digit. dm = moveable digit. cs = cheliceral seta. s = cuticular spine. O = condylar joint. h = *L*1*U* (except for *C.arcuatus*). Note that the teeth in *C. lactis* are proximal to the condyle (i.e., distal from moveable digit tip). Lower: *Glycyphagus domesticus* specimen 224(2)-3 showing $\alpha > 90^{\circ }$ and subtended angle to adductive tendon at a right angle (dashed arrow $=F1$ force). Black circle = condyle

How so? What appears to be happening is a change in moveable digit geometry in *G. domesticus* compared to the other two species. Rather than the adductive tendon insertion point being directly above the condyle (and thus making the angle of *L*2*M* to *L*1*U* axes being at 90$^{\circ }$, the insertion point is more forward (as one would expect of the plesiomorphic state) such that this angle is reduced (Fig. 11). This is the opposite trend to that previously illustrated for *Chortoglyphus arcuatus* by Akimov and Gaichenko (1976), whose advantage is not clear. In both cases this reduces the likely scale of *F*1 (and thus *F*2) at digit occlusion.

Discussions and further references on the evolution of acarine appendages from putative plesiomorphic states can be found in the classic work of Francois Grandjean and Leendert van der Hammen (e.g., Grandjean 1947; van der Hammen 1970a, b). Some workers take chelae to arise from a tibio-tarsal complex of the ancient ‘first pair of appendages’, which are sometimes seen as homologous with the primary antenna of the Mandibulata. More investigations are needed by modern workers of chelicerate embryology and molecular phylogeny. Note that in terms of determining the magnitude of the adductive force *F*1 (transmitted to the moveable digit tip via the condylar joint) it is whether the subtended angle of tendon insertion with the ‘ascending ramus’ of the moveable digit is at $90^{\circ }$ or not. Unfortunately this was not illustrated for *C.arcuatus* by Akimov and Gaichenko (1976) (see Fig. 11) so the issue remains unresolved. Follow-up work is needed.

The relative ‘drape distance’ has been used as a measure of surface rugosity in coral reefs (Fuad 2010, where therein the rugosity index measure $C\equiv \frac{(m-x_{i_{e}})}{m}$). Mean values for *C* in the astigmatid moveable digits were: 0.0575 (*C. lactis*), 0.212 (*G. domesticus*), and 0.170 (*T. putrescentiae*). Comparatively these would be scored therefore as ‘low rugosity’ ($<0.170$), ‘moderate rugosity’ (0.171–0.275), and ‘moderate rugosity’, respectively. The latter two classifications would encompass 80% of benthic surfaces whose rugosity was assayed (using $r_{chain} \equiv \frac{m}{x_{i_{e}}}$) by Friedman et al. (2012), indicating how biologically typical mite digits are even at their minute scale.

### What is the likely origin of digit surface morphological differentiation—stretch, flex or creep?

*C. lactis* in having the lowest relative elongation (*rel*) of the mastication surface (Tables 2, 6, and 10) has the most bar-like moveable digit. *G. domesticus* has comparatively the most ‘toothy‘ moveable digit—effectively having a mastication surface 26% bigger than the corresponding coverage interval on the *L*2*M* axis.

The magnitudes of stretch (*tel*), flex (*f*) and creep (*h*) values cascade downwards correctly within each taxon (Tables 2, 6, and 10). Among the experimental samples from the wild, the moveable digit of *G. domesticus* has a noticeably higher stretch value (*tel*) on average suggesting that, of the three co-existing species, it may have the strongest localised point-process drivers in its differentiation (suggesting differentiation into many features like a saw—see below). The digit of *T. putrescentiae* has the highest flex (*f*) and creep (*h*) values suggesting more concerted local patterns of differentiation along the digit. Deriving the comparative $r_{f}$ and $r_{c}$ values from $m_{f}$ and $m_{c}$ confirms the importance of flexure in the mastication surface of *T. putrescentiae* and creep in both the glycyphagid and acarid. Consiliently the carpoglyphid has the lowest relative flex and creep values.

### What can tribology say about moveable digit surface roughness?

Tribology has been only used once in acarines in the context of surface adhesion (Mizutani et al. 2006). The moveable digit tribological estimates are found in Tables 3, 7, and 11. It is acknowledged that standard tribological measures are not scale-free (Sahoo 2011). However, for this review their use is for simply a comparison across the species (i.e., over the same basis) and no claim for absolute characteristics are made. Comparing the experimental sample individuals shows that digit surface roughness varies across these UK beehive species.

ANSI and ISO standards bodies recommend $R_{a}$ and ${Root\;Mean\;Square}$ (or equivalently $\sigma$) as the usual measure of surface waviness. Waviness includes all irregularities whose spacing is greater than the roughness sampling length and less than the waviness sampling length (Bhushan 2000). The waviness sampling length herein is $\approx \frac{L2M}{18}$ (i.e., determined by the semi-landmark density). The average $R_{a}$ values for all samples of the three species are in the range of international Roughness Grade Numbers N5–N6 (Bhushan 2000). None of the moveable digit mastication surfaces could be described as smooth since in comparison the mean surface roughness ($R_{a}$) given by Mizutani et al. (2006) for plates of: glass at 1.08 nm, mica at 0.36 nm, silicon at 0.24 nm, and even gold at 14.03 nm (i.e., two orders of magnitude less rough than found herein). The ${Root\; Mean\; Square}$ values ($R_{q}$) show the same picture. Indeed, mite moveable digits are much smoother than say the surface of dolphin skin (Wainwright et al. 2019).

When two surfaces are in contact there is a force acting on each that acts in a direction to stop them moving past one another. This is due to friction. There are two possibilities: movement, or no movement. Friction acts on objects at the surfaces so as to prevent or reduce movement between the surfaces. When friction prevents sliding there is grip, when sliding is reduced there is slip. Rough surfaces have more friction than smooth surfaces (consider holding a Bronze Age Corded Ware pot versus a shiny modern glass), and liquids can be used as lubricants to reduce the effect of friction (i.e., increase slip, like oil in car engines). The higher that such roughness of the mastication surface is, the greater the friction as food material moves across it as the chelicera moves into or out of foodstuffs. So the force needed to move the whole chelicera through food of the same resistance should be $G.domesticus> T.putrescentiae > C.lactis$ for these mites of broadly similar body size. Follow-up work could check if this is reflected in the volume of their cheliceral retractor muscles. For sure *G. domesticus* has longer taller chelicerae (Bowman 2021c). The chelicerae of the acarid and carpoglyphid are of a similar reach but that of *C. lactis* is noticeably less tall.

$R_{p}$ and $R_{v}$ are extreme value measurements. From Tables 3 and 7 and 11, one can see amongst the UK field sample that although the $R_{v}$ (and $R_{t}$) measurements on average rank in the order expected from Bowman (2021c)’s conclusion, *T. putrescentiae* has the largest $R_{p}$ value on average. Accordingly the latter species does not, on the face-of-it, look like a simply swollen/shrunken design version between *C. lactis* and *G. domesticus*–*T. putrescentiae* appears to have at least one excessively sized tooth than that expected if it was an intermediate between the other two species. There is, of course, the possibility of character displacement by competition during cohabitation or utilisation of a different trophic resource as the explanation for the acarid’s asymmetric high $R_{p}$ and low $R_{v}$ result. Experimental follow-up work is needed. However, the height of the highest asperities above the mean line is an important parameter because damage to food material may be done by a few high asperities present on one of the two chelal digit surfaces. This fits with *G. domesticus* being considered adapted as a ‘shredder‘. Perhaps the beehive acarids are solving the task of extracting nutrition by attacking tough debris in a different way than the glycyphagids?

Examining the velocity ratio values (and thus the crunch force *F*2) at maximum asperity $R_{p}$ (i.e., $R_{p}[VR]$ and $R_{p}[F2]$ in Tables 5, 9, and 13) shows *T. putrescentiae* compared to *G. domesticus* does indeed have a higher velocity ratio on average at this higher than on average asperity. Accordingly crunch forces (*F*2) at this location are much more similar across the two species than their input adductive forces would suggest. *G. domesticus* is investing in primary cheliceration, whilst *T. putrescentiae* has a designed surface of particular relative dentition. This would match the former being a ‘shredder’ and the latter a selective rasping ‘grazer’.

**Fig. 12:**
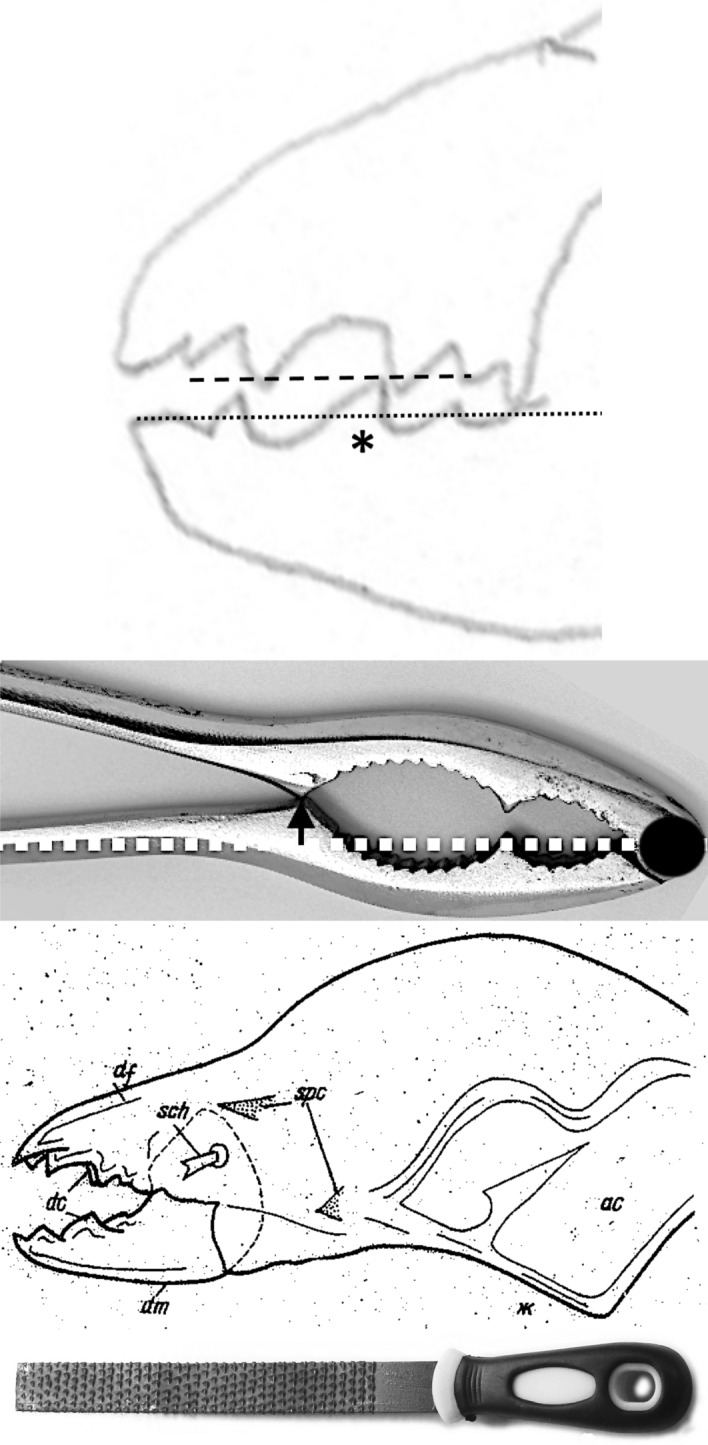
*Tyrophagus putrescentiae*. Upper. Different height moveable digit teeth (specimen 224-1-10a). Food material assumed to rest between digits and around the embedded chela. Dashed line highlights teeth of very diverse sizes (with respect to *L*2*M* axis as dashed line) suitable for scraping different material. Dotted line = tip of moveable digit to articulating condyle. *Major ‘cracking’ tooth approximately half way along mastication surface (inducing ’breasting’ see Fig. 31). Note modest digit depth to resist only modest dorsoventral and torsional forces. Pitch (number of teeth per unit distance) approximately constant. Second row. Characteristic ‘tooth’ of a nutcracker (or lobster cracker) being above the apex of other teeth (which generally line up with the equivalent axis to *L*2*M* as white dashes). Black circle rotating joint (aka ‘condyle’). Amended from https://commons.wikimedia.org/wiki/File:Casse-noix_inox_03.jpg Coyau 14 February 2013 with permission under CC BY-SA 3.0. Third row. Chelicera as a whole (with original abbreviations) from Akimov (1985) with permission. Note how diverse height teeth are ‘set’ at different angles (see Fig. 31). Bottom. Wood rasp showing rows of jagged teeth suitable for abrasive removal of material from surfaces. Amended from https://commons.wikimedia.org/wiki/File:Raspe.j_flat.jpg Tiesse amended by Westbahnhof December 2020 with permission under CC BY-SA 4.0

Examining where along the moveable digit the maximum asperity is (i.e., $x_{i}(R_{p})$) shows that in *G. domesticus* it appears to markedly jump from distal to proximal (with respect to the condyle) in line with the design ‘Type’ (Table 9). Ignoring any possibility of polymorphism within this species, this could be explained simply by small random up-down measurement fluctuations in the height of multiple teeth assayed along an overall saw-like surface. Even with assumed similar levels of measurement error, the opposite is true for *T. putrescentiae* (Table 13) where irrespective of type modelled, the maximum asperity is invariably proximal to the condyle pointing to a particular trophic specialism. This major tooth is illustrated in Fig. 12 (top row).This protruding tooth could act like (one of) the raised teeth of a wood rasp (Fig. 12 bottom row) designed to aggressively remove material quickly when scraped over material. This feature would be most useful in a browser who feeds by gleaning material off of other substrates. In that way, *T. putrescentiae* which is thought to feed on fungi infecting pollen in bumblebee nests (Roz̀ej et al. 2012, has a feeding habit action like a marine Galapagos iguana. The teeth of different heights (Fig. 12 third row) is also indicative of a possible ‘nut-cracker’ action (Fig. 12 second row). In this species the swop from type A to type B is driven rather by up/down fluctuations of the maximum gullet $R_{v}$ at its (most distal) location $x_{R{v}}$ (*results not shown*).

The maximum asperity in *C. lactis* ($R_{p}$ at $x_{R_{p}}$) is invariably proximal to the condyle where the three small teeth illustrated by Johnston (1965) and Akimov (1985) occur. Whatever is crushed at this point in the chela (held by these three tiny teeth) must be fairly soft as the velocity ratio is low (on average 0.541) irrespective of design type, inferring a *F*2 crunch force of approximately a third of that shown by the acarid and around a quarter of that in the glycyphagid (i.e., the foodstuff tucked in here is being gently squished rather than cracked, crushed or sawn). Again, in this species, swopping from design type to type is driven by up/down fluctuations of where the relative size of the maximum gullet is located (i.e., $R_{v}$ at $x_{R_{v}}$, *results not shown*).

Of note is that the $R_{t}$ values for *G. domesticus* and *T. putrescentiae* are around the diameter of various common fungal hyphae. It is worth pointing out that some plant cells are large at 70–130 μm long, so given the gape values tabulated by Bowman (2021c), any astigmatid herbivorous shredding of these would need to be of parts of the cells. However, smaller plant cells of 3–8 μm wide (as in Mahmood et al. 2005) could be easily encompassed by these gape values. On the other hand, valleys in a mastication surface affect lubrication retention and flow. So examining the $R_{v}$ values suggests that *G. domesticus* may have chela which ‘stick‘ in the food-stuff as the chela is moved through it more than the other two species, yielding *staccato* movements and pulses of extra local forces as the chelicerae themselves are dragged (saw-like) through food stuff. A more gliding movement of the digit mastication surface is expected in *C. lactis*.

How does this all relate to digit formation? The Gaussian (normal) distribution has become one of the mainstays of surface classification (Bhushan 2000). Herein in this UK beehive-based study, it is posed that surfaces are formed by cumulative developmental processes. That is, the final shape of each moveable digit region is the cumulative result of a large number of random discrete local events (irrespective of the distribution governing each individual event) producing a cumulative effect that is governed by the Gaussian form. Gaussian behaviour therefore indicates ‘all-over everywhere’ isotropic changes to the mastication surface has occurred. This is a direct consequence of the Central Limit theorem of statistical theory. Further, it is posed that single-point developmental processes (such as moveable digit elongation or flexing) are equivalent to ‘machine-shaping’ and ‘turning’ of a surface in their impact, and extreme-value developmental processes (such as any shearing/creeping in the moveable digit) are equivalent to ‘machine-grinding’ and ‘milling’ of a surface in their impact. In machine surface processing, both of these generally lead to anisotropic and non-Gaussian ‘clumped feature’ surfaces.

For Gaussian surfaces $\sigma \approx \sqrt{\tfrac{\pi }{2}}\cdot R_{a}$ or $\tfrac{\sigma }{R_{a}}\approx 1.25$, Tables 3, 7 and 11 show the results for the wild-collected sample of the three species in this review. Only the mastication surface of the UK sample of *C. lactis* approximates that expected of a random Gaussian process (i.e., the mastication profile may simply be random point location-fluctuations of any developmental process, or alternatively it is essentially an undifferentiated tweezer blade (but see *EVR* versus *O*[*VR*] *z*-test below). All other samples on average are too clumped overall, indicating non-Gaussian surface features in the profile i.e., particular differentiation is likely to have occurred somewhere along their surface during evolution. Indeed Table 11 indicates that *T. putrescentiae* is distinct (including in $\sigma ^2$) compared to a laboratory culture fed on yeast and wheatgerm.

The number of peaks ($\eta _{p}$), the number of valleys ($\eta _{v}$) and the number of ‘zero crossings‘ ($N_{0}$) (Tables 3, 7 and 11) are only broadly similar on average between the UK beehive samples. *G. domesticus* is rather saw-like, while *C. lactis* is noticeably less wavy. There is evidence that perhaps prolonged laboratory culturing on yeast and wheatgerm has reduced the waviness of the moveable digit in *G. domesticus* (cf. compare the $N_{0}$ values in Table 7) effectively smoothing it. There is a clear distinction between *C. lactis* versus the other two species if the number of crossings is rescaled by $x_{i_{e}}$ showing that the distinction is driven by the consequences of mastication surface length changes. Body size itself may not be a strong factor as the latter measurement is similar between the very different sized glycyphagid and acarid mites.

There are two possible types of ‘zero crossings‘: a gullet followed by a tooth (which causes divergent ‘tearing-apart‘ *F*2 forces above them); a tooth followed by a gullet (that cause compressive ‘squeezing‘ *F*2 forces above them); in the food material being grasped (see Fig. 2 of Bowman 2023a). Given that most free-living astigmatids have a valley behind the moveable digit tip, this suggest ‘tearing apart‘ forces on any food material held distal from the condyle, compressive forces on morsels near the middle of the mastication surface, and possible tearing apart actions closer to the condyle. This matches considering the distal parts of the moveable digit to act like vertebrate incisors/canines and the more posterior features to act like vertebrate pre-molars/molars. Modelling the surface in terms of approximating ‘hook’ shapes will examine this further (see Discussion below).

There are insufficient numbers of points along the moveable digit profile for good estimates of skewness and kurtosis of the multiple peaks and valleys so as to characterise saw-like surfaces more. A more intensive sampling in a follow-up study is needed. Note that peaks are defined as being in a ‘peak range above the sea-level’ (of the *L*2*M* axis) and the valleys in a ‘valley range below sea-level’ so that the number of crossings is not the same as the actual number of ups and downs on the mastication surface. Using the measures for elongation, bending, and shearing for the mastication surface together with the Gaussian test is an attempt to investigate this ‘orogenesis’. The non-uniformity across the three species points to the inadequacy of say the median number of peaks/valleys crossings etc., to define where morphometrics should be definitively located on each digit in a follow-up study.

### Does moveable digit ornamentation matter?

**Fig. 13:**
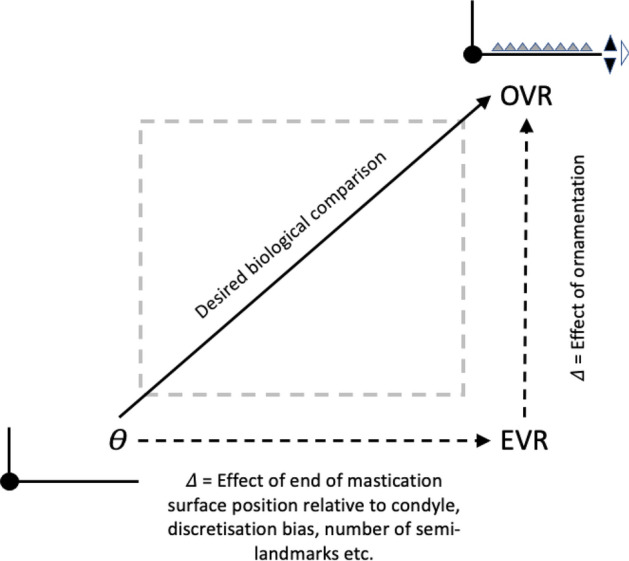
Decomposing the comparisons of interest within any functional change in moveable digit design as a tool (bottom left to top right—see Fig. 3. Grey dashed box is the trophic design space for velocity ratio *VR*. $\varDelta$ means ‘change in’. *EVR* here means specimen profile estimated

Notwithstanding any morphological differences detected, biologists would like to know if the ‘toothiness’ of jaws matters in their differential function between species. Considering the ‘average’ velocity ratio can help here. An average is an expectation often abbreviated as E[...] (see Explanatory Appendix). A path analysis approach is taken to tool comparison herein (Fig. 13).

$\varTheta$ (= the theoretical *E*[*VR*] for an un-ornamented bar-like moveable digit) is tabulated in Tables 2, 6, and 10 for the UK beehive collected specimens. This lowest on average is for *C. lactis* (0.525) and highest for *G. domesticus* (0.659) and *T. putrescentiae* (0.671). In terms of leverage against a range of foodstuffs the latter two species have similar basic chelal operating characteristics.

**Fig. 14:**
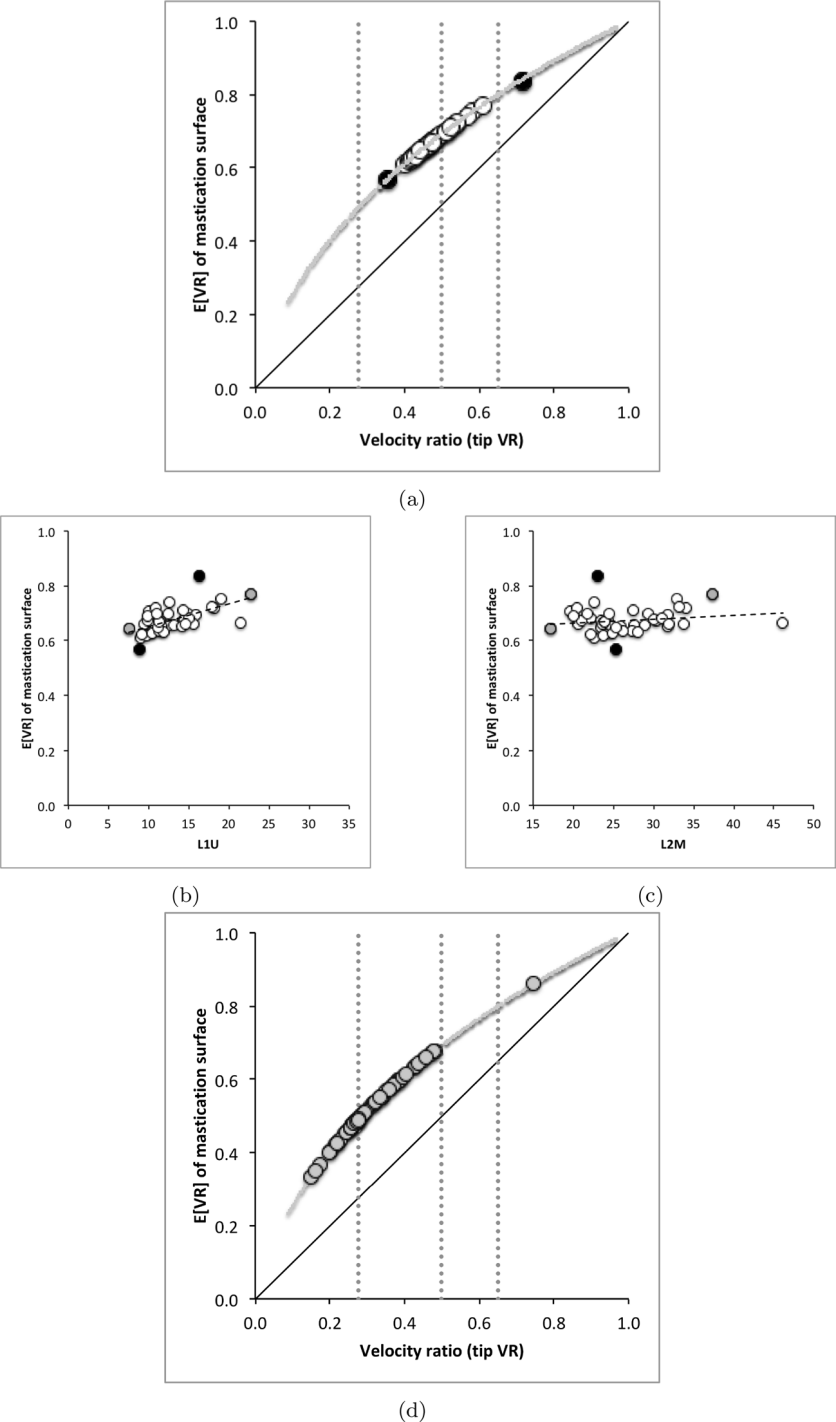
Expected velocity ratio over the moveable digit mastication surface (taken as a simple beam). **a** Versus the velocity ratio for the moveable digit tip. Over 47 reviewed taxa in Bowman (2021c). Lower black circle is Ca4 *Carpoglyphus lactis*. Upper black circle is D5 *Dermatophagoides microceras*. Power trend added in grey $y = 1.0245 \cdot x^{0.5809}\;R^{2} = 0.99821$. Note that this relationship is very similar to that of the masseter muscle (tip of jaw) velocity ratio versus masseter muscle molar m1 velocity ratio in extant and extinct mammals (Grossnickle 2020; Morales-García et al. 2021). Solid black line is $y=x$. Vertical dotted lines match design boundaries from Bowman (2021c, Fig. 27). Note asymptote at high velocity ratio values. **b** Versus *L*1*U* input moment arm. Key as before. Clear circle to far right-hand side is KL *Kuzinia laevis*. Trend line is linear but log trend very similar. **c** Versus *L*2*M* output moment arm. Key as before. Clear circle to far right-hand side is KL *Kuzinia laevis*. Trend line is linear but log trend very similar. **d** Mesostigmatids (grey circles) from Bowman (2021b) as comparators together with design boundaries

For a fixed *L*1*U*, $\varTheta$ behaves asymptotically like the function $y=\tfrac{1}{x} \cdot ln(x)$ which is approximately linear. For fixed *L*2*M*, $\varTheta$ behaves asymptotically like the function $y=ln(x)$ which over the upper range is approximately linear. Figure 14b and c illustrates this for the 47 species reviewed in Bowman (2021c). Further, from Eq. 1, if a moveable digit swells in size such that always $L1U\rightarrow b \cdot L1U$ and $L2M\rightarrow b \cdot L2M$ (i.e., linear growth or proportional conditions), then the average velocity ratio does not change. If the mastication surface $(L2M{-}L1U)$ increases in line with *L*2*M* then that is the same as linear growth or proportional conditions and the expected velocity ratio (and tip VR) does not change. Disproportionate growth (allometry) of say the moveable digit tip would yield a change in expected velocity ratio.

**Fig. 15:**
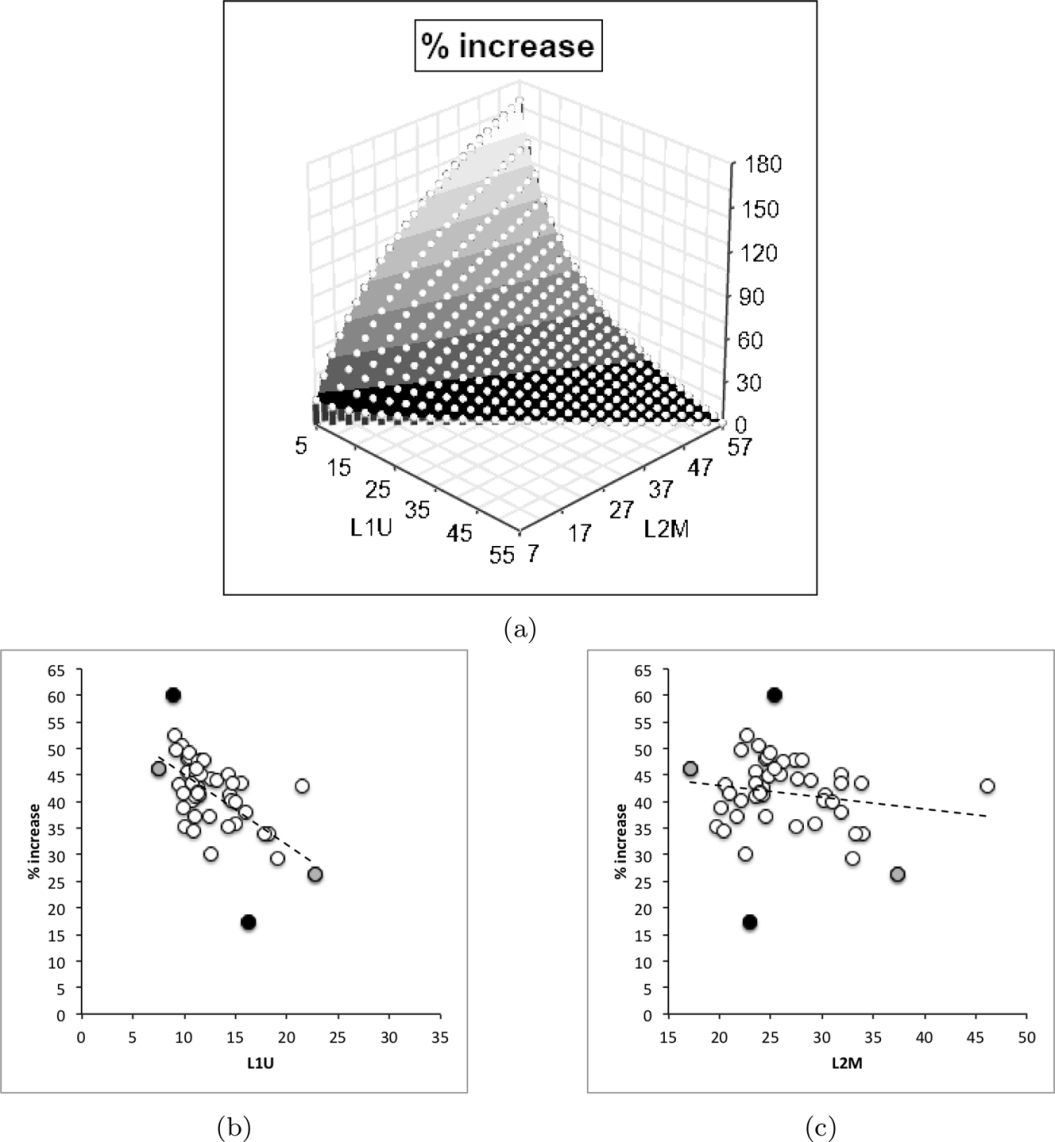
Theory informs measurements in practice. % increase of theoretical expected velocity ratio of the mastication surface (i.e., *E*[*VR*]). **a** Compared to the moveable digit tip velocity ratio for varying sizes of *L*1*U* and *L*2*M* taken from minima and maxima over the 47 astigmatid species in Bowman (2021c). Digit measurement axes are same size to avoid visual distortions. % values grid points as white circles. Grayscale contours indicate %. **b** % increase versus *L*1*U* input moment arm over 47 reviewed taxa from Bowman (2021c). Lower black circle is Ca4 *Carpoglyphus lactis*. Upper black circle is D5 *Dermatophagoides microceras*. Clear circle to far right-hand side is KL *Kuzinia laevis*. Linear trend line confirms expected decline. **c** % increase versus *L*2*M* output moment arm over 47 reviewed taxa from Bowman (2021c). Lower black circle is Ca4 *Carpoglyphus lactis*. Upper black circle is D5 *Dermatophagoides microceras*. Clear circle to far right-hand side is KL *Kuzinia laevis*. Linear trend line confirms decline

Calculating the % increase of the *E*[*VR*] values compared to the tip *VR* is shown in Fig. 15 (note axes re-orderings). This infers that when the moveable digit tip velocity ratio values are low ( $\equiv$ elongate chela, e.g., $L2M=57$, $L1U=5$ μm) the expected velocity ratio over the whole surface is much higher than indicated by that of the tip. This is confirmed in Table 15 and Fig. 14a using the data from Bowman (2021c), especially so for dainty chelae as in *C. lactis*. Again (see right vertical flank on Fig. 46 in the Explanatory Appendix), those chelae with lower and lower tip velocity ratio values as *L*2*M* increases (for a given *L*1*U*) may have higher expected velocity ratio values than the tip, but still decline broadly hyperbolically and have expected velocity ratios for their likely mastication surface closer and closer to the $VR_{tip}$ value. In other words, the overall food handling behaviour of lengthening elongate chelae is dominated by the tip *VR* value.

Note that for an already elongate chela there is a diminishing return in the change in the expected velocity ratio for the digit getting even longer (Fig. 46c in the Explanatory Appendix). This suggests that any evolutionary penalty for elongation is low and perhaps may explain ‘runaway’ morphological processes such as stag beetle mandibles, veigaiid chelae etc., (see Bowman 2021b). This could be the case for *C. lactis* too. However, there is a downside to such since work in scorpions (van der Meijden et al. 2012) has shown that elongate chelae are at a higher risk of failure when operating near their maximum pinch force making them less suitable for high-force functions such as burrowing (or indeed defence).

Conversely however, now for a fixed *L*2*M* (e.g., $L2M=57$ μm in Fig. 46 in the Explanatory Appendix), as the moveable digit tip velocity ratio of the chela becomes larger on *L*1*U* increasing (see left vertical flank in Fig. 44 in the Explanatory Appendix), the expected velocity ratio surface is curved (see left vertical flank of Fig. 46 and the clear gap in that left vertical flank). So, increasing the input moment lever arm produces disproportional gains—these gains are higher at low tip velocity ratios (i.e., for elongate chelae) and decline as chelae become more ‘stubbier’. The % gain of expected velocity ratio over the tip VR is shown in Fig. 15﻿a and confirmed in practice (Fig. 15b and c). Cheliceral height matters in astigmatids.

Maintaining or increasing the input moment lever arm as adductive muscles move backwards (and like vertebrate jaw temporalis muscles pull more horizontally Crompton and Parkyn 1963) during any evolutionary elongation of the feeding apparatus in acarines (see Grandjean 1947; van der Hammen 1970a, b) will be an important driver of the development of a ‘coronoid’ process (e.g., as in synapsid reptile jaws DeMar and Barghusen 1972). How the ascending ramus might develop within this process is not clear.

Table 16 appears to show no importance of ornamentation. However, if the end of the mastication surface used in the *O*[*VR*] calculation (for the actual moveable digit surface) is very different than the distance of *L*1*U* from the condyle along the *L*2*M* axis (as used in the definition of $\varTheta$) then the former estimate may diverge widely from the theoretical $\varTheta$ value solely for that reason. Note that also when calculating the observed actual average velocity ratio of a digitised image with asperities/gullets etc., due to the concavity of the line functional form behind the adductive mechanism, at around the granularity used in this study (18 points), the theoretical expected ratio ($\varTheta$) is a little too conservative. Simulation studies *not shown* suggest that this discretisation bias is small < 6%. So, a better estimate for *E*[*VR*] under a beam-like assumption can be obtained by calculating the measured conditional

$$\begin{aligned} \widehat{E[VR]}=\frac{1}{q} \cdot \sum ^{i_{e}}_{i=1} \left( \frac{L1U}{x_{i}} \right) \end{aligned}$$

where *q* is the number of digitised $x_{i}$ locations along the *L*2*M* axis including the moveable digit tip and the end of the mastication surface. This also allows for any observed shift in end of the mastication surface (from being taken to be $\equiv L1U$ from the condyle).

*z*-tests for any departure of the observed average velocity ratio *O*[*VR*] versus this $\widehat{E[VR]}$ null expectation of a simple bar-like moveable digit are shown in Table 17. This suggests that the dentition pattern for each beehive-collected species, in terms of the mechanical efficiency of their mastication surface taken as a whole is different than that of a simple beam-like bar (i.e., $abs(z) > 1.96$) given the observed $VR_{tip}$ values. Digit ornamentation matters in these mites.

This hypotenuse-based measurement approach is a useful assay of ‘toothiness‘, the effective mastication surface velocity ratio slightly declining with the ornamentation as expected for each specimen. Asperities and or gullets matter in terms of the actual average mechanical function. These samples from the wild show clear evidence of a trophically relevant moveable digit surface morphology differing from any unornamented plesiomorphic state (of a scale that is broadly similar in each species). This is confirmed for laboratory cultured specimens and even for the heterogenous museum sample of *T. putrescentiae*.

Of course, the assumption of essentially a zero-depth *L*2*M* axis for the ‘tooth row’ in the null expectation $\widehat{E[VR]}$ might be a tad unrealistic. A plesiomorphic bar would have some thickness. Some of this might have been effectively above the tip to condyle *L*2*M* axis. In follow-up work one could start with an empirical null assuming say a uniform ‘no-tooth’ beam of thickness of $0.5.R_{a}$ as a basis for a hypotenuse-based formula like the *O*[*VR*] formulation in the expected results section (see Explanatory Appendix). Indeed, even then the active chewing region of this ‘null thin beam’ probably declined in depth in some sort of smooth linear/hyperbolic way, in line with resisting bending and breaking forces downwards on chelal closure (Fig. 2 in Bowman 2023a). Given an assumption that longitudinal reinforcement was in the ventral areas of the digit (i.e., in the ‘saw-plate’), one could consider apportioning the total $0.5.R_{a},x_{I_{e}}$ volume in a suitable cascading manner towards the digit tip when calculating $L2M'$ for each location.

**Fig. 16:**
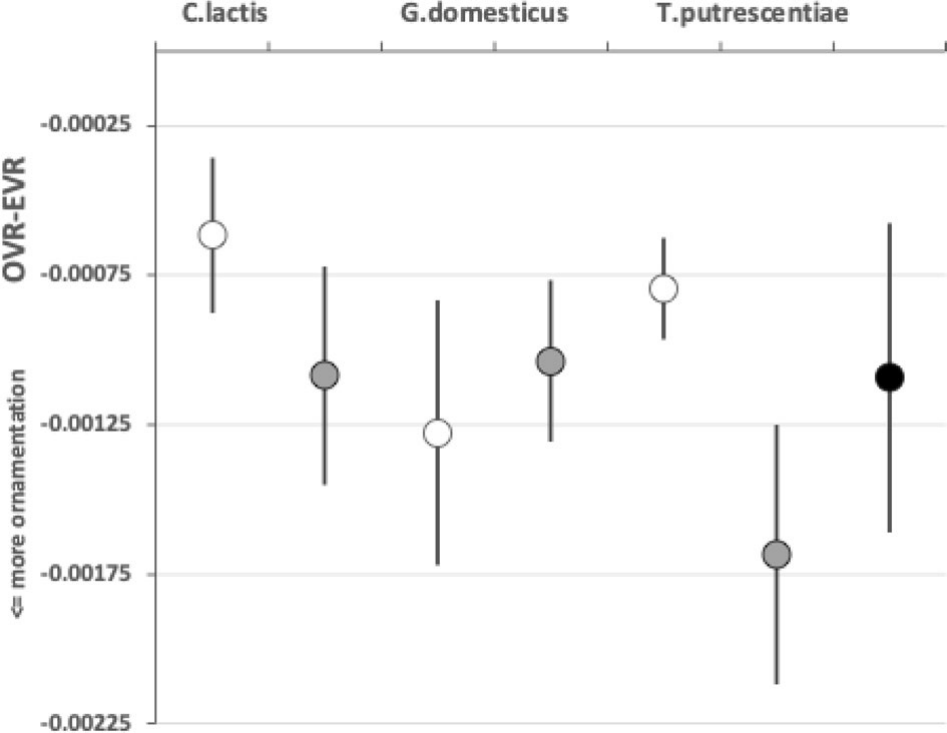
95% confidence intervals for degree of ornamentation in the three UK beehive species. Open circles = mean over wild collected specimens. Note almost non-overlap of the least differentiated *Carpoglyphus lactis* and the most differentiated *Glycyphagus domesticus* confidence intervals indicating that a significant between species change was almost shown. Grey circles = mean of laboratory specimens. Black circle = mean of *Tyrophagus putrescentiae* museum specimens. All species have significantly derived moveable digits (see Table 17)

The fact remains that Fig. 16 illustrates the clear progression of the degree of ornamentation in the wild, from *C. lactis* (least), through *T. putrescentiae* to *G. domesticus* (most) with almost non-overlapping 95% confidence intervals between the two extreme species. Dental features above the level of the the level of the moveable digit tip would have the overall action of driving food material backwards towards the condyle, those below it ejecting material forwards out of the chela with respect to any moveable digit tip strike into foodstuffs.

Table 18 shows that all samples bar *C. lactis* UK beehive individuals, have undergone morphological changes in the chela itself such that the assumption that the mastication surface ends at an equivalent distance to *L*1*U* from the condyle is unlikely (indeed most samples give an excess of higher velocity ratio values in the observed estimate). Using a traditional meta-analytic statistical adjustment of weighting each individual’s difference values by one over the variance of their estimates (i.e., the reciprocal of $var(\text {mean\; of\; values})$ over the mastication surface which is $\propto \;i_{e}$) confounds this and not removes it (*results not shown*).

**Fig. 17:**
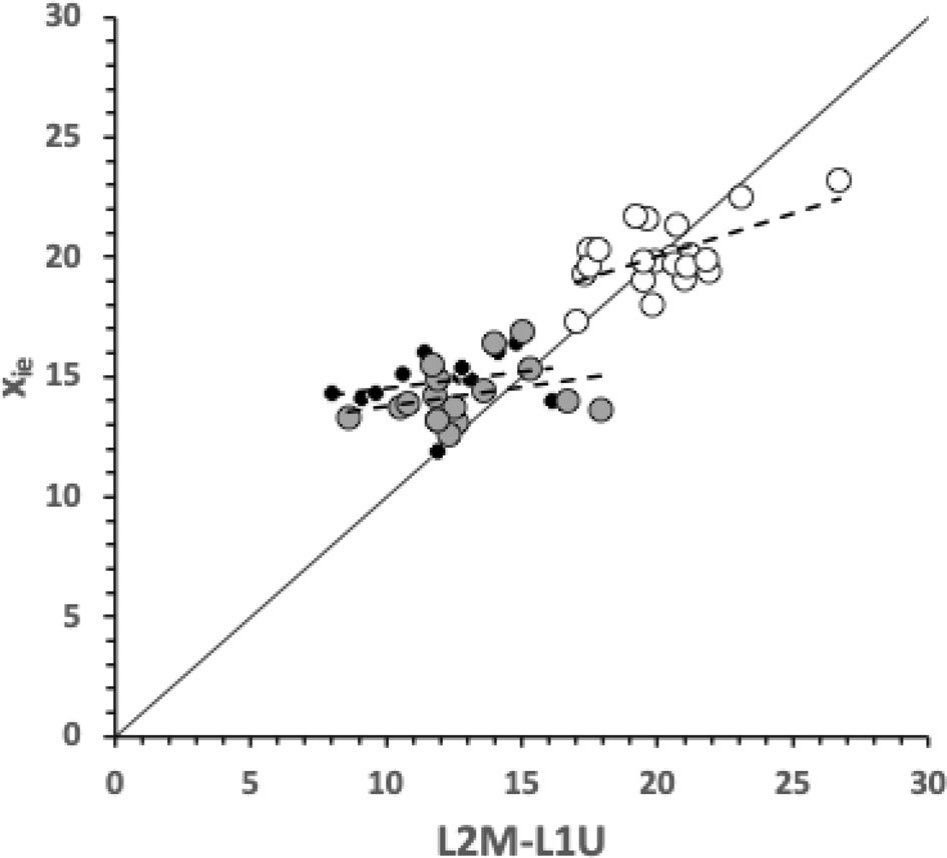
Actual ‘tooth row’ ($x_{i_{e}}$) versus theoretical potential ‘tooth row’ ($L2M{-}L1U$). Open dots = *Carpoglyphus lactis* (includes distal blade area). Grey dots = *Tyrophagus putrescentiae*. Black dots = *Glycyphagus domesticus*. Species with dashed linear regression lines. Solid line is $y=x$

Indeed, Fig. 17 shows that in general the actual ‘tooth row’ length ($x_{i_{e}}$) is fairly constant over a variety of potential ‘tooth row’ lengths (as measured by $L2M{-}L1U$). So, mites do show adjustments to maintain a standard spacing of standard width dentition within species (certainly for the acarid and glycyphagid by similar amounts). If one assumes that morphologically about 50% of the moveable digit in *C. lactis* is available for dentition (aka for the three posterior proximal teeth) then even this species falls into line (cf. translate the cloud left and down along the unity line in Fig. 17). This suggests that three processes: the enhancement of the coronoid process, surface regionalisation (perhaps by digit tip elongation), and changes in height of asperities upon a standard astigmatid chelal Bauplan is the way to look at their comparative evolution (and thus the definition of future informative geometric morphometric measures). Regarding the coronoid size and shape, at least in humans this varies within and between individuals (Sahithi et al. 2016) and between genders (Pradhan et al. 2014; Subbaramaiah et al. 2015; Subbaramaiah and Jagannatha 2018). How the ascending ramus might form from the horizontal ramus posterior of any teeth awaits elucidation.

So, ornamentation does matter but other mechanical aspects will matter too. Features in the mastication surface proximal to the condyle have a larger impact in maintaining a high average *VR* for the surface than those features proximal to the moveable digit tip (which would reduce the overall average). In other words crushing teeth (and opposing gullets on the complementary fixed digit) should be expected nearer to, rather than farther from, the condyle—bigger such features engendering higher *VR* values and consequent *F*2 crunch force. This concurs with the results of Dalrymple (1979a) who showed *Dracaena* as a snail-crushing lizard specialist to have posterior-most teeth closer to the jaw articulation than *Tubinaphis*. Fig. 11 illustrates that the moveable digit of *Carpoglyphus lactis* does have three teeth but they are posteriorly positioned. Here they would exert a decent crushing force on small items of food material held posteriorly between the digits like vertebrate molars. Similarly, elongate moveable digits of low *VR* would be expected to have few if any teeth near their tip—as for a fixed ‘stretching’ of the digit surface (up/down), having teeth at high *L*2*M* values is disfavoured compared to them having low *L*2*M* values near the condyle. Akimov and Gaichenko (1976) consiliently illustrate that the moveable digit of *Carpoglyphus lactis* has no teeth distally but is thus blade-like near the digit tip.

The same logic applies to *grouping* teeth (or gullets). There is more ‘bang for your buck’ in these features being nearer the condyle than the moveable digit tip. Ungulate herbivores have groups of crushing teeth posteriorly and often have gaps to the front ‘nibbling’ teeth. Lack of teeth i.e. a blade-like form in the moveable digit surface, if present like in predatory mesostigmatids, is thus expected distal of the condyle. Any teeth taller nearer the digit tip than lower teeth further back accelerate material back onto the latter. So lowering the condyle with respect to the moveable digit tip, whilst increasing the adductive moment lever arm (DeMar and Barghusen 1972) also ensures that most food material is propelled backwards on chelal closure. Having a digit articulation positioned above the moveable digit teeth (as in vertebrate herbivore jaws Smith and Savage 1959) would ensure food is propelled forward on jaw occlusion. It is not clear how this would be advantageous to a saprophagous astigmatid if such a relative positioning of the condyle occurred. Moving the condyle backwards may increase *L*1*U* but decreases gape and impacts upon space for cheliceral musculature—the chelicera height would have to rise to maintain an appropriate angle of insertion (DeMar and Barghusen 1972). Indeed, this may be the origin of the particular cheliceral basal shape in the Algophagidae (Fashing 1998).

The pattern of the direction of forces exerted by an ornamented moveable digit on chelal occlusion has a further advantage. For example, Fig. 2 of Bowman (2023a) shows locations $i=2, 11$ has forwards-up force vectors, and locations $i=3\ldots10$ has backwards-up vectors with respect to the upwards tangential force on the moveable digit tip at $i=1$. This ensures that any food morsel is subject to a tear between $i=2$ and $i=3$ (and after $i=11$ where the fixed digit edge plunges downwards) with the morsel being compressed backwards over the mastication surface at $i=4...10$. The sharpness of the moveable and fixed digit tips pierce into food material, with any deep moveable digit gullet below the *L*2*M* axis just behind the moveable digit tip effectively ejecting pierced fragments out of the chela. The digits have thus a simultaneous: stab, tear, and chew action (just as in vertebrate incisors, canines and pre-molars/molars respectively). Having a blade shape form to those parts of features (whether tips, teeth or gullets) on their condylar side means that any retraction of the whole chelicera backwards will engender slicing of semi-grasped material as it thus moves and slides through the chelal grasp.

The Explanatory Appendix gives

$$\begin{aligned} \varTheta = \frac{L1U}{(L2M{-}L1U)}\cdot log_{e}\frac{L2M}{L1U} \end{aligned}$$

Another way to look at this Equation (1) is that

- the first term $\tfrac{L1U}{(L2M{-}L1U)}$ is the tip velocity ratio if the condyle was shifted to the posterior-most part of the mastication surface i.e., at $x_{i_{e}}$. So, as an ’elongation index’, being the length of *L*1*U* in terms of the ‘potential tooth row’ i.e., an abbreviated *L*2*M*, this is analogous to MAT m1 in Grossnickle (2020) and Morales-García et al. (2021))
- and, the second term $log_{e}\tfrac{L2M}{L1U}$ is another ‘elongation index’ (being the natural logarithm of the length of L2M in terms of units of L1U i.e., $log_{e}{VR^{-1}}$).

**Fig. 18:**
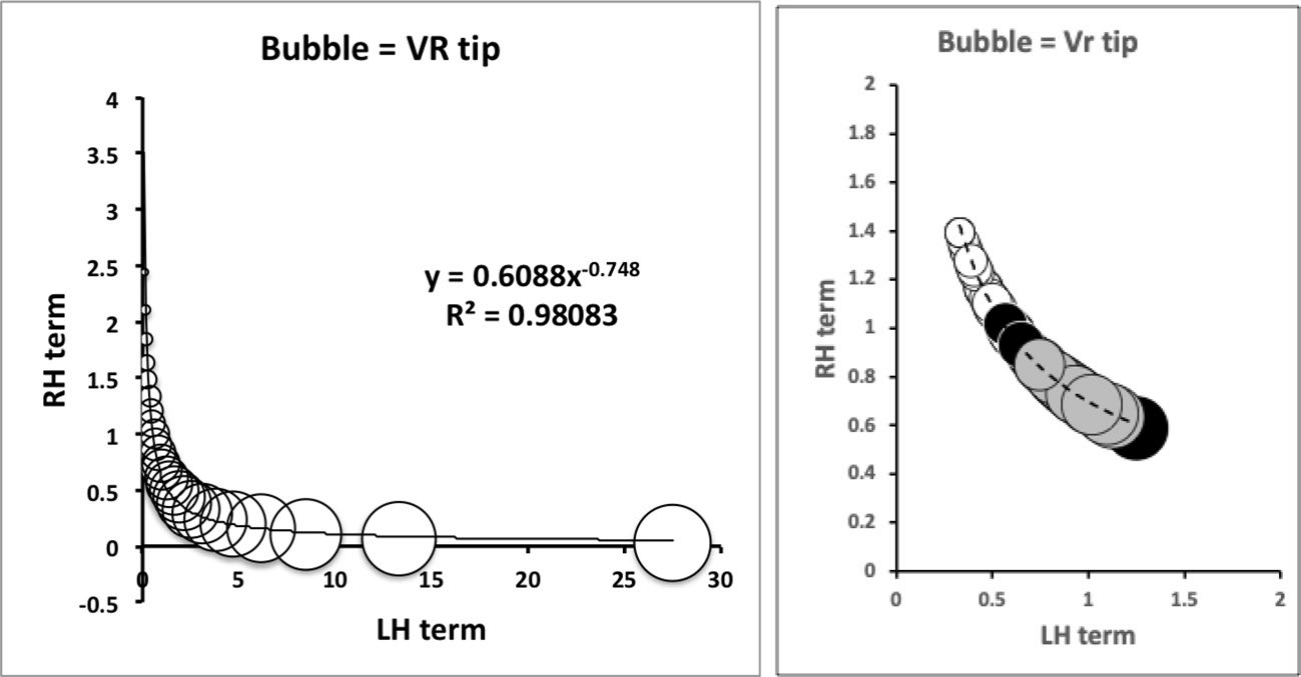
Left hand term versus right hand term of Eq. 1. On left: Theory. Note how at large tip velocity ratio values the left-hand term dominates, at low moveable digit tip values the right-hand term dominates. Fitted indicative power trend line. On right: Observations. Dashed power regression line $y=0.6978x^{-0.643}\; R^{2} = 0.9983$. Open circles = *Carpoglyphus lactis*. grey circles = *Tyrophagus putrescentiae*. Black circles = *Glycyphagus domesticus*. The plots for ${bubble\; size} = E[VR]$ is very similar (not shown)

These ‘aspect ratios‘ interplay with each other such that when one is high, the other is low and vice versa (Fig. 18). In *C. lactis* the RH term dominates, in *G. domesticus* the LH term dominates.

Overall, Eq. 1 could be considered as a *composite* elongation index or composite aspect ratio summarising the whole mastication surface as ‘a tool of many useful features’.

Recalling that

$$\begin{aligned} \varTheta = \frac{L1U}{(L2M{-}L1U)}\cdot log_{e}\frac{L2M}{L1U} \end{aligned}$$

The fitted power line in Fig. 18 suggests that given

$$\begin{aligned} \varTheta =LHS\cdot RHS \end{aligned}$$

where

$$\begin{aligned} LHS=\frac{L1U}{(L2M{-}L1U)} \end{aligned}$$

and the fitted

$$\begin{aligned} {\widehat{RHS}}\approx 0.6 \cdot (LHS)^{-0.75} \end{aligned}$$

then

$$\begin{aligned} \varTheta \approx \frac{L1U}{(L2M{-}L1U)}\cdot 0.6 \cdot \left( \frac{L1U}{(L2M{-}L1U)}\right) ^{-0.75} \Rightarrow 0.6 \cdot \left[ \frac{L1U}{(L2M{-}L1U)}\right] ^{0.25} \end{aligned}$$

The last form of this relationship is an easy measure for taxonomists to use when describing new species since: $(L2M{-}L1U)$ could be considered as the (full length of a *potential*) tooth row (including gullets) along the moveable digit (see Tables 2, 6 and 10); and, $L1U\approx 0.245\times CHI\;(R^{2}=0.9797,$ individual data from Bowman 2021c), where *CHI* is the cheliceral segment height. Note that this horizontal ramus tooth row is therefore longer than the observed parameter 10 in Buryn and Brandl 1992, or morphometric 13 used in Liu et al. 2017 for mesostigmatid mites.

For fixed tooth row size (> 0), this means that $\varTheta$ scales as to the fourth root of the input moment lever arm length (*L*1*U*). So under these conditions, ever increasing *L*1*U* produces less than proportional gains once initial growth in $\varTheta$ occurs (i.e., on the coronoid process/ascending ramus first appearing during evolution). $\varTheta$ also scales as to the fourth root of the input moment lever arm length expressed in units of this tooth row parameter. Again showing less than proportional gains providing digit growth vertically and horizontally is kept in sync (i.e., proportional or $b=\text{constant}$ for $L2M=b\cdot L1U$). Conversely for fixed *L*1*U* (and $L1U<L2M$), the theoretical expected velocity ratio scales with ${tooth \;row\; size}^{-0.25}$. So in elongate chelae the gain is almost linear, but major falls occur in $\varTheta$ if the tooth row increases during evolution when it was originally at low values. The approximate breakpoint (*result not shown*) between these latter two behaviours is around a $\varTheta \approx 0.5$, or $VR_{tip}\approx 0.7$, well above the typical free-living saprophagous astigmatid values (Bowman 2021c, Fig. 27). This suggests that major changes in digit design may be important in oribatid primary and secondary decomposers but that the three astigmatid taxa studied herein all sit in the single space of crushing-style mesostigmatid designs (see Bowman 2021b).

Now Bowman (2021b, c) shows that *L*2*M* is indicative of moveable digit length (*MDL*) and *L*1*U* is correlated with cheliceral height *CHI* in mites in general. *CHI* is correlated, given the lack of any dorsal constraint by a gnathotectum in astigmatids, with chelal crunch force (*F*2). So if the tip velocity ratio given an effective rotation point shift to the most posterior part of the moveable digit mastication surface is kept fixed during development or fixed across species evolutionarily, then $\varTheta$ (the expected velocity ratio of the whole moveable digit) scales log linearly with chelal relative elongation (i.e., chelal aspect ratio). Given the strong correlation of cheliceral aspect ratio with chelal aspect ratio (Fig. 24c in Bowman 2021c) then this is a clear explanation of the concerted evolutionary path to trophic adaptation in these mites.

Furthermore, the chelal crunch force (*F*2) is $=F1 \cdot VR$, so the expected chelal crunch force over the mastication surface $E[F2]=F1\cdot \tfrac{L1U}{(L2M{-}L1U)} \cdot log_{e}\tfrac{1}{VR_{tip}}$ In other words, the overall ability to tackle hard versus soft food of different sizes depends upon the composite elongation index. That is, dependant upon how diminishingly elongate a species’ cheliceral chela is ($log_{e}\tfrac{1}{VR_{tip}}$) and what for that species is the pulling force (*F*1) on the chelal adductive tendon relative to the mastication surface translated‘ velocity ratio’ ($\tfrac{L1U}{(L2M{-}L1U)}$). One concludes that, for at least their ‘jaws’, aspect ratio is an over-arching functional design factor in these mites, not just being used by morphologists to partial out absolute scale differences between astigmatid species.

Clearly any pointed teeth locally concentrate forces applied by a jaw or the chelicera (e.g., the pointed hypapophyses in egg-eating snakes), and conversely sharp changes in surface contours like V-shaped gullets (Frost 1971) give rise to areas of high risk of mechanical failure in the crushing tool. One would thus expect extra sclerotisation under these ‘stress risers’ to obviate the ‘notch effect’. Further microscopic work could look for this.

Bowman (2021b) found that adductive force on the cheliceral tendon (*F*1) scaled with $\text {intra-cheliceral shaft volume}^{0.8}$ and *F*2 scaled close to the theoretical relationship for all animals (i.e., $\text {body size}^{\frac{2}{3}}$) in mesostigmatids (where $\text {body size}=IL^{3}$). Examining the summary figures from Bowman (2021c) shows a similar relationship for *F*1 for astigmatids but no clear scaling of *F*2 with body size. For this study herein using $F1\approx CHI^{2}$ (as in Perdomo et al. 2012) the relationship with $\text {intra-cheliceral shaft volume}^{0.8}$ is again found and now indications that *F*2 (calculated as $VR\cdot PHI^{2}$) scales with $body\ size^{\frac{2}{3}}$ both within each sample and across samples also is found.

It is stressed that $\varTheta$ (the expected velocity ratio) is conditional upon that particular beam-like structure for the moveable digit. It is not the velocity ratio (for that *L*1*U*) for the *zenith* of a tooth or the *nadir* of a gullet located that far from the condyle *along the bar itself*, which would be $VR^{\ast}=\tfrac{L1U}{condyle\ to\ tooth\ or\ gullet}$ (see Fig. 2). Similarly no allowance has been made for any change in the subtended angle of the adductive force along the chelal closing tendon, nor any projection due to the nadir or zenith of features being be above or below the notional beam.

### How differentiated are chelal moveable digits as poly-functional tools?

**Fig. 19:**
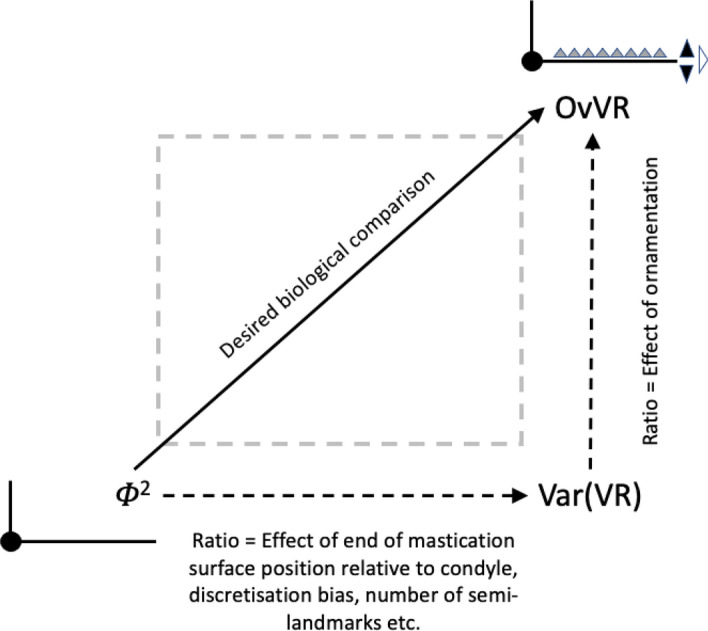
Decomposing the comparisons of interest within any functional change in moveable digit design as a tool (bottom left to top right—see Fig. 3. Grey dashed box is the trophic design space for the velocity ratio *VR*. *Var*[*VR*] here means specimen profile estimated

Notwithstanding any morphological differences detected, biologists would like to know if the ‘spikiness’ of jaws matters in their differential function between species i.e., what does the within individual variation of ‘toothiness’ look like? Considering the velocity ratio variance (*Var*[*VR*]) can help here. A path analysis approach is taken to tool comparison herein (Fig. 19).

A large *Var*[*VR*] indicates the possibility of more *different* mechanical behaviours along the digit. Numerically solving for when the slope of *Var*[*VR*] is zero with respect to $VR_{tip}$ shows that this null value is a maximum when $VR_{tip}\approx 0.178$ i.e., the opportunity for variability in velocity ratio values over the mastication surface in an un-ornamented moveable digit is greatest around the observed moveable digit tip velocity ratio values of veigaid chelae (see Bowman 2021b). So the theoretical trophic opportunity for a poly-functional mastication surface increases with elongation of the moveable digit (relative to the input moment arm) up to this, almost certainly, practical limit of the condylar joint handling and keeping stable a very elongate digit that would naturally ‘waggle‘ and dislocate itself. An un-ornamented moveable digit in *C. lactis* thus would appear to be designed more for, in part, cutting than crushing, whilst those for the acarid and glycyphagid (even without dentition) would appear to better match the trophic bandwidth of typical single style crushing-action designs. What material could it be that *C. lactis* might slice? Can even more excessive moveable digit length carpoglyphid sub-populations be found in beehives or elsewhere?

Tables 2, 6, and 10 show $\varPhi ^{2}$ values for each specimen including substantiating the design of *C. lactis* with its lower moveable tip velocity ratio, and the lower expected velocity ratio ($\varTheta$) of the mastication surface having a higher variance for that velocity ratio. Another way of looking at $\varPhi ^2$ is that it indicates the (theoretical) null ‘profile spikiness’ or ergodicity of the velocity ratio values over the mastication surface for that design of chela. In other words, the moveable digit of *C. lactis* has (*even if there is no extra ornamentation*) a wider variety of velocity ratio values along its length than that of the acarid or glycyphagid. A chela designed as a tweezer can grip a large variety of objects including small fragments positioned near the condyle where they may be bitten strongly. However, a ‘stubbier’ more powerfully designed short moveable digit chela cannot behave like a tweezer and is effectively consolidated to single function, i.e., a narrow set of velocity ratio values. Behavioural multi-functionality in a mastication surface is therefore facilitated by chelal elongation. Analogously, omnivorous dogs and pigs have a longer mandible than more specialised predators like cats amongst vertebrates (see Bowman 2021b for mesostigmatid mite examples). Are shorter moveable digit oribatids behaviourally specialist *per force*.

As with the *O*[*VR*] formulation in the expected results section (see Explanatory Appendix), the variance of the observed velocity ratio values over the sampled digitised locations of the ornamented mastication surface (i.e., the observed ‘spikiness‘) for a specimen can be calculated as

$$\begin{aligned} Ov[VR]=\frac{1}{q-1} \cdot \left[ \sum _{i=1}^{i_{e}}(VR`_{i}-O[VR])^{2} \right] \end{aligned}$$

these figures being shown in Tables 4, 8 and 12. Note again when calculating the observed actual velocity ratio variance of a digitised image with teeth/gullets etc., that due to the concavity of the line function, the variance $\varPhi ^{2}$ is a little too conservative (i.e., mild discretisation is present). An estimate for *Var*[*VR*] on a beam-like moveable digit form (allowing also for $x_{i_{e}}$ varying with respect to the condyle) can be obtained by calculating

$$\begin{aligned} \widehat{Var[VR]}=\frac{1}{q-1} \cdot \left[ \sum ^{i_{e}}_{i=1} (\tfrac{L1U}{x_{i}})^{2}-(q \cdot (\widehat{E[VR]})^{2})\right] \end{aligned}$$

where *q* is the number of digitised $x_{i}$ locations along the *L*2*M* axis including the moveable digit tip and the end of the mastication surface. This $\widehat{\varPhi ^{2}}$ like *Ov*[*VR*], allows for any variation in the relative positioning of the condyle with respect to the end of the mastication surface. Tables 4, 8 and 12 shows these figures. How to test these?

**Table 8:** *Glycyphagus domesticus*. Stochastic characterisation

Specimen	$\widehat{E[VR]}={\hat{\varTheta }}$	Ov[VR]	$\widehat{Var[VR]}=\widehat{\varPhi ^{2}}$	$\frac{1000\cdot R_{t}}{IL}$
224(2)-1	0.582	0.0166	0.0169	12.88
224(2)-2	0.669	0.0285	0.0299	16.38
224(2)-3	0.620	0.0186	0.0187	8.82
224(2)-4	0.589	0.0222	0.0227	11.93
224(2)-5	0.516	0.0166	0.0173	18.85
224(2)-6	0.795	0.0410	0.0416	12.91
224(2)-7	0.735	0.0358	0.0368	12.26
224(2)-8	0.706	0.0316	0.0317	9.76
224(2)-9	0.578	0.0120	0.0121	10.30
224(2)-9a	0.707	0.0240	0.0242	8.23
224(1)-10	0.572	0.0216	0.0226	15.25
224(1)-20	0.632	0.0190	0.0193	13.60
Summary	0.642	0.0226	0.0231	12.60
	(0.0814)	(0.00865)	(0.00882)	(3.144)
G5 (n = 20)	0.624	0.0189	0.0192	14.15

Summary is mean, median or geometric mean as appropriate. SD in (....)

Firstly, recall the standard result that the sampling distribution of a variance $S^{2}$ given its fixed true scalar value $\sigma ^{2}$ is defined by $\tfrac{(n-1)\cdot S^{2}}{\sigma ^{2}} \sim \chi ^{2}_{(n-1)}$ where *n* is the number of elements making up that variance. In other words if the hypothesised value was 7 and there were $i=1\ldots31$ measurements $y_{i}$ on a single specimen say, then conservatively $\sum _{i=1}^{31}(y_{i}-{\bar{y}})^{2}$ can be tested against $\chi ^{2}$ values with 30 degrees of freedom and the null (traditionally) rejected if the calculated figure exceeds the tabulated $p=0.05$ value. The $(n-1)$ index is used as the mean (${\bar{y}}$) has been estimated from the data.

However, in the case of the astigmatids in this study, each specimen yields its own fixed theoretical $\varPhi ^{2}$ null value and a ‘paired’ $\widehat{Var[VR]}$ value from its moveable digit digitised profile. Ignoring, in the first instance, the small change in the number of points used and the mastication surface relationship to condyle position varying between each specimen in $\widehat{Var[VR]}$, and that both values are conditional on the moveable digit tip value, then each individual (*j*) contributes a $\frac{S_{j}^{2}}{\sigma _{j}^{2}}\equiv \frac{\widehat{Var[VR]}_{j}}{\varPhi _{j}^{2}}$ or a $\chi ^{2}$ variate.

A standard result is that a sum of *k* identical independent $\chi ^{2}_{(1)}$ variates is $\sim \chi ^{2}_{(k)}$. Ignoring that the null varies slightly across individuals (i.e., treating it as a pre-specified fixed scalar), one wants to know if there is evidence of departures from the overall null for that species i.e., if the set of $\chi ^{2}$ variates from the $j=1\ldots k$ individuals for that species are overall different from 1. A simple approximate way is to sum these ‘within-specimen’ $\chi ^{2}$ variates over the *k* specimens for that species, and then test this versus $\chi ^{2}_{(k)}$ i.e., by calculating $\sum _{j=1}^{k}\frac{S_{j}^{2}}{\sigma _{j}^{2}}\equiv \sum _{j=1}^{k}\frac{\widehat{Var[VR]}_{j}}{\varPhi _{j}^{2}}$.

However, due to the sources of extra variation feeding through, this arithmetic sum may be too big in practice and thus facilitate rejecting the overall null. For instance $\varPhi _{j}^{2}$ now being a random variate is not a fixed scalar of the same value $\forall \ j$. A smaller denominator will effectively over inflate $\widehat{Var[VR]}$ and encourage rejection of the null. A conservative heuristic (given that the geometric mean of a set of numbers is always smaller than or equal to its arithmetic mean) is instead to calculate *k* times the geometric mean of these $\frac{S_{j}^{2}}{\sigma _{j}^{2}}$ values instead i.e., $k \times \root k \of {\varPi ^{k}_{j=1}}(\frac{\widehat{Var[VR]}{j}}{\varPhi _{j}^{2}})$ and compare it to % probability points for $\chi ^{2}_{(k)}$. In this case data departures need to be large and obvious before the null is rejected (especially useful in small sample studies like this).

So, calculating from the values in Tables 4, 8 and 12 for the UK beehive sampled *C. lactis*, *G. domesticus* and *T. putrescentiae* yields $\chi ^{2}_{(21)}=21.0666$, $\chi ^{2}_{(12)}=12.0375$ and $\chi ^{2}_{(17)}=16.7214$ respectively. All are $p>0.05$ as intuition would suggest. An estimate of *VR* variability for a beam should be near its theoretical value. Unlike the tests of the expected velocity ratios above, this does not suggest that any systematic departures from base assumptions (e.g., due to the distance to the condyle along the *L*2*M* axis from the end of the mastication surface in practice varying from the theoretical one *L*1*U* unit, concavity of function bias etc.,) have an impact upon the basic ergodic *VR* ‘spikiness’ of a non-ornamented beam for these beehive species.

Secondly, recall that for two estimated sample variances ($S_{a}^{2}, S_{b}^{2}$), a ratio of two independent $\chi ^{2}$ statistics i.e., a *F* statistic test for any departures from the null that the variances are equal to each other, is $\frac{S_{a}^{2}}{S_{b}^{2}}\sim F_{(n-1,m-1)}$ with *n* and *m* here being the number of elements making up those variances. One could argue that this ratio for this review is in some sense ‘paired’ as the extra sources of variation for a particular specimen for the numerator and the denominator are the same. However, at least the measurement variation may be different between the two as internal to *Ov*[*VR*] it relies upon the *y* measurement of the profile (for ornamentations) as well as the *L*2*M* *x*-axis in its constituents, whilst $\widehat{Var[VR]}$ only relies upon constituents derived from the *L*2*M* *x*-axis.

So a conservative heuristic argument like that above can be applied to the standard result as for sure there is real inter-individual variation in both the observed assessment $\widehat{Var[VR]}$ and its ‘paired’ *Ov*[*VR*] in this small sample sized study. For a set of $j=1\ldots k$ individuals in a species then $\frac{\chi ^{2}_{a}}{\chi ^{2}_{b}}$ for an overall test $\approx \frac{ k \times \root k \of { \varPi ^{k}_{j=1}Ov[VR]_{j} }}{ k \times \root k \of {\varPi ^{k}_{j=1}\widehat{Var[VR]_{j}}}}=\root k \of {\varPi ^{k}_{j=1}\frac{Ov[VR]_{j}}{\widehat{Var[VR]_{j}}}}$ which can be compared to % probability points for $F_{(k,k)}$. In a small study one does not want outliers to determine the inference of whether hypothesised differences are likely to be true too strongly.

Indeed, using the values in Tables 4, 8 and 12 for the UK beehive sampled *C. lactis*, *G. domesticus* and *T. putrescentiae*, gives $F_{(20,20)}=0.9996$, $F_{(11,11)}=0.9982$ and $F_{(16,16)}=0.9994$ respectively (ignoring the paired nature of the data). All are of similar scale and $p>0.05$, so the mastication surface profile *VR* spikiness allowing for any *VR* ‘toothiness’ i.e., asperities and gullets being present, is not different than that expected for a sampled simple beam-like bar given that moveable digit $VR_{tip}$ value.

**Fig. 20:**
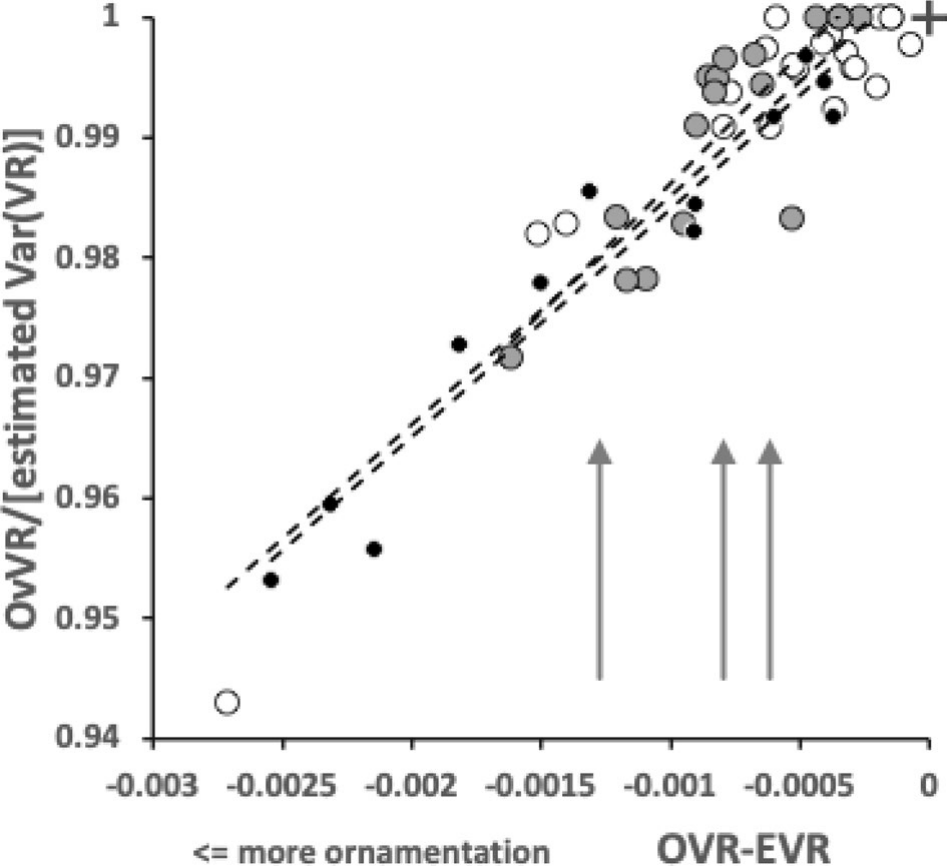
Mite specimens with largest magnitude ornamentation measure ($x\text{-axis}$ in negative direction) also shows less ergodic profile of velocity ratio *VR* values (*y*-axis). Lower *y* values indicate a more ‘spikily’ differentiated velocity ratio (*VR*) profile of the mastication surface. Open circles = *Carpoglyphus lactis*. Grey circles = *Tyrophagus putrescentiae*. Black dots = *Glycyphagus domesticus*. All linear regression dashed line fits for each species in clear agreement with each other. Grey arrows are mean *x* for each species: Right = *C. lactis*, Middle = *T. putrescentiae*, Left = *G. domesticus*, showing comparative progression of moveable digit design away from a simple beam-like form (marked as a cross)

This suggests that the astigmatid moveable digit is visibly ornamented for a different reason than just offering a variety of velocity ratio values and thus ‘crunch force’ (*F*2) locations. Indeed mites with the lowest $OVR{-}EVR$ i.e., the most ornamented moveable digit ones have the lowest $\frac{OV[VR]}{\widehat{Var[VR]}}$ because by their very nature such features have similar (i.e., correlated) *VR* values and so reduce the basal ergodic degree of *VR* spikiness (Fig. 20). Think of a run of random numbers, if you change some of the (originally different) numbers to be the same as some of the others nearby in the run, the variability of the set goes down as it becomes differentiated. Ornamentation in mite digits naturally begets a focus of trophic function to different parts of the moveable digit mastication surface. Indeed, the variable values for the laboratory cultured T13 and museum specimens in Table 12 suggest that for this species, altering within digit variation might be important during evolution. Fixed homologies needed as landmarks in geometric morphometrics is not supported.

## Discussion

This, as a functional ecomorphological (Feilich and López-Fernández 2019) review *per force* examines what nature sees in the phenotype determining survival i.e., ‘fitness‘ here determined by the efficiency of the design of a mite’s food-grasping tools. How things actually work in practice is crucial in posing any evolutionary argument. As Disston (1907) says “We find many good mechanics who frankly acknowledge that they never could file a saw satisfactorily; the probable reason is that they never studied the principle of the action or working of the tool.“ This discussion elaborates upon how astigmatid mite chelae work in UK beehives based upon the geometric results above and what macro-scale human tools they may approximate. Each of the chelal morphological edges has a role to play, and a change to any one of them has an effect upon the others that must be considered. The discussion focuses on what type of food material might be processed ‘best’ by each mite’s design but of course many functional groups commonly used in ecology and food-web modelling actually feed upon multiple types of food resources (Popatov et al. 2022) in the wild.

Indeed, there is a danger that acarologists might ‘shoe-horn’ mites into ideal feeding types. This can be so when mites are seen feeding upon one thing and it is concluded that they solely feed upon that all of the time. To a degree, free-living, non-parasitic mites must be opportunistic feeders whose chelicerae can serve multiple functions. For instance, phytoseiids despite being specialist predators (Bowman 2023b) also eat pollen and yeasts, mesostigmatids from the family Parasitidae eat fly larvae yet also decaying flesh, even *Tyrophagus* spp. consume nematodes (not just fungi). This is why this review seeks to map astigmatid feeding apparatus to tools as to how they might be deployed and function when an astigmatid is faced with the challenge of eating ‘something’. Specialism then becomes a modality rather than a means of locking in a particular mite to an over-specialised type. As the famous physicist Feynman (1988) wrote: “You can know the name of a bird in all the languages of the world, but when you’re finished, you’ll know absolutely nothing whatever about the bird... So let’s look at the bird and see what it’s doing—that’s what counts. I learned very early the difference between knowing the name of something and knowing something”.

As there are only two homologous landmarks (i.e., the moveable digit tip and the condyle) plus 17 semi-landmarks (whose position is arbitrary), geometric morphometrics (Bookstein 2018) although possible appears poorly defensible as an initial primary analytical approach. A large number of interpolations from sliding each landmark to fit bending energy constraints could be poorly determined given the paucity of fixed homologous landmarks. Rather, the moveable digit dentition profiles are first analysed statistically and homologous features could be proposed (e.g. tooth, gullet, blade etc.) a posteriori. These, in turn, could be then summarised by an appropriate (x, y) co-ordinate pair and could be taken to be homologous features across individuals in geometric morphometric follow-up work. Although using such teeth, pocket or blades as geometric morphometric landmarks (of relative location) in that way may not be appropriate because the results of this study show no commonalities of regional *pattern* (see $\eta _{p}$, $\eta _{v}$ and $N_{0}$ in Tables 3, 7 and 11) across species. That is any smooth transforming deformation would *per force* need discontinuous ‘crinkles’.

A common criticism of morphological studies especially those involving laboratory cultured species is that they are not typical of populations in the field. This is not so for the mites in this review. As the three species co-occur in beehives, one would expect that they would show trophic distinctions between each other to reduce competition. The three species do have different chelal designs (Tables 16, 17 and 18). Cheliceral teeth matter in UK beehive free-living astigmatids. Chelal features have a quantifiable impact upon trophic efficiency.

Care will be taken in this ensuing discussion to draw the distinction between profile characterisation with respect to *L*2*M* axis in order to define regions and growth processes perhaps acting during evolution versus tribological measures with respect to average surface in order to characterise the physical performance of the mastication surface as a composite tool. Velocity ratio is the key large scale driver.

### The mastication surface and the fungus *Bettsia*

Bowman (2023a) discusses at length the comparison of mastication surface sizes for the three UK beehive species. *C. lactis* (as a food for predatory phytoseiid mites) is reared commercially on yeast and pollen. However, there have been unpublished claims by beekeepers (masterBK Queen Bee, Feb 22, 2015, https://beekeepingforum.co.uk/threads/pollen-mites.32686/) concerning *C. lactis* that: "This microscopic pink mite doesn’t feed off the pollen but eats the fungal mycelium of *Bettsia alvei* (the fungus associated with mouldy pollen).". This mite would thus be performing a useful function within bee colonies.

*Bettsia* is a mono-typical genus in the Ascosphaeraceae family of fungi (Betts 1912). The typical sizes of its hyphae are 2–6 μm in diameter, and its thick walled chlamydospores (intended for resting in unfavourable conditions) are $8{-}6$ by 7–5 μm in size on average. Its dispersal and reproductive cysts found in early Spring/May are sub-globose, 30 μm in diameter and contain numerous spores of average 4.3 μm in diameter. Accordingly, chlamydospores could then be grasped by all three UK beehive mite species however, the cysts are too large (being even bigger than the effective gape of *C. lactis*). Interestingly, the span of the three posterior teeth in *C. lactis* at 4–6 μm (Fig. 11) is just right to grasp the biggest mycelium, and its gullets just right to grasp the smallest mycelial hyphae.

This fungal family also contains *Ascosphaera apis* which causes chalk-brood disease in honey bees. This commonly-looked for syndrome by UK beekeepers, rarely kills infected colonies but can weaken them rendering them susceptible to other pests and diseases and reduced honey yields. Mature spores are oval ($2 \times 1.2$ μm) and tightly packed inside spherical spore balls 8–16 μm in diameter Li et al. (2018). More than ten of these in turn are wrapped into a spherical, nearly hyaline spore cyst 50–60 μm in diameter. Although the spore balls could be grasped by the mites in the hive if they come across them, the latter cyst appears to be far too large to chew. Furthermore it is not clear what the typical cell sizes of the mycelium of this pathogenic fungus are (as it infiltrates throughout the bee larva) so as to infer whether any of the three astigmatids studied herein might also be able to chew them. Certainly exploiting high protein dead honey-bee larvae would be consilient with the probably necrophagic origin of these free-living astigmatids (Bowman 2021c). Mycological follow-up is needed.

### Differential sclerotisation in the chela

The full mechanical design space for cuticular-derived structures in chelicerates is outlined in Politi et al. (2021). Assuming a composition of uniform density, Fig. 2 of Bowman (2023a) shows how the depth of the moveable digit under any tooth or pocket seen laterally closely matches the scale of food crushing forces theoretically applied at that point. This assumed stiffness being an advantage so as to resist any bending (as in bone-cracking hyaenid jaws, Ferretti 2007). Despite arthropod cuticle having a tensile strength in excess of aluminium and of a scale similar to that of bone (Holwill and Silvester 1973), an astigmatid chela could suffer two particular failure modes on its teeth crushing any food material between the two opposing cheliceral digits.

Firstly, the adductive force continually pulling upon the adductive tendon (*F*1) is such that its action could dislocate the condylar joint by the moveable digit moving backwards into the shaft when the chela is fully closed on foodstuffs (Holwill and Silvester 1973). Condylar strengthening could prevent this as well as could cope with any large induced forces downwards upon mastication (Smith 1978). A tight condylar pivot also facilitates any cutting action of the chela, after all scissors with a loose rivet will not cut well (Smith and Savage 1959).

**Fig. 21:**
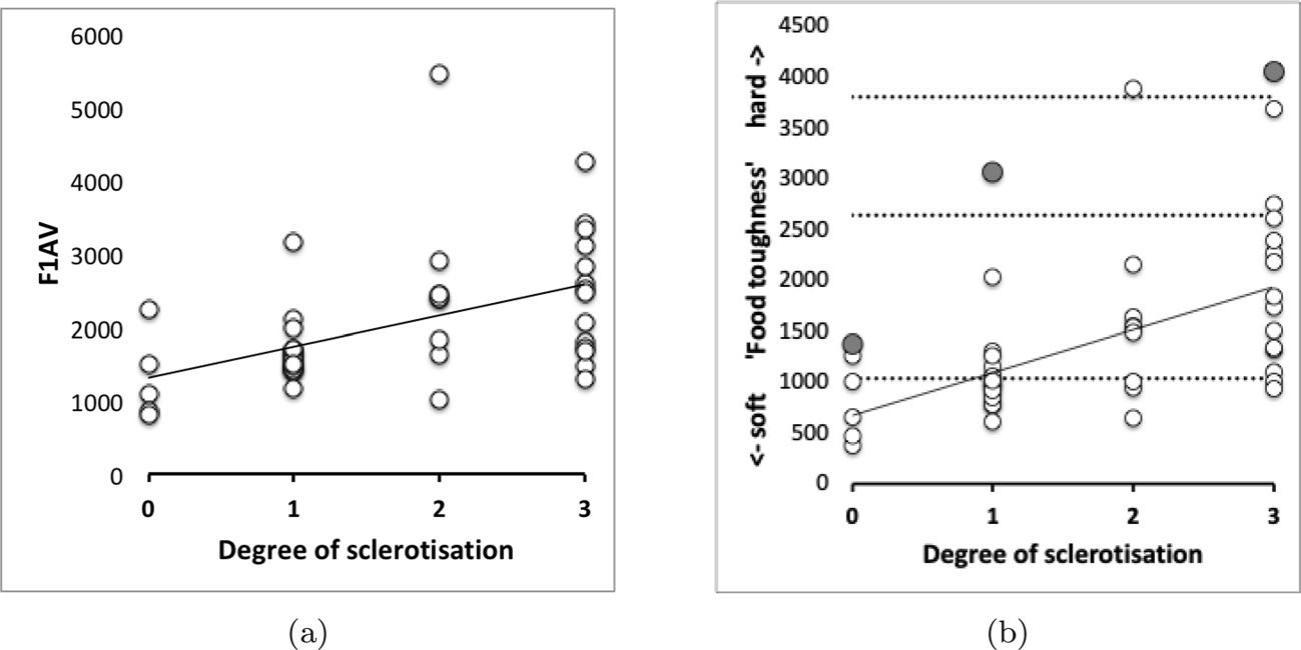
Strengthening in astigmatid chelae in 47 taxa studied by Bowman (2021c) (0 = not or feebly sclerotised, 1 = pale but sclerotised, 2 = brown moderately well sclerotised, 3 = dark brown heavily sclerotised). Individual taxa plotted as open circles. **a** Degree of chelal condylar sclerotisation versus adductive tendon force (*F*1) with indicative linear trend added. **b** Degree of moveable digit sclerotisation versus surrogate of food toughness ($VR \cdot PHI^{2} \approx F2$) with indicative linear trend. Horizontal dotted lines denote morphological feeding type boundaries (see Fig. 27 in Bowman 2021c): $y\le 1039$ are hypocarnivores, $1039< y \le 2643$ are microsaprophages, $2643< y \le 3807$ are macrosaprophages). Grey circles = UK beehive species averages left to right *Carpoglyphus lactis*, *Tyrophagus putrescentiae*, *Glycyphagus domesticus*

Figure 21a shows that the visual degree of sclerotisation of the condylar joint to be associated with a greater adductive force on the closing tendon (for the 47 free-living astigmatid species studied by Bowman 2021c). This latter force must be resisted by the condylar apparatus. Similarly any pressure back from say grabbed struggling nematodes against individual teeth by an omnivorous astigmatid induces a forward reaction at the rotation point (Fig. 2a in Smith and Savage 1959) which must also be resisted at the articulation point.

Tables 4, 8 and 12 show *F*1 values for the three species from UK beehives. Bowman (2023a) gives their corresponding chelal sclerotisation score averages. The average (and sd) $VR \cdot PHI^2={\hat{F2}}$ values following Bowman (2021b) for the three species in this beehive study were: *C. lactis* 1375.9 (340.74), *G. domesticus* 4051.9 (637.68), *T. putrescentiae* 3066.8 (817.50). Noting the raised position of these averages on Fig. 21b, this suggests that the wild-collected carpoglyphid and acarid would be classed as microsaprophages and the glycyphagid as more than a macrosaprophage i.e., like a secondary decomposer oribatid. Laboratory cultured exemplars may have been selected for a degree of trophic feebleness.

Secondly, as the chela closes against hard foodstuffs to crush them, tensive, compressive and shearing stresses (Frost 1971) will be set up in the fabric of the moveable digit as the food material resists crushing (as, “For every action, there is an equal and opposite reaction“ Isaac Newton). This could produce compressive, tensive, and torsional stresses, reversible elastic bending and shearing strains, or irreversible plastic deformations, or even breakages in the acarine moveable digit itself.

Note that for a slender narrow beam like the plesiomorphic moveable digit, bending and stretching is easier than elongation. As end-on forces are seemingly not generated on chelal closure (but would be in cheliceral protrusion into foodstuffs), ‘buckling‘ (Lautrap 2011) is not explicitly considered here. Figure 2 of Bowman (2023a) shows how the lower profile of the moveable digit almost matches the reflection of the scale of *F*2 adductive forces during mastication.

**Fig. 22:**
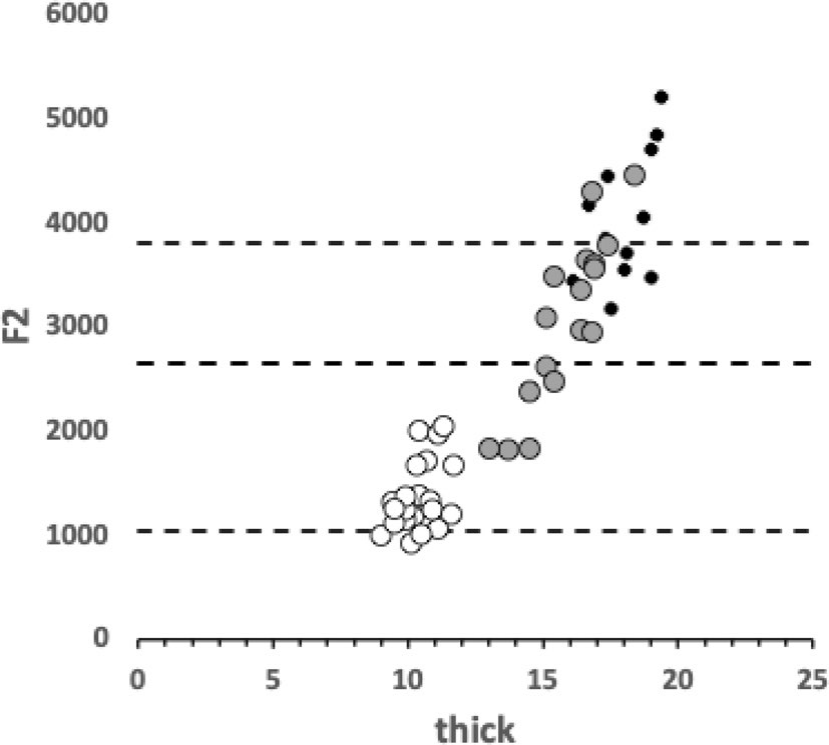
Moveable digit thickening and digit tip crunch force $F2=VR.PHI^2$ show a strong relationship consilient with degree of sclerotisation in wild-collected mites from UK beehive. Sclerotisation score *C. lactis*=0, *T. putrescentiae*=1, *G. domesticus*=3. Boundaries indicate functional group change, see Fig. 21b

As in those vertebrate jaws with dominant temporalis musculature, one expects the moveable digit to be mainly under tension roughly from the moveable digit tip to the top of the ’coronoid’, so one expects any thickening to be between these points, that is along the base of the teeth and the ‘anterior (ascending) ramus’ of the coronoid Smith and Savage (1959). This is exactly where the gleaming actinochitinous nature of the moveable digit is found (Fig. 2 upper). Indeed comparing estimates of moveable digit thickness to digit tip crunch force *F*2 (Fig. 22) shows a strong positive relationship. Matching fixed digit strengthening is probably the origin of the more bulbous chelal shape distally in the larger upper convex hull group of species in Fig. 25c in Bowman (2021c), stylised in Fig. 20 therein.

Fixed digit teeth occluding against the moveable digit also increases the chance of local failure. This, roughly vertical force applied to the moveable digit teeth on food crushing, induces the danger of the condyle being driven downwards and backwards out of its socket (e.g., Fig. 3a in Smith and Savage 1959). Figure 21b shows that the visual degree of sclerotisation of the moveable digit is associated with a greater toughness in food material grasped.

The boundary at ‘food toughness’ of just over 1000 is midway between the values of fragmentary and microphytophagous oribatids (Bowman 2021c). The boundary at ‘food toughness’ just over 2500 is midway between the values of microphytophagous and macrophytophagous oribatids (Bowman 2021c). The boundary just below 4000 in ‘food toughness’ is midway between those values of macrophytophagous oribatids and those scored as non-specialised or panphytophages by Schuster (1956). Only those mites $>\,4000$ on the *y*-axis are likely to contain primary decomposers (Bowman 2021c). In mites there is no analogue of the masseter musculature or sliding articulations found in vertebrate jaws which facilitate herbivory. In that way there must be a limit to the degree of strengthening the astigmatid mite’s chela can undergo given its mechanical design is essentially that of a carnivore, and thus there will be a limit to the size of hard food that can be triturated (and thus so by even the largest most powerful oribatid).

The moment of inertia (*I*) of the moveable digit (treating it like a thin rod of homogeneous material rotating at one fixed end) is:

$$\begin{aligned} I=\frac{1}{3}\cdot M \cdot (L2M)^2 \end{aligned}$$

where *M* is the mass of the moveable digit (Ohanian 1989). Torque (U), the force generating any rotation perpendicular to the long axis of the moveable digit is

$$\begin{aligned} U = L2M \cdot F2 \cdot sin\left( \frac{\pi }{2}\right) =L2M \cdot F2 \end{aligned}$$

where *F*2 is the perpendicular crunch force generated by the tip of the moveable digit.

Newton’s Second Law of Motion is that $F=m\cdot a$, so analogously $U=I \cdot {\tilde{a}}$ where ${\tilde{a}}$, is the angular acceleration produced during each prompt chelal closure. Substituting and rearranging means:

$$\begin{aligned} {\tilde{a}}=3 \cdot \frac{F2}{M \cdot L2M}=3 \cdot \frac{F1 \cdot VR}{M \cdot L2M}=3 \cdot \frac{F1 \cdot L1U}{M \cdot {L2M}^2} \end{aligned}$$

For an astigmatid, sclerotisation is liable to increase *M* thus reducing the angular acceleration of the moveable digit. This can only be offset by either increasing the size of the input lever moment arm (*L*1*U*, which for fixed *L*2*M* effectively means a change in chelal design), and/or by increasing the magnitude of the muscular pulling force on the adductive tendon (*F*1), and/or by a reduction in the output lever arm (*L*2*M*, which again infers a design change). Adjustments in the latter for fixed *L*1*U* produces less than proportionate gains. For a fixed *F*1, elongation of the moveable digit can only preserve angular acceleration if the mass of the digit is decreased, and/or the velocity ratio increases.

Using the average *L*2*M* and crunch force (*F*2) figures for free-living astigmatids from Bowman (2021c) and regarding the mass of the moveable digit of *C. lactis* to be 1 unit, this all infers that to retain the same specified angular acceleration then the mass of the moveable digit for *T. putrescentiae* would have to be $\tfrac{594.26}{23.8}\cdot \tfrac{25.3}{302.25}=2.09$ units, and that for *G. domesticus* would have to be $\tfrac{1094.29}{28.8}\cdot \tfrac{25.3}{302.25}=3.18$ units. As the length of the moveable digit in *T. putrescentiae* is 6% smaller than that for *C. lactis*, its idiosomal index is smaller than the carpoglyphid, and Table 15 only indicates a mild change in sclerotisation scores between them, this calculated mass change is considered unlikely. Rather, it is expected that the angular acceleration is different comparatively between the two species (it being probably higher in *T. putrescentiae*). Turning to *G. domesticus*, the length of the moveable digit is 14% larger than that in *C. lactis*, and although the idiosomal index value is smaller (being akin to that of the acarid), Table 15 indicates a strong change in sclerotisation scores between them. This calculated mass change is then considered much more likely. Moveable digit angular acceleration (for the digit considered as a rod) may be conserved between *C. lactis* and *G. domesticus*.

If one considers the moveable digit not just like a slender spiky rod (with teeth) but perhaps of a ‘slab and spike‘ shape, then it is trivial to minimise the overall rotational moment of inertia for the moveable digit by concentrating its mass mainly around the condylar rotation point. Such would engender the classic shape of acarine chelae, whereby the slender spike-like base-plate of the moveable digit mastication surface gives way to an ascending ramus and a coronoid-like process posteriorly. This would much like the large hips in vertebrates and large coxae in arthropods and the fact that leg appendages get progressively thinner distally. Mite designs cannot beat the constraints of physics.

**Fig. 23:**
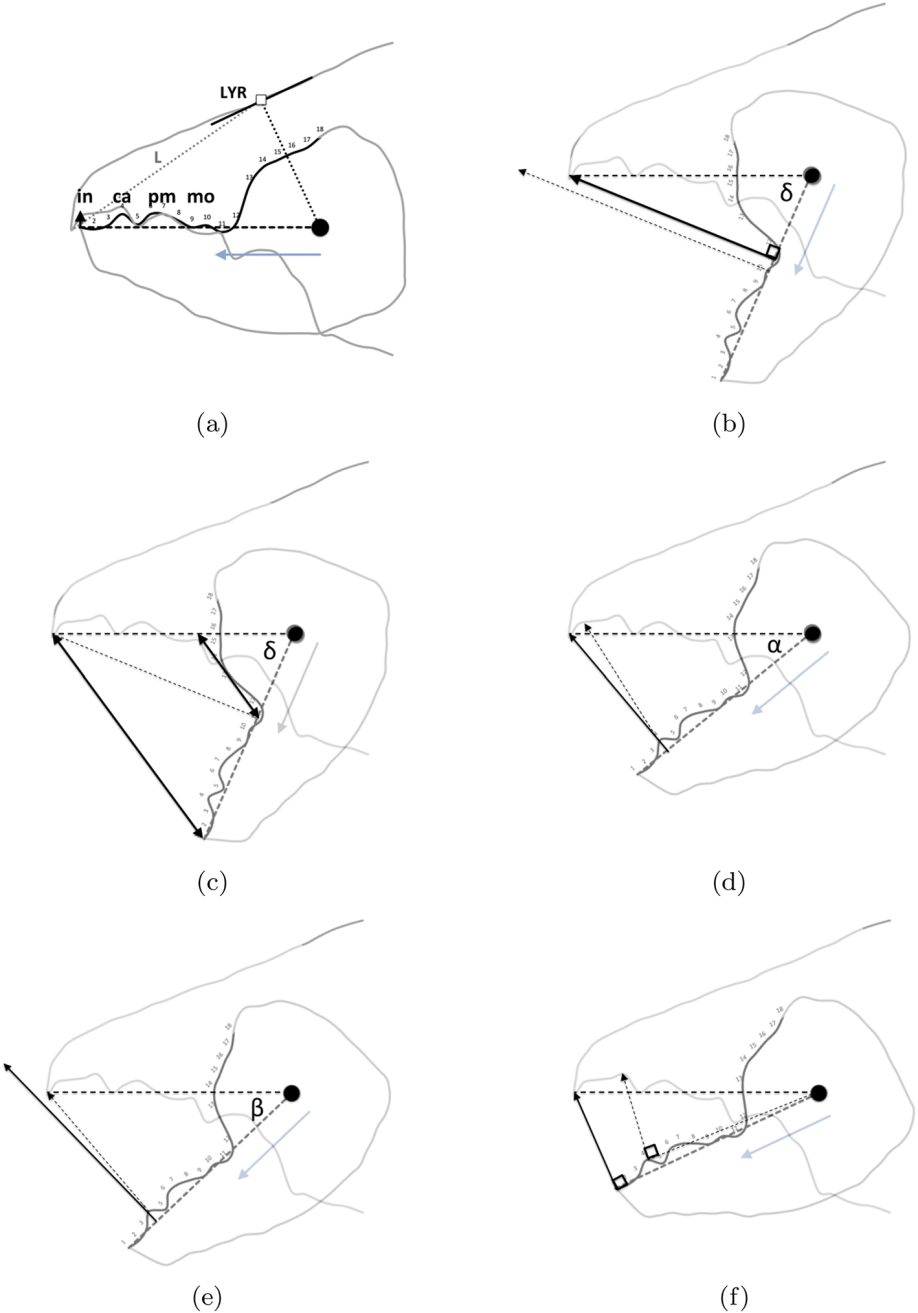
Dentition affects gape (female *Tyrolichus casei* (Oudemans) chelicera for clarity). Black circle = condyle. *L*2*M* axis dashed line. Semi-landmarks highlighted by grey numbers. Grey arrow is *L*1*U* distance from condyle. Mastication surface ends at semi-landmark 11. **a** Closed chela—all digit features (analogous to vertebrate in = incisors, ca = canine, pm = premolars, mo = molars) engaged as foodstuff completely crushed by adductive force. Open square (LYR) is fixed digit lyrifissure here angle of $65^{\circ }$ to *L*2*M* axis shown by dotted line that then runs onto fixed digit tip along line *L*. Solid black line is orthogonal (i.e., tangential) at position of LYR. **b** Point of maximum effective gape (‘span‘ of moveable digit). Note that although the adductive muscle tendon (inserting just posterior of semi-landmark 18 and pulling approximately parallel to *L*2*M* axis—see Fig. 11 top left) would have to flex around it, the coronoid process does not impinge internally upon the dorsum of the chelal shaft. Angle given by $\delta$. Any force at the end of moveable digit mastication surface (solid arrow here at semi-landmark $i_{e}=11$ on *L*2*M* axis) is resisted by fixed digit tip. Forces for moveable digit features distal of this indicated by dotted arrow (with no opposition from the fixed digit). **c** Maximum $\left(=2\cdot L2M\cdot \sin\left(\frac{\delta }{2}\right)\right)$ and minimum $\left(\approx 2\cdot L1U\cdot \sin\left(\frac{\delta }{2}\right)\right)$ grip-able fragment or morsel depth when chela at maximum effective gape indicated by solid double headed arrows. For exact minimum use $(L2M-x_{i_{e}})$ rather than *L*1*U*. Note minimum grazes leading edge of coronoid process. Sub figure from Bowman (2023a). **d** Elevated features on the moveable digit ensure engagement of crushing force with a thicker part of the opposing fixed digit (dashed arrow). Solid arrow assume simple bar-like beam. Note fixed digit tip elongation (‘overbite‘) therefore increases moveable digit feature engagement with a potentially thicker and thicker opposing structures. **e** Elevated features on the moveable digit allow a larger gape angle than when assuming a bar-like beam mechanics for the moveable digit action ($\beta >\alpha$). The larger the moveable digit distal tooth the bigger this effect. Fixed digit tip elongation (‘overbite‘) synergises this. Backward curving moveable digit teeth (as in snakes) exacerbate this effect. Actual gape reduced by height of distal features. **f** Gape angle when moveable digit tip force is opposed by fixed digit tip. Height of moveable digit teeth posterior of $x_{4}$ approximate the dashed hypotenuse $hyp_{4}$ at that $x_{i}$ location showing no ‘breasting’

There still remains the issue of the form of the ascending ramus. Figure 23 illustrates essentially a circular profile to the coronoid process around the condyle with a dorsal bulge for the adductive tendon attachment. Could this be a different way of decomposing digit morphological development during evolution (i.e., a basal ramus plus augmentation, and a separate horizontal $\rightarrow$ ascending ramus process, Fig. 5)?

### How might the fixed digit dorsal lyrifissure be related to effective gape?

Bowman (2021c) states that taking *L*2*M* to be equivalent to the real gape (i.e., the maximum diameter of a food morsel that can be gripped) assumes a maximum opening angle of around $60^{\circ }$ for the chela in practice. Figure 23b shows that for a mite where the moveable digit tip engages with the fixed digit tip and the end of the mastication surface is *L*1*U* units from the condyle along the *L*2*M* axis, then $L2M\cdot \cos (\delta )=L1U$ or the gape angle

$$\begin{aligned} \delta =cos^{-1}\left( \frac{L1U}{L2M}\right) =cos^{-1}(VR_{tip}) \end{aligned}$$

Calculating the isocelean angle $\delta$ from the average values given in Table 2 of Bowman 2021c, gives in Table 19, a range of $44^{\circ }$ (D5 = *Dermatophagoides microceras*) to $69^{\circ }$ (CA4 = *Carpoglyphus lactis*) with an average over the 47 taxa as $61^{\circ }$ (validating an almost equilateral triangle assumption overall).

For the beehive specimens assayed herein where the moveable digit tip engages with the fixed digit tip but the length of the mastication surface is not necessarily $[L2M-L1]$ as Bowman (2023a) assumed, $\delta$ needs recalculating as $\delta ^{\ast}$. Then the actual maximal effective gape *G* (ignoring digit dentition) for any mite at the end of the moveable digit mastication surface is given by

$$\begin{aligned} G=L2M\cdot \sin(\delta ^{\ast}) \end{aligned}$$

where

$$\begin{aligned} \delta ^{\ast}=\cos ^{-1}\left( \frac{L2M-x_{i_{e}}}{L2M}\right) \end{aligned}$$

These ‘span of the moveable digit’ and $\delta$ values for the wild-collected specimens (used by Bowman 2023a as well but therein labelled as $\delta$) are shown in Tables 3, 7 and 11. The average effective gape (*G*) is always less than the gape estimated using the assumption in Bowman (2021c). Angle $\delta ^{\ast}$ averages for the wild-collected samples of all three species match well those $\delta$ values for the same taxa from Bowman (2021c) in Table 19.

‘Overbite‘ i.e., the fixed digit tip overhanging the moveable digit tip on chelal occlusion found in some phytoseiids (Adar et al. 2012) as well as in some uropodoids), therefore increases maximum effective gape. ‘Underbite‘ i.e., the converse, the opposite. One example of chelicerate chelal underbite is *Neocarus* sp. Vázquez and Klompen (2015). Underbite could allow the moveable digit to be used like a plough or like pachyderm tusks to scour into a substrate. Could the moveable digit of *C. lactis* be used like this to plough through and thus ‘fish’ in fluids? The underbite in the schizogyniid *Terrogynium* (Seeman 2022) certainly may allow the moveable digit to lever up beneath the sclerites of their millipede host—to lift, puncture and saw into held material.

Interestingly $\delta$ (on average) is almost exactly the same as the angle (on average) that a line drawn from the condyle to the position of the lyrifissure in the dorsal surface of the fixed digit surface makes to the *L*2*M* axis (in a 2D plot—see Fig. 23a and the overall average values of $61^{\circ }$ versus $57^{\circ }$ respectively in Table 19). This suggests that the putative fixed digit cuticle strain receptor is actuated, to a greater or lesser extent, when any food material is actually being gripped by the mastication surface somewhere (as if the moveable digit opens wider than *G*, there is no pressure being applied to the fixed digit tip by any masticatorily active part of the moveable digit). As the moveable digit closes, more and more of the mastication surface is recruited to push the food morsel against the ventral side of the fixed digit and flex the fixed digit tip upwards, up to a maximum when the chela is fully closed.

The fact that the angles match, validates the assumption that the lyrifissure is a ‘strain gauge’ proprioceptor of chelal use in feeding. This means that as the moveable digit elongates during astigmatid evolution the lyrifissure should move posterior relative to the digit tip (as Akimov and Gaichenko 1976 illustrates for *C. lactis* where it is approximately 68$^{\circ }$), and conversely as the chela becomes ‘stubbier‘ the lyrifissure should move more anteriorly. This is consilient with the morphometric result in Bowman (2021c). For more information on chelicerate strain gauges and other proprioreceptors see Politi et al. (2021).

Further, Fig. 23a shows that there is a dorsal clearance above the moveable digit ‘coronoid process‘ (where the adductive tendon joins) of about 40% of *L*1*U* in *Tyrolichus casei* i.e., the height here is $\approx 1.4\cdot L1U$. A simple stylised triangular shape for the fixed digit is therefore that the slope of its chitinous dorsal surface with respect to the moveable digit *L*2*M* axis (given the digit tips meet at [0, 0]) would be of $slope=\frac{1.4 \cdot L1U}{L2M}$. As the moveable digit opens to maximum effective gape (*G*), the position where the adductive tendon attaches runs along an arc centred on the condyle of radius *L*1*U*. Figure 23b shows that this smooth running with no internal impediment is possible at maximum gape for *Tyrolichus casei*. A follow-up study could examine if this is always possible for all free-living astigmatid cheliceral chelae as they become ‘stubbier‘?

Given that $\delta \approx lyrifissure\ angle$, then this constraint becomes: Is

$$\begin{aligned}L1U\cdot \text{sin(lyrifissure\,\, angle})< \text{slope} \cdot (L2M-(L1U \cdot \text{cos)}(lyrifissure\,\, angle) \end{aligned}$$

or not? Using the figures for the taxa in Table 2 of Bowman (2021c), it is.

However, at only 30% ‘*L*1*U* headroom‘, D5 (*Dermatophagoides microceras*) would becomes false—the basal coronoid process would not fit internal to the cheliceral shaft at this mite’s maximum effective chelal gape. By 20%, this taxon would be joined by CH1 (*Chortoglyphus arcuatus*) and LA1 (*Neosuidasia* sp.). Design margins are tight in astigmatids (and perhaps extreme in the uropodoids illustrated in Bowman 2021b where the ‘Rollplatte’ may be the tendon protector?).

Considering the digit tip in this argument above assumes maximum leverage at the lyrifissure ‘buckling point’, is this reasonable? Figure 23a also shows that the fixed digit dorsal surface in *Tyrolichus casei* is approximately orthogonal to the line from the condyle to the lyrifissure (only diverging from this dorsal profile near the fixed digit tip). An approximation to this i.e., a line from the lyrifissure to the fixed digit tip represents *L* the length over which any force (that is any *F*2) applied upwards at that tip caused by the occluding moveable digit would be if the fixed digit was considered to be cantilever of homogeneous material fixed to and supported by the cheliceral shaft at the lyrifissure i.e., ahead of the condyle.

The actual angle of *L* over the species in Bowman (2021c) is around $80^{\circ }$ on average (Table 19). The lyrifissure would then be the location of the resultant maximum bending moment ($F2\cdot L$) and the maximum shear for the dorsal chitinous fabric of the fixed digit. Any chitinous strengthening distally (i.e., above and around the dotted line *L* in Fig. 23a) by digit sclerotisation, as in Fig. 2 Upper, will concentrate the fabric flexing and tearing forces even more at the lyrifissure (cf. the uropodoid chela head flexing mechanism). As explained above, the maximum effective gape (*G* represents that angle at which the moveable digit mastication surface on moveable digit closing can first exert an upward force upon the fixed digit ventrally so it is unsurprising that the chela is designed such that ${lyrifissure\,\, angle} \approx \delta$ for all the taxa. This explains the morphometric results in Bowman (2021c). Note that the third angle in Fig. 23a (i.e., ‘Angle at tip‘ in Table 19) is a good summary of overall chelal chitinous elongation yet to be used by acarologists. Although some acarologists have used the curvature of the moveable digit to understand feeding adaptations (Liu et al. 2017).

Bowman (2021c) points to a shift in chelal fixed digit lyrifissure and condyle position in the smallest chelae such as *C. lactis* consilient with the shift to snake-head shaped chelae in uropodoids. However there is no sessanoid bone-like ‘Rollplatte’ structure to strengthen adductive tendons in astigmatids. Rather, the digits in *Carpoglyphus lactis* are lengthened tweezer-like without tendon strengthening. It is unlikely that this is an adaptation for efficient high speed snapping of the digit tips as, unlike say in *Veigaia* spp., the digits are not strongly sclerotised overall to cope with resultant stresses and strains. Rather this is interpreted as a ‘picking‘ adaptation suitable for processing very small items such as yeasts from liquid exudates.

### Pockets and claw-like structures

**Fig. 24:**
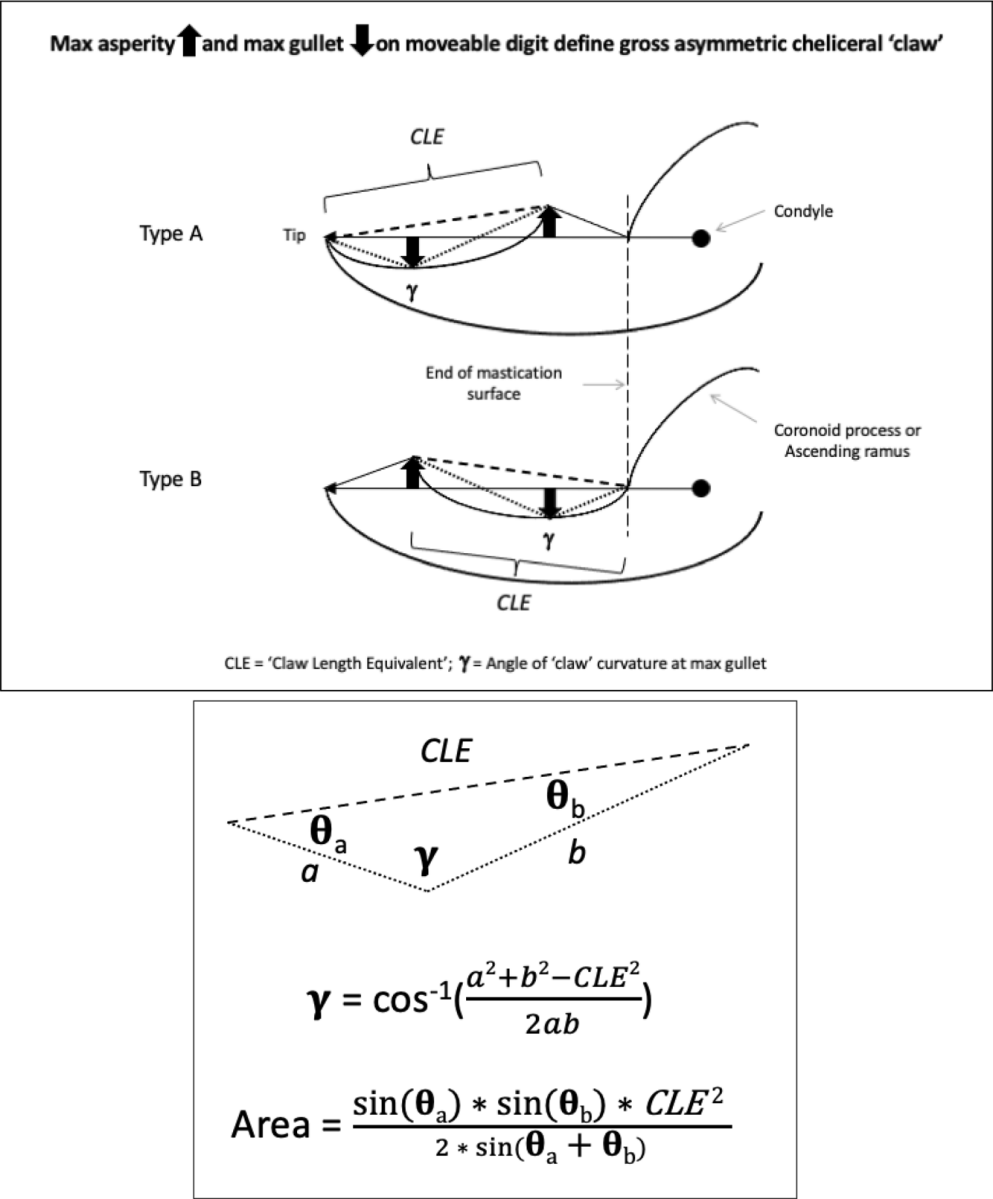
Moveable digit geometry shares similarity with mite claws. Simple gross model. Upper: ‘Pocket’ on each type of digit surface design where food material can be trapped defined by the relative position of maximum gullet and asperity. Lower: Estimated area of scalene ‘pocket’ multiplied by *thick* from Bowman (2023a) yields the approximate volume of material held within each moveable digit chelal surface

Astigmatid moveable digits can be characterised into two gross types according to where the maximum asperity and gullet is (Fig. 24 Upper).

Type A, the classic arrangement for instance of a vertebrate herbivore mandible like in squirrels, is where the distal pocket-like gullet allows plant tissue to be easily repositioned as it is chewed by the proximal teeth (Fig. 3). This is the vertebrate design when much chewing is required to break foodstuffs up. Type A is thus not just a tipped tearing design but also a masticating design. Type B is like the mandible of extinct browsing dinosaurs e.g., *Diplodocus*, who specialised (as do modern wild Exmoor ponies) in the ‘nipping’ of foliage by their distal teeth. Such ancient vertebrates then relied upon gastric processes to break up plant material after swallowing (Button and Zanno 2020) rather than further teeth-driven trituration as in modern ruminant mammals. Free-living astigmatids do show motile gastric boli comprised of ingested food fragments (work of Erban, Hubert etc., discussed in Bowman 2023a) which may act like a gastric ‘mill’. Type B does less mastication and Type B grazing will have the tendency to produce closely cropped ‘lawns’ in foliage (or indeed in fungal mycelia). In that way astigmatids may act as selective gardeners much like how marine reef fish maintain their allotments. This categorisation is consilient with the location of the highest asperity on the moveable digit surface ($x_{R_{p}}$ in Tables 5, 9 and 13).

**Table 9:** *Glycyphagus domesticus* sorted by type

Specimen	Type	CLE	*γ* (°)	Pocket *EVR*	Pocket vol.	F1	Pocket *E*[*F*2]	Slicing blade Retr.	Cutting edge Retr.	Slicing blade Protr.	Cutting edge Protr.
224(2)-1	A	13.8	150	0.549	241.4	3080.4	1691.2	4.2	–	–	2.0
224(2)-2	A	13.5	143	0.630	275.7	2596.8	1636.0	2.1	–	–	1.9
224(2)-3	A	13.2	140	0.587	254.2	2926.0	1717.5	3.9	–	–	1.9
224(2)-6	A	12.8	139	0.747	260.5	2825.5	2110.7	2.3	–	–	2.3
224(2)-9	A	04.3	104	0.473	43.6	2482.1	1174.0	13.9	–	–	1.6
224(2)-9a	A	09.4	133	0.613	170.6	2736.8	1677.7	7.7	–	–	1.5
224(2)-4	B	10.7	56	0.647	1001.5	3030.2	1960.5	–	2.3	5.6	–
224(2)-5	B	11.1	52	0.564	1056.5	2362.8	1332.6	–	2.3	5.6	–
224(2)-7	B	09.7	56	0.807	738.7	2508.7	2024.5	–	1.8	4.6	–
224(2)-8	B	09.6	50	0.773	783.3	2605.9	2014.4	–	1.5	4.9	–
224(1)-10	B	10.2	147	0.631	124.4	2202.2	1389.6	–	1.6	5.3	–
224(1)-20	B	10.4	67	0.670	568.9	2458.4	1647.1	–	1.9	3.6	–
Tear/chew	A	11.2	135	0.600	207.7	2774.6	1667.8	5.7	–	–	1.9
Nibble	B	10.3	71	0.682	712.2	2528.0	1728.1	–	1.9	4.9	–
	Overall					2651.3 (272.53)					

Specimen	Shank of hook Retr.	Total Retr.	Tear prop. Retr.	Shank of hook Protr.	Total	Tear prop. Protr.	$\tfrac{CLE}{CLI}$	$R_p[VR]$	$R_p[F2]$	$x_{R_p}$	No. teeth^a^	Pitch^a^
224(2)-1	19.8	33.5	0.41	–	–	–	0.11	0.723	2227.2	13.8	4	0.25
224(2)-2	15.3	28.7	0.47	–	–	–	0.13	0.880	2284.0	13.5	4	0.26
224(2)-3	18.8	31.9	0.41	–	–	–	0.11	0.774	2264.8	13.1	3	0.20
224(2)-6	14.4	27.0	0.47	–	–	–	0.12	1.042	2943.2	12.7	4	0.28
224(2)-9	31.5	33.5	0.06	–	–	–	0.04	0.504	1251.9	4.0	3	0.21
224(2)-9a	22.5	31.8	0.29	–	–	–	0.08	0.733	2005.9	9.3	4	0.27
224(2)-4	14.4	25.1	0.42	5.3	16.0	0.67	0.09	0.497	1507.2	5.3	4	0.25
224(2)-5	15.1	26.2	0.42	5.3	16.4	0.68	0.10	0.433	1023.6	5.3	3	0.18
224(2)-7	12.5	22.2	0.44	4.4	14.1	0.69	0.09	0.614	1539.6	4.4	3	0.21
224(2)-8	13.2	22.8	0.42	4.7	14.3	0.67	0.09	0.596	1553.9	4.7	3	0.21
224(1)-10	13.5	23.7	0.43	5.2	15.4	0.66	0.09	0.482	1062.1	5.2	3	0.19
224(1)-20	13.7	25.8	0.47	3.3	15.4	0.79	0.09	0.526	1292.3	3.3	3	0.25
Tear/chew	20.4	31.1	0.35	–	–	–	0.10	0.776	2162.9	11.0		
Nibble	13.8	24.3	0.43	4.7	15.3	0.69	0.09	0.525	1329.8	4.7		
								0.650 (0.1841)	1746.3 (592.94)	7.9 (4.23)	3.4 (0.51)	0.23 (1.205)

^a^Subjective measures. Indet. = indeterminate. – = data impossible. Summary is mean, median or geometric mean as appropriate. SD in (....)

Unlike in plant eating dinosaurs (Button and Zanno 2020), comparative elongation of the lower adductive surface whether measured by $x_{i_{e}}$ or the drape distance *m* (Tables 2, 6 and 10), is not associated with a downwards reflexed distal tip. These astigmatids do not disturb any foodstuff substrates like extinct deinotheriid mega-fauna.

**Table 10:** *Tyrophagus putrescentiae* derived measures from original data in μm

Specimen	*O*[*VR*]	$\varTheta$	$\varPhi ^{2}$	Smoothness (*s*)	Stretch (*tel*)	Flex (*f*)	Creep (*h*)	Relative elongation (*rel*)	$m_{f}$	$m_{c}$	$r_{f}$	$r_{c}$	*Potential* ‘Tooth row’ $(L2M{-}L1U)$
224(1)-2	0.712	0.666	0.0220	0.116	5.3	2.3	2.2	1.11	14.8	12.1	1.24	1.14	11.9
224(1)-3	0.680	0.672	0.0215	0.056	7.0	2.1	1.3	1.17	18.1	15.2	1.21	1.13	16.5
224(1)-4	0.621	0.661	0.0225	0.059	7.0	3.0	1.5	1.25	14.7	12.1	1.29	1.24	15.4
224(1)-5a	0.659	0.680	0.0207	0.113	8.6	3.2	2.0	1.28	16.2	13.0	1.30	1.22	16.0
224(1)-6	0.594	0.643	0.0241	0.103	7.1	3.8	1.9	1.30	14.1	11.6	1.29	1.17	15.6
224(1)-6a	0.615	0.657	0.0229	0.103	7.5	3.2	1.9	1.23	15.3	12.7	1.23	1.19	16.5
224(1)-7	0.615	0.635	0.0248	0.120	8.9	3.7	2.4	1.21	16.2	13.6	1.23	1.18	16.2
224(1)-7a	0.662	0.661	0.0225	0.027	3.9	2.0	1.0	1.11	14.3	12.5	1.18	1.16	13.8
224(1)-7b	0.640	0.669	0.0218	0.044	5.5	2.1	1.2	1.16	16.0	13.5	1.21	1.18	17.1
224(1)-7c	0.724	0.717	0.0174	0.063	4.8	2.3	1.2	1.19	13.4	11.2	1.20	1.17	12.6
224(1)-7d	0.650	0.677	0.0210	0.124	5.7	2.6	1.8	1.27	15.1	12.4	1.26	1.20	15.3
224(1)-8a	0.724	0.694	0.0195	0.061	5.0	2.3	1.4	1.15	17.6	14.3	1.21	1.14	15.4
224(1)-8b	0.585	0.655	0.0230	0.060	6.2	2.1	1.1	1.22	14.5	12.1	1.24	1.22	18.3
224(1)-10	0.690	0.678	0.0209	0.125	6.9	3.2	2.3	1.20	14.3	12.0	1.21	1.16	12.9
224(1)-10a	0.798	0.711	0.0179	0.097	6.2	3.1	2.3	1.21	17.1	14.2	1.22	1.14	12.5
224(1)-14	0.553	0.635	0.0248	0.085	7.9	2.8	1.5	1.24	15.4	12.3	1.28	1.20	19.2
224(1)-odd2	0.726	0.697	0.0192	0.069	5.9	2.6	1.8	1.19	14.9	12.8	1.21	1.18	13.0
Summary	0.662	0.671	0.0215	0.078	6.4	2.7	1.7	1.21	15.4	12.8	1.24	1.18	15.2
	(0.0628)	(0.0239)	(0.00217)	(0.0310)	(1.36)	(0.57)	(0.46)	(0.055)	(1.29)	(1.05)	(0.036)	(0.032)	(2.11)
T13 ($n=20$)	0.619	0.634	0.0224	0.087	7.8	3.0	2.0	1.19	14.1	11.9	1.22	1.18	10.6
Museum ($n=17$)	0.632	0.659	0.0218	0.079	5.5	2.6	1.7	1.19	11.8	9.9	1.25	1.22	7.0

Summary is mean, median or geometric mean as appropriate. SD in (....)

The two gross design types (A versus B) are a simplified abstraction as a part of an epistemological cascade of finer and finer more detailed models. They successfully characterise all the 12 *G. domesticus* specimens studied (although 50% were allocated to design A and 50% to B, Tables 5, 9 and 13). This apparent random preference between order of maximum asperity and maximum gullet along the moveable digit *L*2*M* axis suggests if the surface has multiple teeth that they are of similar size i.e., the surface would be saw-like.

Twelve of the 19 *T. putrescentiae* specimens were classified as design A (tearing and masticating), three as of a ’nipping’ design B and four *incertae sedis*. This suggests that the acarid, if it does have multiple teeth, may invest in a particular pattern of dentition whereby a somewhat larger asperity occurs relatively proximal to the condyle.

**Fig. 25:**
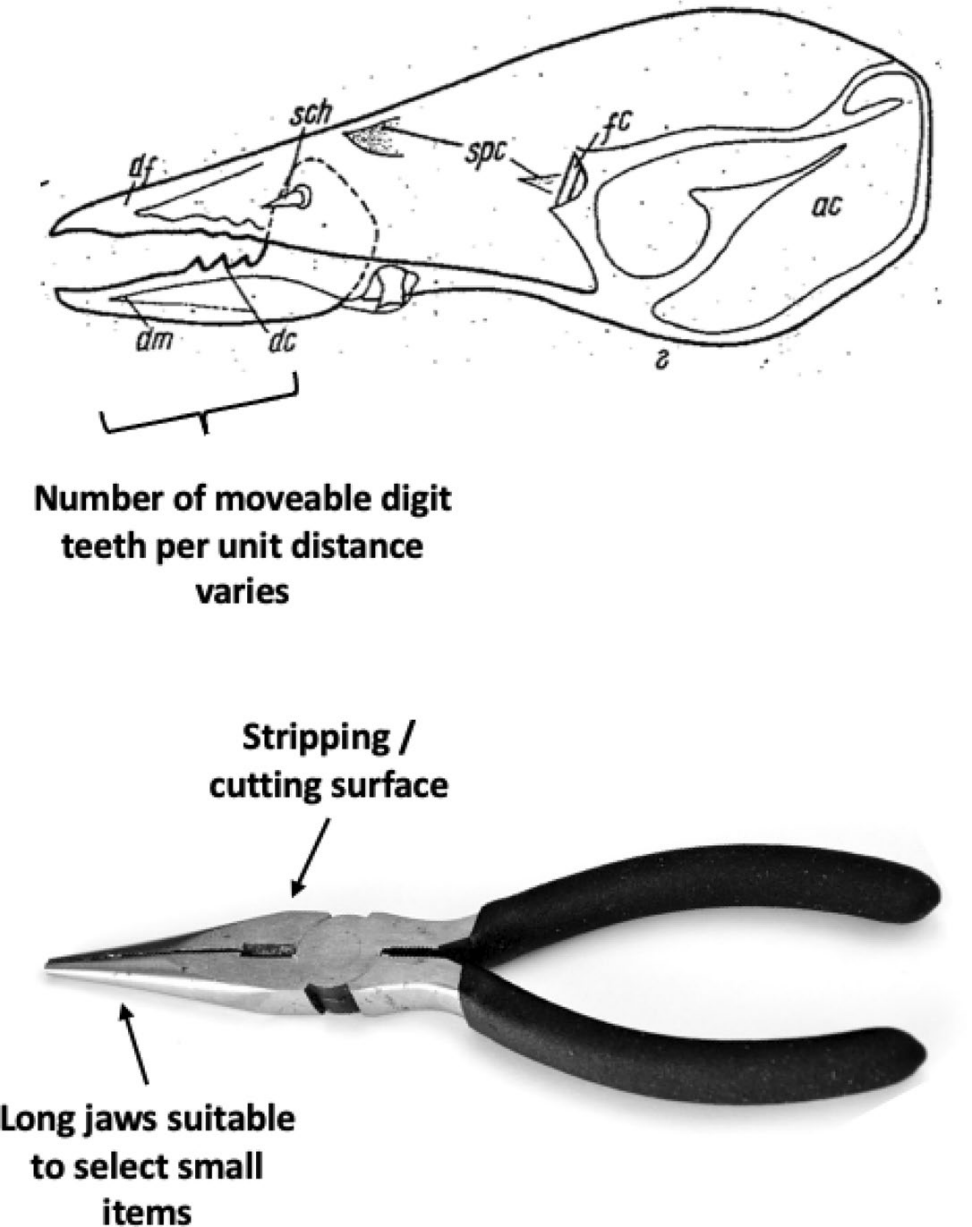
*Carpoglyphus lactis*. Upper. Variable pitch in moveable digit dentition. Small teeth unlikely to be significantly ‘set’ (see Fig. 31). Annotated including with original abbreviations © Akimov (1985) with permission. Lower. Traditional mechanical analogy of carpoglyphid cheliceral chela to needle-nose pliers (see argument in Fashing 1998). Amended from https://commons.wikimedia.org/wiki/File:Long-nosePliers.jpg © Raysonho 2015 with permission under Creative Commons CC0 1.0 licence. Fifteen of 21 wild-collected specimens examined matched this ‘picking’ form

More than 50% of the 21 specimens of *C. lactis* could not be unequivocally allocated to either gross design, only four matched type B and six type A. It seems that the carpoglyphid moveable digit is invariably very bar-like with repeated minor fluctuations in surface height rather than a clearly dentate jaw shape that can be modelled comparatively. As such it is reasonable to interpret the chela as an analogy to needle-nosed pliers (Fig. 25).This concurs with Fashing (1998)’s view of it being a collecting-picking specialist species.

Within each gross type, the maximum asperity and maximum gullet on the mite moveable digit define a gross asymmetric cheliceral ‘claw’ whose characteristics can be compared to actual mite claws Grandjean (1941). For instance Pfingstl (2017) points out that leg claw form varies rationally with habitat features in intertidal ameronothroid mites. Tables 5, 9 and 13 give claw length equivalents (*CLE*) and the angle of moveable digit ‘claw’ curvature ($\gamma$) values for the three astigmatid species. *CLE* determines the size of objects over which the ‘claw’ can hook.

For the real leg claw of a littoral oribatid, this ‘grasped diameter’ increases during development (Pfingstl and Kerschbaumer 2022) and must support a proportion of the mite’s whole body weight, so Pfingstl et al. 2020 scales it by body length. This may not be appropriate for astigmatid digits (see below). Due to their curved grapnel-like shape and high stiffness, claws promote attachment by interlocking to surface roughness Politi et al. (2021). Herein this mechanism is referred to as a meso-attachment. $\gamma$ determines the shape of objects which can be grasped. *CLE* and $\gamma$ are negatively correlated—an acute angle $\gamma$ *per force* indicates a likely small diameter of object grasping in general. A large *CLE* indicates a likely flatter grasped surface.

Over all the three species, *CLE* values for the type B design are on average shorter than for the chewing type A design, particularly so for the few *T. putrescentiae* scored with this design.

*C. lactis* indeed has the largest *CLE* values for the type A design and the largest $\gamma$ consilient with its proposed collecting/picking style of chelal action. Comparing values to Pfingstl and Kerschbaumer (2022) shows in general that the size of intertidal oribatids leg claws are two to three times that of astigmatid jaw Type A *CLE*s—only the leg claws of some oribatid species’ larvae overlap in size. Type A moveable digit astigmatids also have moveable digit $\gamma$ angles that majorly exceed the typical mangrove oribatid claw design, suggesting low efficiency for hanging onto material even of that scale of (assumed low) relative roughness. All three species $\gamma$ values on average for type A designs are well in excess of the $\gamma \approx 97^{\circ }$ mean value of adult parasitic isopod thoracic 2 dactyli (inner surface over species visually extracted from Supplemental Figure 3 of van der Wal and Haug 2020). The latter animals have to steadfastly hold on to their fish host to avoid dislodgement.

**Fig. 26:**
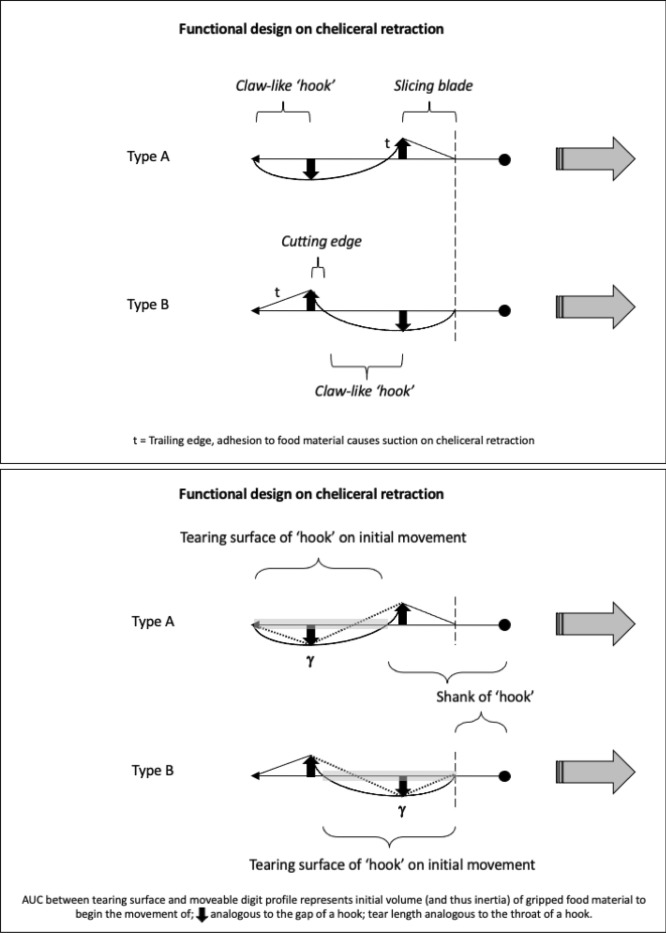
Moveable digit sawing function on cheliceral retraction. The hook-like claw is another mode for such pockets to fulfil than just grasping food against the fixed digit

**Fig. 27:**
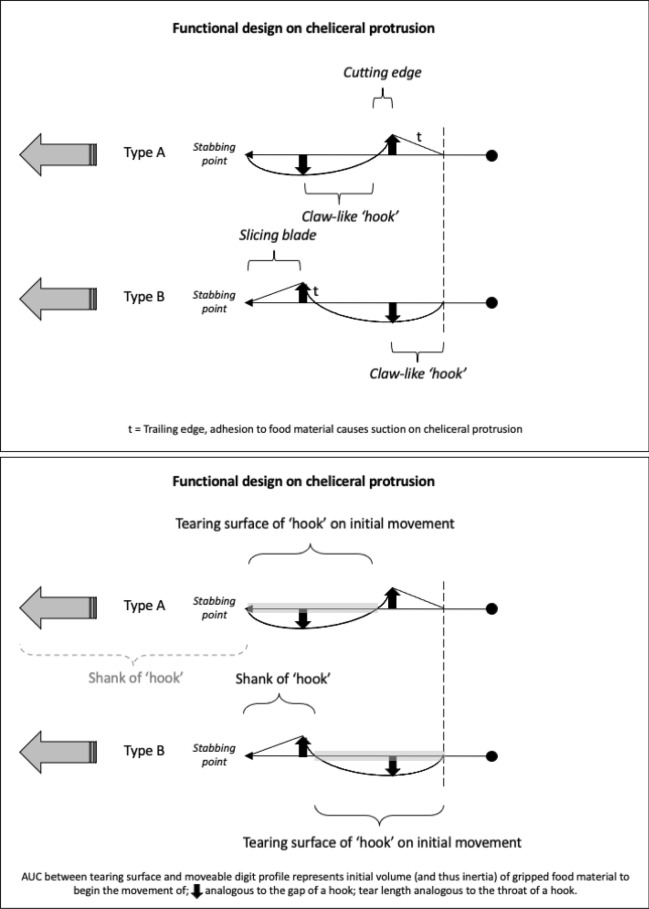
Moveable digit sawing function on cheliceral protrusion.The hook-like claw is another mode for such pockets to fulfil than just grasping food against the fixed digit

All in all then, the acarine moveable digit design A may thus function more like a simple coping saw blade where such astigmatid claw-like features do not strongly attach but will only hook into micro-irregularities in food and tear it as micro-features cut and slice material on cheliceral retraction (Fig. 26), or on protrusion (Fig. 27). Such tearing of foodstuff would facilitate trituration. A coping saw is used to cut fine, intricate cut-outs or shapes in carpentry or woodworking and is ideal for delicate applications such as curves or patterns.

Type B moveable digits have even lower *CLE* values on average and markedly narrower $\gamma$ values (often much less than the $\gamma$ values for the claws of intertidal oribatids from rocky substrates) indicative of a true micro-attachment of the digit surface to food material. The exact adhesion being dependent in detail upon the relative location of the maximum asperity and gullet. Indeed, even the moveable digit design B in *C. lactis* would be able to perform as well as a generic parasitic isopod dactylus (van der Wal and Haug 2020). The glycyphagid (at $71^{\circ }$) and in particular the acarid (at $65^{\circ }$) would exceed this performance. As these chelae are designed to hang onto material which by its cohesive nature will resist being dragged to the mites’ mouth, then perhaps scaling by *CLI* is more appropriate. Comparison to Pfingstl et al. (2020) shows general agreement of body length scaled oribatid leg claw sizes with the cheliceral length scaled astigmatid digit *CLE* values (Tables 5, 9 and 13), meaning the fundamental physics of attachment is probably similar despite the likely different actual scale of forces.

Tables 5, 9 and 13 give the hook shank size for the simple gross ‘claw’ model of the three astigmatid species’ moveable digits. A short hook shank and low $\gamma$ valued surface (e.g., *G. domesticus* type B) would function like an agricultural sickle attacking tough local patches of specific material in modest amounts. This is like a fish ‘mouth-hook’ that is designed not to be swallowed deeply but to be fairly ‘fish-friendly’ like circle hooks (Beverly 2010 which do not majorly ‘deep hook’ a fish’s throat or gut). Circle hooks do not need the angler to ‘set them’ when bitten on. but will catch on the fish lip (i.e., with only mild reeling-in of the fishing line $\equiv$ little if any closure of a chelal carrying such a pocket design). Accordingly such architectures can be easily de-hooked from their edge of mouth attachment point in fish ($\equiv$ the moveable digit grasp on foodstuff by this mite’s pocket design being straightforwardly reversible). Thus *per force* they do not penetrate thick skin, but easily slip in and slip out of surfaces. Circle-hook analogues also are much less likely to snag on anecdotal debris. The pocket *EVR* velocity ratio values in Tables 5, 9 and 13 shows that the type B designs are tuned for certain micro-attachments dependant on the species both on chelal retraction and protrusion.

In contrast, a long hook shank and high $\gamma$ surface (e.g., type A for all three species on chelal retraction and type B for *C. lactis* on chelal protrusion) would function like a long-stroke agricultural scythe sweeping through looser packed material often of larger volume. This design approximates *J* hooks used for actively catching larger fish of thicker skin. These are optimal to latch onto to all sort of types of material deep inside (although any gut hooking will also run the risk of puncturing internal fish organs). *J* hooks need ‘setting’ by the angler to catch the fish—so active chelal closure will facilitate the action of this type of Type A moveable digit pocket. As in isopods Baillie et al. 2019), reduced $\gamma$ values would indicate a steeper ‘arch’ to the hook and if the curved geometry was symmetric, would be able to resist greater forces of compression (as for instance on cheliceral protrusion into food stuff).

**Fig. 28:**
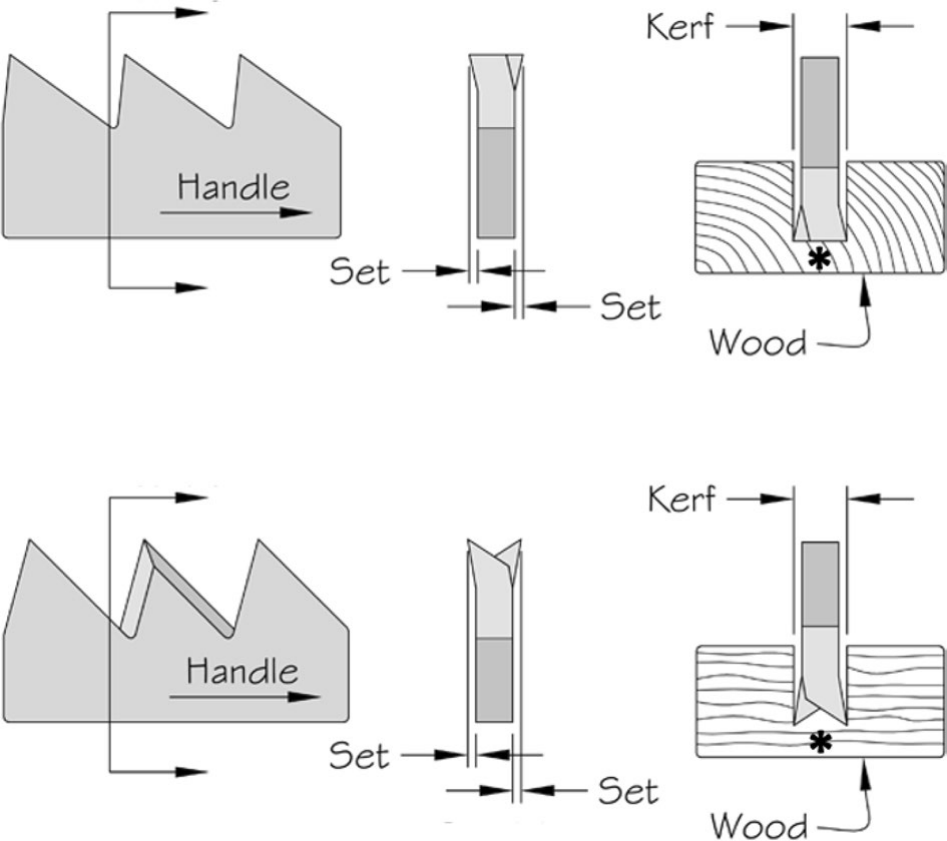
Adding set to saw teeth (i.e., a bending away of the tips from the mid-axis) increases the width of any cut (= kerf). Note different characteristic base of slot (*) depending upon if it is a rip saw with no point slope (upper) or a cross-cut saw with point slope and sloped gullets (lower). Amended from https://www.blackburntools.com/articles/saw-tooth-geometry/index.html © Isaac Smith 2012, with permission

Turning to each species. *G. domesticus* irrespective of moveable digit type shows on average the largest (rip-saw-like) ‘pocket’ volumes of the three species (Tables 5, 9, and 13). The type A tearing/chewing design acts like a long serrate carving/bread knife or bladed sword as it has on average the longest distance being retracted, the longest hook shank (on retraction) and the largest slicing blade on cheliceral retraction for that type across the three cohabiting species. In that way, it may function like the samurai sword-derived ‘Naginata’ weapon of Japanese ‘Onna-musha’ warrior nuns. Consilient with this analogy also is that the type B nibbling moveable digit design has on average the longest distance being retracted, the longest hook shank (on retraction), the largest cutting edge on cheliceral retraction and the highest tearing surface proportion on cheliceral protrusion (to clear its kerf, Fig. 28) for that type across the three cohabiting species. This marks the glycyphagid moveable digit acting rather like a rip-saw blade tearing into material analogous to wood in its properties. Saw-like features are further discussed below. *G. domesticus* really is a shredding saprophage by design.

*T. putrescentiae* exhibits medium sizes for ’pocket’ volumes (Table 13). The type A design of this species has on average the largest protrusion cutting edge. Other parameters were often on average approximately midway between those of the other two species pointing to in part the compromise portmanteau browsing/grazing design of this species (Tables 5, 9, and 13)). *T. putrescentiae* probably feeds primarily on mouldy debris and nematodes in bee nests, without causing any damage to the bees. In laboratory experiments, *T. putrescentiae* was able to feed on a wide range of food items: bee bread, pollen, beehive debris, dead brood bees, mould, honey, propolis, combs and wax (Chmielewski 1991). This species, like *C. lactis*, was able to consume royal jelly.

**Fig. 29:**
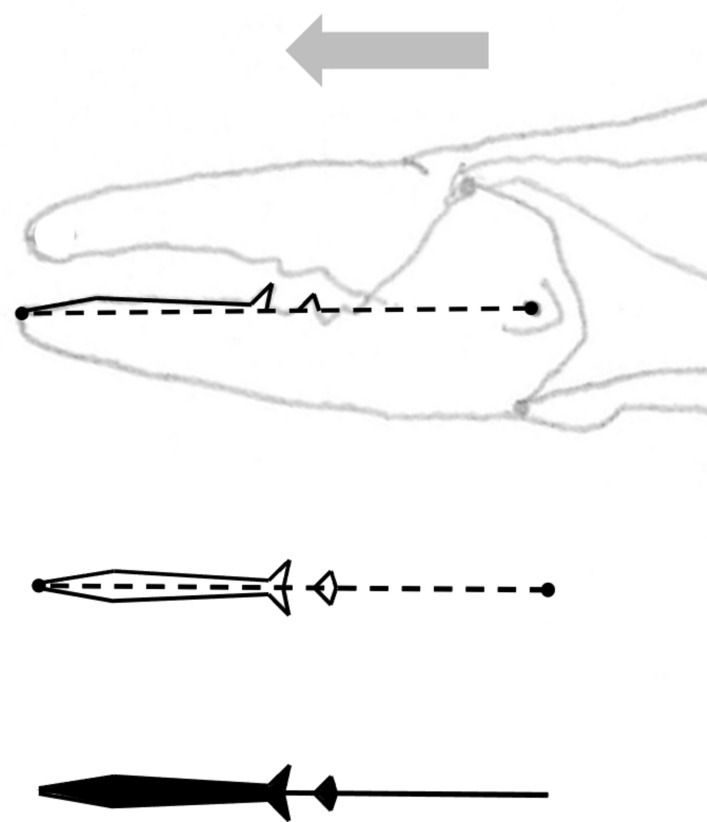
*Carpoglyphus lactis* moveable digit surface (specimen 224(1)-11).The moveable digit may act like a barbed weapon on cheliceral protrusion (grey arrow). *Upper*. Stylised surface in heavy outline. Dots at digit tip on left and articulating condyle on right. Middle. Half outline with reflected surface over dashed *L*2*M* axis. *Lower*. Volume of revolution shows overall moveable digit functional commonality with ancient barbed spears (e.g., https://www.123rf.com/photo_3303336_barbed-iron-spear-point-from-africa-isolated-on-white.html, harpoons and barbed (fishing) arrows. Six of 21 wild-collected specimens examined matched this form which clearly visualises the distal bladed nature of the digit (and hides the remaining tooth)

*C. lactis* has the smallest pocket volumes on average over the three species (Tables 5, 9, and 13) irrespective of moveable digit design type consilient with it being bar-like in form. This species has the highest proportion of tearing surface on retraction irrespective of design type. Unlike the acarid and glycyphagid, its type B micro-attachment pocket has a $\gamma$ value overlapping with the range of tarsal claws of terrestrial oribatids (Kerschbaumer and Pfingstl 2023b) suggesting that this species’ micro-attachment design better approximates almost a meso-attachment process. *C. lactis* also showed the highest total distance for protrusion, the largest shank for hook protrusion and the longest slicing blade on cheliceral protrusion consilient not with a push-pull ripsaw as in *G. domesticus* but with a long shaft spear or, a (Middle Ages historical) pike-like device, or an elongate stabbing dagger. Simple versions of these tools can often have just a modest number of teeth (Langley 2016). A follow-up SEM study of *C. lactis* needs to examine if the small teeth point upwards or backwards to crush or to catch-onto material respectively. Rotating the chela longitudinally shows that the moveable digit overall approximates half the surface of a barbed or ice spear (Fig. 29). Such elongation would need longitudinal strengthening to prevent bending and buckling. Stabbing material ‘open-mouthed’ and anchoring it from movement may release fluids and other material for the carpoglyphid to ingest. The third tooth just being another barb. Indeed, could it be that the whole carpoglyphid closed chela functions like a cocktail drink stirring stick to agitate material in semi-fluids?

Mineralisation of cuticle (as is known in other arthropods Tadayon et al. 2020) may make the digits even stiffer, so whilst strength may increase they could be more prone to fracture. Of course, a degree of condylar strengthening to prevent joint dislocation or digit breakage would be needed for any proposed stabbing action of the moveable digit (*C. lactis*, Fig. 29).

To what extent is food procurement in this species just passive pharyngeal driven drinking with only occasional cracking of small items by the three rear teeth? For sure, the large body size of *C. lactis* confers the advantage of an increased intake rate (of dilute material). Further when the chela is open, even though fairly narrow in width, could the carpoglyphid moveable digit be used as a ‘flat-spear-tusk’ like in the jaw of the extinct *Platybeledon* to shovel up aquatic and semi-aquatic material Lambert (1992)? In that way is *C. lactis* more of a fluid feeder with less dependence upon any gut processing? It certainly is the most hypocarnivorous (as judged by speed of chelal closing/food toughness plots in Bowman 2021b, c). More detailed micro-examination of carpoglyphid gut contents would be useful and the finding of any gut histological details or transit timings more like mesostigmatids (Bowman 2019) than cryptostigmatids would support this.

These pocket volumes are markedly less than the pre-ingestion morsel size ($TMv_{G}$) estimated as 4031 μm^3^ in *C. lactis*, 5228 μm^3^ in *G. domesticus* and 4246 μm^3^ in *T. putrescentiae* in Bowman (2023a). However, they are still markedly larger than the tiny food fragments which are much much smaller than the gastric bolus within the caecal lumen observed in astigmatids by Hubert et al. 2014. These fragments are approximately 0.5–2 μm in diameter i.e., around < 5 μm$^3$ in volume. Rather, if these pocket volumes were considered spherical, they would map to a range of radii: $\root 3 \of {105.7}{-}\root 3 \of {123.8}$ for *C. lactis*, $\root 3 \of {207.7}{-}\root 3 \of {712.2}$ for *G. domesticus*, and $\root 3 \of {144.9}{-}\root 3 \of {395.6}$ for *T. putrescentiae* dependent upon chelal design type. This resolves to a minimum to maximum range of 9.5–17.9 μm, nicely matching the approximate 10–15 μm magnitude of trans-neural mass oesophageal diameters (through which food must pass to get to the astigmatid gut for further processing). It seems that this mechanism is a good candidate for the main oral trituration process (sought by Bowman 2023a) whereby food material that has been grabbed, trimmed laterally and dorsoventrally is torn off in pocket-sized chunks and then directly ingested.

Material caught in the pockets, of course, can be crushed against the fixed digit surface as the chela closes. Considering the ‘pocket’ *E*[*F*2] values (calculated by $F1.(Pocket\ EVR)$) unsurprisingly shows the type B micro-attachment design having higher crunch force values than the meso-attachment type A. *C. lactis* has the lowest values for each type on average over the three species marking it out as a relatively fast closing species against any small pockets of grasped material. Whilst values are similar for the acarid and glycyphagid tearing/chewing design (approximating that of a secondary decomposer cheliceral chelal tips overall—see Fig. 27 in Bowman 2021c), the nibbling micro-attachment design for *T. putrescentiae* shows velocity ratio values comparable to the overall cheliceral chelal tip velocity ratio values of primary decomposer oribatids who specialise in attacking durable substrates. The noticeably larger chelal adductive lever input force *F*1 for *G. domesticus* individuals (Table 9) probably places them at a competitive advantage over other forms (as also found in crabs Kaiser et al. 1990). The expected pocket crunch force values *E*[*F*2] values confirm that for both design types in *G. domesticus* and the Type B micro-attachment acarid design, the scale of the occluding force at this point is similar to the moveable digit tip value of oribatids confirming these astigmatids’ ability to crunch if necessary fairly robust foodstuffs if repositioned within the chela. At around half to a third of these values, *C. lactis* again shows a weak occluding force. This is all consilient with *G. domesticus* morpho-functionally typifying the non-avian dinosaur convergent regime 2 (with possible more klinorhynchid feeding pose) and *C. lactis* more resembling in part the dinosaur convergent regime 1 (with a possible more airorhynchid feeding pose). The acarid offering a gleaning style morpho-functional way-point between them.

### Consequences of teeth or gullets

**Fig. 30:**
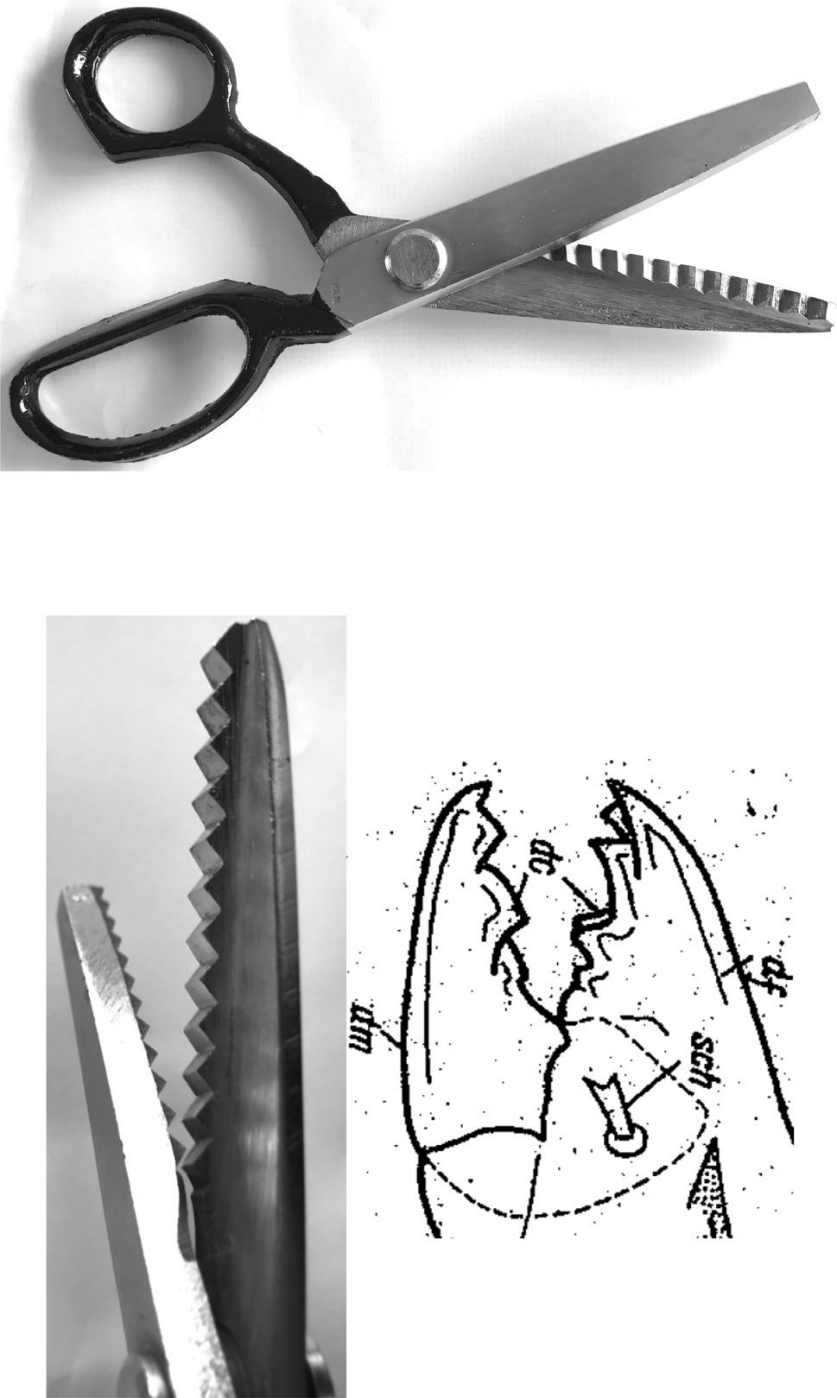
Upper. Dressmakers ‘pinking shears’ used for cutting loose weave fabric edges without fraying. Lower. Teeth on each blade interlock and slice through material on occlusion whether for shears (left) or cheliceral chela of *Tyrophagus putrescentiae* (right). Mite chelicera © Akimov (1985) with permission. dm = moveable digit. df = fixed digit. dc = chelal teeth. sch = cheliceral seta

Interlocking between teeth (and gullet) features left and right on the moveable digit in *Tyrophagus putrescentiae* and those corresponding on the fixed digit during chelal occlusion will produce the scissor-like action of ‘pinking shears’ (Fig. 30). For another example see the abaxial and adaxial surfaces of *Sancassania (= Caloglyphus) berlesei* chela shown in Figs. 2, 3, and 7 of Johnston (1965). In that way material can be sheared off during substrate browsing without necessarily majorly damaging the substrate itself.

Teeth can vary in height and gullets can vary in depth. This has consequences. For example, of course, the size of any distal tooth such as at $x_{4}$ in Fig. 23c reduces the maximum grip-able fragment (or morsel depth) $mgf_{4}$ at this location. $\delta$ is effectively changed, yielding $\zeta \zeta =\delta -sin^{-1}(\frac{y_{4}}{hyp_{4}})$ where $hyp_{4}=\sqrt{x_{4}^{2}+y_{4}^{2}}$ and the new maximum grip-able fragment (or morsel depth) $mgf_{4}=2.(L2M-x_{4}).sin(\frac{\zeta \zeta }{2})$ so that the difference $\varDelta mgf_{4}=2.(L2M-x_{4}).(sin(\frac{\delta }{2})-sin(\frac{\zeta \zeta }{2}))<0.$ A gullet at $x_{4}$ conversely increases the effective maximum grip-able fragment. Although the actual maximum grip-able fragment varies between the three species, the change in maximum grip-able fragment and the %change in maximum grip-able fragments are similar.

At semi-landmark 4, a modest 1 μm elevation of the mastication surface from the *L*2*M* axis (as in chelal claw type B) generates $\approx$ 3.9 μm reduction in gape, a 1 μm depression (as in chelal claw type A) yields a $\approx$ 3.9 μm increase in gape. This at $\approx$ 15.1% change is not insubstantial. Alternatively, at semi-landmark 8, a 1 μm elevation from the *L*2*M* axis (as in chelal claw type A) generates only $\approx$ 1.3 μm reduction in gape, a 1 μm depression (as in chelal claw type A) yields a $\approx$ 1.3 μm increase in gape. Here a lower change of $\approx$ 6.9%. Trivially (as in vertebrate jaws) tall teeth therefore must tend to occur at the front of chelae and small teeth at the rear simply due to space limitations (e.g., tall proximal teeth in *C. lactis* would prevent digit occlusion). The three teeth *have* to be small.

Yet for a notional 1 μm change less %impact occurs for gape at proximal teeth. A (relatively) large moveable digit tooth proximal to the condyle (i.e., analogous to a vertebrate ‘molar tooth‘) would only be useful to crack hard objects which were much smaller than could be maximally held between the digit tips. Is this the function of the three small posterior teeth in *C. lactis*? Do they crack open ‘swept-up’ small items collected ‘tweezer-like‘ or scythe-like by the digit tip actions?

Note that all this here does not allow for any adjustment of $\delta \rightarrow \delta ^*$ but the principles would still be the same. If one considered the moveable digit tip to be analogous to ‘incisor teeth‘ in vertebrates, the above example at $x_{4}$ gives a rationale for enlarged ‘canine teeth‘ i.e., higher, longer teeth posterior of the moveable digit (aka jaw) tip extremum but still distal from the jaw articulation (here the condyle).

**Fig. 31:**
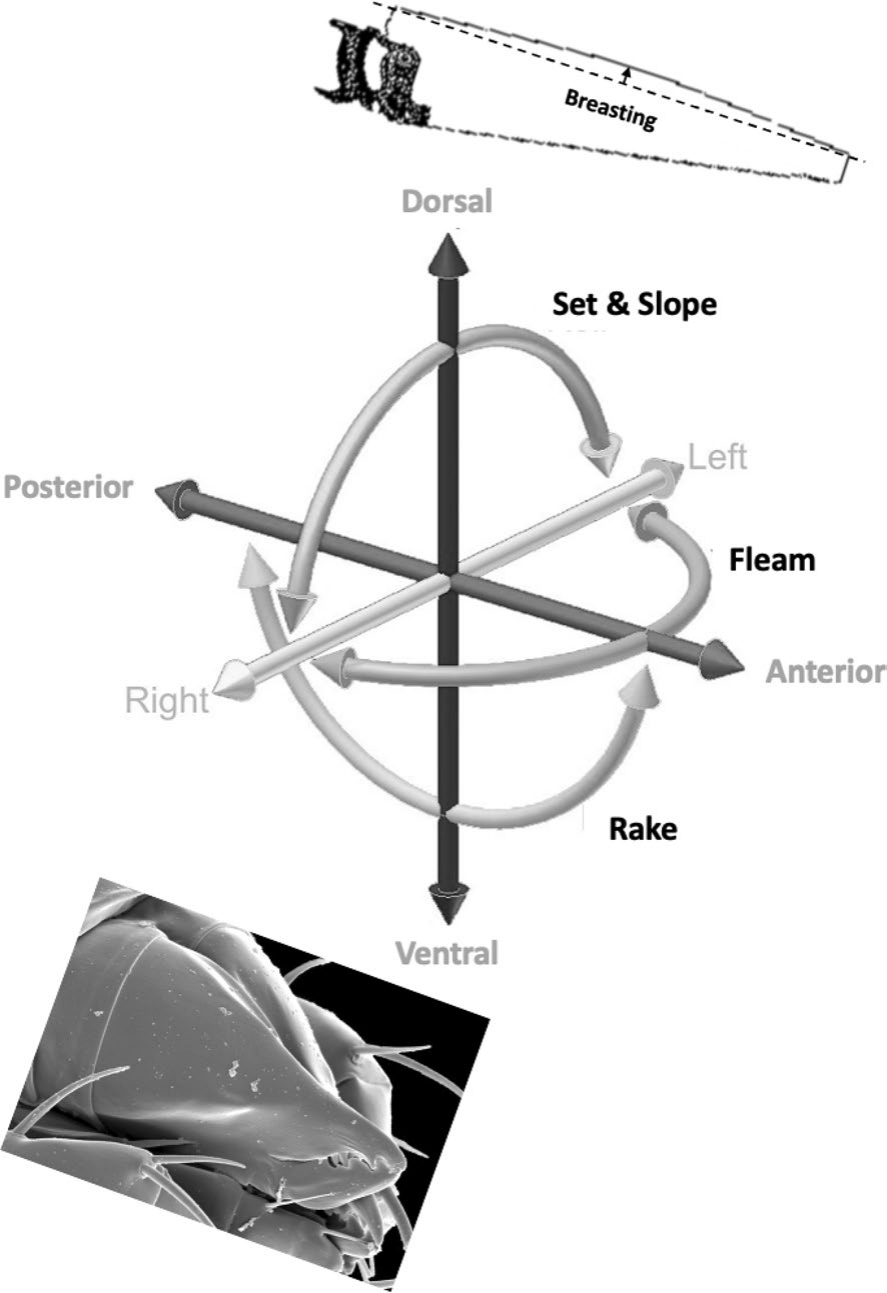
Eight degrees of freedom in astigmatid moveable digit and dentition design when considered as a saw. Central subfigure: Rake of asperities matches ‘Aerodynamic pitch’. ’Aerodynamic Roll’ matches Set for the whole tooth and matches Point slope for their tips. Adding Fleam to the points of asperities is equivalent to an ‘Aerodynamic Yaw’ action. Distance from ‘Left to Right’ is equivalent to the Base plate of teeth on a saw. Posterior to anterior distance is *L*2*M* axis for mastication surface. Amended from https://commons.wikimedia.org/wiki/File:6DOF_en.jpg © Horia Ionescu 2010 with permission. Upper subfigure: Any rise in teeth baseplate posterior $\rightarrow$ anterior causes breasting as in a saw. Amended from http://www.disstonianinstitute.com/glossary.html © Erik von Sneiden 2001–2023 with permission. Lower subfigure: SEM of astigmatid chelicera orientated similarly. From selecting ’chelate-dentate’ in https://idtools.org/id/invasive_mite/Invasive_Mite_Identification/key/0_Glossary/Mite_Glossary.htm © DE Walter 2005 with permission

The question then arises what if there are two locations for ‘distal‘ teeth on the moveable digit—say at $x_{4}$ and $x_{7}$ for this *Tyrolichus casei* example? Logic infers that the maximum gape angle will be determined by the forces at the tip of the tooth at $x_{4}$ effectively opposing the fixed digit tip, with the tooth at $x_{7}$ being forcibly embedded into the foodstuff if the second tooth is particularly taller than that one at $x_4$. This would be an example of ‘breasting‘ in a saw blade (Fig. 31). The moveable digit of *T. putrescentiae* shows this. There is a consistent central tooth (Fig. 10).

Strong breasting can be seen on both chelal digits of North American burrowing crayfish (*Cambarus* spp.) and the fixed digit of *Scylla serrata*. Should an astigmatid chela be closed sufficiently for the fixed digit tip to be opposed by the force at the moveable digit tip (Fig. 23f) then any ‘canine‘ tooth would be embedded into the foodstuff increasing the grip between the two.

**Fig. 32:**
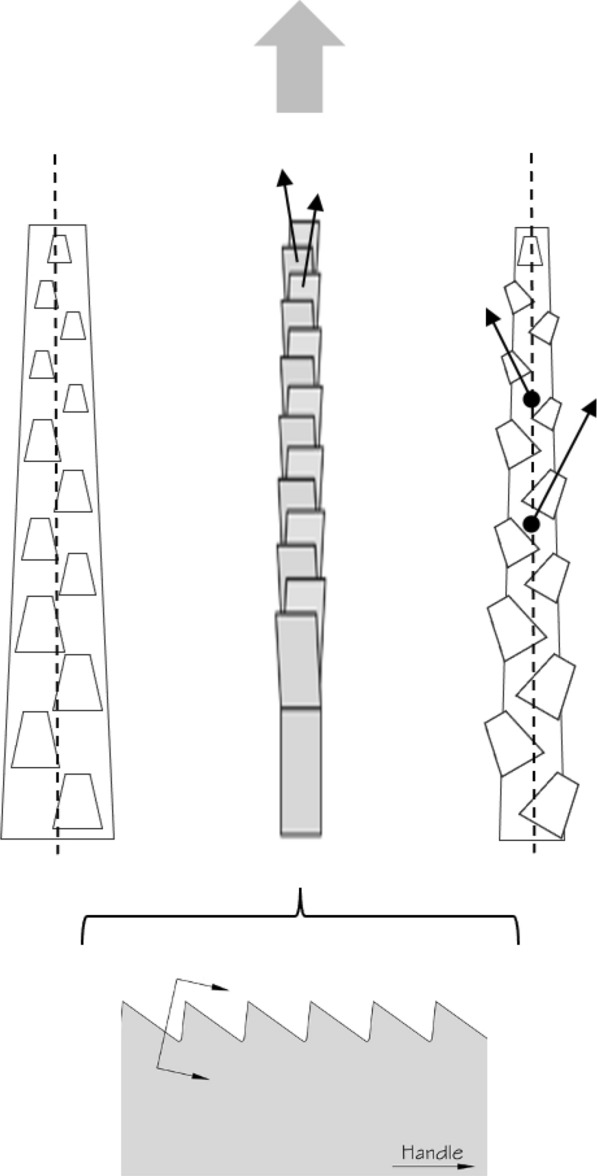
Action of moveable digit on being pushed into material (grey arrow). Views from above. Left: Two rows of chisel-like asperities staggered around dashed mid-line of astigmatid moveable digit ‘base plate’ ($\equiv$ horizontal ramus) anterior upwards. Mirror image staggering may occur on a matching fixed digit—see Fig. 7 in Johnston (1965). Middle and Lower: Rip saw teeth along saw plate showing tooth slope (black arrows) effectively yielding ‘set’ teeth. Annotated from: Rip teeth viewed from the side and the toe © Isaac Smith 2012 with permission. Right: Chisel-like astigmatid teeth showing slope with respect to their bases on mite moveable digit *L*2*M* axis (black arrows with basal dots). Note such a digit will saw a wider slot in food material

Counterfactually, to facilitate simple simultaneous grasping of foodstuff by both teeth, the tooth peaks should follow a gentle decline in line with the height of $hyp_{4}$ above the *L*2*M* axis for each more and more posterior location $x_{i}$. This is consilient with the jaw design of mammalian insectivores and crocodilians who grasp slippery prey. None of the three UK beehive species shows this, indeed *G. domesticus* shows a level point line on its moveable digit (Fig. 32).

Now consider, the fixed digit tip being further away from the condyle than the moveable digit tip is, which is such that on chelal closure there is excess fixed digit surface free. Note that such fixed digit tip elongation (‘overbite‘) increases the maximum effective (*G*) gape as the ‘jaw’ can be opened more before the end of the mastication surface constraint in Fig. 23b applies. Underbite ( = moveable digit length longer than fixed digit when the chela is closed) has the opposite effect. How do moveable digit teeth interact with this? Taking underbite first (Fig. 23d). Here

$$\begin{aligned} \alpha =cos^{-1}\left( \frac{L2M-x_{4}}{L2M}\right) \end{aligned}$$

and in this case for *Tyrolichus casei*

$$\begin{aligned} G_{4}\approx (L2M \cdot sin(\alpha ))-y_{4} \end{aligned}$$

This approximation being worse for larger and larger $y_{4}$. The tooth at this gape would press any food material held in the chela onto more sclerotised fixed digit features posterior of its tip. This may be of advantage for harder food material to be crushed (especially in Type B designs). For

$$\begin{aligned} \beta>>\alpha \end{aligned}$$

i.e., a chela opened a lot further (Fig. 23e) such that the force orthogonal to $[x_{4},y_{4}]$ now lines up with the fixed digit tip. This means that $G_{4}$ becomes undefined in that any food material engaged with by the *L*2*M* axis and any features beyond $[x_{4}]$ have nothing to push against. This reduces the overall effective mastication surface. Overbite has the advantage that (distal) gullets at $x_{4}$ which would normally fail to press food against the fixed digit tip for large chelal opening angles will now have something to push food against (this may be of advantage to Type A designs). Moreover curtailment of the effective mastication surface distally will not occur until very large angles of chelal opening. Any increase in $\beta$ over $\alpha$ of course increases the maximum food fragment size that can be stabbed (but not grasped) by the moveable digit tip, with the morsel volume being changed accordingly in a complicated way.

Similar calculations can be made for other asperities. It would be useful in follow-up work with other mites to check for an appropriate correlation between moveable digit type and fixed digit over- or under-bite when digit tip(s) mismatch features (e.g. uropodoid mesostigmatids, plant pest predators etc.).

### Tribological consequences—friction

Many models of surface contact have been proposed (Tavares 2015). Friction (https://en.wikipedia.org/wiki/Tribology) between touching surfaces can be counterintuitive. It is described by three laws:

1. Amontons’ First Law: The force of friction is directly proportional to the applied load.
  So acarine chelal moveable digit regions proximal to condyle have higher friction against food on crushing than distal ones (due to the normal force of ‘crunching’ $F2_{i}=F1.VR_{i}$ where *i* indexes location along the *L*2*M* axis).
  Further, that the more powerful the $F2_{i}$, the greater the friction between the moveable digit surface and food material (i.e., size and cheliceralisation matters in astigmatid chewing).
  Also, that the bigger the cheliceral retractive or hydrostatic protrusion forces, the greater the friction between food and the moveable digit surface at it slides posteriorly or anteriorly (i.e., size and gnathosomatisation matters in astigmatid chelal sawing).
2. Amontons’ Second Law: The force of friction is independent of the apparent area of contact.
  So drape distance (*m*) *per se* is not important in determining friction. Nor will the classification of the type of digit grossly affect it.
3. Coulomb’s Law of Friction: Kinetic friction is independent of the sliding velocity.
  So once moving, the kinetic friction of the moveable digit passing against food (by chelal closing or cheliceral movement) does not change with the speed of movement.

Friction is a current active area of research. If one assumes that astigmatid food material is elastomeric (i.e., like a rubbery material composed of long chainlike molecules, or polymers) then Popov et al. (2015) has a few useful conclusions.

- It is the surface gradient which determines the coefficient of friction on velocity.
  - So, the shape and thus the sharpness of any toothiness on the digit will matter on chelal retraction/protrusion. How this varies randomly or spikily will matter.
- As surfaces usually have larger surface gradients on smaller scales, then, usually the smallest scales give the main contribution to the coefficient of friction
  - So, even small scale sharpness features matter for astigmatid digit/food friction.
- The governing parameter of the contact configuration between surfaces is the ‘indentation depth’, and the latter is connected with the normal force through stiffness, which is determined practically only by the macroscopic form or the largest wave length contributions to the roughness.
  - So, the overall form (and thus type) of the digit (i.e., the “..macroscopic form of the indenter”) matters, and this is so even for the indentation of a viscous liquid or any viscoelastic material (e.g., amorphous or semi-crystalline biopolymers making up mite foodstuff) ‘chewed’ upon.

In other words “...the friction force is almost entirely dependent on the smallest-scale roughness, its weak dependence on the normal force is related only to the large scale roughness”. So, the ‘drape distance‘ (*m*) only determines the friction between the chela and foodstuffs it passes through on chewing if larger *m* gives the possibility of larger sharper asperities and gullets (and via a change in effective occlusive velocity ratio $VR_{i}$ the crunch force *E*[*F*2]).

Imperfections in surfaces (micro-hills and micro-valleys) are the source of dry friction between surfaces that are in contact with each other and rub against each other (like chelal digits and foodstuffs). Consiliently at human scale, steep ‘hooks‘ catch on clothing for instance. Decreased moveable digit smoothness *s* (shown in Tables 2, 6 and 10), or similarly $\sigma ^{2}$ in Tables 3, 7 and 11), that is increased rugosity (https://en.wikipedia.org/wiki/Rugosity), therefore has a disadvantage when stabbing chelae are pushed ‘open mouthed‘ into food material. Increased friction between their surfaces with its asperities and any (non-lubricated) food particles will occur.

**Table 11:** *Tyrophagus putrescentiae* Tribological parameters of mastication surface

Specimen	$R_{a}$ (μm)	$R_{q}$ (μm)	$\sigma ^{2}$	$\frac{\sigma }{R_{a}}$	$R_{p}$ (μm)	$R_{v}$ (μm)	$R_{t}$ (μm)	No. of peaks ($\eta _{p}$)	No. of valleys ($\eta _{v}$)	No. of crossings ($N_{0}$)	$\frac{N_{0}}{x_{i_{e}}}$
224(1)-2	0.45	0.59	0.223	1.04	0.71	− 0.69	1.40	3	3	5	0.38
224(1)-3	0.74	0.94	0.884	1.28	1.44	− 1.64	3.08	2	2	3	0.18
224(1)-4	0.80	0.97	0.764	1.10	1.10	− 1.49	2.59	2	3	4	0.30
224(1)-5a	0.78	1.11	0.832	1.17	0.94	− 1.85	2.79	2	2	3	0.21
224(1)-6	0.79	0.92	0.840	1.16	1.85	− 0.95	2.80	2	2	3	0.23
224(1)-6a	0.86	1.02	0.987	1.16	1.55	− 1.41	2.96	2	3	4	0.28
224(1)-7	0.82	1.10	0.867	1.13	1.46	− 1.40	2.86	2	3	4	0.27
224(1)-7a	0.40	0.51	0.245	1.24	0.87	− 0.53	1.41	3	3	5	0.37
224(1)-7b	0.59	0.69	0.439	1.11	1.07	− 0.95	2.02	2	3	4	0.26
224(1)-7c	0.52	0.65	0.408	1.22	1.27	− 0.83	2.10	2	3	4	0.32
224(1)-7d	0.63	0.97	0.949	1.55	2.32	− 1.60	3.92	1	2	2	0.15
224(1)-8a	0.48	0.64	0.400	1.30	1.42	− 0.74	2.04	2	3	4	0.24
224(1)-8b	0.71	0.91	0.737	1.20	1.46	− 1.27	2.73	2	3	4	0.29
224(1)-10	0.69	0.76	0.549	1.07	1.24	− 0.81	1.95	2	3	4	0.30
224(1)-10a	0.57	0.69	0.466	1.19	1.40	− 0.86	2.25	3	3	6	0.39
224(1)-14	0.98	1.25	1.350	1.18	2.16	− 1.51	3.27	2	2	3	0.21
224(1)-odd2	0.59	0.67	0.448	1.14	1.18	− 0.87	2.05	2	3	4	0.29
Summary	0.67	0.85	0.600	1.19	1.38	− 1.14	2.48	2	3	4	0.27
	(0.160)	(0.213)	(0.3036)	(0.115)	(0.426)	(0.399)	(0.665)	(0.49)	(0.47)	(0.93)	(0.068)
T13 ($n=20$)	0.59	0.94	0.491	1.22	1.18	− 1.19	2.32	2	3	4	0.28
Museum (n = 17)	0.52	0.73	0.373	1.19	0.90	− 1.09	1.97	2	2	3	0.28

For Gaussian surfaces $\frac{\sigma }{R_{a}}\approx \sqrt{\tfrac{\pi }{2}}=1.25$ (Bhushan 2000). Number of peaks and number of valley does not include moveable digit tip. Number of crossings does not include shift from $L2M=0$ axis at moveable digit tip, nor any shift after $x_{i{e}}$. Summary is mean, median or geometric mean as appropriate. SD in (....)

**Fig. 33:**
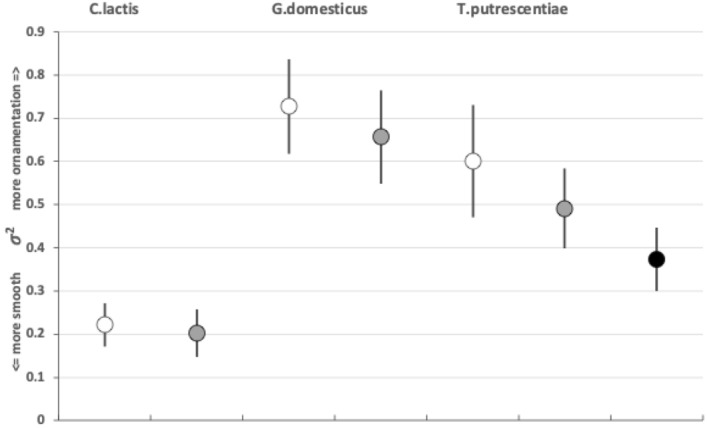
Approximate 95% confidence intervals and average over specimen values for moveable digit rugosity $\sigma ^2$. Open circles = wild collected specimens (Tables 3, 7, and 11). Grey circles = laboratory specimens. Black circle = museum *Tyrophagus putrescentiae* specimens

Consiliently at a much larger scale, tools like spears are smooth and sharp-sided therefore. Hills and valleys on the chelal digits will interlock into food ‘nooks and crannies’, deform and consequently oppose motion. A larger penetrative force will be needed by the action of the chelicera or the astigmatid body as a whole for the digits to cause plastic, elastic or break deformations in the foodstuff. Similarly, increased rugosity would mean increased friction when sharp chelal teeth are dragged back through food. The $\sigma ^2$ values show that moveable digits in *C. lactis* (irrespective of source) have markedly different rugosity than the other two species (Fig. 33). While not significantly different the acarid appears to have smoother digits overall than the glycyphagid which would agree with the latter having a saw-like action.

Note, from the above comparative *z*-test results, the features driving this rugosity have no direct functional impact upon the overall mastication surface velocity ratio (*VR*) so their importance must be mainly in terms of the hooking, sawing etc., details of food processing. Lubrication with a shear-able layer of fluid will reduce both static and kinetic friction, energy loss and waste heat production. This could be by *C. lactis* skimming in fluids or an extra perhaps supra-coxal gland secreted fluid being deployed over the chelae in the glycyphagid and acarid. The comparative form of the supra-coxal seta in the three species could be examined in a follow-up SEM study.

The curvature radii of roughness peaks is key in determining contact interactions between surfaces (Bigerelle et al. 2013). This requires very intensive surface sampling beyond comparative morphological zoologists. So in the interim, more work is needed to explore practical surrogates. Say if *Ov*[*VR*], since it measures the spikiness of moveable digit toothiness, might be approximately related to ‘large scale’ friction against food material as the surface is pulled or pushed and chewed during feeding (since *Ov*[*VR*] is linearly proportional to the variation in the normal force i.e., $Ov[F2]=F1.Ov[VR]$)? Indeed, given that there are insufficient semi-landmarks to consider any spectral decomposition of mastication surface deviations, perhaps one could look at using the moveable digit profile gradient $g_{i}$ values (used for the flex measure *f*) herein transformed into the velocity ratio domain as a simple basis of an appropriate ‘small scale sharpness’ measure? Perhaps one could use a velocity ratio version of the ‘RMS profile slope’ ($R\varDelta _{q}$, see Tavares 2015)?

Follow-up SEM work could look for nano-scale features in the architecture of moveable digits in newly eclosed adults. In isopods there is an absence of ridges on heavily loaded surfaces which may enhance the dissipation of forces (Vittori 2021). Could this occur proximally (where there is high crunch force (*E*[*F*2]) on chelal occlusion) especially in the glycyphagid? In the spider *Cupiennius salei*, the epicuticle on the active, concave (ventral) side of the claw is thickened and shows surface irregularities at various length scales which increase the friction with the surface and secure the grip (Politi et al. 2021). Are such present in astigmatid digit ‘pockets’? The smoothness of the surface of the fang tip in *Cupiennius salei* suggests that friction is reduced as compared to the ventral surface of the claw. This smoothing of the surface allows the spiders to use their fangs to repeatedly puncture prey cuticle and to easily retract the fangs. Is the same within tooth surface differentiation (to manipulate frictional forces) present in astigmatid moveable digit teeth?

### Tribological consequences—wear

**Fig. 34:**
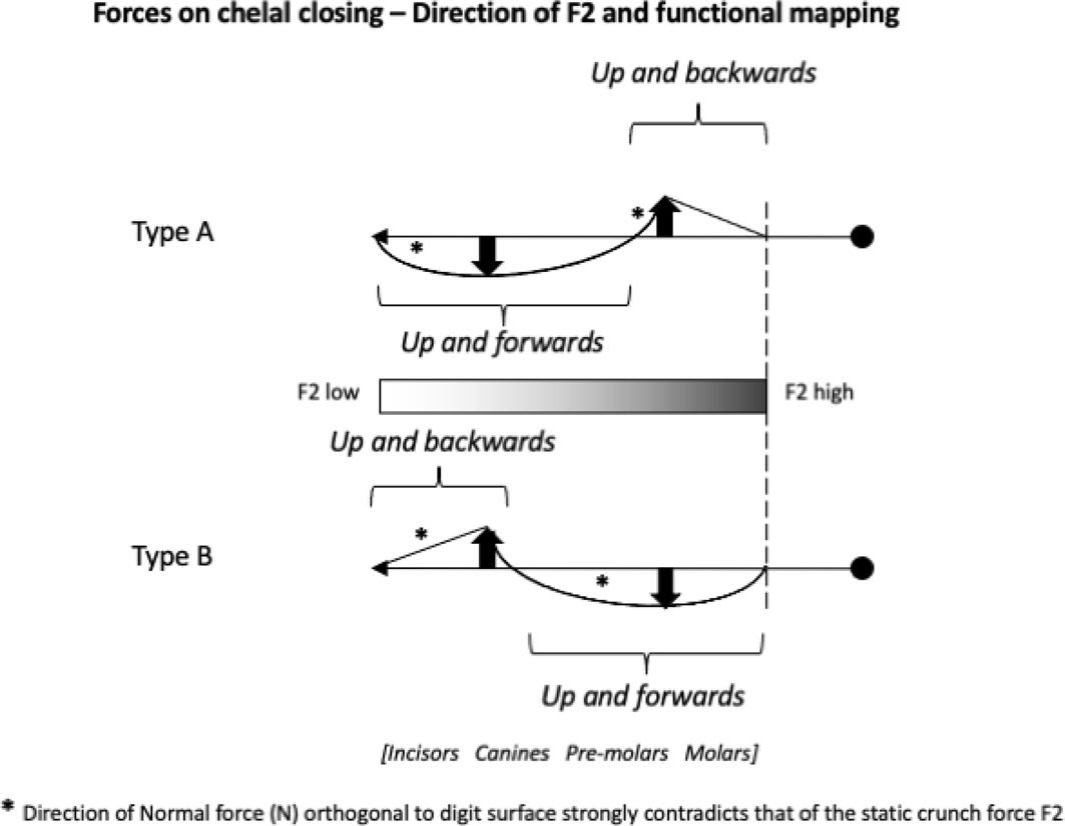
Moveable digit regions have conflicting forces on chelal closure

According to the moveable digit design type, different regions will show conflicting forces on chelal closing (Fig. 34). Bigger teeth and bigger gullets means, on balance, the chance of higher friction on cheliceral/idiosomal retraction when the mite is tearing at foodstuff. However, there is no straightforward estimate of this retractive force. So assuming that it is likely to be proportionately similar to *F*1 as a guide, shows that the $R_{t}$ values do rank appropriately (Tables 3, 7 and 11). However, wear into the foodstuff surface caused by the digits is actually not always in direct correlation to the friction between them.

An obvious wearing action is (two body) abrasive wear which consists of the cutting effort of the hard ‘crystalline‘ chelal chitinous surfaces acting upon any softer food surface. Here, with respect to wear, not just smoothness (*s*) but also independently the roughness $\sigma ^2$ (in Tables 3, 7 and 11) of the profile matters as the tips of asperities cut off material against which they rub. So the rougher form of the moveable digit in *T. putrescentiae* (average smoothness values = 0.078) will wear food more on cheliceral retraction than that of *G. domesticus* (average smoothness values = 0.046), which will in turn will wear food more than that of *C. lactis* (average smoothness values = 0.012).

Given that the chitinous surface and the mite’s normal food stuff are assumed to have very different levels of hardness (and the former is expected to be tougher than the latter), asperities on the mastication surface should move across the soft food surface with a micro-ploughing mechanism. At the microscopic level, a prow will be formed ahead of the abrading digit micro-feature and food material will be continuously displaced sideways to form ridges adjacent to any groove produced. With repeated cheliceral passes, many grooves will be formed parallel to the direction of displacement of the abrasive asperities. This could be looked for in a follow-up SEM study. The proximity of the grooves eventually weakens any ductile food material which deforms locally and matter is removed with a micro-fatigue mechanism (Zum Gahr 1987).

Shredding (as posed for *G. domesticus*) depends upon wear occurring in the foodstuff. Looking at $R_{v}$ values (Tables 3, 7 and 11) suggests that the micro-valleys are acting as expected in helping shed abraded food material material from the digits across the three species. However, examining $R_{p}$ values suggests the greatest wear of the foodstuff surface is by *T. putrescentiae* and *G. domesticus* and the least by the moveable digits of *C. lactis*. Note that tougher food is associated with more sclerotised digits (Fig. 21b). For *G. domesticus* (and for any astigmatid puncturing arthropod or nematode cuticle with their chelae) two-body abrasion by a higher level of digit surface hardness may act differently. The (larger) microasperities ($R_{p}$) on any harder surface cut the more ductile foodstuff surface cleanly, with no plastic deformation, using a micro- cutting mechanism on cheliceral retraction. The shape and volume of the resultant groove corresponding exactly to the volume of material displaced. This may be how *T. putrescentiae* gleans.

If in addition these two surfaces are subjected to high pressure, some corresponding surface asperities formed in the foodstuff may become detached by a micro-cracking mechanism. Small cracks then form along the main groove, which propagate and then nucleate within the food material, with resultant ‘blocks’ of material then becoming detached (Zum Gahr 1987). Could this be the nut-cracker action in *T. putrescentiae* browsing?

With repeated cheliceral passes, all the food surface asperities are subjected to one or more of these actions and become dissociated so that the cumulative effect of these microscopic losses results in macroscopic wear (i.e., foodstuff trituration). This shift in how the digit acts from tending to ‘crush‘ to tending to ‘cut‘ may be the systematic change in *T. putrescentiae* in the UK beehive sample compared to the expected mode of action indicated by their tip velocity ratio (*VR*) values. Do wild acarids in fact browse/glean upon something needing scraping and slicing with comparatively larger teeth? Is this design why *T. putrescentiae* can be a facultative predator of some bees (da Silva et al. 2022) and soil nematodes?

Three body abrasive wear as well as adhesive wear, fatigue wear (due to repeated ‘chewing‘), fretting, and particle-driven erosion (but not corrosive wear) may also occur to foodstuff due to astigmatid chelal action. These are not discussed herein. However, follow-up SEM work could look for nano-scale wear changes in moveable digits by comparing surfaces between say newly eclosed adults and those near the end of their life (especially for durophagous species).

In isopods (Vittori 2021) there is selective texturing of surfaces which may reduce wear by eliminating small particles. Might this be occurring in astigmatids and in turn be ‘worn-out’ by chelal use? In the spider *Cupiennis salei* (Politi et al. 2021 the rough epicuticular surface of a claw is more prone to wear than a smooth surface would be. This risk is mitigated by an increased wear-resistance of the cuticle at the ventral surface as compared to the base of the claw achieved by the incorporation of a small amount of Mn and Ca ions that act as cross-linkers in the proteinaceous matrix. This leads to abrasion tolerance at similar levels even to some highly mineralised matrices such as human enamel and mollusc nacre. Does this also happen in astigmatids?

### Moveable digit blades, tearing hooks and saw-like features

Tribology distinguishes various models of two-body abrasion suitable to describe mite cheliceral chelal actions, determined according to the angle of attack and the geometry of the asperities, the friction coefficient, the speed of their displacement, the pressure, the distance and the differential hardness between the two active surfaces in contact (Abebe and Appl 1998, Zum Gahr 1998).

Individual moveable digit asperities could be considered for instance to act like arrow-heads or like wood chisels on chelal closing. As such their width and the cross-sectional shape of their sharpening determines their masticatory efficiency.

Such forms could be arranged linearly along the *L*2*M* axis, offset (like Viking shipwright Side-axes which keep their handles free of the woodwork https://regia.org/research/ships/Ships1.htm), or even staggered (Fig. 32). Chisels scrape material. The arrangement of alternate fixed and moveable digit teeth of *Sancassania (Caloglyphus) berlesei* (illustrated in Fig. 7 of Johnston 1965) may function like this as they pass over material. Arrow-heads of different form have different functions (see https://en.wikipedia.org/wiki/Arrowhead) and there are at least 13 types of chisels each of different specific uses (https://en.wikipedia.org/wiki/Chisel). Blunt moveable digit asperities crush (like vertebrate pre-molars and molars), sharp asperities cut or impale (like vertebrate incisors and canines). A chisel sharpened to a lower angle will penetrate material with less effort than one sharpened with a higher bevel, but *per force* will be less durable. A follow-up SEM study could examine the facets of individual astigmatid teeth to classify them as arrow-heads or as chisels and look for grip and wear adaptations on each.

**Fig. 35:**
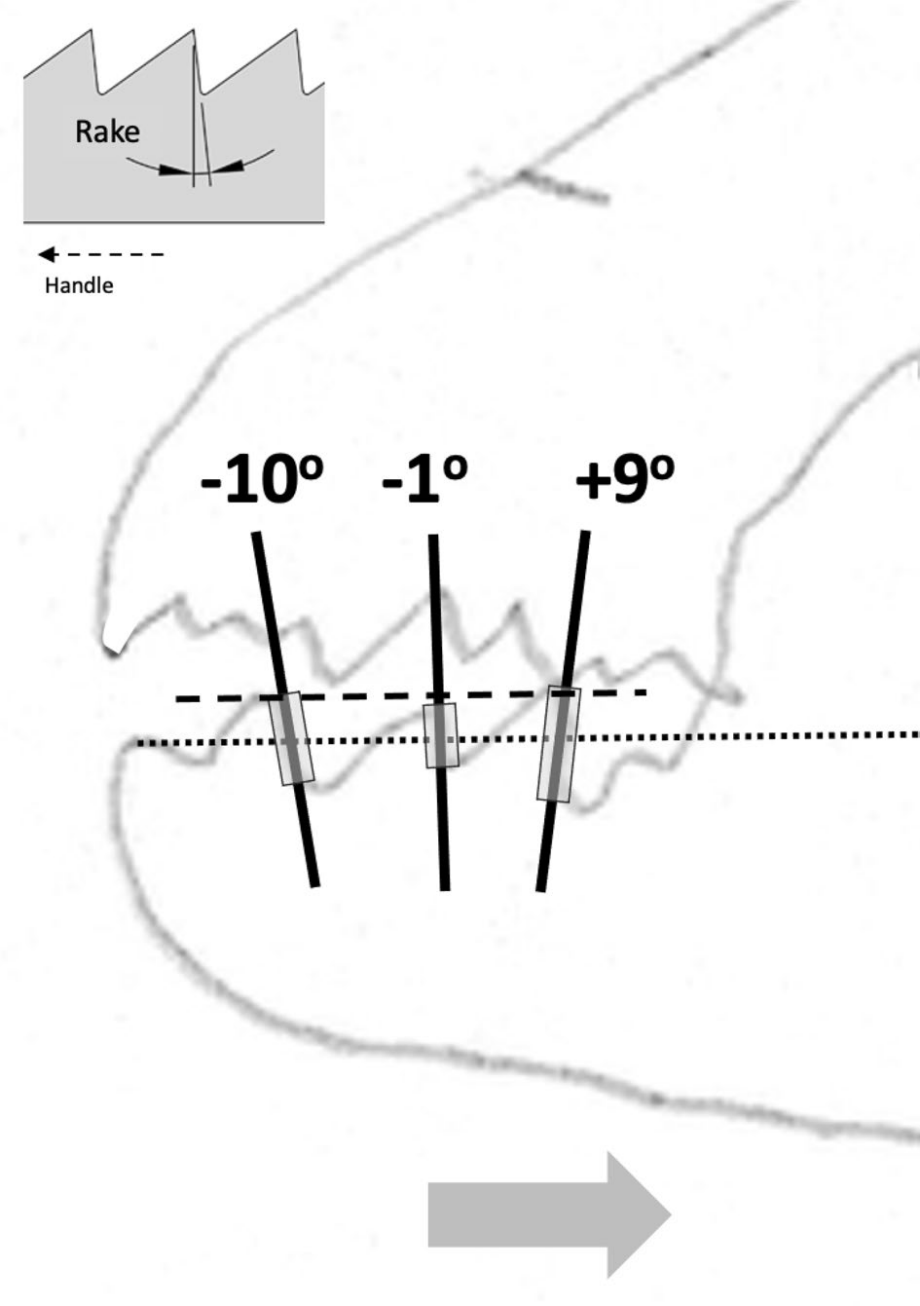
Complex saw-like moveable digit teeth in *Glycyphagus domesticus* (specimen 224(1)-2) on retraction of chelicera (large grey arrow). Food material assumed to be resting between digits and around embedded chela. Note apparent fourth tooth around start of ascending ramus. Dashed line = the saw’s ‘Point line’ showing the three major teeth are of similar size (with respect to *L*2*M* axis). Cutting surface of each tooth with point slope (Fig. 32) proximal to the condyle highlighted in grey boxes. Dotted line = tip of moveable digit to articulating condyle ($\approx$ saw plate). Pitch (number of teeth per unit distance) fixed. Rake, which varies efficiency of cut, explained in top left subfigure (© Isaac Smith 2012 with permission). Positive rake angle of tooth ($0{-}20^{\circ }$) bites into softer materials and is harder to start moving past the food. As it hangs onto material ("grabber cutting") the saw blade climbs up as it digs in (indirectly occluding the chela). It thus needs more experience to use such a saw. A low (less than 5°), neutral (0°) or negative hook (down to − 7°) produces a smoother, slower cut, and is ideal for hard and brittle materials such as those food materials similar to melamine and metal. Negative hook angles also inhibit the blade’s tendency to climb the material being cut (which means that they are a must for use in miter and radial arm saws). Note large digit depth to resist dorsoventral and torsional forces (Bowman 2023a) like a backsaw’s reinforced back providing stability (https://en.wikipedia.org/wiki/Backsaw). Saw action moveable digits need condylar strengthening to prevent joint dislocation

Cuticular mineralisation may make the digits even stiffer, so whilst strength may increase they could be more fracture prone. Indeed, a degree of condylar strengthening to prevent joint dislocation or digit breakage would be needed for any substantial sawing ‘to and fro’ action of the moveable digit within food material (*G. domesticus*, Fig. 35).

Any shortening of the mastication surface restricts the number of special features it can biologically provide along it, encouraging the consolidation of features (for instance multiple teeth merging into blades). Thus the moveable digit of low $\frac{CLI}{IL}$ or low $\frac{L2M}{CLI}$ is more likely to have undergone this merging during evolution than one with a high value for such (i.e, merging or feature loss should occur given the particularly short chela for a mite of that reach or that body size). Chelal digit blades can be considered to function like axes, adzes or knives depending upon the force applied by the moveable digit over their length (i.e., their actual average *F*2 adjusted for their null *E*[*F*2] given for those locations when considered as a simple bar-like beam) and the ‘grind‘ or cross-sectional shape of their sharpening (e.g., V or flat, lenticular or convex, asymmetrical semi-convex, asymmetrical flat, compound double bevel, hollow or concave, chisel, chisel with flat grind edge, chisel with back bevel, chisel with Urasaki etc.,). Different shapes lead to chelal tools that could function as a ‘fillet knife‘, or a ‘gullet knife‘, or a ‘meat cleaver‘, or a ‘scraper‘, or a ‘gouger‘ etc. The shape of the different bevels rectifies the resistive forces of the foodstuff into different compressive forces in the teeth and thus the likely pattern of sclerotisation in cheliceral digits. More SEM characterisation of astigmatid chelae is needed.

Long blades have a particular advantage (as shown by the form of shipwright axes used in historical boat building, see the Bayeux Tapestry for instance). Long blades slightly offset from the exact horizontal axis of their handle allow the longitudinal splitting of material (such as felled tree logs into planks). Is there such an offset in *C. lactis*? Long blades can also be used for gouging linear features in semi-solid material (or ‘skimming’ in fluids). Any structure laminated into distinct layers like angiosperm and gymnosperm pollen grains (with their intine and multiple layered exine of various forms Doyle 2009) could be split and layers peeled away through the skilful use of such blades by say *C. lactis*, much as boat builders fashion objects from layered wood. The perispore, endospore and exospore could similarly be split apart in pteridophyte and bryophyte microspores. SEM characterisation of beehive pollen attacked by astigmatids (and whether this is different between angiosperm and gymnosperm species, which vary in their exine structure Doyle 2009) together with looking for enzymic degradation (as in pollenophagous mesostigmatids Royce and Krantz 1989) would be very useful in a follow-up study.

Considering the stationary movable digit just as a slender rod-like cantilever fixed at the condyle would indicate that downward forces along it will induce a longitudinal (i.e., parallel to *L*2*M*) stretching stress in the dorsal surfaces and a longitudinal compressive stress ventrally (Lautrap 2011). This could be balanced by sclerotisation stiffening the digit fabric here. A computerised micro-tomography/finite element modelling structural analysis (like Tadayon et al. 2020) of astigmatid chelicerae in a follow-up study could be useful in investigating if this is the case.

**Fig. 36:**
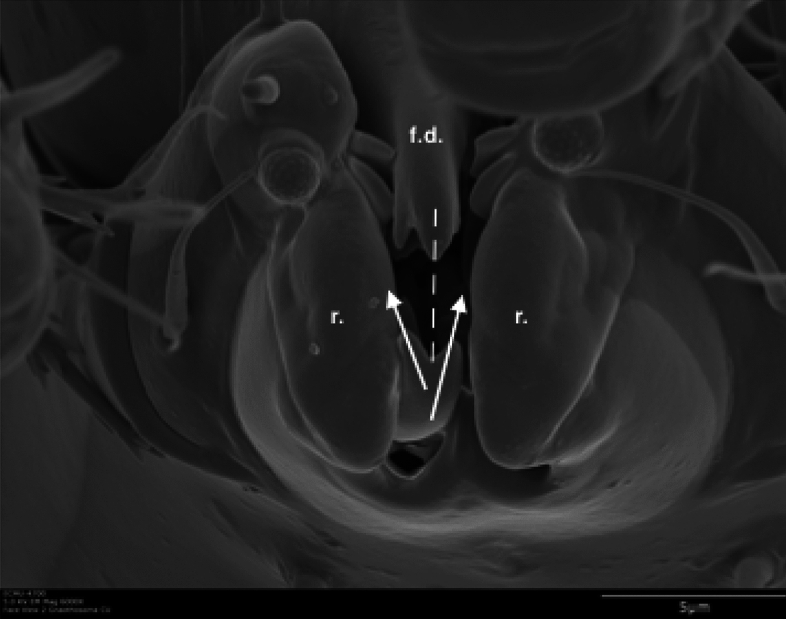
*Tyrophagus putrescentiae* from anterior showing ‘set’ (white arrows) in moveable digit teeth. Dashed line = vertical axis of digits. i.d. = fixed digit. r. = rutellum. Note fleam on fixed digit left hand tooth. Horizontal distance between rutella $\approx$ 2.0 μm. Annotated from Plate B351_016.tif © Pavel Klimov with permission

Any multiple peaked asperities merged into blades can be considered together with their valleys (gullets) in between each element as saws. A glossary of terms for saw design can be found at http://www.disstonianinstitute.com/glossary.html. Trivially, one deploys more teeth per unit distance on a saw ($\equiv$ higher pitch) for harder material. Fewer teeth is associated with cutting softer material. The geometry of saw teeth is more complex than that of planes or chisels but the basic elements are easily understood when they are examined individually. The six elements that control saw function and apply to the design of astigmatid chelae are: ‘Set‘, ‘Fleam‘, ‘Pitch‘, ‘Rake‘, ‘Point slope‘, and ‘Breasting‘. These will be considered in that order with respect to possible astigmatid mastication surfaces (Fig. 31).

Although there are many specific saw types (https://en.wikipedia.org/wiki/Saw), traditionally Western saws are of two types—‘rip-teeth saws’ and ‘crosscut teeth saws’. Can these designs be mapped to the beehive astigmatids? In both cases the teeth are bent away outwards (‘set‘) from the long axis of the blade, each tooth in the opposite direction to the one next to it (Fig. 36). This means that the resultant cut in the food material ($\equiv$‘kerf‘) would be wider than the moveable digit ‘saw blade‘ itself. This prevents it from getting stuck due to friction and binding as it cuts into foodstuff. However, the greater the set (i.e., the amount of offset the digit teeth have to either side of the tooth line) and thus the wider the kerf, the more material is removed and therefore the more work is required to push or pull the saw ($\equiv$ chelal retraction force back into the idiosoma or protrusion outwards in mites). Likewise the wider the kerf the sloppier the action as the digit basal ‘saw plate‘ can now wiggle about in the wider kerf and throw off a precise cut in the food.

This is why joinery saws (for careful cutting) always have less set than rough work hand-saws. More set usually yields deeper and rougher cuts. Now, softer and/or wetter material needs more set in an acarine digit saw because the spongy and sticky detrital ‘dust‘ generated by foodstuff trituration will not clear as readily from the kerf and more room is needed for the ‘saw‘ to run. If the kerf gets tight not only will the saw bind, but it can deflect in the kerf as it tries to find a way around the build-up ‘detrital dust‘, thus making the saw not run true.

High set blades are hard to cut straight with but easier to correct when cutting inappropriately. Less set blades cut drier material more smoothly and shallowly. They are easy to cut accurately with but harder to correct when they cut wrongly. As such astigmatid mastication surface geometries should be related to the typical moisture content of food material that they consume.That is, the presumably drier food-liking *G. domesticus* should be designed like a joinery saw and the moisture-loving tyroglyphid group like a rough work-saw design (when examined by say SEM in a follow-up study). Note that less set teeth allow the possibility of a ‘taper ground’ base plate or moveable digit cross-section much thinner than its normal thickness. Is this the case in algophagids and bladed fluid-loving carpoglyphids? Where does the wet cadaver-loving *Sancassania (Caloglyphus) berlesei* fit in with this?

**Fig. 37:**
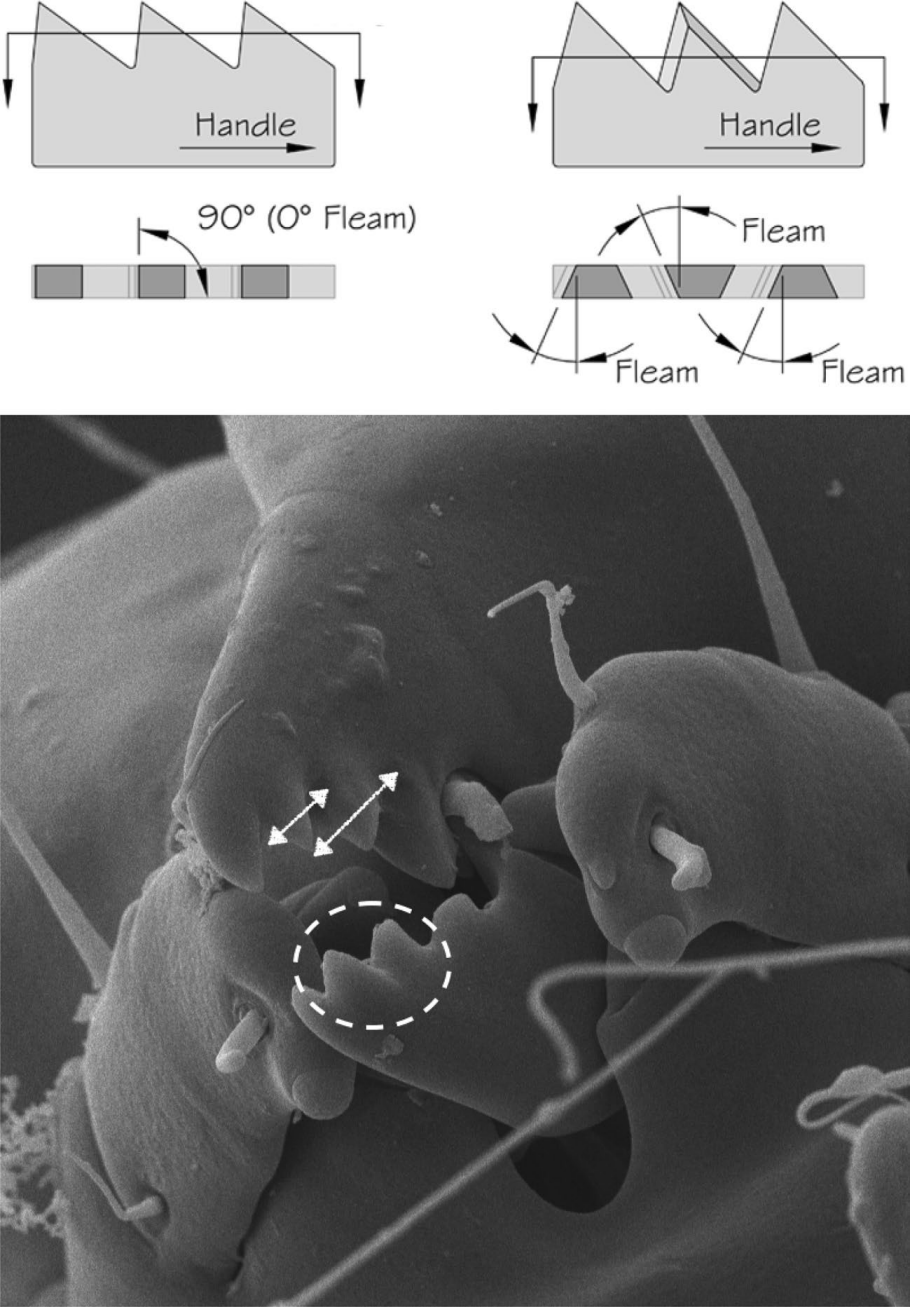
Adding fleam produces sub-conical asperities. Upper. Left: No fleam on rip saw teeth. Right: Fleam on a cross-cut saw lacking any set (or slope to the teeth). Amended from: ‘Fleam on rip and cross cut saws (no set shown)’ © Isaac Smith 2012 with permission. Lower. *Thyreophagus* sp. Arrows indicate fleam on fixed digit teeth making then sub-conical. Note also prominent sloped gullet behind second moveable digit tooth in centre of dashed circle and diagnostic difference in gullet minima. The horizontal distance between the rutella is $\approx$ 9.9 μm (cf. morphometrically-estimated mean values for *thick* in Bowman (2023a) of 10.4, 18.0 and 15.8 μm for *Carpoglyphus lactis*, *Glycyphagus domesticus* and *Tyrophagus putrescentiae*, respectively). Horizontal width of hypostomal notch below moveable digit $\approx$ 4.3 μm. Annotated from Thyreophagus_sp_f7_BMOC 20-0101-065.tiff © Pavel Klimov with permission

Cross-cut saw teeth are also beveled on their inside edges so as to slice through difficult material like a series of little knives. Is this what Akimov (1985) is trying to show for *T. putrescentiae* (see Fig. 12)? This bevelling is called ‘adding fleam‘ (Fig. 37). Hard material requires a smaller fleam angle on a cross-cut saw than the angle needed for soft materials. So *G. domesticus* is then predicted to have a small fleam angle on its digit teeth. An examination of the digits of *G. domesticus* in a follow-up scanning electron microscope study would be useful. Conversely the three basal teeth on *C. lactis* despite their small size may have both set and appreciable fleam so as to handle softer wetter material.

Bowman (2023a) points to some other triturative mechanism than the rutella-based trimming of food fragments being needed to generate the internal 0.5–2 μm food fragment size illustrated by Hubert et al. (2014) in astigmatid guts. For at least *Thyreophagus* sp., if material was dragged through the hypostomal notch (Fig. 37) this would only trim it to about 4 μm. Rather it would appear that these fragments in the gut better approximate the ‘detrital dust’ of moveable digit teeth sawing at food while it is retracted back between the rutella (Fig. 36), since their sizes match the roughness and scale of moveable digit asperities and gullets (see $R_{a}$, $R_{q}$, $R_{p}$, $R_{v}$ and $R_{t}$ in Tables 3, 7, and 11). It seems that this mechanism is a good candidate for a subsidiary oral trituration process whereby food material that has been grabbed, trimmed laterally and dorsoventrally (Bowman 2023a) is partly sawn up before final ingestion.

Cheliceral teeth with high fleam (i.e., ‘smooth cutters’) would themselves need to be strengthened by sclerotisation (=‘hardening‘) compared to other teeth as fleam weakens teeth and reduces the tooth-front facing the material. So, as such, high fleam saws cut more cleanly and should be used on softer material that will not push back so much on the more fragile teeth. Is this the case with the chelal digits of *T. putrescentiae* or *C. lactis*? Are they size-for-size less or more sclerotised? Given that these are actinochitinous species, would a quantitative birefringence follow-up study comparing different astigmatid chelae help?

Less effort is required to move high fleam teeth through material. Conversely a saw tooth with little to no fleam (= a ripsaw) will leave a rougher cut and require more effort (the latter being the cheliceral retraction force back into the idiosoma or protrusion force outwards) to move through the material. This design may thus be restricted to large powerful mites (like those listed in Bowman 2021c)? High fleam teeth would be expected to be found in the other soft food feeding astigmatids (e.g., those listed in Bowman 2021c). A follow-up SEM study would help confirm or refute this postulate.

Rip teeth do not have an angled edge, which means they work more like little chisels, scraping material away rather than cleanly slicing through it without ‘splintering‘ and tearing it. This could be the standard design for ‘shredder‘ mites (perhaps therefore for *Acarus siro*?). Rip blades often have fewer teeth and can handle a faster feed rate (producing more waste heat). Is this the case for particularly voracious astigmatids or pest species?

Saws can vary in the height (and depth) of their asperities to produce hybrid tools. Raising the height of a feature would be ideal for cutting thick food material but reduces the ‘engaged-tooth ratio‘ for the whole mastication surface. So comparing $\eta _{p}$ values for the UK beehive sample in Tables 3, 7 and 11 suggests that *G. domesticus* can triturate thicker food material than either *T. putrescentiae* or *C. lactis* but that of *C. lactis* engages along a noticeably longer foodstuff surface than either of the other two species (cf., compare drape distance *m* values). Amongst the UK beehive sampled individuals, $R_{t}$ for *C. lactis* is significantly lower on average than *T. putrescentiae* (Welch‘s t = 4.589, df = 36.257, p value = 5.169 × 10^−5^) and thus must also be distinct from that of *G. domesticus*. However the $R_t$ values for the acarid and glycyphagid are similar (Welch‘s *t* = 1.5591, df = 23.292, p value = 0.1325). On the face of it the mastication surface of *C. lactis* is a finer dissected saw than that of the other two species. However, the three are not simply shrunken/swollen versions of each other as Bowman (2021c) concluded using laboratory cultures, since comparing idiosomal length scaled $R_{t}$values (i.e., $1000 \times \frac{R_{t}}{IL}$, Tables 4, 8 and 12) shows that although on average these are similar between *T. putrescentiae* and *G. domesticus* (Welch‘s *t* = 2.3842, df = 21.128, p value = 0.02657), that of *C. lactis* is very distinct from its nearest neighbour *T. putrescentiae* (Welch‘s *t* = 4.2331, df = 25.432, p value = 0.0002638). Size for size, the carpoglyphid has a lot less dissected mastication surface.

**Table 12:** *Tyrophagus putrescentiae*—stochastic characterisation

Specimen	$\widehat{E[VR]}={\hat{\varTheta }}$	Ov[VR]	$\widehat{Var[VR]}=\widehat{\varPhi ^{2}}$	$\frac{1000\cdot R_{t}}{IL}$
224(1)-2	0.714	0.0473	0.0481	6.90
224(1)-3	0.681	0.0320	0.0321	13.89
224(1)-4	0.623	0.0180	0.0184	10.25
224(1)-5a	0.660	0.0204	0.0205	9.99
224(1)-6	0.595	0.0172	0.0175	11.47
224(1)-6a	0.616	0.0179	0.0183	10.52
224(1)-7	0.617	0.0241	0.0248	11.42
224(1)-7a	0.662	0.0298	0.0298	5.91
224(1)-7b	0.640	0.0205	0.0205	6.54
224(1)-7c	0.724	0.0261	0.0261	8.85
224(1)-7d	0.651	0.0200	0.0201	15.20
224(1)-8a	0.725	0.0354	0.0354	7.58
224(1)-8b	0.585	0.0118	0.0120	10.68
224(1)-10	0.691	0.0319	0.0321	8.31
224(1)-10a	0.799	0.0581	0.0583	9.07
224(1)-14	0.554	0.0110	0.0111	12.35
224(1)-odd2	0.727	0.0355	0.0357	8.08
Summary	0.663	0.0244	0.0247	9.82
	0.0628)	(0.01242)	(0.01247)	(2.562)
T13 (n = 20)	0.621	0.0248	0.0260	10.87
Museum (n = 17)	0.633	0.0188	0.0192	11.35

Summary is mean, median or geometric mean as appropriate. SD in (....)

Woo et al. (2022) recently investigated bacteria and fungi associated with *Dermatophagoides* spp. in house dust. Xerophiles like the food contaminant fungus *Wallemia* (Zajc and Gunde-Cimerman 2018) as well as mycoparasitic yeast such as *Cystobasidium* were found. *G. domesticus* and *T. putrescentiae* can be found in houses. Could the former microbe be grasped by and then sawn by astigmatids in general (including the UK beehive ones)? The cell or hyphal diameter of *Wallemia* spp. vary from about 3–9 μm depending upon salinity conditions (Kunčič et al. 2010) which represents less than 50% of the gape reported for the three *Dermatophagoides* species studied by Bowman (2021c). So, they could be grasped in the chela. Soil *Cystobasidium* species are 2–7 μm wide (Abu-Mejdad et al. 2019), so they could be easily grasped too. Indeed even the average length of the hyphal compartment in *Wallemia muriae* and *Wallemia sebi* at less than 20 μm means it could be grasped lengthways. Cell-wall thickness in the species studied by Kunčič et al. (2010) at around 0.25–0.75 μm on average approximates at best only 50% the $R_{t}$ size of astigmatids if *C. lactis*, *G. domesticus*, and *T. putrescentiae* are typical. So by analogy to a full-scale tool cutting a sheet of wood, an effective saw action against *Wallemia* is possible.

The glycyphagid and acarid specimens had approximately 2–5 teeth per mastication surface visible on micro-examination. As a general guideline for a saw, one wants at least five or six teeth in a cut at any time, otherwise the saw will catch too easily. However, as the fineness of a saw cut increases, so does the number of teeth in the cut, although a limit is reached when the gullets of the teeth (= depressive regions between them) become too small to carry out the detrital ‘dust‘ or ‘shavings‘, and the saw blade begins to bog down. With modest numbers of digit ‘mountainous regions‘ on astigmatid digits, as a saw they may ‘catch‘ into foodstuff material from time to time as the moveable digit moves saw-like back and forth. Observations are needed as to how mites might deal with this during feeding.

The diameter of the tiny fragments observed in the gut of *C. lactis* by Hubert et al. (2014) are smaller than hyphal widths and better approximate the scale of chelal macro-waviness (i.e., $\sqrt{(}\sigma ^{2})$) and the size of digit teeth and gullets i.e., $R_{p}, R_{v}, R_{t}$ (see Table 3, 7 and 11). As posed above, these fragments almost certainly represent detrital dust or ‘shavings debris‘ from the action of the chela as a saw on the food material either when initially grabbed or when a chunk is repeatedly attacked whilst being constrained by the rutella and held between the pedipalps. Could this above action be the sought-for pre-ingestion trituration process? That is astigmatid crunching on one chelal closure is more to do with holding material that is then sawn at by the other chela? This action being alternate but not necessarily the ‘take it in turns’ chewing implicitly assumed by acarologists to date?

Note that unicellular spore fragments are < 1 μm in size Yamamoto et al. (2012). Given the tiny oesophagus diameter, in that way even large morsel grabbing powerful ‘shredding‘ astigmatids must also intensively triturate their food material before ingestion (and not just masticate it by a crunch-like chewing). The maxim parents say to children: “chew your food a hundred times before swallowing“ comes to mind here. However vegetables on the dinner-plate may need to be sawn-up first (as well as meat needing to be cut up, ‘sauce’ gleaned and small items ‘fished’ for).

This is all energy intensive and for any astigmatid consuming low nutritional value foodstuffs would mean that, like vertebrate ungulates, having grabbed a chunk of food such mites must almost continually orally attack it. Are degrading salivary enzymes added pre-ingestion? Does the lateral supracoxal gland deliver lubrication by a capillary action? Do the beehive species have to essentially sit and munch all day to extract enough nutrition? Observations on the relative continuity of feeding in different astigmatids is needed in follow-up work.

Harder material needs more teeth per inch (‘pitch‘) in a saw blade than soft material (NB pitch here is not the same use as the term pitch in aerodynamics). Alternatively a faster, rougher, low quality finished cut which removes more material (i.e., a ‘tear-out‘) is produced by fewer teeth per inch. Cross cut blades have about twice the number of teeth than rip blades. Usually the finer the pitch of a saw the smaller the set of the blade. So, thinking of the astigmatid moveable digit as a saw blade, as the number of saw teeth per digit length would be approximately twice $\frac{N_{0}}{x_{i_{e}}}$ in Tables 3, 7 and 11, this suggests that *G. domesticus* and *T. putrescentiae* can handle tougher food than *C. lactis*. However, considering $N_{0}$ (number of crossings) values, the moveable digit of *C. lactis* would triturate food material faster than *T. putrescentiae* which in turn would be faster than *G. domesticus*. In terms of the relative numbers of crossings, the moveable digit mastication surface of *C. lactis* is acting like a crosscut blade, that of *G. domesticus* much more like a rip blade (with that of *T. putrescentiae* a hybrid tool somewhere in between). This is partly confirmed by pitch being estimated as the subjectively scored $\tfrac{Number\ of\ teeth}{x_{ie}}$ in Tables 5, 9 and 13.

**Table 13:** *Tyrophagus putrescentiae* sorted by type

Specimen	Type	CLE	*γ* (°)	Pocket *EVR*	Pocket vol.	F1	Pocket *E*[*F*2]	Slicing blade Retr.	Cutting edge Retr.	Slicing blade Protr.	Cutting edge Protr.
224(1)-3	A	14.8	144	0.638	269.0	2103.9	1342.3	2.6	–	–	2.0
224(1)-6	A	11.5	141	0.562	118.1	1814.8	1019.9	3.6	–	–	2.6
224(1)-7	A	10.2	133	0.528	177.5	2209.0	1166.3	5.2	–	–	2.1
224(1)-7b	A	11.4	147	0.578	93.0	2254.6	1303.2	5.9	–	–	1.5
224(1)-7c	A	09.5	138	0.655	80.2	1691.3	1107.8	4.7	–	–	1.8
224(1)-7d	A	10.5	118	0.592	180.5	2232.9	1321.9	5.6	–	–	3.3
224(1)-8a	A	11.2	143	0.621	160.1	2296.9	1426.3	5.5	–	–	2.0
224(1)-8b	A	09.9	144	0.537	116.2	1906.6	1023.8	7.8	–	–	2.1
224(1)-10	A	08.8	131	0.592	99.1	1459.3	863.9	4.6	–	–	1.7
224(1)-10a	A	10.9	146	0.675	98.0	1745.5	1178.2	4.8	–	–	2.0
224(1)-14	A	11.7	131	0.526	253.8	2187.5	1150.6	6.4	–	–	3.1
224(1)-odd2	A	10.8	149	0.651	93.0	1962.9	1277.8	3.3	–	–	1.7
224(1)-6a	B	03.9	67	0.772	103.7	2651.8	2047.2	–	2.2	10.6	–
224(1)-5	B	10.0	39	0.740	996.9	1890.4	1398.9	–	1.3	3.0	–
224(1)-8	B	04.8	88	0.924	86.3	1886.6	1743.2	–	1.4	9.6	–
224(1)-2	Indet.										
224(1)-4	Indet.										
224(1)-5a	Indet.										
224(1)-7a	Indet.										
	A	10.9	139	0.596	144.9	1988.8	1181.8	5.0	–	–	2.2
	B	06.2	65	0.812	395.6	2142.9	1729.8	–	1.7	7.7	–
	Overall					2039.8 (297.32)					

Specimen	Shank of hook Retr.	Total	Tear prop. Retr.	Shank of hook Protr.	Total	Tear prop. Protr.	$\tfrac{CLE}{CLI}$	$R_p[VR]$	$R_p[F2]$	$x_{R_p}$	No. teeth^a^	Pitch^a^
224(1)-3	16.8	31.4	0.47	–	–	–	0.15	0.888	1867.6	14.7	4	0.24
224(1)-6	16.4	27.7	0.41	–	–	–	0.12	0.739	1341.5	11.3	3	0.23
224(1)-7	18.3	28.3	0.35	–	–	–	0.10	0.662	1461.3	10.0	3	0.20
224(1)-7b	20.8	32.2	0.35	–	–	–	0.11	0.725	1633.7	11.4	3	0.20
224(1)-7c	17.3	26.8	0.35	–	–	–	0.10	0.821	1388.0	9.4	3	0.24
224(1)-7d	19.0	29.4	0.35	–	–	–	0.10	0.741	1655.1	10.4	3	0.22
224(1)-8a	19.7	30.8	0.36	–	–	–	0.10	0.784	1801.9	11.2	3	0.18
224(1)-8b	23.4	33.3	0.30	–	–	–	0.10	0.643	1226.7	9.9	2	0.15
224(1)-10	16.1	24.9	0.35	–	–	–	0.09	0.742	1082.1	8.8	2	0.15
224(1)-10a	15.4	26.2	0.41	–	–	–	0.11	0.893	1558.2	10.9	3	0.19
224(1)-14	21.9	33.5	0.35	–	–	–	0.11	0.656	1435.4	11.7	3	0.21
224(1)-odd2	15.4	26.2	0.41	–	–	–	0.11	0.859	1686.7	10.8	3	0.22
224(1)-6a	14.2	19.6	0.28	10.5	16.0	0.34	0.04	0.697	1848.9	10.5	4	0.28
224(1)-5	11.1	21.1	0.47	2.8	12.7	0.78	0.11	0.547	1034.1	2.8	3	0.24
224(1)-8	12.8	17.6	0.27	9.6	14.4	0.33	0.05	0.792	1493.4	9.6	3	0.21
224(1)-2										9.1	3	0.23
224(1)-4										Indet.	3	0.23
224(1)-5a										Indet.	3	0.21
224(1)-7a										Indet.	4	0.29
										0.0		
	18.4	29.2	0.37	–	–	–	0.11	0.763	1511.5	10.9		
	12.7	19.4	0.34	7.6	14.4	0.48	0.07	0.679	1458.8	7.6		
								0.758	1537.5	10.7	3.1	0.22
								(0.0970)	(258.84)	(2.45)	(0.53)	(1.287)

^a^Subjective measures. Indet. = indeterminate. – = data impossible. Summary is mean, median or geometric mean as appropriate. SD in (....)

Indeed, some mites may show ‘progressive pitch‘ (i.e., a variable pitch value) along a long moveable digit (Fig. 25) and that could also be looked for in a follow-up SEM study of mites with elongate chelae (like say in other carpoglyphids or even mesostigmatid veigaids). Such ‘many possible teeth’ designs may also exist at the nano-scale level. This design may make the digit easier to start moving through the food material (i.e., having a lower inertia) while taking advantage of the momentum and power present towards the middle and end of a stroke. Of course if the function is to act on cheliceral protrusion or on cheliceral retraction then the form will be different in each case to match (see Table 20). This suggests that the blade and three-teeth form in *C. lactis* is to facilitate food processing on cheliceral protrusion i.e. it is a barbed spear/pike-like functional design (compare this to the classic flesh-slicing mesostigmatid design on cheliceral retraction in mesostigmatids Bowman 2021b). In that way the high velocity ratio (*VR*) crushing proximal teeth may also function as an attachment mechanism.

**Fig. 38:**
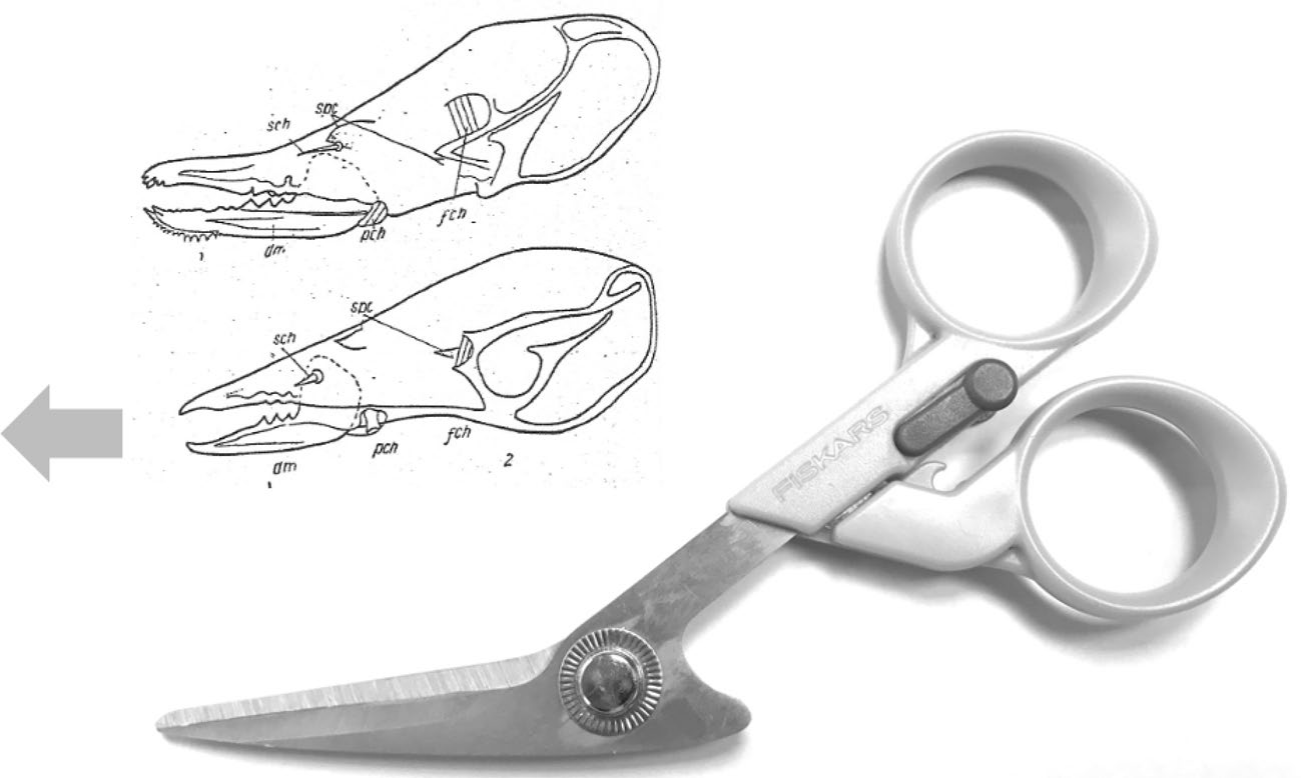
Potential seam-ripper scissor action of carpoglyphine chelae. Grey arrow direction of mite or scissors movement through ‘soft’ substrate. Large image is dressmakers scissors designed to be held vertically and orthogonally run along the table surface where the fabric is lying so as to cut open the seam stitches. Inset figure is from Akimov (1985) (including original abbreviations) with permission. Upper species = *Hericia* sp., lower species = *Carpoglyphus lactis*. Note the same three posterior teeth as in *C. lactis*. The exterior fringe to the ventral tip of moveable digit anteriorly in *Hericia* sp. should also facilitate collecting micro-material (Fashing 2008) as the semi-fluid substrate is ‘sliced’ through

Pursuing the narrow-bladed spear analogy (Fig. 29) a little further, suggests that the carpoglyphine chelicera might function like seam-ripper scissors (Fig. 38), slicing surface material as it moves forward. Indeed if the chela was tilted with respect to the horizontal axis of the mite, which then moves forward with its moveable digit dug in, one could pose a slicing action by the narrow blade, much as the how the Black skimmer (*Rynchops niger*) feeds. This bird fishes in a unique way, flying low and fast over streams (https://en.wikipedia.org/wiki/Rynchops). Their lower mandible skims or slices into the water’s surface, ready to snap shut on, and ‘collect’, any small fish unable to dart clear. Carpoglyphines may do this in oozing soft viscous exudates (‘sap flux’ or ‘slime flux’ Fashing 2008) as they (or their chelicerae) move forward snapping onto rich microbial material as they browse, which are then cracked open by the automatic occlusion of the three posterior teeth.

For sure, extreme thinness of cheliceral digits as in the algophagid *Fusohericia heliconiae* for example (see SEMs in Figs. 1–3 of Fashing and Glist 2012 would facilitate this sort of action. There, microbes in fluids may be also gathered into the distal ‘basket’ whilst other material is being sliced and cracked by the digit teeth. Of course, drawing cross-species analogies about skim-feeding can be misleading (Humphries et al. 2007), so looking for such consequential characteristic foodstuff trails when carpoglyphine mites actually feed would be useful in follow-up work. Some oblique support for the idea that elongate structures might be useful in parting through fluids to grab things is that Pfingstl et al. (2020) reports elongation of oribatid claws is a feature of those mites subject to tidal and flooding and wave action versus terrestrial (and freshwater) oribatids. Perhaps longer forms can slice through the surface tension of liquid menisci and grab the substrate material below.

**Fig. 39:**
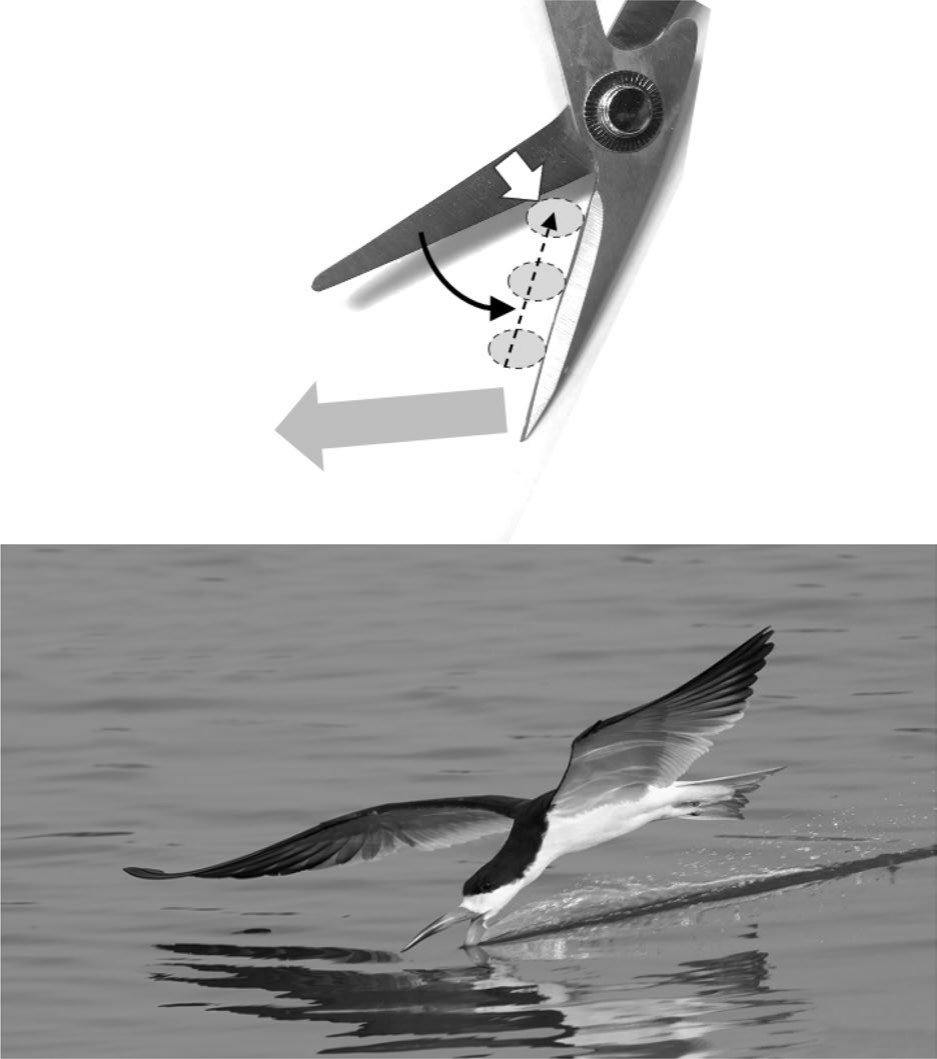
Potential skimming action of carpoglyphine chela. Upper figure with grey arrow showing direction of movement of tip of lower seam-ripper scissor blade as it engages with substrate when ‘open-mouthed’. If it encounters an object (dotted grey circle) resisting its trajectory, it drives the object into the axil (dashed arrow), where the momentum of the chela causes the upper blade to snap shut (black arrow), compressing (white block arrow) and crushing the object. J-hook designs used like this would facilitate catching even large thick-skinned objects given this scythe-like protrusion. Lower photograph Black skimmer (*Rhynchops niger*) amended from ‘Black skimmer (Rynchops niger) in flight.jpg’ taken in Pantanal, Brazil, 6 September 2015 © Charles J Sharp, www.sharpphotography.co.uk with permission under Creative Commons Attribution-Share Alike 4.0 International. Note trailing ‘sliced’ substrate

Slightly under-strengthening the digits in *C. lactis* may make them somewhat spring-like under increasing adductive muscle load, whereby the ‘tweezering‘ action of the chela when holding a hard food morsel say half way along the mastication surface allows slight bending flexibly around the latter‘s profile to facilitate grip. Such springiness may prevent fracture if any ‘skimming action’ in fluids hits a very hard object (see Fig. 39).

Deeper gullets (i.e., the depressed space in between the teeth) create a more aggressive chiselling action producing a bigger ‘chip‘ of material cut. So comparing $R_{v}$ in Tables 3, 7 and 11, shows that *G. domesticus* (but not *T. putrescentiae*) has a more aggressive action moveable digit mastication surface than *C. lactis* (if the latter species digs into food surfaces at all). Gullets clear a saw of debris so considering $\eta _{v}$ suggests that the moveable digit mastication surface of *G. domesticus* and *T. putrescentiae* may unhelpfully block with food material more than that of *C. lactis*—recall that edges below the *L*2*M* axis eject material anteriorly of the mite’s gnathosoma. Special shape gullets reduce vibration and waste heat in saw blades and might also be spotted in any follow-up SEM study of astigmatid chelal digits.

The direction of a blade’s teeth (i.e., the ‘rake‘ or hook angle, Fig. 35) determines its efficiency in dealing with different materials. Note that for the moveable digit being pushed into material, the cutting edge needs to be distal (not proximal). Rake controls the aggressiveness of a saw. Standard hook angles range from $^{+}5{-}15^{\circ }$. Steeper angles, from $18{-}22^{\circ }$, are most effective for ripping and cutting into softer materials. Positive hook angle means that the teeth lean forward (i.e., $\equiv$ towards the moveable digit tip on cheliceral protrusion or $\equiv$ towards the condyle on cheliceral retraction) and will even bite in as the whole chelicerae moves forward. Hard materials require a higher rake than softer materials (irrespective of rip or cross-cut or hybrid saw design). The higher the hook angle (i.e., to almost orthogonal to the *L*2*M* axis), the more aggressively the cheliceral blade will cut through a lot of material (especially when ripping). Low (negative) hook angles in saws i.e., those that have teeth that angle backwards ($\equiv$ almost parallel to the *L*2*M* axis backwards along a moveable digit), are good at a macro-scale for cutting materials like plastics and metal, so this may be an indicator of efficiency in mite moveable digit surfaces cross-cutting arthropod or nematode cuticle on cheliceral protrusion. Low rake saws are harder to start cutting compared to high rake saws but they cut faster. However, they are harder to control. High rake saws cut slowly but smoothly. They are easier to start and control. Rake angle is thus a key item for future morphological acarologists to measure particularly in mesostigmatids and other predators where one wants a blade that is easy to start sawing yet take advantage of the momentum and power present towards the middle and end of the moveable digit stroke action.

**Fig. 40:**
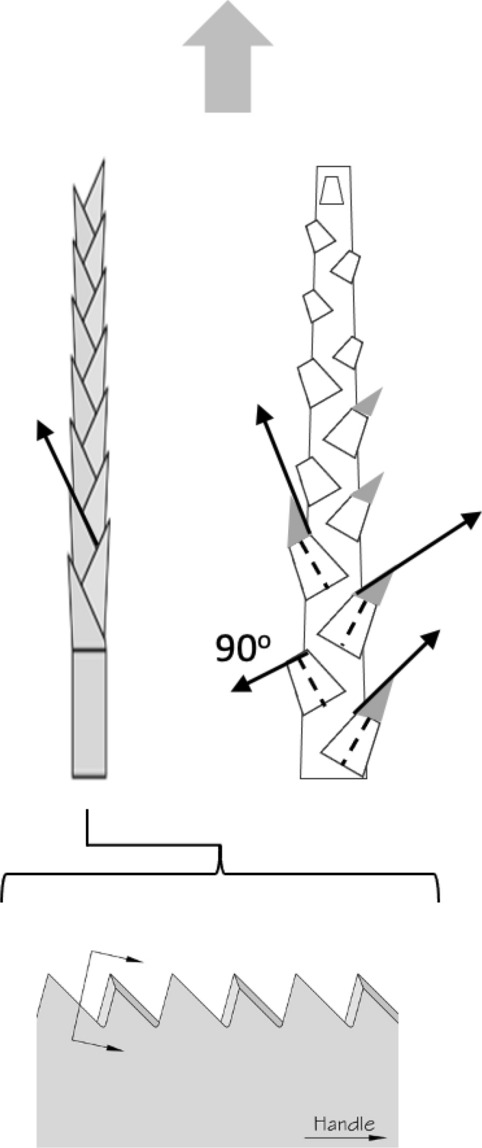
Point slope changes the operating characteristics of asperities (on being pushed into food material, grey arrow). Views from above. Left and Lower: Cross-cut saw teeth. Black arrow indicates angle with respect to the side of the base-plate. Amended from: Cross cut teeth viewed from the side and the toe © Isaac Smith 2012 with permission. Right: Astigmatid moveable digit schematic with variable point slopes (black arrows with respect to side of the equivalent base-plate given the slope of that tooth as dashed line)

**Fig. 41:**
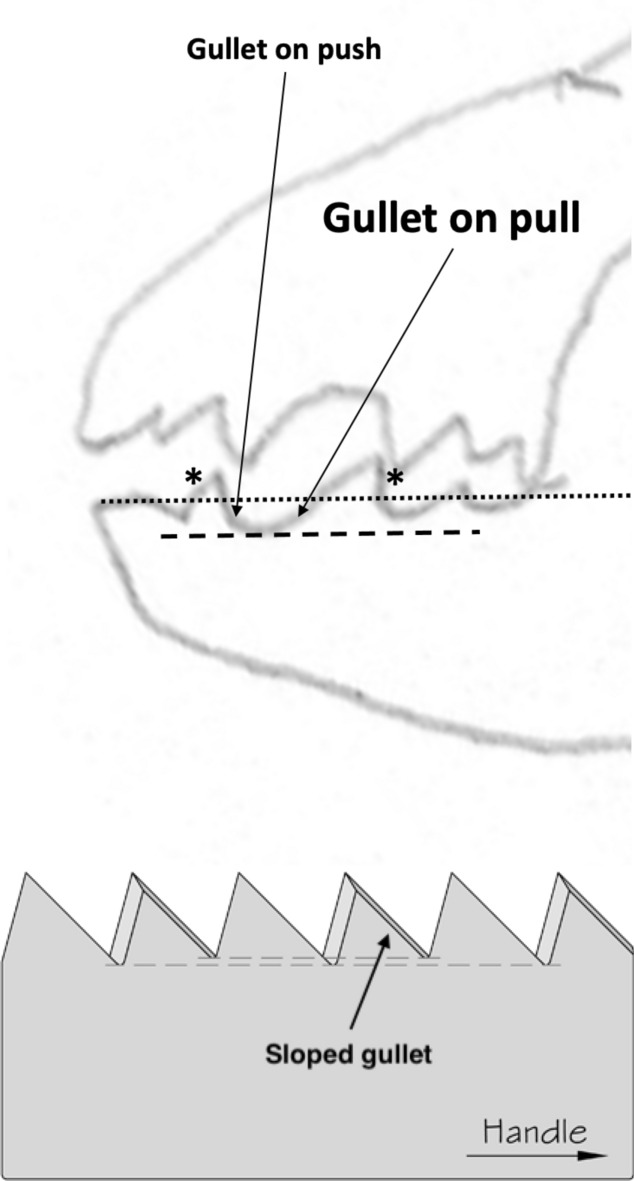
Upper.*Tyrophagus putrescentiae* (specimen 224-1-10a) showing depth of gullets on chelal moveable digit varies (highlighted by dashed line). Dotted line = tip of moveable digit to articulating condyle. *On left = leading edge of moveable digit asperity on ‘push’. *On right = leading edge of moveable digit asperity on ‘pull’. Lower. Rip saw with both point slope leading and sloped gullets trailing saw’s direction of movement ($\Rightarrow$ cross-cut saw). Note saw is pushed away from handle. Annotated from: Side view of sloped gullets, showing alternating baseline (© Isaac Smith 2012 with permission)

Point slope is the angle that the tip of a tooth creates with the side of the base plate (e.g., for an asperity with no slope, Fig. 40). It is also a by-product of adding fleam to a tooth. So a smaller more acute point slope is useful for softer material and faster cutting (but such a saw has less durable points) than a larger more acute point slope size. To complicate matters, this slope may be altered by having teeth with sloped gullets (Fig. 41). Depending upon whether the astigmatid moveable digit is designed to be pushed into or pulled out of food material to saw it, a follow-up SEM study needs to look for such slope.

The most important role that sloped gullets provide is by adding ‘slope‘ itself, in that one can strengthen the points of the teeth while maintaining the desired fleam angle, optimising a saw blade for cutting harder materials. Alternate gullets can be sloped in opposite directions. The telltale sign of sloped gullets is visible at the baseline, i.e., viewed from the side, the bottom of every other gullet is lower than its neighbours. As at least four gullets are needed to detect this, it is not clear if this is a design feature in the beehive astigmatids as the number of ‘crossings‘ is modest (Tables 3, 7 and 11). A secondary benefit of sloped gullets is the resultant increased volume of the gullet, which allows more detrital ‘dust‘ to be cleared on each stroke of the digit blade. This can be advantageous when cutting wetter material or when cutting across wide objects. Sloped gullets are found primarily on crosscut saws, but they can also be used on rip saws. Is this a design feature of predatory mites?

Gullet depth is somewhat bigger the further one is away from the moveable digit tip overall and for both types of design (A and B) for *G. domesticus* ($R^{2}=0.1655,\; 0.1533$, and 0.2087 respectively), confirming an invariably somewhat asymmetric form to its ‘pockets’. The strength ($R^{2}=0.3894$) and slope of this relationship is even larger for the type B designs in *T. putrescentiae*, which thus in comparison shows even more asymmetry commensurate with average profile in Fig. 10. However, type A designs for the acarid show no relationship with distance ($R^{2}=0.0028$), indicating little real depth differentiation in this design. Only type A designs in *C. lactis* show any relationship of gullet depth with distance from the moveable digit tip ($R^{2}=0.2198$) and the slope of this is slightly positive, i.e. gullet depth gets less, agreeing with the positioning of the three small teeth proximal to the condyle. These distinctions for example can be seen by comparing the actual mite drawings in Figs. 12, 29, and 35). Better estimating each gullet’s shape and volume together with the proportion of different size food micro-fragments within the gut in a follow-up study of these astigmatids could be most illuminating.

Breasting (Fig. 31) is a measure of the convexity of a line which as a gentle arc joins the tooth points (for teeth of a uniform size). Not all saws are breasted. In general, the heavier or coarser the work the saw is intended for, the greater the breasting (and the larger the teeth). Less breasting is associated with shorter saws. The main advantage of breasting is in keeping the teeth in better contact with the centre of the cut, resulting in smoother cutting. This is the antithesis of the case in Fig. 35. Breasting (shown by *T. putrescentiae* in Fig. 40) is aliased with developing taller teeth in the central sections of an otherwise uniform blade, since an overall bulge in the cutting surface could be by swelling the base plate carrying same size teeth instead. Astigmatid jaws being relatively short are hard to unequivocally score as breasted although the overall creep or shear values in Tables 6 and 10 do suggest regional differentiation in *G. domesticus* and (particularly) in *T. putrescentiae*.

Finally, tooth configuration on a saw can vary—common tooth types are Flat Top, Alternative Top Bevel and Combination Tooth. A saw (like a chisel) sharpened to a lower angle will penetrate material with less effort than one sharpened with a higher bevel. However, as with a chisel, an acutely filed saw tooth will be less durable.

Moreover, pitch can vary along a saw’s length. Calculating the subjective number of teeth (Tables 5, 9 and 13) divided by $x_{i_{e}}$ shows that the glycyphagid and acarid have similar pitch. The lower value for *C. lactis* is almost certainly because unlike the other two species, teeth are restricted to only a part of the moveable digit surface. If $x_{i_{e}}$ was reduced by 25% in *C. lactis* (i.e., the opportunity for any teeth to be present upon the digit surface to be the most proximal 75%) then there might be a fundamental free-living astigmatid jaw pitch of around 0.22–0.23 μm^−1^. This would imply that evolutionarily digit tip elongation in the carpoglyphid was essentially confined to the distal zone.

**Table 14:** Mean values for Laboratory ($n=20$) and Museum specimens ($n=17$)

*IL*	*CLI*	*L*2*M*	$x_{i_{e}}$	*m*	Distance from the condyle (in *L*1*U* units)	$\delta ^{\ast}$	G	$VR_{tip}$	Bite/grab max volume $M_{vG}$	*thick*	*L*1*U*	$TM_{vG}$	Estimated idiosomal	No. of bite/grabs volume	Excavation time (min)	$i_{e}$	*CHI*
Ca4																	
201.2	81.7	26.0	17.3	18.5	0.90	71	24.6	0.358	4922	9.7	9.3	2766	13,085,162	2694	45	13	32.4
G5																	
186.6	105.5	29.7	14.0	17.4	1.20	58	25.2	0.455	4860	17.2	13.5	4677	11,258,362	2322	39	9	57.8
T13																	
218.1	91.1	24.8	13.1	15.4	1.10	62	21.4	0.430	3105	12.7	10.4	2599	17139141	5750	96	10	42.6
Museum																	
182.5	86.7	22.8	10.8	12.8	1.17	58	19.3	0.461	2232	12.4	10.2	2007	10745123	4587	81	9	41.9

However, the argument above regarding the real size of the tooth row matching *Bettsia* sizes suggests that the three teeth are on a base-plate of a maximum say 7 μm length, which is only about a third of the average 20.1 μm value for $x_{i_{e}}$. If the proposal for a universal pitch across species was true then evolutionarily the teeth in *C. lactis* would have not only to have become miniaturised, but effectively translated proximally.

**Fig. 42:**
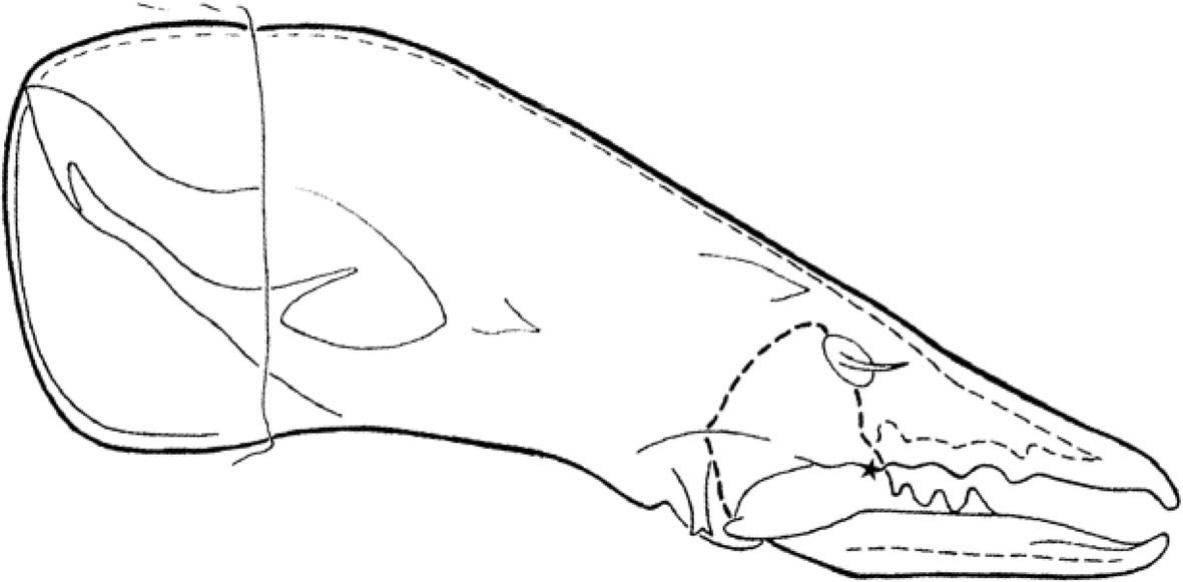
Chelicera of *Carpoglyphus lactis* from Johnston (1965) with personal permission and under ‘fair use’ (https://guides.osu.edu/copyright/copyright-exceptions). Star is the mild swelling of the ascending ramus posterior of the mastication surface (cf. fourth tooth in Fig. 35). This feature of the coronoid process (which needs an explicit developmental model) also appears to exist in *Acarus farris*, *Sancassania (Caloglyphus) berlesei*, *Chortoglyphus arcuatus* and perhaps *Lepidoglyphus destructor*. Note distal blade of moveable digit and anyerior cutting edge of first slicing tooth

Could this have been by a coronoid development process overwhelming the surface region posterior of the teeth i.e., proximally this (horizontal ramus) surface has been flexed up (as perhaps the mild proximal swelling in Fig. 23 in Johnston 1965 at the anterior of the coronoid shows, Fig. 42)? For sure, in mammals, coronoid initiation (thus its ‘starting’ location in evolution) and coronoid growth are two separate phenomena (Anthwal et al. 2015). Care in any future geometric morphometric study is thus needed.

Alternatively, perhaps a proportionate fall in teeth width as teeth heights declined during evolution in *C. lactis* exacerbated this effect allowing digit tip elongation to occur over more of the distal surface? For sure, the strong diminution of potential tooth row in the laboratory culture Ca4 fed upon yeast and wheatgerm for many years (but not for the acarid and glycyphagid, Tables 2, 6 and 10) suggests that evolutionary selection against digit elongation in this specialist is clearly possible whilst still retaining the diagnostic three proximal teeth. Whether the coronoid changed size or shape needs follow-up investigations, for sure this overall basal structure is very variable across individuals even in the wild-collected samples (Fig. 10).

Kerschbaumer and Pfingstl (2023a) found no phylogenetic signal within the claw shape trait for the closely related oribatids, so it is reasonable to conclude that ecology (i.e., different surfaces and substrates) will and has acted as one of the primary selective forces in the diversification of mite ‘attachment’ shapes. Further reducing digit elongation will consiliently increase the surface’s velocity ratio characteristics (increasing it and making it relatively more inconsistently spiky, see Table 4). Note that comparing the subjective number of teeth to the number of crossings shows no real relationship ($R^2=0.1317$ *C. lactis*, $R^2=0.0007$ *G. domesticus* and $R^2=0.0002$ *T. putrescentiae*) showing that the latter is simply detecting surface roughness at this study’s density of semi-landmarks.

The width of a saw blade (i.e., of the ‘blade plate‘) on which the digit teeth are formed also determines the size of the kerf. Saws with less set usually have taper-ground saw plates. So, analogously, the thickness of cheliceral digits has an impact upon the damage a moveable digit action can do on foodstuff (cf. impacts the morsel size). A solid reliable blade plate is one of the features of a good blade—hence the sclerotisation shown in Table 15) is important in astigmatid chelal function.

**Table 15:** Mean over astigmatid individuals for moveable digit for species in Table 1 of Bowman (2021c)

Taxon	*VR*	*E*[*VR*] $\varTheta$	*var*[*VR*] $\varPhi ^{2}$	*F*1*AV*	$VR.PHI^2$	(a)	(b)	*E*[*F*2]
A1	0.444	0.646	0.0262	1394.48	816.34	1	1	900.87
A10B	0.476	0.673	0.0229	2442.39	1641.35	2	2	1644.37
A15	0.456	0.657	0.0234	1813.96	1104.04	3	3	1192.65
A17	0.400	0.610	0.0282	1158.94	614.81	1	1	706.51
A4	0.476	0.672	0.0239	1675.21	1060.13	1	1	1125.93
AC204	0.463	0.662	0.0239	1095.50	652.81	0	0	725.68
AL2	0.538	0.721	0.0183	3403.75	2750.20	3	3	2453.33
C10	0.426	0.631	0.0274	1632.47	944.67	2	2	1030.23
C3	0.429	0.635	0.0263	2235.49	1263.11	0	0	1418.57
C5	0.427	0.633	0.0263	1718.01	1005.51	3	3	1086.82
CA4	0.355	0.568	0.0318	850.02	377.58	0	0	483.21
CH1	0.582	0.752	0.0160	2587.40	2265.30	3	3	1946.73
CV1(66)	0.442	0.645	0.0256	801.31	475.76	0	0	516.81
D3	0.568	0.740	0.0210	1479.43	1320.59	3	3	1094.61
D4	0.514	0.699	0.0257	2522.16	2180.69	3	3	1762.79
D5	0.715	0.838	0.0126	1286.81	1338.92	3	3	1078.46
F1	0.486	0.681	0.0222	1475.45	992.78	1	1	1005.09
G3	0.503	0.694	0.0216	2885.98	2153.53	2	2	2003.04
G5	0.456	0.657	0.0242	2387.13	1549.72	2	2	1569.36
G6	0.484	0.678	0.0234	3109.67	2391.44	3	3	2109.45
KL	0.465	0.665	0.0229	5447.86	3889.70	2	2	3622.41
L1	0.523	0.707	0.0224	1485.42	1004.45	0	0	1050.70
L3	0.476	0.671	0.0250	1579.71	961.21	1	1	1060.51
LA1	0.610	0.771	0.0153	4239.06	3683.25	3	3	3270.27
R1	0.461	0.662	0.0235	3151.33	2035.69	1	1	2084.89
R2	0.448	0.651	0.0249	2829.22	1736.28	3	3	1841.26
S5	0.534	0.717	0.0193	1530.96	1159.21	1	1	1098.08
T11	0.466	0.666	0.0230	1610.00	967.95	1	1	1071.98
T13	0.411	0.618	0.0288	1440.27	775.59	1	1	890.14
T17	0.416	0.623	0.0277	1454.57	777.34	1	1	906.78
T21	0.451	0.653	0.0241	1693.11	1018.09	1	1	1105.72
T32	0.460	0.660	0.0246	1641.15	1004.60	1	1	1082.50
T34	0.475	0.672	0.0228	1415.63	897.95	1	1	951.91
T38	0.487	0.681	0.0225	2379.37	1540.67	2	2	1621.13
T40	0.449	0.652	0.0244	2114.81	1308.31	1	1	1378.16
T44	0.420	0.628	0.0265	1574.16	851.42	1	1	987.88
T6	0.473	0.670	0.0234	1690.54	1056.18	1	1	1133.41
T62	0.461	0.661	0.0235	2451.10	1487.40	2	2	1620.50
T66	0.539	0.722	0.0177	3322.37	2611.66	3	3	2398.77
T7	0.431	0.636	0.0264	1679.92	936.18	3	3	1068.29
T8	0.426	0.631	0.0283	1828.11	1008.91	2	2	1153.58
T87	0.496	0.689	0.0215	1017.64	651.92	2	2	700.81
T89	0.442	0.646	0.0249	1990.43	1264.02	1	1	1285.49
T9	0.473	0.670	0.0238	1460.09	916.00	1	1	978.78
T90	0.509	0.699	0.0205	1484.49	1017.21	1	1	1037.50
TH3	0.509	0.698	0.0214	2062.27	1508.27	3	3	1439.59
TH4	0.525	0.711	0.0198	2472.79	1839.87	3	3	1757.30

*VR* = moveable digit tip velocity ratio. *E* = ‘expected’, *var* = variance, *F*1*AV* = adductive force on closing tendon, $VR.PHI^2$ = surrogate for food toughness (from Bowman 2021c), **a** sclerotisation of condyle (0 = not or feebly sclerotised, 1 = pale but sclerotised, 2 = brown moderately well sclerotised, 3 = dark brown heavily sclerotised), **b** sclerotisation of moveable digit (0 = not or feebly sclerotised, 1 = pale but sclerotised, 2 = brown moderately well sclerotised, 3 = dark brown heavily sclerotised) $E[F2]=F1AV\cdot \varTheta$

**Table 16:** Paired *z* tests for departures of observed average velocity ratio (i.e., an ornamented digit) from theoretical expected values for an equivalent beam-like digit (i.e., $O[VR]-\theta$) given that moveable digit tip velocity ratio

Taxon	Mean difference	SE of mean difference	*n*	*z*
*Carpoglyphus lactis*	− 0.006697673	0.010288993	21	− 0.650955172
*Glycyphagus domesticus*	− 0.018537303	0.011500731	12	− 1.611836921
*Tyrophagus putrescentiae*	− 0.009313935	0.01049667	17	− 0.887322841

Note possible mild evidence for the glycyphagid, however two confounding factors are in play see Fig. 13

**Table 17:** Paired *z*-tests for departures of observed average velocity ratio (i.e., an ornamented digit) from measured expected average velocity ratio for an equivalent beam-like digit (i.e., $O[VR]-\widehat{E[VR]}$) given that moveable digit tip velocity ratio

Taxon	Mean difference	SE of mean difference	*n*	*z*	
*Carpoglyphus lactis*					
Wild-collected	− 0.000615959	0.000133100	21	− 4.627804933	*
Laboratory Ca4	− 0.001084912	0.000186410	20	− 5.820035582	*
*Glycyphagus domesticus*					
Wild-collected	− 0.001277178	0.000225708	12	− 5.658546384	*
Laboratory G5	− 0.001036701	0.000137693	20	− 7.529058704	*
*Tyrophagus putrescentiae*					
Wild-collected	− 0.000795285	8.63657E−05	17	− 9.208339941	*
Laboratory T13	− 0.001684591	0.000221299	20	− 7.612296491	*
Museum	− 0.001091796	0.000263828	17	− 4.138288707	*

*$p<0.05$. See Fig. 16 for between the species comparison. Significant evidence is shown using this approach (based upon the small negative velocity ratio difference between actual hypotenuses to horizontal projections onto *L*2*M*) even for the apparently bar-like *C. lactis*. The statistic is negative as *per force* *O*[*VR*] has a longer $tooth_{i}$ hypotenuse than $L2M_{i}$ so the velocity ratio of teeth or gullets is less. This test allows for discretion bias and for variation in the position of the ascending ramus

**Table 18:** Paired *z* tests for departures of measured expected average velocity ratio for a ‘no asperities/gullets‘ bar-like digit versus theoretical expected values (i.e., $\widehat{E[VR]}-\varTheta$) given that moveable digit tip velocity ratio

Taxon	Mean difference	SE of mean difference	*n*	*z*	
*Carpoglyphus lactis*	− 0.006081714	0.010291647	21	− 0.590936894	
*Glycyphagus domesticus*	0.104648762	0.024907394	12	4.201513881	*
*Tyrophagus putrescentiae*	0.139913633	0.021037379	17	6.650715991	*

*$p<0.05$. Note the strong evidence for the glycyphagid and acarid where their $\widehat{E[VR]}$ values are comparatively too big i.e., they contain more high velocity ratio values as the position of maximum jerk is more posterior (its distance from the condyle on average is too low in these individuals compared to the integration within $\varTheta$ being over $L2M{-}L1U$). $\widehat{E[VR]}$ is over the mastication surface $x_{i_{e}}$ which is not quite the same limit of integration since *L*1*U* only approximates where the maximum jerk position (see Tables 3,7, and 11). Discretisation bias is not allowed for in $\varTheta$ for the concave nature of the relationships

**Table 19:** Gape angle ($\delta$) by taxon calculated from average values in Table 2 of Bowman 2021c (using Pythagoras Theorem and The Law of Cosines) together with average angle of lyrifissure to *L*2*M* axis (LYR)

Taxon	Code	$\delta$ ($^{\circ }$)	Angle to LYR (°)	Angle with *L* (°)	Angle at tip (°)
*Acarus chaetoxysilos*	AC204	62	56	86	38
*Acarus farris*	A17	66	56	88	36
*Acarus gracilis*	A4	62	61	77	42
*Acarus immobilis*	A1	64	55	89	36
*Acarus siro* [‘H‘]	A10B	62	62 $\dag$	78	39
*Acarus siro* [SW sp.]	A15	63	56	85	39
*Aleuroglyphus ovatus*	AL2	57	54	78	49
*Sancassania berlesei*	C3	65	67	79	34
*Cosmoglyphus oudemansi*	C10	65	64	80	36
*Cosmoglyphus hughesae*	C5	65	63	80	37
*Kuzinia laevis*	KL	62	46 $\ddag$	88	46
*Lardoglyphus konoi*	L1	58	72	66	42
*Lardoglyphus zacheri*	L3	62	$83^{\max}$	$64^{\min}$	33
*Neosuidasia* sp	LA1	52	51	73	56
*Madaglyphus legendrei*	T34	62	61	81	38
*Rhizoglyphus echinopus*	R1	63	63	79	39
*Rhizoglyphus echinopus*	R2	63	67	77	37
*Suidasia pontifica*	S5	58	66	66	48
*Thyreophagus* sp	TH4	58	57	78	45
*Thyreophagus entomophagus*	TH3	59	62	74	44
*Tyroborus lini*	T66	57	54	88	38
*Tyrolichus casei*	T62	63	65	77	38
*Tyrophagus brevicrinatus*	T89	64	62	78	40
*Tyrophagus longior*	T40	63	56	84	40
*Tyrophagus nieswanderi*	T6	62	63	83	35
*Tyrophagus palmarum* [‘A‘]	T17	65	60	85	35
*Tyrophagus palmarum* [‘B‘]	T32	63	62	82	36
*Tyrophagus vanheuri*	T7	64	53	80	47
*Tyrophagus perniciosus* [‘A‘]	T8	65	60	84	35
*Tyrophagus perniciosus* [‘B‘]	T38	61	68	73	39
*Tyrophagus putrescentiae* [‘A‘]	T13	66	59	84	38
*Tyrophagus putrescentiae* [‘B‘]	T9	62	62	78	40
*Tyrophagus robertsonae*	T87	60	58	85	37
*Tyrophagus similis* [‘A‘]	T44	65	59	80	40
*Tyrophagus similis* [‘B‘]	T21	63	65	82	33
*Tyrophagus savasi*	T11	62	58	84	38
*Tyrophagus tropicus*	T90	59	57	83	39
*Carpoglyphus lactis*	CA4	$69^{\max}$	59	$96^{\max}$	$25^{\min}$
*Chortoglyphus arcuatus*	CH1	54	50$\dag \dag$	73	57
*Glycycometus hughesae*	G3	60	45	87	48
*Lepidoglyphus destructor*	G6	61	37^min^	82	61
*Glycyphagus domesticus*	G5	63	41	91	48
*Dermatophagoides farinae*	D4	59	41	75	64
*Dermatophagoides microceras*	D5	$44^{\min}$	37	74	$69^{\max}$
*Dermatophagoides pteronyssinus*	D3	55	39	73	68
“Winterschmidtiidae“ sp	CV1(66)	64	43	90	47
*Forcellinia galleriella*	F1	61	53	86	42
Average		61	57	80	42

Minima and maxima marked for each column. Taxon order as in Bowman (2021c). ${\dag}\approx$ 62° in Akimov and Gaichenko (1976). ${\dag\dag}\approx$ 65° in Akimov and Gaichenko (1976). ${\ddag}\approx$ 50° in Akimov and Gaichenko (1976)

It is a trivial assertion that larger asperities (indicated by larger $R_{p}$) should be associated with strengthening to sustain greater loads. Crunch forces on the maximum peak $R_{p}[F2]$ (calculated by $F1 \times R_{p}[VR]$ in Tables 5, 9 and 13) can be substantial (approximating the digit tip forces of oribatids in *G. domesticus*). So the sclerotisation in *G. domesticus* G5 is expected given its larger tip crunch force *F*2 (Bowman 2021c). For a saw blade to operate smoothly and to make a true cut without a lot of scoring on the edge of the cut, the blade plate has also to be substantial enough to absorb vibration and to handle the waste heat generated during the cut. Overly thin moveable digits (e.g., *Hericia* Fashing 2008, *Fusohericia* Fashing and Glist 2012) would thus be prone to wobble (yet still be suitable for working in liquids). A saw blade’s teeth, of course, would normally have to make a wide enough cut to allow the blade plate to pass through the kerf. Excessive force (i.e., *F*2) would be needed otherwise to overcome any excessive friction when plunged into solid material. One predicts therefore that for a chela with appreciable set, the (cross-section of the) cheliceral digit itself will not be ‘taper ground‘. This could be verified in a follow-up study.

The introduction of this review introduced the idea that teeth isometry across astigmatids could be a key criterion across species. Values for $1000.\frac{R_{t}}{IL}$ are shown in Tables 4, 8 and 12. On the face of isometry is seemingly rejected (at least between *C. lactis* and the other two species). However, it is now clear from this review, that acarine dentition patterns are an adapted module within an adapted chelal module (Bowman 2021c, 2023a) within an adapted cheliceral/gnathosomal module (Bowman 2021b, 2023b). Moreover, although these can be embedded into a rational scheme of life style, each element is not necessarily strongly correlated, so the appropriate divisor to definitively scale $R_{t}$ is not clear.

### Final thoughts for the future

**Fig. 43:**
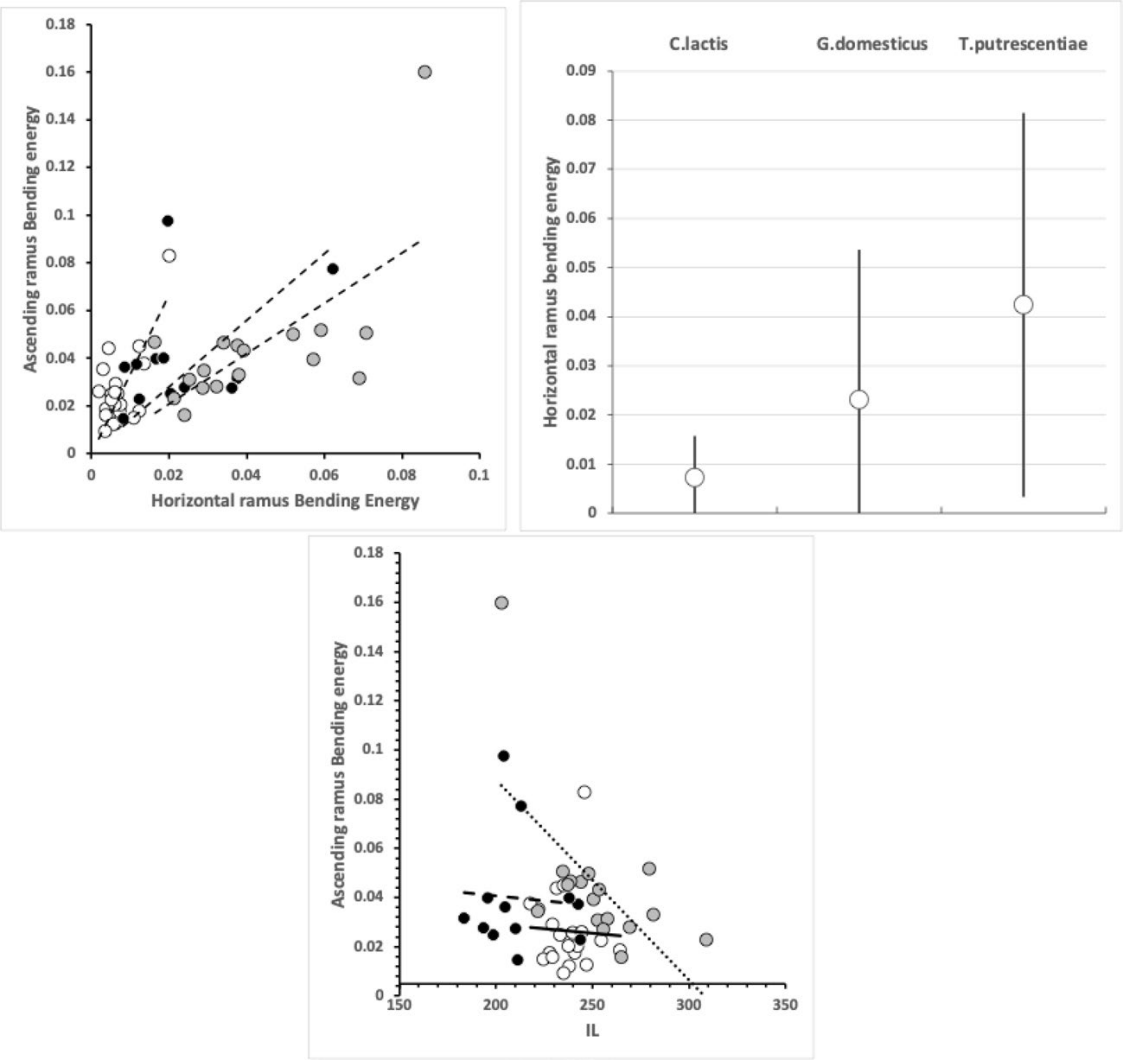
Estimated bending energy (${\widehat{BE}}$) shows two different processes during moveable digit evolution in UK beehive species. Upper left. Plot of bending energy for horizontal ramus carrying teeth and gullets ($\widehat{BE_{hr}}$ over tip to end of mastication surface at $x_{i_{e}}$) versus bending energy for ascending ramus ($\widehat{BE_{ar}}$ over end of mastication surface at $x_{i_{e}}$ to semi-landmark 16). Open circles = *Carpoglyphus lactis*. Grey circles = *Tyrophagus putrescentiae*. Black circles =*Glycyphagus domesticus*. Dashed linear regression lines through zero showing modest relationship. Upper right. Mean and 95% confidence intervals for $\widehat{BE_{hr}}$ for each species. That of *Tyrophagus putrescentiae* would be regarded as significantly different from zero. Lower. Plot of ascending ramus bending energy versus body size (*IL*) with indicative linear regression lines within each species. Note consilient increase in bending energy with smaller size. Open circles and solid line = *Carpoglyphus lactis*. Grey circles and dotted line = *Tyrophagus putrescentiae*. Black circles and dashed line = *Glycyphagus domesticus*

**Fig. 44:**
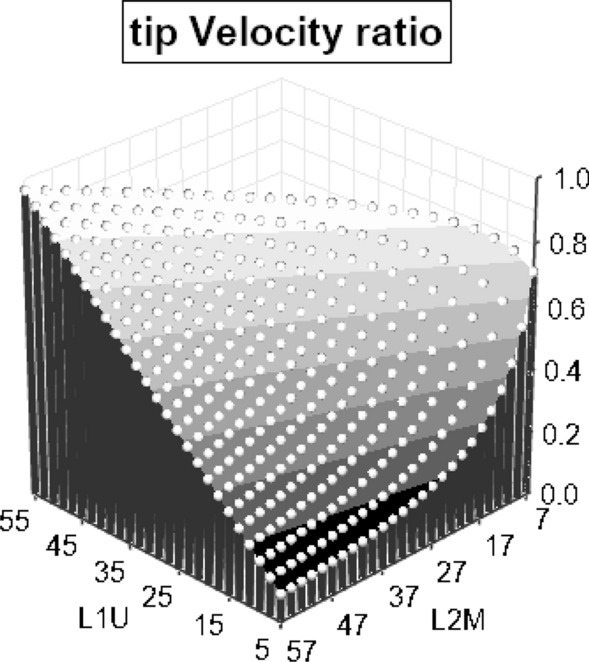
Velocity ratio of the moveable digit tip (i.e., $VR=\frac{L1U}{L2M}$) surface plot for varying sizes of *L*1*U* and *L*2*M* taken from minima and maxima over the 47 astigmatid species in Bowman (2021c). Digit measurement axes are same size to avoid visual distortions. Grid points as white circles. Grayscale contours indicate VR

**Fig. 46:**
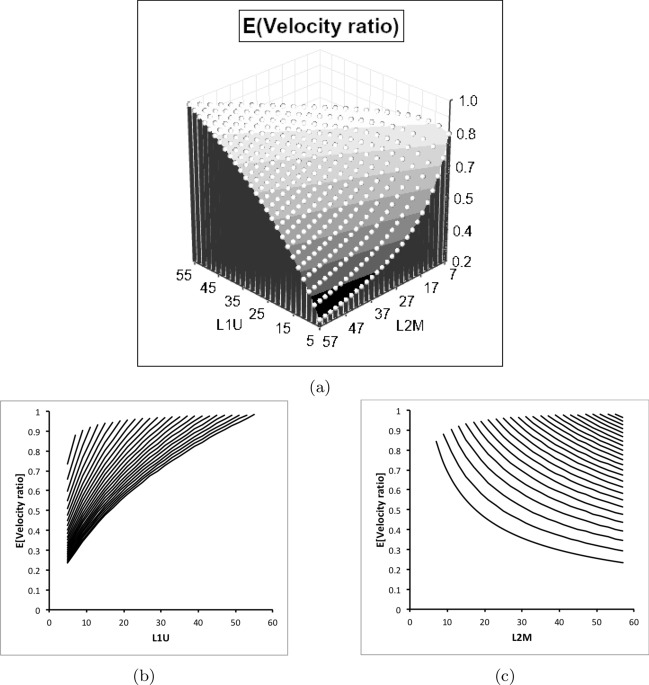
Theoretical velocity ratio of the mastication surface (i.e., *E*[*VR*]) plots for varying sizes of *L*1*U* and *L*2*M* taken from minima and maxima over the 47 astigmatid species in Bowman (2021c). Digit measurement axes are same size to avoid visual distortions. **a** Surface plot. Grid points as white circles. Grayscale contours indicate *E*[*VR*]. **b** Lines for fixed $L2M>L1U$, **c** Lines for fixed $L1U<L2M$

What might be the best way forward for morphologists to understand astigmatid digit changes? Bending energy values (*BE*) for the moveable digit surface in the three UK beehive species are shown in (Table 21). These reveal (Fig. 43 Upper left) that two different morphological processes during evolution in the UK beehive species are at play. The horizontal *x*-axis measures increasing toothiness (and increasing anisotropy of those teeth). The vertical *y*-axis appears to indicate an adaptation to deliver and sustain more powerful forces across species. The bending energy of an object is dependent upon its overall size. Smaller objects have higher energies consilient with the physical intuition that it takes more energy (i.e., work) for example to bend a one cm piece of thin metal into a circle than it does to bend a 100 cm piece into a circle. Figure 43 lower confirms this effect for mite moveable digit surfaces. If necessary the bending energy of the ascending ramus could perhaps be used to build a dynamic model (like in Moulton et al. 2023). The three species rank accordingly (Fig. 43 Upper right) as a collector, a sawing shredder and a scraping gleaner.

**Table 20:** Location of variable pitch depends upon whether the sawing action of the moveable digit toothed blade is designed to be on cheliceral protrusion or on retraction

Fewer points per μm	More points per μm
ON PROTRUSION	
On distal m.d. tip area	On proximal mastication area
ON RETRACTION	
On proximal mastication area	On distal m.d. tip area

Proximal means near to condyle

**Table 21:** Estimated bending energy for the moveable digit horizontal ramus ($\widehat{BE_{hr}}$) and the ascending ramus ($\widehat{BE_{ar}}$) by specimen and UK beehive species (see Fig. 5)

Specimen	$\widehat{BE_{hr}}$	$\widehat{BE_{ar}}$
*Carpoglyphus lactis*		
224(1)-1	0.0073	0.0176
224(1)-5	0.0038	0.0185
224(1)-6	0.0123	0.0176
224(1)-6a	0.0031	0.0353
224(1)-6b	0.0069	0.0250
224(1)-6c	0.0076	0.0204
224(1)-10	0.0063	0.0291
224(1)-11	0.0063	0.0126
224(1)-11a	0.0046	0.0440
224(1)-11b	0.0200	0.0828
224(1)-11c	0.0051	0.0247
224(1)-12	0.0061	0.0203
224(1)-12a	0.0053	0.0226
224(1)-13	0.0110	0.0149
224(1)-13a	0.0038	0.0159
224(1)-15	0.0061	0.0257
224(1)-15a	0.0058	0.0121
224(1)-15b	0.0036	0.0092
224(1)-16	0.0019	0.0260
224(1)-18	0.0124	0.0450
224(1)-19	0.0137	0.0377
Summary	0.0073	0.0265
SD	0.00434	0.01628
*Glycyphagus domesticus*		
224(2)-1	0.0123	0.0228
224(2)-2	0.0197	0.0975
224(2)-3	0.0117	0.0374
224(2)-4	0.0166	0.0398
224(2)-5	0.0377	0.0316
224(2)-6	0.0186	0.0398
224(2)-7	0.0623	0.0773
224(2)-8	0.0204	0.0250
224(2)-9	0.0086	0.0362
224(2)-9a	0.0083	0.0147
224(1)-10	0.0241	0.0277
224(1)-20	0.0362	0.0273
Summary	0.0230	0.0398
SD	0.01560	0.02387
*Tyrophagus putrescentiae*		
224(1)-2	0.0859	0.1598
224(1)-3	0.0291	0.0346
224(1)-4	0.0253	0.0308
224(1)-5a	0.0592	0.0517
224(1)-6	0.0341	0.0463
224(1)-6a	0.0380	0.0331
224(1)-7	0.0572	0.0393
224(1)-7a	0.0163	0.0468
224(1)-7b	0.0213	0.0229
224(1)-7c	0.0376	0.0453
224(1)-7d	0.0690	0.0315
224(1)-8a	0.0323	0.0279
224(1)-8b	0.0288	0.0273
224(1)-10	0.0708	0.0505
224(1)-10a	0.0521	0.0499
224(1)-14	0.0240	0.0160
224(1)-odd2	0.0393	0.0433
Summary	0.0424	0.0445
SD	0.01991	0.03152

Bending energy for the horizontal ramus carrying teeth and gullets is over the digit tip to the end of mastication surface at $x_{i_{e}}$. Bending energy for the ascending ramus is over the end of mastication surface at $x_{i_{e}}$ to semi-landmark 16. Summary is Mean and SD

Embracing the ‘discover, determine, design‘ approach of Gebeshuber and Majlis (2011) by synthesising the above into an hypothetical example mite, that might be selected for in evolutionary time suggests: (1) If you want an astigmatid polydont moveable digit surface to cut tough material—then have large sclerotised asperities. (2) If you want it to cut fast—then reduce the rake of the teeth. (3) If you want it to start cutting easily (yet still cut fast)—then increase the fleam a bit to make the teeth slice more ($\equiv$ to skewing a hand-plane). This also has the effect of making a smoother cut surface in the food material. You can also reduce the set to create a cleaner cut since a more uniform tooth line will not present as jagged an edge to the foodstuff. This in turn allows you to reduce the fleam to make a stronger tooth. The beauty of this is that by altering all three of these in concert you can produce the ‘perfect‘ tool for that astigmatid’s life-style need. Each design element plays a role. Tooth geometry should rely on all three to do the job.

A SEM follow-up study could highlight which astigmatid moveable digit features operate in which of these ways (in three dimensions) for various species in relation to their known food habits (Bowman 2021c). In particular, how the digits might be cleaned of any accumulating residue on their blades during repeated use (not just how the rutella chop excess food off) could be investigated. Mechanical saws are usually halted and cleaned of ‘blade drag‘ residue with solvents—is there a post-prandial potential liquid supply mechanism in astigmatids? Is that what the supracoxal gland is supplying? Is there any relationship to supracoxal setal form? Careful SEM techniques will be needed (Murillo et al. 2013). Understanding the time course of gland action with respect to feeding will be key. A chela with low maximum asperities ($R_{p}<1$ μm) and/or designed for soft food (i.e., having a low crunch force *F*2) *per force* does not need strong cleaning, so its gullets do not need to be large ($abs(R_{v})<1$ μm). Micro-roughness of a surface affects its ‘wettability‘ (Bowman 2023b). How sticky is astigmatid food material in the field? If this foodstuff easily clogs chitinous surfaces, then is feeding upon such correlated with more digit lubrication by the astigmatid or the possession of nano-size surface structures? Are there signs of a particular feeding action in any characteristic damage to food stuffs by each species visible with scanning electron microscopy? There is much for a keen acarologist to investigate.

## Conclusion

The expected chelal velocity ratio over its mastication surface for an individual mite as a summary of masticatory efficiency better represents differential adaptations than digit tip estimates. A clear shift from just considering astigmatid chelae in investigations to be a simple crushing appendage, towards understanding them as a species-specific composite tool, like a saw, flow from the results of this review.

Using wild collected specimens when looking at fine trophic detail is important. Astigmatid cheliceral chelal moveable digits do vary in a rational way. Teeth (and gullets) matter. All three species are designed like mammalian herbivores (i.e., *C. lactis* despite its gnathosomal elongation is not designed like the ‘nibbling’ convergent regime 1 of non-avian dinosaurs). As such the general comparative biomechanical design rules (for temporalis muscle functioning) in mammals Morales-García et al. 2021, already used by Bowman (2021b, 2021c, 2023a) apply.

Fashing (1998, 2008) was right in principle. This study outlines that astigmatids can be categorised as:

- demolishing ‘shredders‘ who ingest leaf-derived and woody material and associated microbes by biting off chunks of material (to saw up), versus
- browsing ‘scrapers‘ (grazers) who crop fungal hyphae and/or other microbes and shear off detritus from the substrate surface, versus
- ‘collectors‘ who pick, filter, accumulate and crack microbes and fine particulate matter from ‘speared‘/sliced substrates and ‘skimmed’ aquatic films.

However, moveable digit design does not grade in the saprophagous astigmatids co-occurring in UK beehives. *Tyrophagus putrescentiae* does not appear to be an intermediately designed trophic form between *Carpoglyphus lactis* and *Glycyphagus domesticus*.

The three co-occurring species in UK beehives are trophically distinct at all morphological levels in different ways. Immatures of each species need to be examined to look for evidence of within species trophic separation. Further wild-collected samples should confirm the likely scales of variation within and between mites and thus better define the critical boundaries of the changes in digit patterns. More species need to be examined before whether the ‘back teeth‘ of moveable digits can be used to predict diet as it is for molar1 teeth in mammals (Grossnickle 2020). Follow-up biological work to show that food material selection is a rational size-dependent behaviour arising from the clear dental differentiation between the three species (as found in crabs Aronhime and Brown 2009) would validate these morphology-based assertions.

## Figures and Tables

**Fig. 45:**
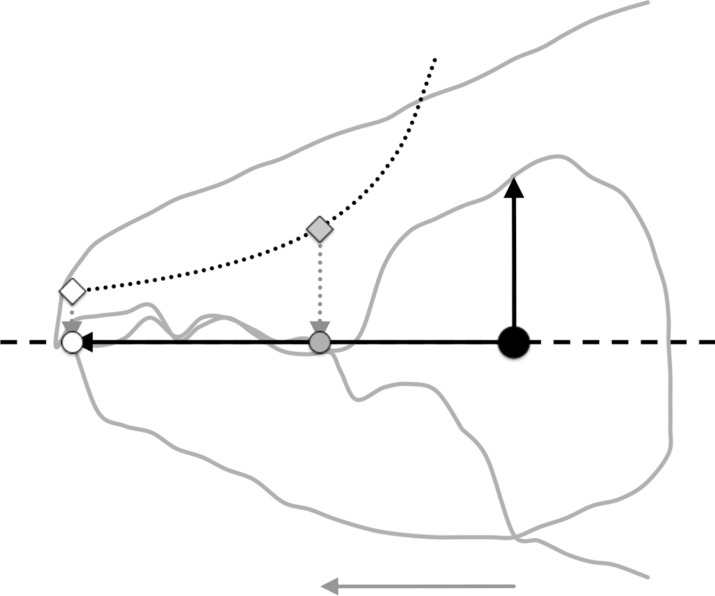
Average velocity ratio (*E*[*VR*]) over mastication surface is the (rescaled) integral under the velocity ratio curve function (dotted line) for that *L*1*U* value (vertical arrow) and that *L*2*M* value over the range from the large black circle condyle to small black circle moveable digit tip along the dashed *L*2*M* axis, divided by [$L2M{-}L1U$] (the distance between the grey circle and the white circle). Diamonds show location of arrowed vertical drops at the integration limits of the line function (clear diamond = moveable digit tip, grey diamond = at *L*1*U* from the condyle. Larger female *Tyrolichus casei* (Oudemans) chelicera for clarity. Large grey arrow below chela = length of adductive lever moment arm *L*1*U* (see Bowman 2021c) forwards from condyle in *L*2*M* direction

**Fig. 47:**
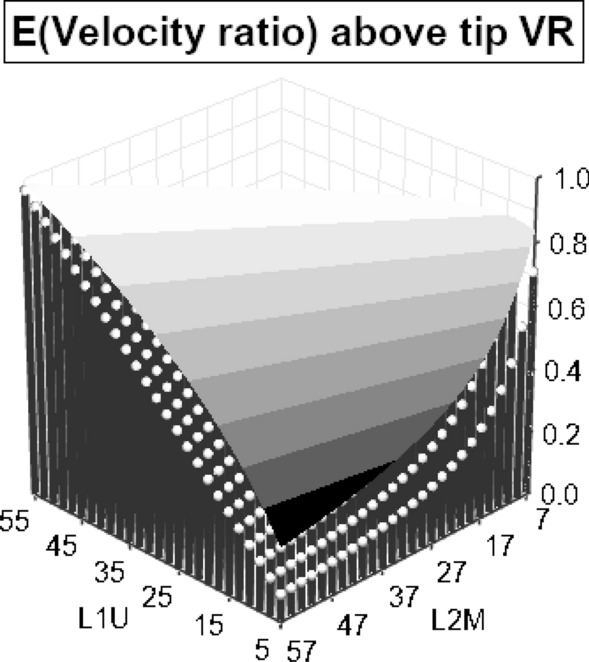
Expected velocity ratio of the mastication surface (i.e., theoretical *E*[*VR*]) surface contour plot overlain upon moveable digit tip velocity ratio for varying sizes of *L*1*U* and *L*2*M* taken from minima and maxima over the 47 astigmatid species in Bowman (2021c). Digit measurement axes are same size to avoid visual distortions. Tip velocity ratio grid points as white circles. Grayscale contours indicate *E*[*VR*]

**Fig. 48:**
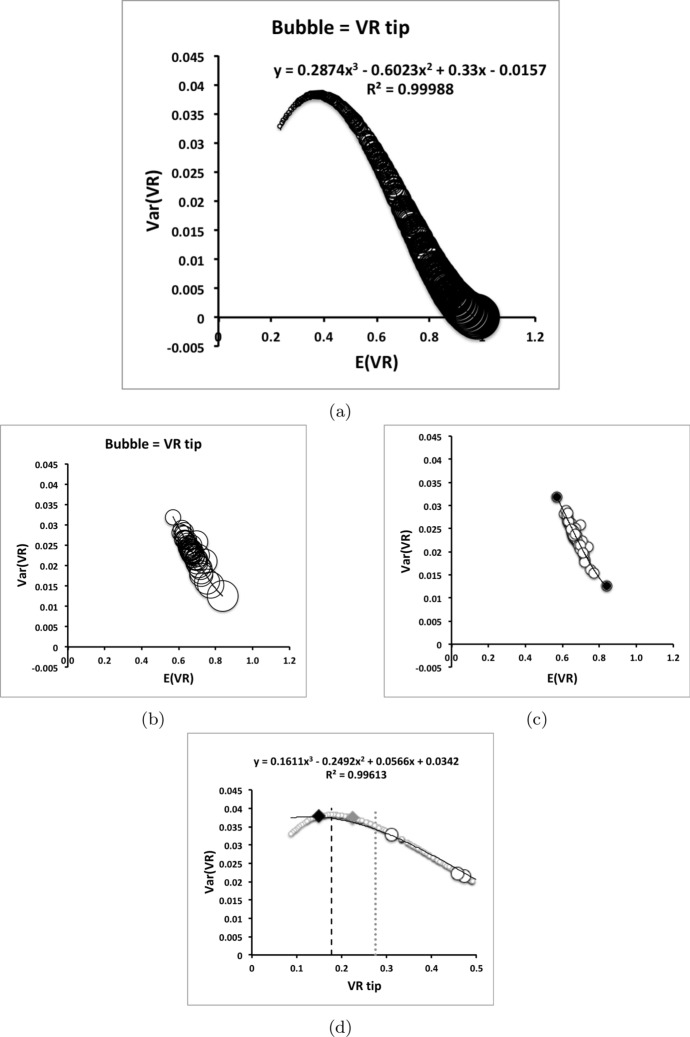
Theory matches observations. **a** Relationship between variance of theoretical velocity ratio over mastication surface versus expected velocity ratio over the same range of mastication surface approximates a cubic, first rising from elongate low tip velocity ratio chelae, then falling as *L*1*U* dominates over *L*2*M*. **b** Results for 47 species in Bowman (2021c) with cubic trend. **c** Same plot of results for 47 taxa in Bowman (2021c) (with cubic trend) now showing Ca4 *Carpoglyphus lactis* as upper black circle and durophage D5 *Dermatophagoides microceras* as lower black circle. **d** Numerical solution of maximum *Var*[*VR*] ($\varPhi ^2$) versus chelal moveable digit tip velocity ratio. Dashed line at $VR_{tip}\approx 0.178$ maximum, note cubic fit is relatively poor over this zoomed up region. Large circles are experimental samples for (left to right): *C. lactis*, *G. domesticus*, *T. putrescentiae*. Black diamond = *Veigaia cervus*, grey diamond = *Veigaia nemorensis*(old) and dotted line at $VR_{tip}=0.276$ threshold between cutting killing-style and crushing killing-style designed chela from Bowman (2021b). The hypocarnivorous *C. lactis* approximates a specialist predator’s operating characteristics

## Explanatory appendix

### Velocity ratio

Bowman (2021b, c) uses the moveable digit tip velocity ratio ($VR=\frac{L1U}{L2M}$, Fig. 44) of the astigmatid chela as a part summary when investigating overall cheliceral design in mites (Fig. 2), treating the tip of the moveable digit as analogous to the end of a swinging simple bar or beam of uniform density. In a frictionless system, the mechanical advantage and the velocity ratio are equivalent. In this review herein the latter term is used. *L*1*U* is the adductive input lever moment arm, *L*2*M* is the adductive output lever arm. The velocity ratio acts like a force multiplier of the muscular pull on the adductive tendon within the cheliceral shaft (i.e., force *F*1) closing the moveable digit tip against the fixed digit tip (with a force $F2=VR \cdot F1$). As a jaw, astigmatid chelae have a scissor-like straight up-and-down action much as in herbivorous rhynchosaurs (Benton 1983). They do not grind laterally like in ungulates. Jaws with a small mechanical advantage (i.e., low *L*1*U*) are adapted to produce rapid but relatively weak movements, whereas those with high mechanical advantage (i.e., high *L*1*U*) produce slow but strong movements (Smith and Savage 1959).

Bowman (2021c) found that all free-living astigmatids studied have moveable digit tip velocity ratios of relatively high leverage similar to crushing-style mesostigmatid hypocarnivores, cryptodire turtles (Dalrymple 1979b), microphytophagous oribatids, or some macrophytophagous oribatids. For fixed *L*2*M* (i.e., broadly speaking for a constant length of the moveable digit), the velocity ratio at the digit tip declines linearly with decreasing *L*1*U* (left vertical flank in Fig. 44). That is, increasing the input moment lever arm just produces proportional gains. However, for a fixed *L*1*U*, the velocity ratio at the digit tip declines not linearly but hyperbolically with increasing *L*2*M* (right vertical flank in Fig. 44). Under these conditions, a specified increment in *L*2*M* when *L*2*M* is large i.e., for an already elongate moveable digit, has less impact on the tip velocity ratio (*VR*) than when *L*2*M* is originally small. Any evolutionary ‘penalty’ of digit elongation is thus less, leading perhaps to accelerated or ‘run-away’ morphological processes as seen in spider and beetle mandibles (and perhaps the chelicerae of *Veigaia* spp.; Bowman 2021b). This may also explain the tweezer-like form of the chela in *Carpoglyphus lactis*. If a moveable digit swells in size such that always $L1U\rightarrow b.L1U$ and $L2M\rightarrow b.L2M$ (i.e., linear growth or proportional conditions), then the tip velocity ratio does not change.

### Mastication surface

Of course the above bar-like form in the mechanical model is a simplification, as the moveable digit of astigmatids *does* have teeth along it (i.e., it does not have just effectively one ‘incisor/canine tooth‘ = the digit tip). Each of these features will have a different crunch force (Fig. 2). Misalignment of these with fixed digit teeth will form a couple tending to rotate food material within the chelal gape (Gans 1974).

The chelal mastication surface is defined as that part of the upper region of the moveable digit able to grasp food material in some way (thus pressing it against the fixed digit). It stops at the rise of the basal ‘coronoid’ process on the moveable digit. This, like in other mites, is essentially at a distance from the condyle along the *L*2*M* axis approximately equivalent to the size of *L*1*U* e.g., for mesostigmatids in Buryn and Brandl (1992) with a *L*1*U* value comparable to astigmatids (i.e., < 27 μm), the value of [*L2M – Digitus mobilis length of tooth row*] from their study is on average = 99.4% of *L*1*U*. In other words, the ‘coronoid’ or ‘basal ramus’ begins to rise at a location where the moveable digit velocity ratio = 1. This is appropriate as for a Class I lever to be considered in mechanical equilibrium requires a bite force to be less than the applied force, i.e., the ‘masticatory machine’ should have a mechanical advantage < 1.0 (Smith 1978).

A question arises as to how this mastication surface is formed? A variety of summaries of the toothed, gulleted and bladed mastication surface of the moveable digit of the astigmatid cheliceral chela will be used in this review depending upon assumptions as to how the digit might be deformed in evolutionary development. That is, the chelal digit surface when looked at laterally in two dimensions might be morphologically: stretched vertically (Fig. 6), flexed or bent up-and-down by local rotations (Fig. 7), or suffer creep or shearing (Fig. 8), at any one point along it. These are convenient artifices. Like thin-plate splines (Bookstein 2018) they are free of the actual cellular mechanism and differential tissue growth changes carried out to achieve them. Of course, each [*x*, *y*] co-ordinate location on the moveable digit mastication surface, with respect to the condyle-to-tip *L*2*M* axis, has its own matching velocity ratio suitable for different functional uses. As a first summary approximation, before trying to ascertain homologous landmarks along the chela across species for geometric morphometrics (Bookstein 2018), one can summarise over the whole mastication surface an average or expected velocity ratio (denoted *E*[*VR*] or $\varTheta$) again assuming the moveable digit to be a simple bar as follows.

### Average velocity ratio

Let us take the moveable digit to be a simple solid bar i.e., ignoring any asperities/gullets etc., for now (i.e., effectively setting all $y=0$) and define *a* (the *x* coordinate) to be along the *L*2*M* axis from the condyle (just as in Akimov and Gaichenko 1976). Now, each [*x*] co-ordinate location along the moveable digit mastication surface (for that example value of *L*2*M*) has its own matching velocity ratio (and thus chelal crunch force equivalent) along the bar. This is like the illustrated forces $Fb_{t1}, Fb_{t2}$ and $Fb_{t3}$ in Fig. 4 of Heethoff and Koerner (2007).

Then, the theoretical average or expected velocity ratio value ($\varTheta$) over the whole mastication surface, as a beam (with $L2M>L1U$), from the rise of the moveable digit basal process (i.e., at $x=L1U$) through to the moveable digit tip with respect to that *L*2*M* axis (*a*) is:

$$\begin{aligned} \varTheta =\tfrac{1}{(L2M{-}L1U)}\cdot {\mathop {\int }\limits _{L1U}^{L2M}}VR_{a}\text {d}a \end{aligned}$$

This definite integral is illustrated on a typical chela in Fig. 45. Here this is integration of a fully deterministic hyperbolic valued function not an expectation over sampled values each of which belongs to a probability distribution from different individuals. It can be evaluated by substitution into the formula

$$\begin{aligned} \frac{a}{U-L}\cdot log_{e}\frac{U}{L} \end{aligned}$$

from the Technical Appendix, to give

1 $$\begin{aligned} \varTheta = \frac{L1U}{(L2M{-}L1U)}\cdot log_{e}\frac{L2M}{L1U} \end{aligned}$$

$\varTheta$ (= the theoretical *E*[*VR*] for an un-ornamented bar-like moveable digit) is illustrated in Fig. 46. $\varTheta$ is thus the velocity ratio of a simple bar-like lever with the rotation point now effectively positioned at the posterior end of the moveable digit mastication surface multiplied by the natural logarithm of one over the *original* velocity ratio of the moveable digit tip. Note that the term ‘expected mechanical advantage’ does not appear to be used by workers. The expected velocity ratio has a different meaning in the field of gear ratios (see https://www.cs.cmu.edu/~rapidproto/mechanisms/chpt7.html. Note herein that $L2M{-}L1U$ in the first term above alternatively could be considered as the length of the teeth (and gullet) row on the moveable digit (cf. parameter 10 in Buryn and Brandl 1992, or morphometric 13 used in Liu et al. 2017 for mesostigmatids). However neither of these latter measures cover the whole potential mastication surface but simply that area where teeth can be subjectively observed (see their Figures). Accordingly it is difficult to make comparative cross-checks between studies.

The algebra infers trivially that the expected velocity ratio of the mastication surface is always above that of the digit tip (Fig. 47).

By virtue of including velocity ratio values posterior of the moveable digit tip, it is always true that $\varTheta > VR_{tip}$ and it is a more representative measure of the utility of the whole digit as a tool than just considering solely the mechanics of its tip. An assumption of linear growth (i.e., proportional conditions) would mean inserting $L1U\rightarrow b \cdot L1U$ and $L2M\rightarrow b\cdot L2M$ into this equation. $(L2M{-}L1U)$ would then also scale linearly, and *b* cancels top and bottom throughout showing that $\varTheta$ would not change. In other words a linearly swollen/shrunken version of a particular chela has the same average velocity ratio over its mastication surface as before. Figure 14d shows the $\varTheta$ values for mesostigmatids falling to the left and right of the boundary (at $VR=0.276$) between cutting style killing types and crushing style killing types of carnivores as expected.

### Variance of velocity ratio

By definition (the population) *variance* of a sampled [*x*] $= E(x^2)-(E(x))^2$ (where *E* = expectation operator). So, the theoretical variance of the deterministic velocity ratio line function over the whole mastication surface ($L2M>L1U$) from the rise of the moveable digit basal process (i.e., at $x=L1U$) through to the moveable digit tip with respect to that beam-like *L*2*M* axis (*a*) is

$$\begin{aligned} Var[VR]=\varPhi ^{2}=\left[ \tfrac{1}{L2M{-}L1U}\cdot {\mathop {\int }\limits _{L1U}^{L2M}}(VR_{a})^{2}\text {d}a \right] - \varTheta ^{2} \end{aligned}$$

It can be evaluated by substitution into the formula from the Technical appendix

$$\begin{aligned} \frac{a^{2}}{U.L}-\varTheta ^{2} \end{aligned}$$

to give

2 $$\begin{aligned} Var[VR]=\varPhi ^{2} = \left[ \frac{L1U}{L2M}\right] -\varTheta ^{2} \end{aligned}$$

In other words the variance of the theoretical velocity ratio over the mastication surface ($\varPhi ^{2}$), when the moveable digit is considered as a plesiomorphic bar-like shape, equals the moveable digit tip velocity ratio minus the square of the expected velocity ratio over that mastication surface. This is illustrated in Fig. 48 where a relationship with *E*[*VR*] is shown approximating a cubic, first rising for very elongate low tip velocity ratio chelae, then falling as *L*1*U* dominates over *L*2*M*. Again it is stressed that these integrals are over a fully deterministic value not expectations over specimen-based sampled values each of which belongs to a probability distribution. Since it is derived from an assumption of a beam-like *L*2*M* axis, $\varPhi ^{2}$ is the null value for any test of a measured (i.e., observed or sampled) variance of the mastication surface velocity ratio when the moveable digit has been evolutionarily adjusted with teeth, gullets and blades.

### Expected results

Given the three concepts above, expected results can be derived and the physical advantage of actually having an ornamented moveable digit can be judged. This is because $\varTheta$ is the null value for any paired test of the (sampled) mean observed mastication surface velocity ratio (denoted *O*[*VR*]) for each specimen when the moveable digit surface has been adjusted with teeth, gullets and blades across individuals as follows.

Consider the *i*th feature in the dentition of a moveable digit ornamented mastication surface. With respect to the condyle-to-tip *L*2*M* axis, each feature defined by its co-ordinate $y_{i}$ (with its $x_{i}$) has its own velocity ratio. In each case the input moment arm *L*1*U* is fixed as the condyle position is constant. However, each $[x_{i},y_{i}]$ location forms a hypotenuse to the condyle with respect to the original *L*2*M* axis (e.g., length $L2M'$ in Fig. 2), so its velocity ratio is $VR'=\tfrac{L1U}{L2M'}$ and thus

$$\begin{aligned} O[VR]=\frac{1}{q} \cdot \sum _{i=1}^{i_{e}}[VR'_{i}] \end{aligned}$$

where *q* is the number of digitised $x_{i}$ locations along the *L*2*M* axis including the moveable digit tip and the end of the mastication surface. As the hypotenuse lengthens, the calculated velocity ratio declines. As the tip of the moveable digit moves vertically upward, features above the *L*2*M* axis engender a movement in grasped food material upwards and backwards (i.e., towards the condyle), features below the axis engender a movement in material upwards and forwards (i.e., towards the moveable digit tip—see Fig. 2 in Bowman 2023a). These features will then produce compressive and shearing forces within statically grasped food (or drive rotations in dynamically processed food particles).

Inspection of published illustrations (see Fig. 11) suggests that a variety of moveable digit locations in *Acarus siro*, *Chortoglyphus arcuatus*, and *Kuzinia laevis*, would therefore engender a force upwards and backwards on chelal closure. Evolutionarily, they represent digit surface areas of developmental ‘stretching’, or ‘rotational flexing’ or morphological ‘creeping/shearing’ of any originally beam-like moveable digit surface (where their positivity means an upwards change).

If the moveable digit features are significant asperities above, or gullets below, the *L*2*M* axis, averaging these $VR'$ values (i.e., ignoring the direction of the adductive force) over the mastication surface should always produce a value noticeably different than assuming a simple bar shape for the moveable digit. For the example in Fig. 2 of Bowman (2023a), $\varTheta =0.644$ versus an $O[VR]=0.670$ (with a moveable digit tip $VR=0.439$). The difference between the actual average velocity ratio of an ornamented moveable digit surface and the theoretical expected value without ornamentation for a digit of that tip velocity ratio and mastication surface length is an objective measure of the masticatory efficiency advantage of having chelal teeth, gullets and blades for that astigmatid. As it is a measured value it will have sampling variation that must be dealt with in an appropriate manner.

## Technical appendix

The velocity ratio $\frac{L1U}{L2M}$ as a function of position along bar-like beam of length *L*2*M* rotating at the origin can be represented generically as $y=\tfrac{a}{x}$ where *a* is a constant ($\equiv L1U$). The first derivative is then $\tfrac{dy}{dx}=\frac{-a}{x^{2}}$ which is $<0\ \forall \ x$ i.e., $y=\tfrac{a}{x}$ is monotonic decreasing. The second derivative is then $\tfrac{d^{2}y}{dx^{2}}=\frac{2a}{x^{3}}$ which is $>0\ \forall \ x$ as $y=\tfrac{a}{x}$ is concave.

Define $L={lower} \,{limit}$ and $U={upper}\, {limit}$ of the mastication surface along the beam, then the average values ($\equiv E[y]$) over the mastication surface for each of these three velocity ratio functions *y*, $\tfrac{dy}{dx}$ and$\tfrac{d^{2}y}{dx^{2}}$ is given by

$$\begin{aligned}{} & {} \frac{1}{U-L}\cdot {\mathop {\int }\limits _{L}^{U}} y\ \text {d}x=\frac{a}{U-L}\cdot {\mathop {\int }\limits _{L}^{U}} \frac{1}{x}\ \text {d}x=\frac{a}{U-L}\cdot log_{e}x \Big |_{L}^{U}=\frac{a}{U-L}\cdot log_{e}\frac{U}{L}\\{} & {} \frac{1}{U-L}\cdot {\mathop {\int }\limits _{L}^{U}} \frac{dy}{dx}\ \text {d}x=\frac{-a}{U-L}\cdot {\mathop {\int }\limits _{L}^{U}} \frac{1}{x^{2}}\ \text {d}x=\frac{a}{U-L}\cdot \frac{1}{x} \Big |_{L}^{U}=\frac{a}{U-L}\cdot \frac{L-U}{U.L}=\frac{-a}{U.L}\\{} & {} \frac{1}{U-L}\cdot {\mathop {\int }\limits _{L}^{U}} \frac{d^{2}y}{dx^{2}}\ \text {d}x=\frac{2.a}{U-L}\cdot {\mathop {\int }\limits _{L}^{U}} \frac{1}{x^{3}}\ \text {d}x=\frac{-a}{U-L}\cdot \frac{1}{x^{2}} \Big |_{L}^{U}=\frac{a}{U-L}\cdot \frac{U^{2}-L^{2}}{U^{2}.L^{2}} \end{aligned}$$

respectively. Think of the $\tfrac{1}{U-L}$ term to be like $\tfrac{1}{n}$ in the discrete case and the integral to be like the sum $\sum _{i=1}^{n}$.

These formulae can simplify further as $a=L1U$ and if *L*1*U* is the lower limit of definite integration i.e., $L=L1U$. Then *L*2*M* is taken to be *U*.

The average squared value for *y* ($\equiv E[y^{2}]$) over the mastication surface is given by

$$\begin{aligned} \frac{1}{U-L}\cdot {\mathop {\int }\limits _{L}^{U}} y\cdot y\ \text {d}x= & {} \frac{a^{2}}{U-L}\cdot {\mathop {\int }\limits _{L}^{U}} \frac{1}{x^{2}}\ \text {d}x=\frac{-a^{2}}{U-L}\cdot \frac{1}{x}\\ \Big |_{L}^{U}= & {} \frac{a^{2}}{U-L}\cdot \frac{U-L}{U.L}=\frac{a^{2}}{U.L} \end{aligned}$$

The average squared value for $\frac{dy}{dx}$ over the mastication surface is given by

$$\begin{aligned} \frac{1}{U-L}\cdot {\mathop {\int }\limits _{L}^{U}} \frac{dy}{dx}\cdot \frac{dy}{dx}\ \text {d}x=\frac{a^{2}}{U-L}\cdot {\mathop {\int }\limits _{L}^{U}} \frac{1}{x^{4}}\ \text {d}x=\frac{-a^{2}}{3.(U-L)}\cdot \frac{1}{x^{3}} \Big |_{L}^{U}=\frac{a^{2}}{3.(U-L)}\cdot \frac{U^{3}-L^{3}}{U^{3}.L^{3}} \end{aligned}$$

The average squared value for $\frac{d^{2}y}{dx^{2}}$ over the mastication surface is given by

$$\begin{aligned} \frac{1}{U-L}\cdot {\mathop {\int }\limits _{L}^{U}} \frac{d^{2}y}{dx^{2}} \cdot \frac{d^{2}y}{dx^{2}}\ \text {d}x=\frac{2^{2}.a^{2}}{U-L}\cdot {\mathop {\int }\limits _{L}^{U}} \frac{1}{x^{6}}\ \text {d}x=\frac{-4.a^{2}}{5.(U-L)}\cdot \frac{1}{x^{5}} \Big |_{L}^{U}=\frac{4a^{2}}{5.(U-L)}\cdot \frac{U^{5}-L^{5}}{U^{5}.L^{5}} \end{aligned}$$

respectively.

These formulae can simplify further as $a=L1U$ and if *L*1*U* is the lower limit of definite integration i.e., $L=L1U$. Then *L*2*M* is taken to be *U*.

Taking these averages values as expected values (*E*[...]), then the variance (*Var*) of the three velocity ratio functions *y*, $\tfrac{dy}{dx}$ and$\tfrac{d^{2}y}{dx^{2}}$ over the mastication surface can be calculated as $E(...^2)-(E(...))^2$ (where *E* = expectation operator). That is,

$$\begin{aligned}{} & {} \left[ \frac{a^{2}}{U\cdot L}\right] - \left[ \frac{a}{U-L}\cdot log_{e}\frac{U}{L} \right] ^{2}\\{} & {} \left[ \frac{a^{2}}{3\cdot (U-L)}\cdot \frac{U^{3}-L^{3}}{U^{3}\cdot L^{3}}\right] - \left[ \frac{-a}{U\cdot L} \right] ^{2}\\{} & {} \left[ \frac{4a^{2}}{5\cdot (U-L)}\cdot \frac{U^{5}-L^{5}}{U^{5}\cdot L^{5}}\right] - \left[ \frac{a}{U-L}\cdot \frac{U^{2}-L^{2}}{U^{2}\cdot L^{2}} \right] ^{2} \end{aligned}$$

respectively.

These can simplify further as $a=L1U$ and if *L*1*U* is the lower limit of definite integration i.e., $L=L1U$. Then *L*2*M* is taken to be *U*.

In particular the first equation of the latter three means that

$$\begin{aligned}{} & {} var(VR\ of\ mastication\ surface)\\{} & {} =VR_{tip}-(average\ VR\ of\ mastication\ surface)^{2} \end{aligned}$$

The first term in [....] of the last equation of the three above is the numerator of the theoretical bending energy of the mastication velocity ratio surface.

## Abbreviations

*Ar*: Ascending ramus of coronoid process
$c_{i}$: Profile curvature at *i*th semi-landmark
$\varDelta$: “Change in...” (e.g., change in volume $\varDelta V$)
$\delta$: Moveable digit subtended angle
$\delta ^{\ast}$: Adjusted moveable digit subtended angle
$\eta _{p}$: Number of peaks
$\eta _{v}$: Number of valleys
$\gamma$: ‘Claw’ or ‘hook’ angle
$\lambda c$: Roughness wavelength cutoff
$\varPhi ^2$: Theoretical velocity ratio variance
$$\sigma ^2$$: Tribological variance
$\varTheta$ or $\theta$: Theoretical expected variance ratio
*BE*: Bending energy
*CHI*: Cheliceral height index
*CLE*: Claw length equivalent
*CLI*: Reach or cheliceral length index
*E*: Expected or Expectation
*E*[*F*2]: Expected chelal crunch force
*E*[*VR*]: Expected velocity ratio (EVR)
*f*: Flexure measure
*F*1: Chelal adductive input lever arm muscular force ($PHI^2 \equiv CHI^2$ in Bowman 2021b)
*F*1*AV*: Chelal adductive input lever arm muscular force in Bowman (2021b)
*F*2: Occluding chelal crunch force (*VR*.*F*1 in Bowman 2021b)
*F*2*AV*: Occluding chelal crunch force (*VR*.*F*1*AV* in Bowman 2021b)
*G*: Maximum effective chelal gape
$G_{I}$: Maximum effective gape at semi-landmark *i*
$g_{i}$: Profile gradient at *i*th semi-landmark
*h*: Shear or creep measure
*Hr*: Horizontal ramus (i.e., potential tooth row)
*IL*: Idiosomal length index
*L*: Lyrifissure to fixed digit tip distance
*L*1*U*: Adductive input moment lever arm
*L*2*M*: Adductive output moment lever arm
$L2M_{i}$: Output moment lever arm at location $x_{i}$
*m*: Length of mastication surface (‘drape’ or ‘chain’ distance) over asperities/gullets (consequence of stretch)
$m_c$: Consequence of creep
$m_f$: Consequence of flexure
$M_{vG}$: Maximum volume of a chela bite of ‘grab’ ($mgf_{i}$ for *i*th semi-landmark)
*MDL*: Moveable digit length (or ‘Gape’ approximation)
$N_{0}$: Number of times the profile crosses the average value
*O*[*VR*]: Observed average velocity ratio (OVR)
*Ov*[*VR*]: Observed variance of velocity ratio (OvVR)
*Ov*[*F*2]: Observed variance of occlusive chelal crunch force
$pocket\ E[F2]$: Average crunch force (*F*2) of modelled pocket on moveable digit
$pocket\ E[VR]$: Average velocity ratio of modelled pocket on moveable digit
*r*: Correlation coefficient (statistical use)
*rel*: Relative elongation
$R^{2}$: Percentage variation explained
$R_{a}$: Tribological average absolute deviation, mean surface roughness
$$r_c$$: Relative creep
$$r_f$$: Relative flexure
$R_{p}$: Distance between highest asperity and average, highest peak (at $x_{Rp}$)
$R_{p}[F2]$: Crunch force at maximum tooth peak
$R_{p}[VR]$: Velocity ratio at maximum peak of dentition
$R_{q}$: Tribological *Root Mean Square*
$R_{t}$: Distance from highest peak to lowest valley
$R_{v}$: Distance between average and lowest valley, lowest gullet (at $x_{Rv}$)
*s*: Smoothness (i.e., variance of curvature over mastication surface)
SEM: Scanning electron microscopy
*tel*: Stretch measure
*Tj*: Tendon junction with basal ramus of coronoid process
$TM_{vG}$: Food fragment truncated volume
*Var*: Variance
*Var*[*VR*]: Variance of velocity ratio
*VR*: Adductive moveable digit lever arm velocity ratio $\frac{L1U}{L2M}$ (or mechanical advantage Bowman 2021b)
$VR_{i}$: Adductive moveable digit lever arm velocity ratio at location $x_{i}$
$VR_{tip}$: Velocity ratio of moveable digit tip
$x_{1}$: Moveable digit tip (i.e., zero)
$x_{i_{e}}$: End of mastication surface (*e*) distance along *L*2*M* axis towards condyle
$x_{Rp}$: Position of max peak along *L*2*M* axis
